# Supplementary material for: Discovery of a potent HMG-CoA reductase degrader that eliminates statin-induced reductase accumulation and lowers cholesterol
Source: Nat Commun. 2018 Dec 3;9:5138. doi: 10.1038/s41467-018-07590-3 (PMC6277434; doi:10.1038/s41467-018-07590-3)
Supplement: Supplementary file 1 — Supplementary Information [file 41467_2018_7590_MOESM1_ESM.pdf]

SUPPLEMENTARY INFORMATION

Discovery of a potent HMG-CoA reductase degrader that eliminates statin-induced reductase accumulation and lowers cholesterol

Jiang et al.

Supplementary Figures

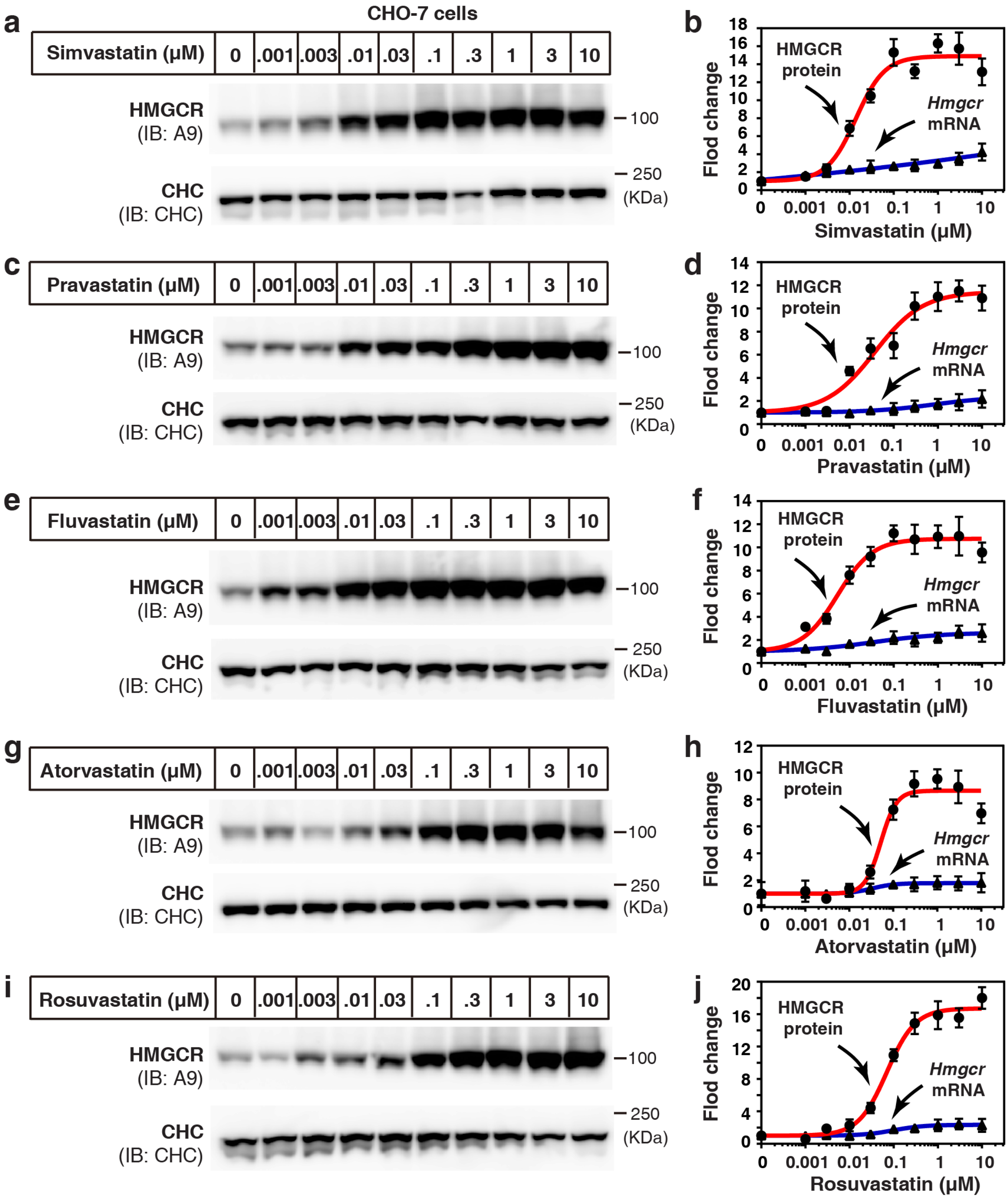

Supplementary Figure 1. Statins induce HMGCR protein accumulation.

a-**j** CHO-7 cells were treated with indicated concentrations of statins (simvastatin, pravastatin, fluvastatin, atorvastatin and rosuvastatin) for 16 h, and then harvested for immunoblotting (**a**, **c**, **e**, **g**, **i**) and RT-qPCR analysis (**b**, **d**, **f**, **h**, **j**). The HMGCR protein level and *Hmgcr* mRNA level in DMSO-treated cells were defined as 1, respectively. Data are from 3 independent experiments and presented as mean ± SD. Uncropped immunoblots are shown in **Supplementary Figure 11**.

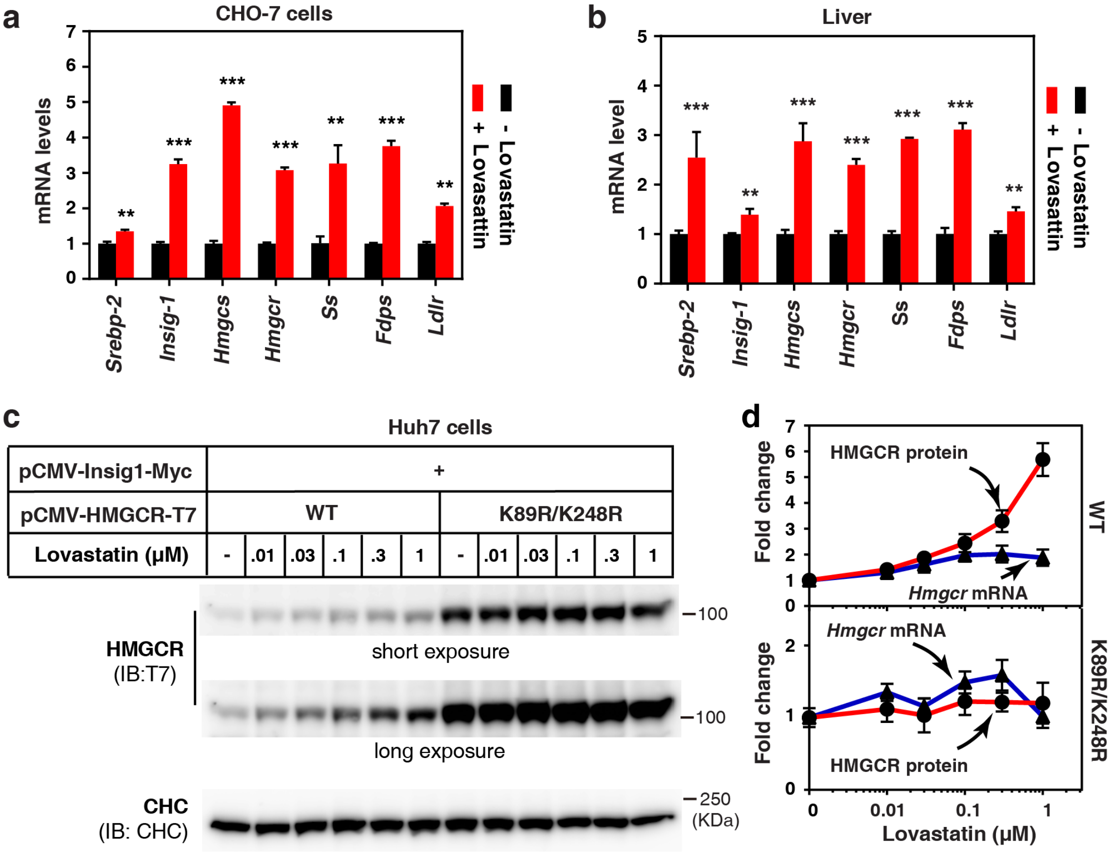

Supplementary Figure 2. Lovastatin drastically increases HMGCR protein level. **a** CHO-7 cells were treated with or without 1 μM lovastatin in medium B with 5% LDPS and 50 μM mevalonate for 16 h, and harvested for total RNA isolation and RT-qPCR. Quantifications of the relative mRNA levels of indicated SREBP target genes are shown. *Gapdh* was the referene gene. The relative mRNA abundance in DMSO-treated cells was defined as 1. **b** Quantifications of the relative mRNA levels of indicated SREBP target genes in the livers of mice treated without (-) or with (+) 60 mg/kg/day lovastatin (n = 3 mice per group). *Cyclophilin* was the reference gene. The relative mRNA abundance in vehicle-treated mice was defined as 1. **c-d** Huh7 cells were transfected with indicated plasmids and treated with indicated concentrations of lovastatin. The exogenous wild-type (WT) and mutant HMGCR protein and mRNA levels were analyzed by immunoblotting and RT-qPCR. Data are from 3 independent experiments and presented as mean ± SD. **P < 0.01, ***P < 0.001, unpaired two-tailed Student's *t*-test. Uncropped immunoblots are shown in **Supplementary Figure 11**.

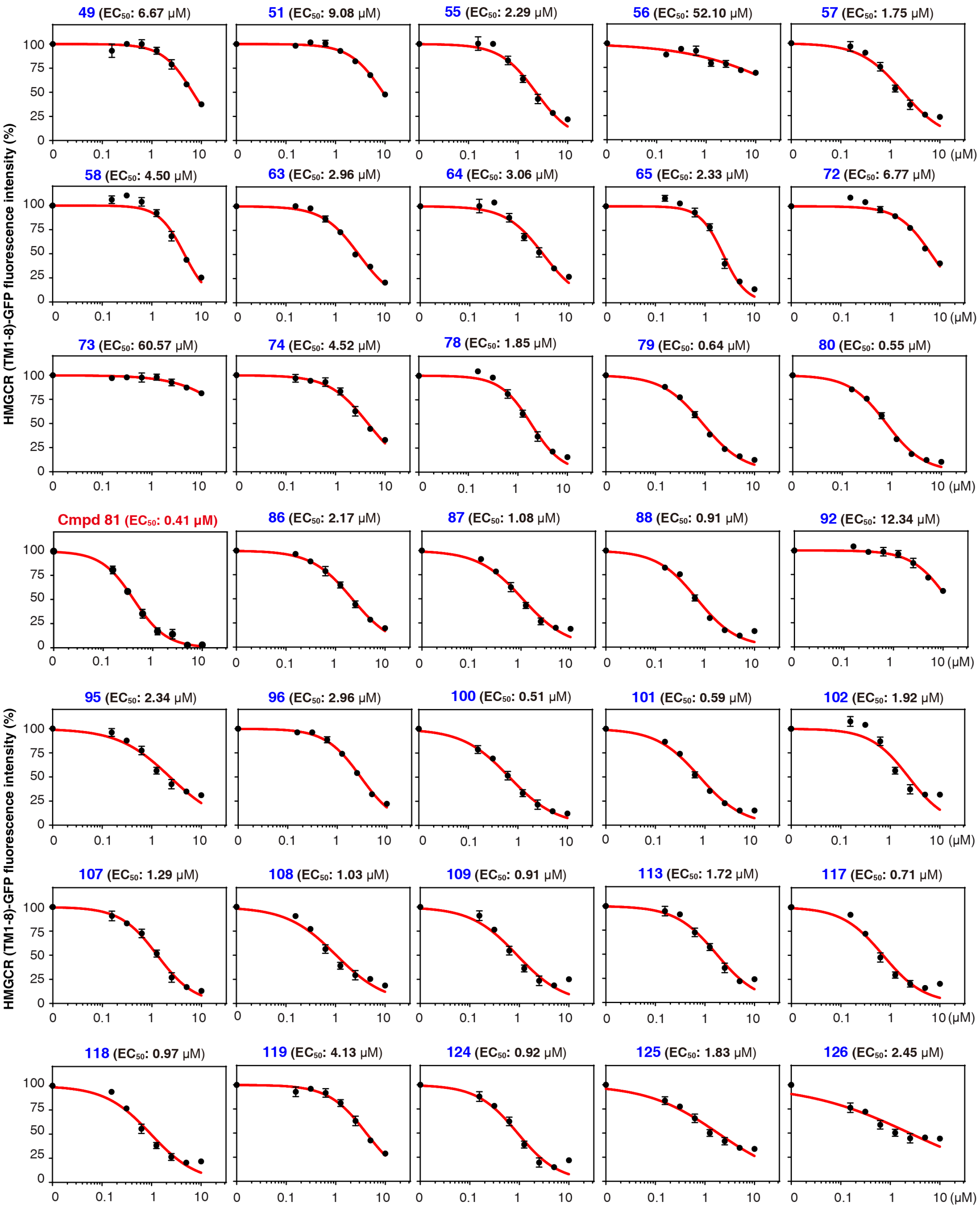

Supplementary Figure 3. Dose-response curves of indicated compounds. CHG cells were incubated with indicated compounds at increasing concentrations up to 10 μM for 16 h. Cells were then fixed for immunofluorescence analysis. The GFP intensity of DMSO-treated cells was defined as 100. Compound names and the EC_{50} values are indicated above each curve. The most potent Cmpd 81 is highlighted in red. The EC_{50} values were determined with dose-response curves by Prism software, and summarized in **Figure 4**. Data are from 3 independent experiments and presented as mean ± SD.

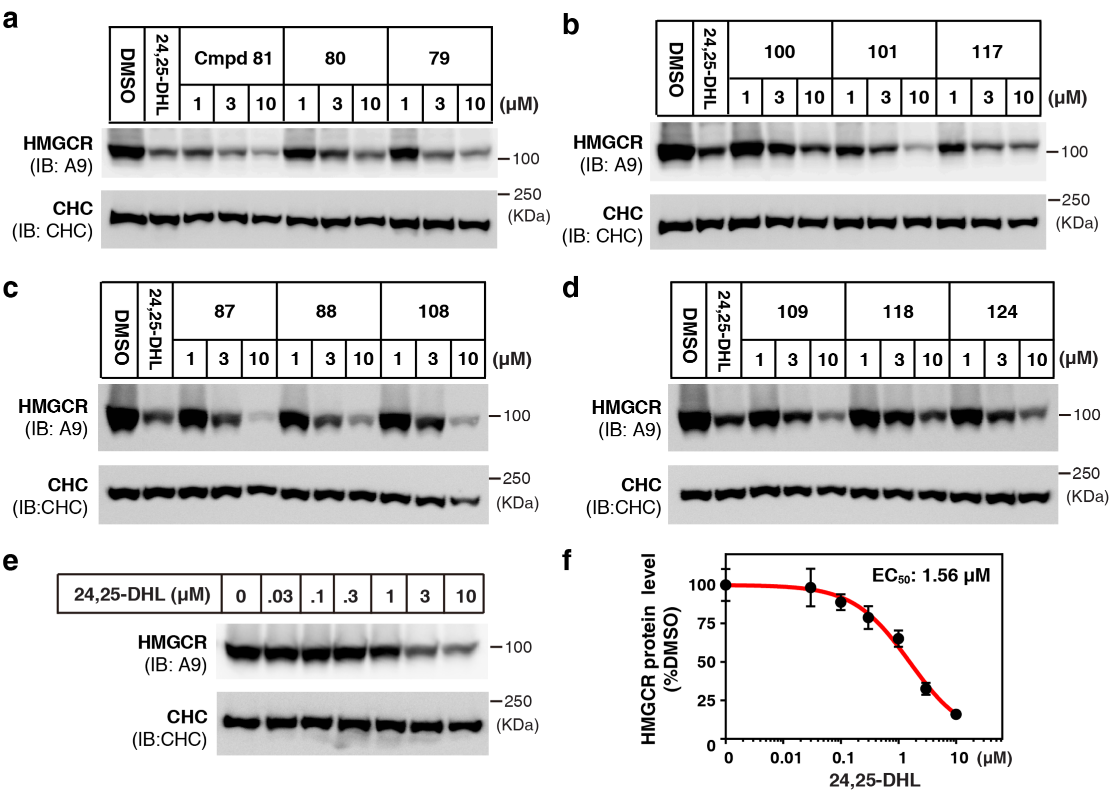

Supplementary Figure 4. Compounds that promote HMGCR degradation. a-d CHO-7 cells were incubated with indicated compounds for 16 h, and then harvested for immunoblotting analysis of endogenous HMGCR protein (IgG-A9). 3 μM 24,25-DHL was used as a control. **e-f** CHO-7 cells were incubated with varying concentrations of 24,25-DHL for 16 h. Immunoblotting analysis (**e**) and dose-response curve (**f**) of endogenous HMGCR protein in response to varying concentrations of 24,25-DHL are shown. The mean HMGCR protein level in DMSO-treated cells was defined as 100. The EC_{50} value of 24,25-DHL was 1.6 μM. Data are from 3 independent experiments and presented as mean ± SD. Uncropped immunoblots are shown in **Supplementary Figure 12**.

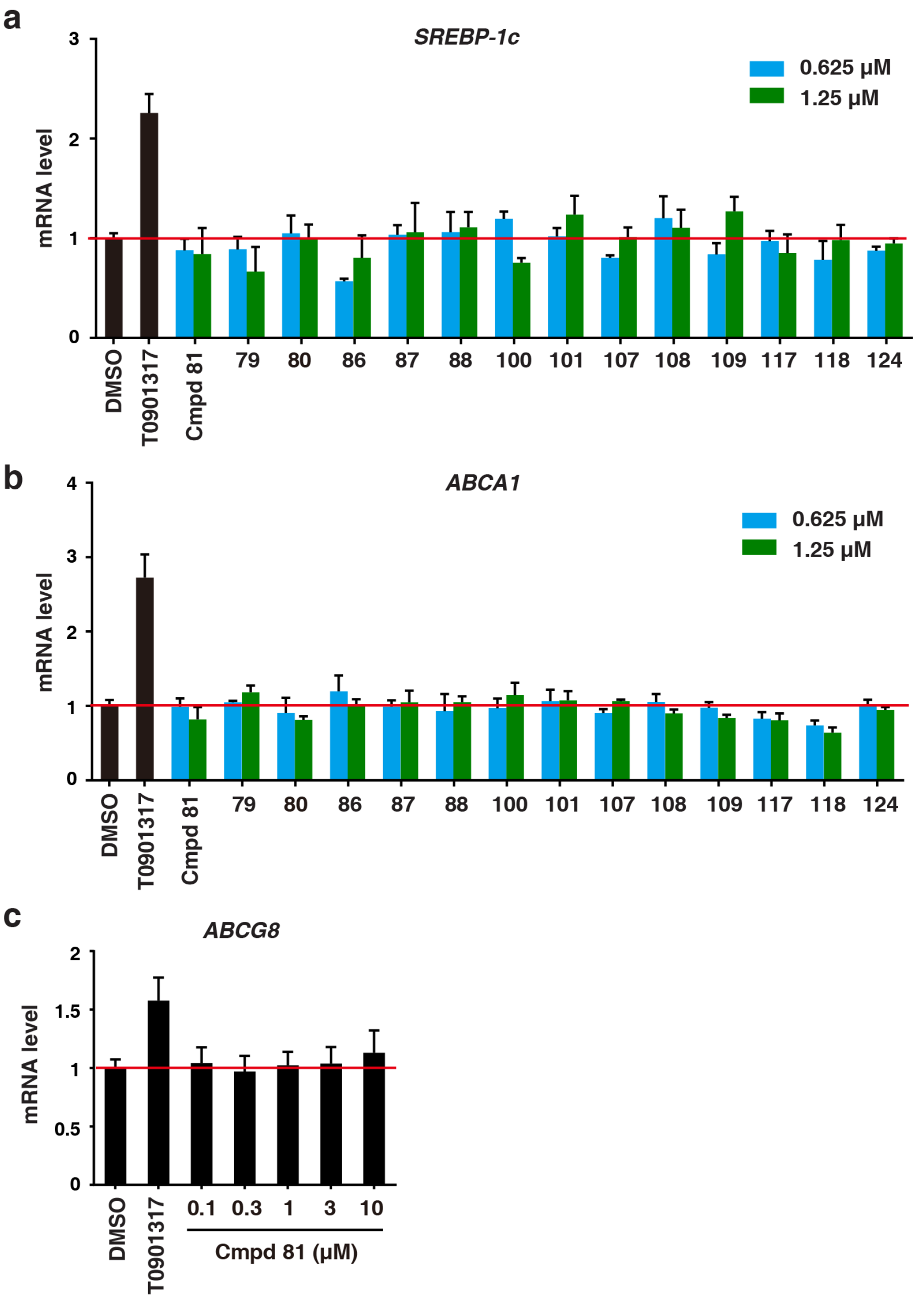

Supplementary Figure 5. Cmpd 81 and its analogs do not activate the LXR pathway. a-c Huh-7 cells were treated with indicated compounds for 16 h and then harvested for total RNA isolation and RT-qPCR. Cells treated with LXR agonist T0901317 were used as positive controls. The relative mRNA levels of *SREBP-1c* (**a**), *ABCA1* (**b**) and *ABCG8* (**c**) were quantified. *GAPDH* was the reference gene. The relative mRNA level in DMSO-treated cells was defined as 1. Data are from 3 independent experiments and presented as mean ± SD.

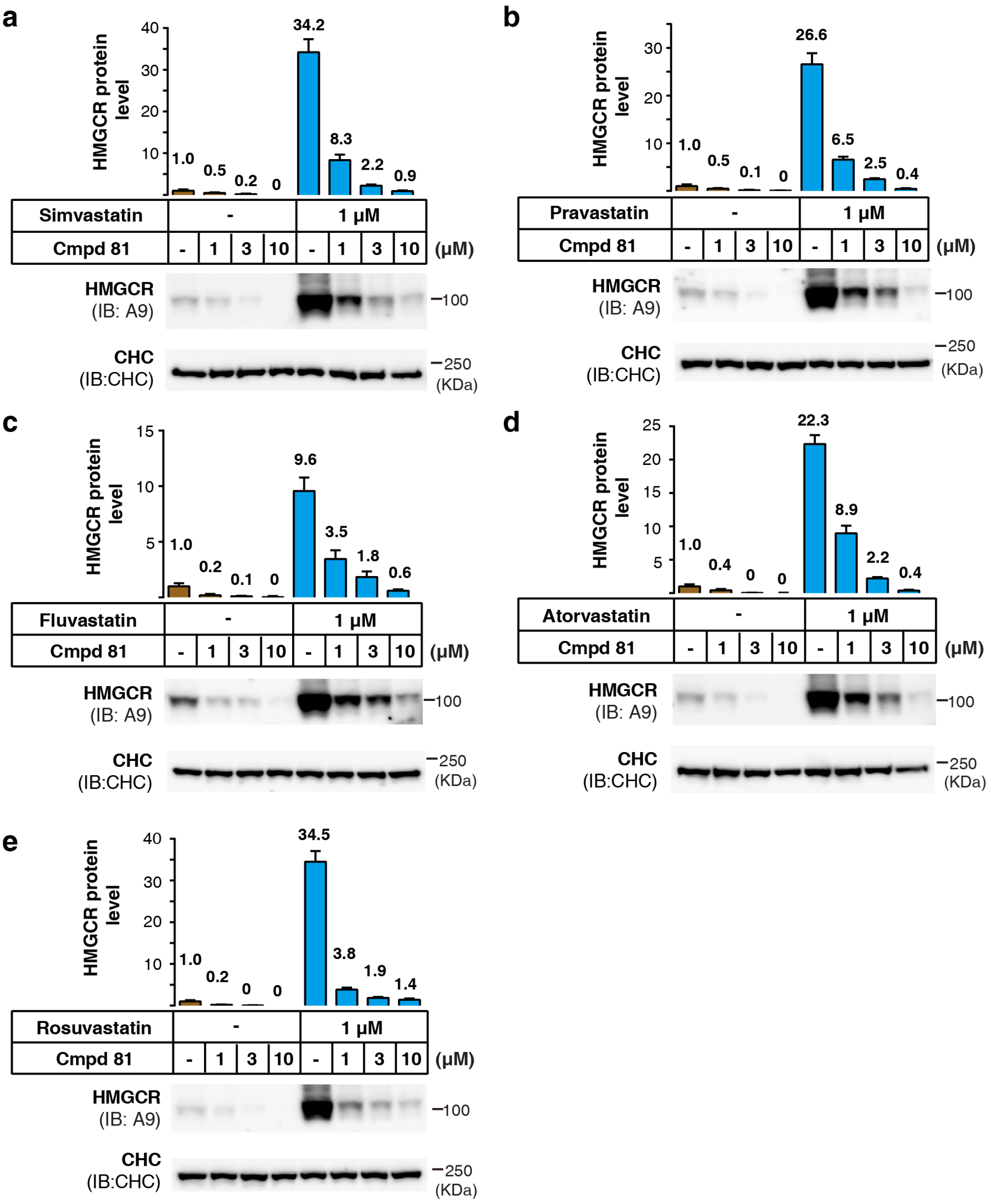

Supplementary Figure 6. Cmpd 81 prevents statin-induced HMGCR accumulation. a-e CHO-7 cells were treated with indicated concentrations of Cmpd 81 together with or without simvastatin, pravastatin, fluvastatin, atorvastatin, or rosuvastatin for 16 h, and then harvested for immunoblotting. Data are from 3 independent experiments and presented as mean ± SD. Uncropped immunoblots are shown in **Supplementary Figure 13**.

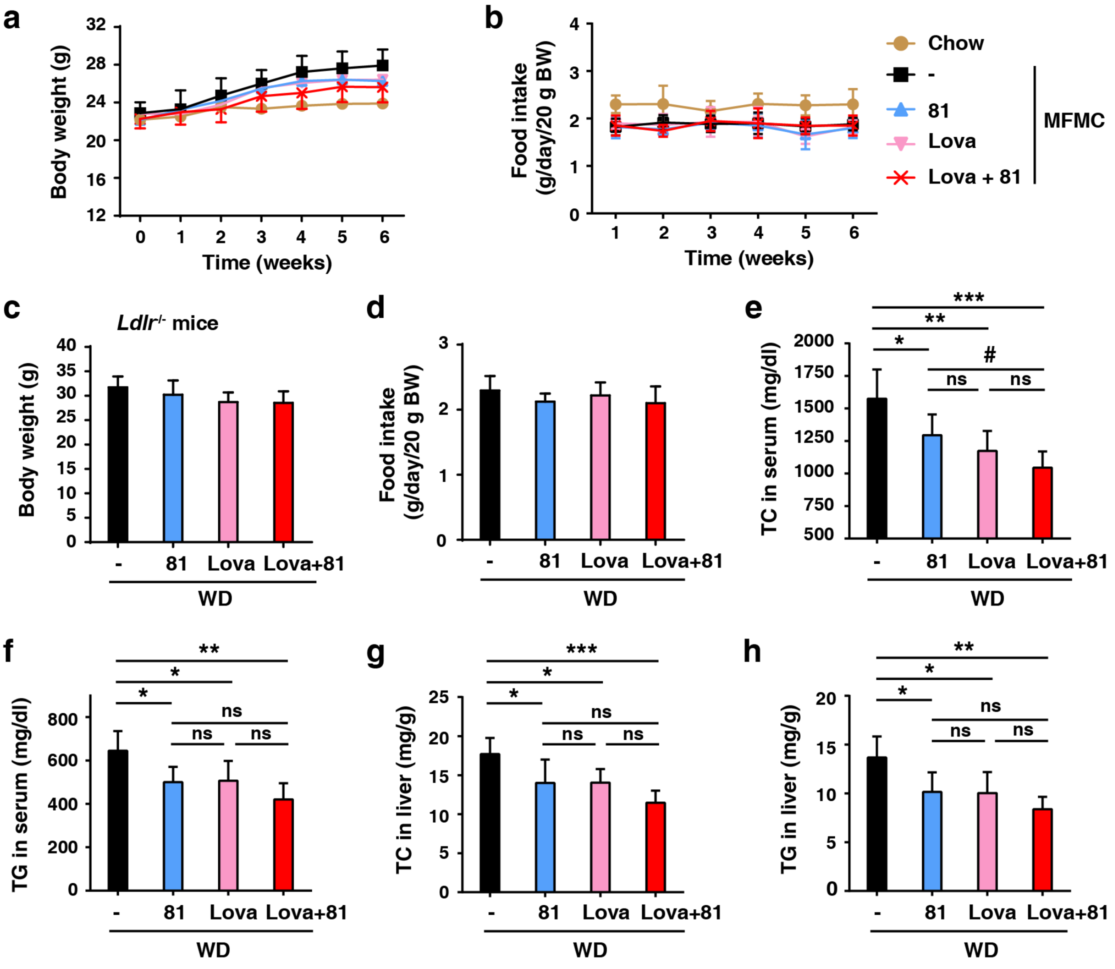

Supplementary Figure 7. The effects of Cmpd 81 on mouse lipid profile. a-b Male C57BL/6J mice (n = 5 per group) were randomly grouped and allowed *ad libitum* access to water and chow diet or MFMC diet. Mice were then gavaged with vehicle, Cmpd 81 (60 mg/kg/day), lovastatin (60 mg/kg/day), or both for 6 weeks. Body weight (**a**) and food intake (**b**) were examined during the 6-week experiments. **c-h** Male *Ldlr*$^{-/-}$ mice (n = 5 per group) fed on WD were gavaged with vehicle, Cmpd 81 (60 mg/kg/day), lovastatin (60 mg/kg/day), or both for 20 weeks. Body weight (**c**) and food intake (**d**) were examined during the 20-week experiments. Mice were sacrificed and examined for TC levels in the serum (**e**), TG levels in the serum (**f**), TC levels in the liver (**g**), and TG levels in the liver (**h**). Data are presented as mean ± SD. Asterisks in (**e-h**) indicate the differences between mice gavaged with vehicle and those with Cmpd 81 and lovastatin, $^{*}P < 0.05$, $^{**}P < 0.01$, $^{***}P < 0.001$; hash symbols indicate the differences between mice gavaged with both lovastatin and Cmpd 81 and those with either lovastatin or Cmpd 81, $^{\#}P < 0.05$, ns, not significant, one-way ANOVA followed by Dunnett's multiple comparisons test.

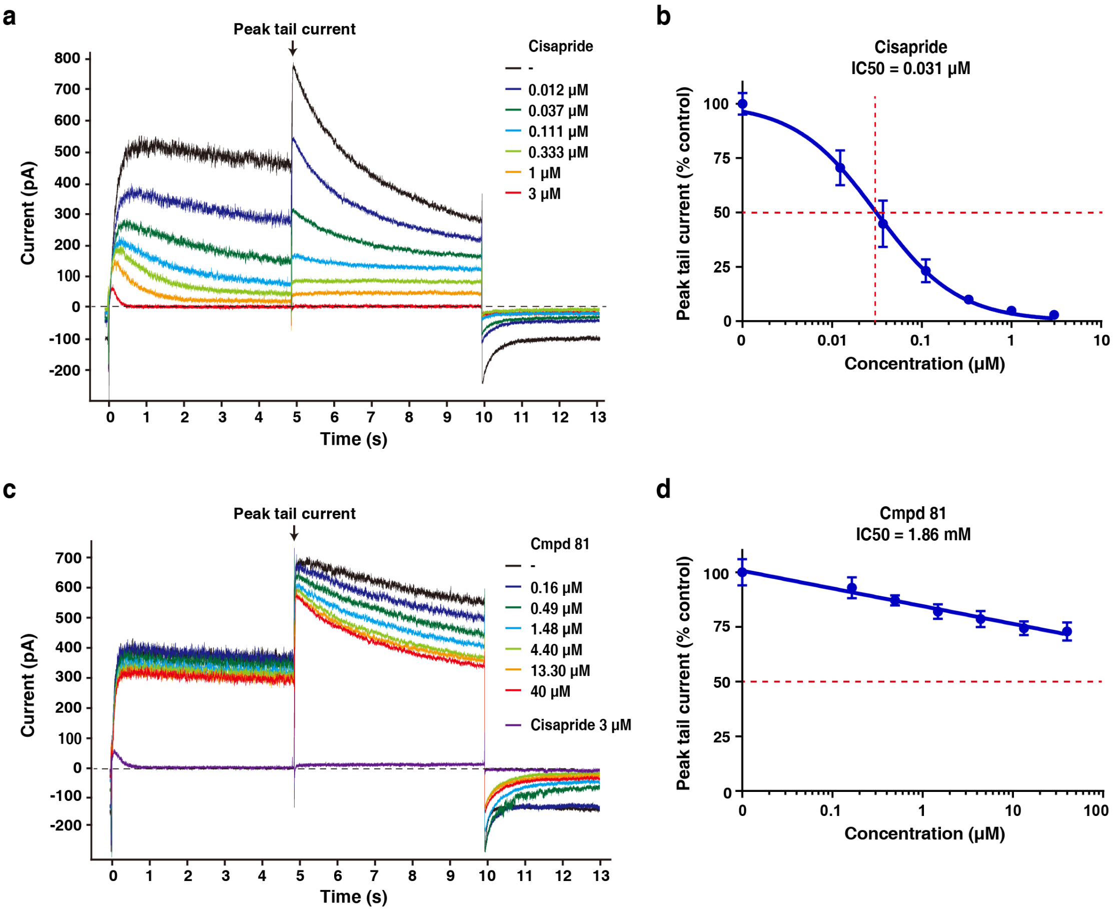

Supplementary Figure 8. The effects of Cisapride and Cmpd 81 on the current of hERG potassium channel. a Sweep plot assessment of Cisapride on the current of hERG potassium channel. Each trace represents the average of three measurements. **b** Dose-response curve of peak tail current values versus concentrations of Cisapride in **a**. Data are presented as mean ± SD and the IC_{50} value was determined by GraphPad Prism 6 software. **c** Sweep plot assessment of Cmpd 81 on the hERG channel. **d** Dose-response curve of peak tail current values versus concentrations of Cmpd 81.

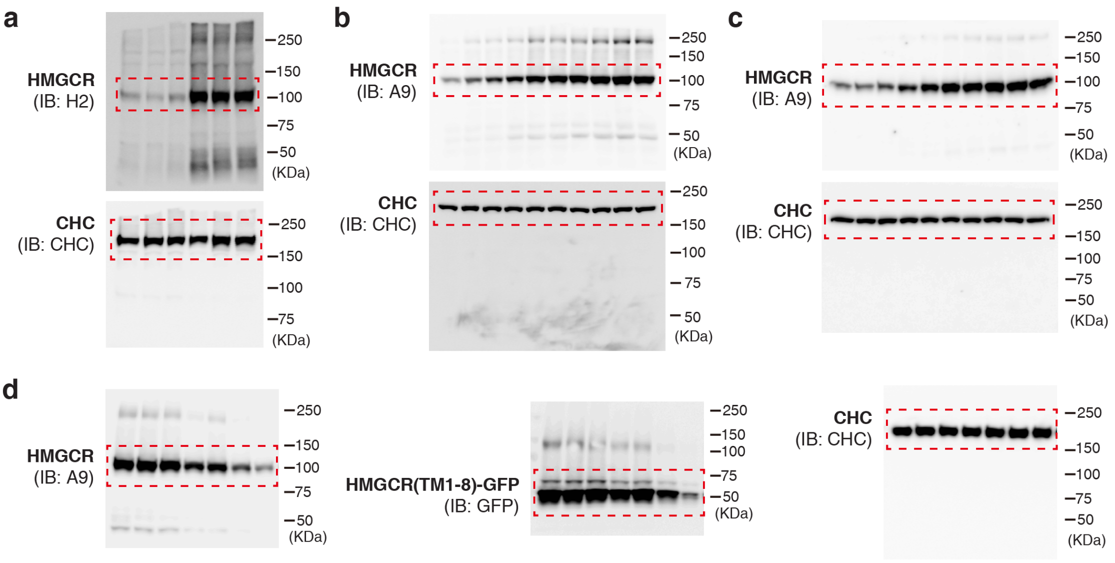

Supplementary Figure 9. Uncropped immunoblots for indicated Figures. a for Figure 1a. **b** for Figure 1d. **c** for Figure 1f. **d** for Figure 2b.

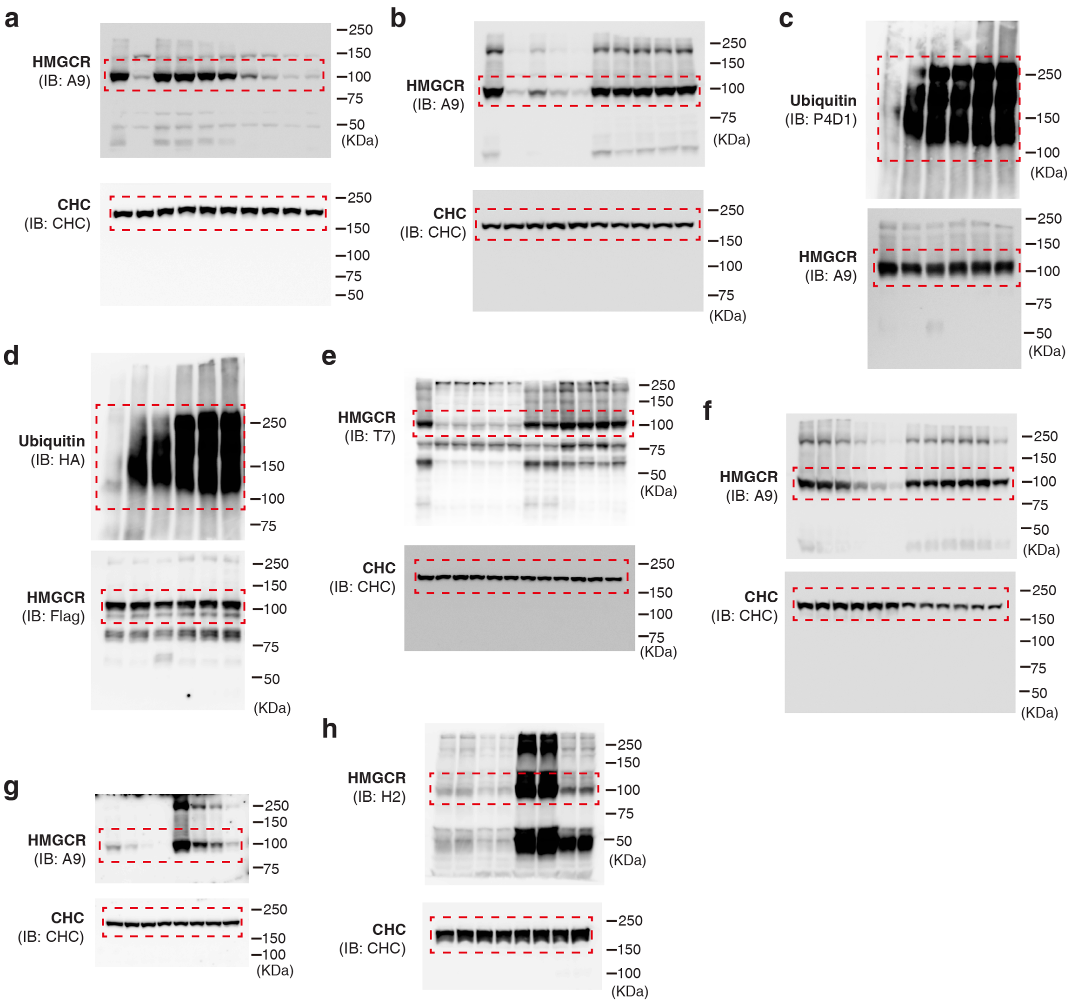

Supplementary Figure 10. Uncropped immunoblots for indicated Figures. a for Figure 5a. **b** for Figure 5c. **c** for Figure 5d. **d** for Figure 5e. **e** for Figure 5f. **f** for Figure 5g. **g** for Figure 6a. **h** for Figure 6b.

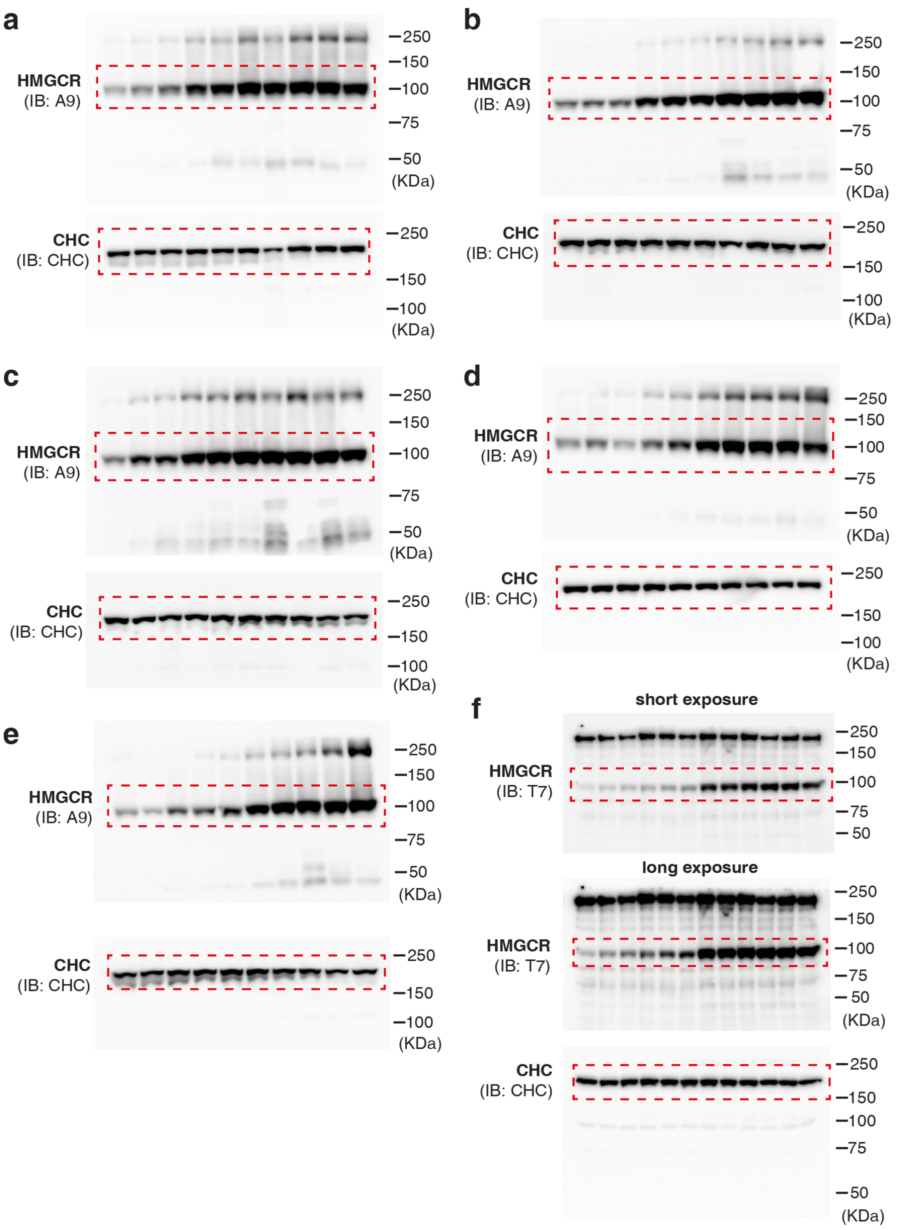

Supplementary Figure 11. Uncropped immunoblots for indicated Supplementary Figures. **a** for Supplementary Figure 1a. **b** for Supplementary Figure 1c. **c** for Supplementary Figure 1e. **d** for Supplementary Figure 1g. **e** for Supplementary Figure 1i. **f** for Supplementary Figure 2c.

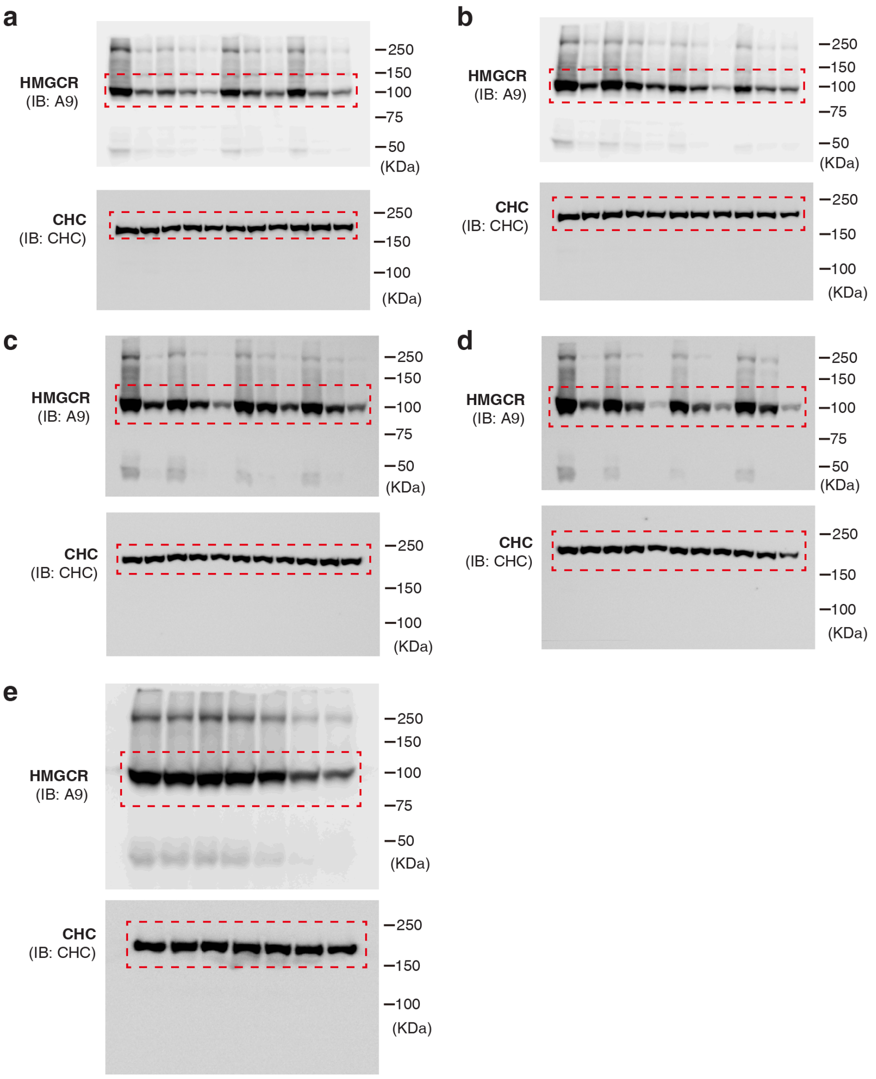

Supplementary Figure 12. Uncropped immunoblots for indicated Supplementary Figures. a for Supplementary Figure 4a. **b** for Supplementary Figure 4b. **c** for Supplementary Figure 4c. **d** for Supplementary Figure 4d. **e** for Supplementary Figure 4e.

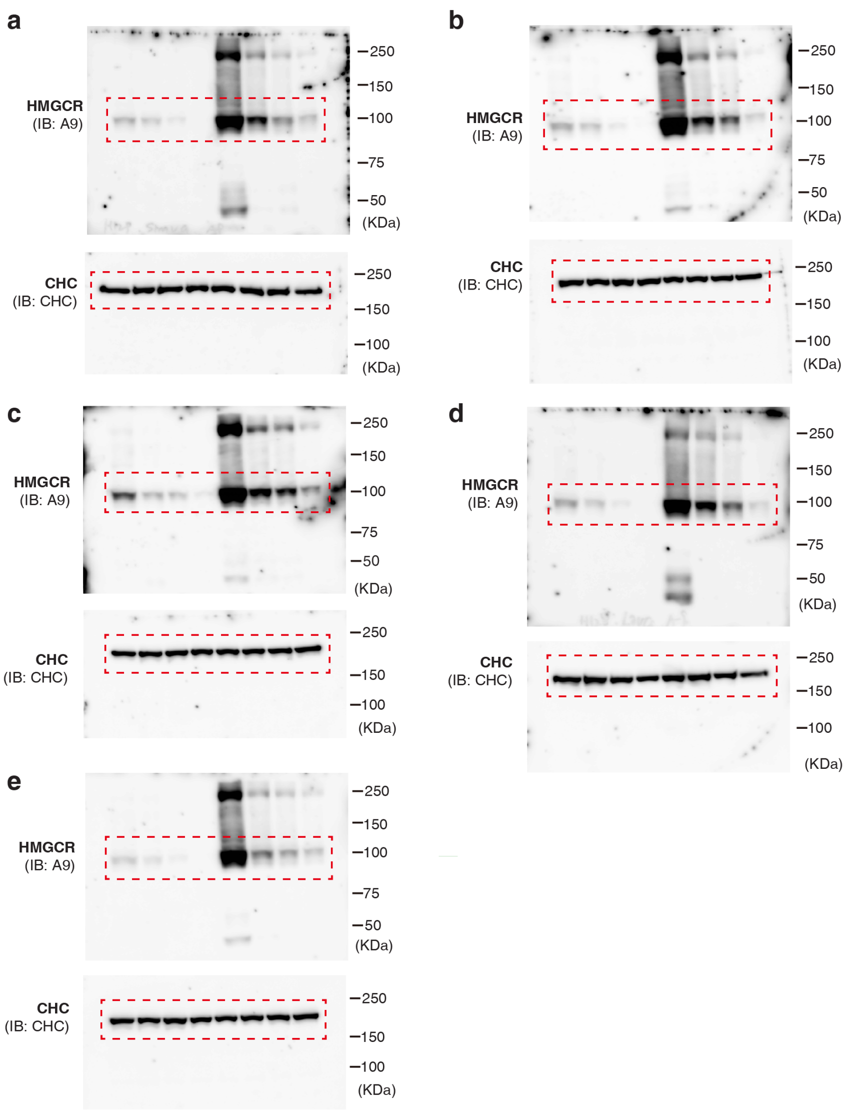

Supplementary Figure 13. Uncropped immunoblots for indicated Supplementary Figures. a for Supplementary Figure 6a. **b** for Supplementary Figure 6b. **c** for Supplementary Figure 6c. **d** for Supplementary Figure 6d. **e** for Supplementary Figure 6e.

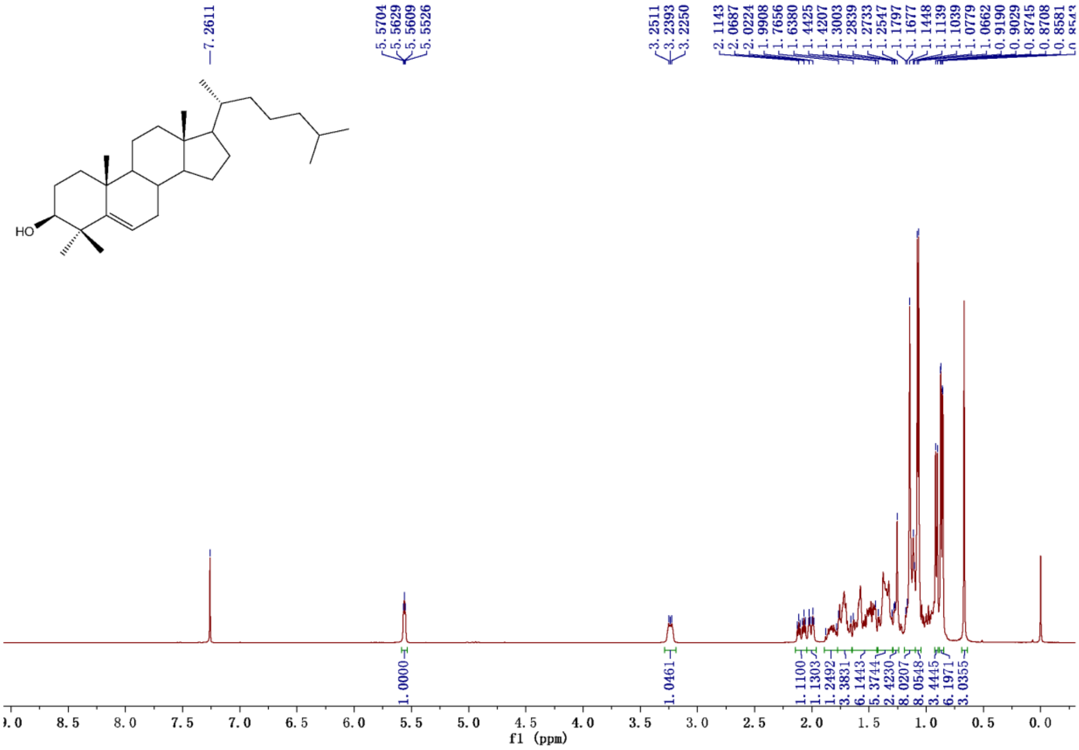

Supplementary Figure 14. Compound 7: ^{1}H-NMR (400 MHz, $CDCl_3$)

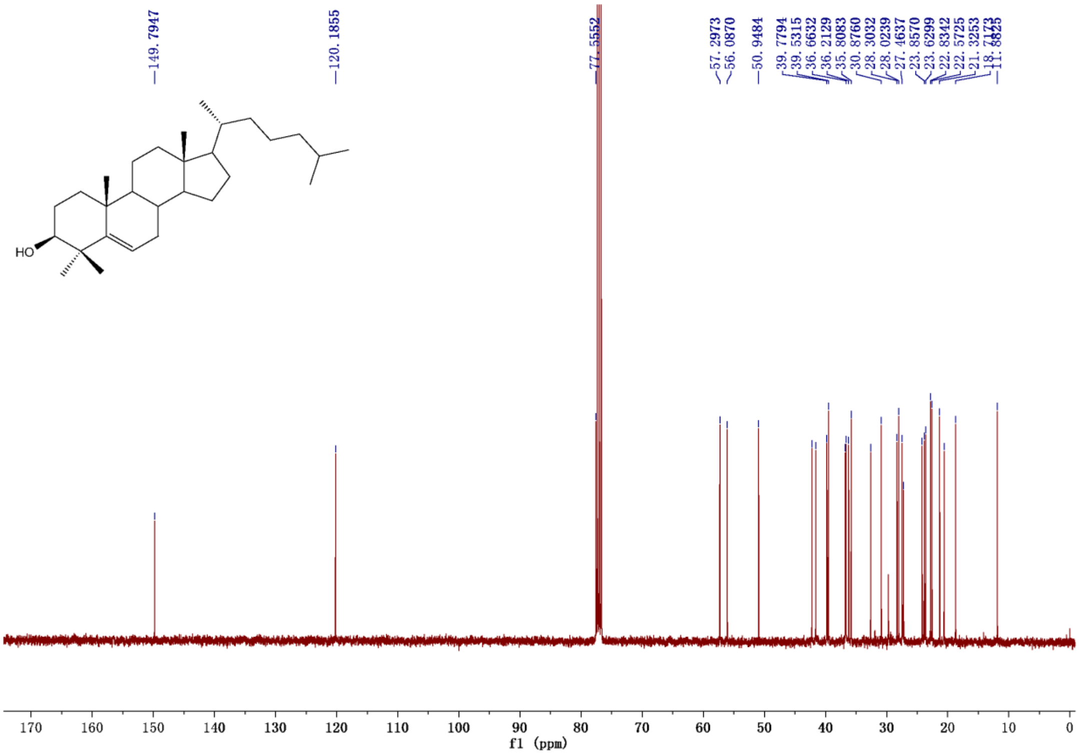

Supplementary Figure 15. Compound 7: ^{13}C-NMR (100 MHz, $CDCl_3$)

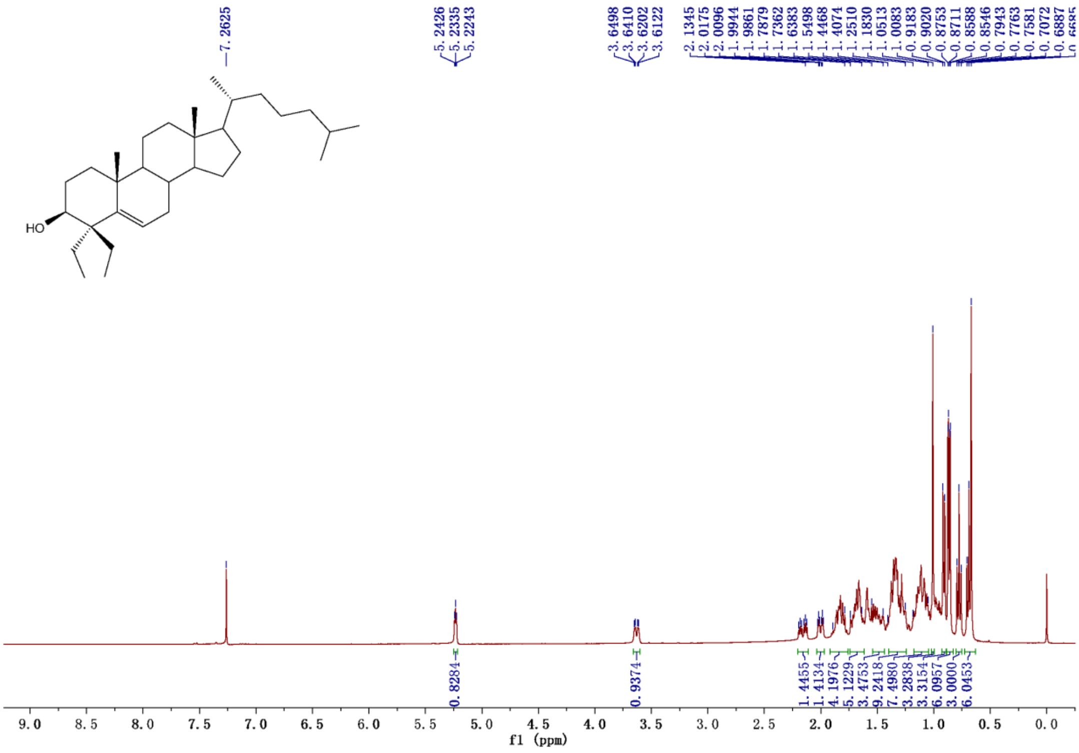

Supplementary Figure 16. Compound 8: ^{1}H-NMR (400 MHz, $CDCl_3$)

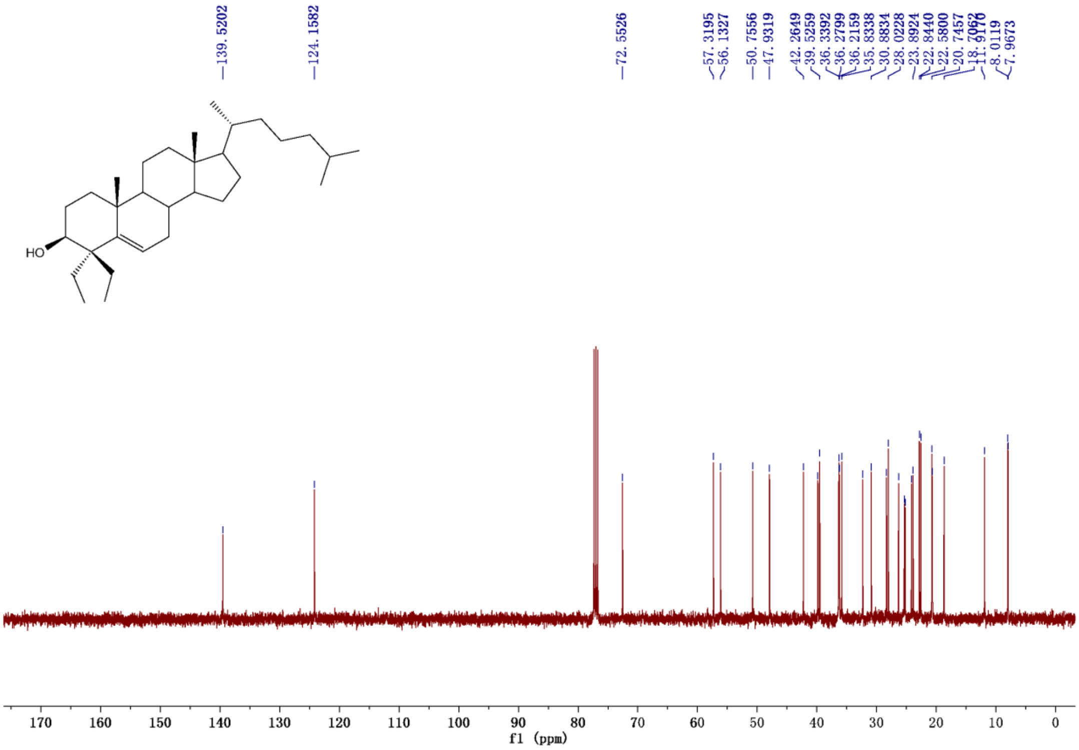

Supplementary Figure 17. Compound 8: ^{13}C-NMR (100 MHz, $CDCl_3$)

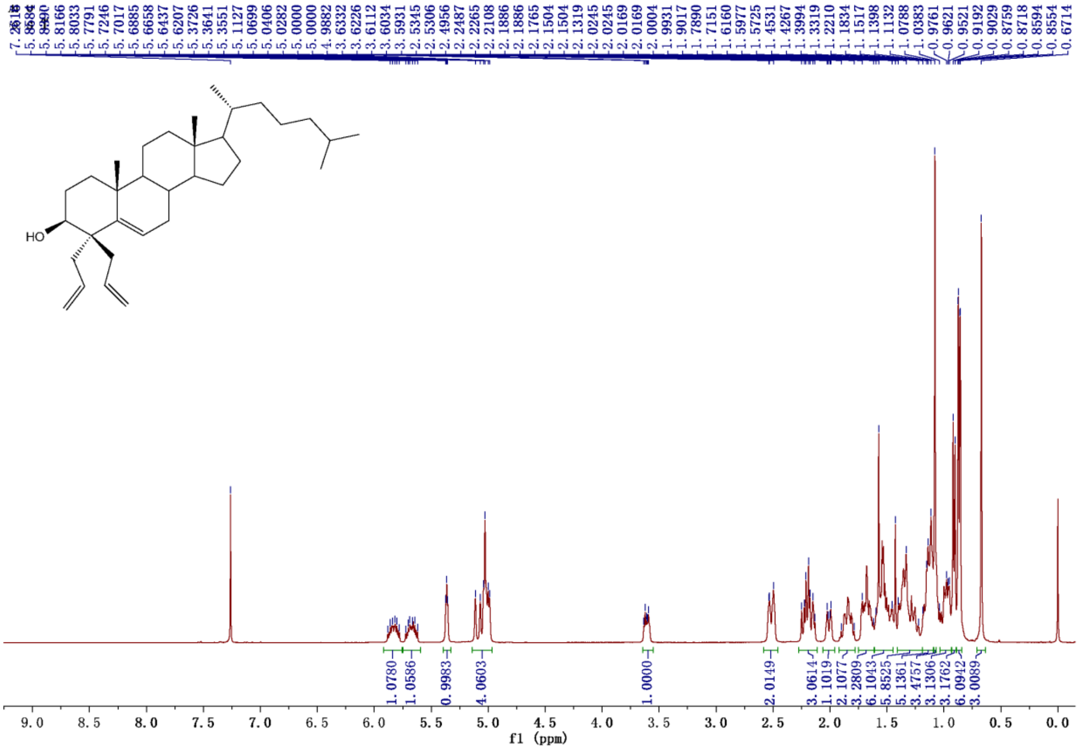

Supplementary Figure 18. Compound 9: ^{1}H-NMR (400 MHz, $CDCl_3$)

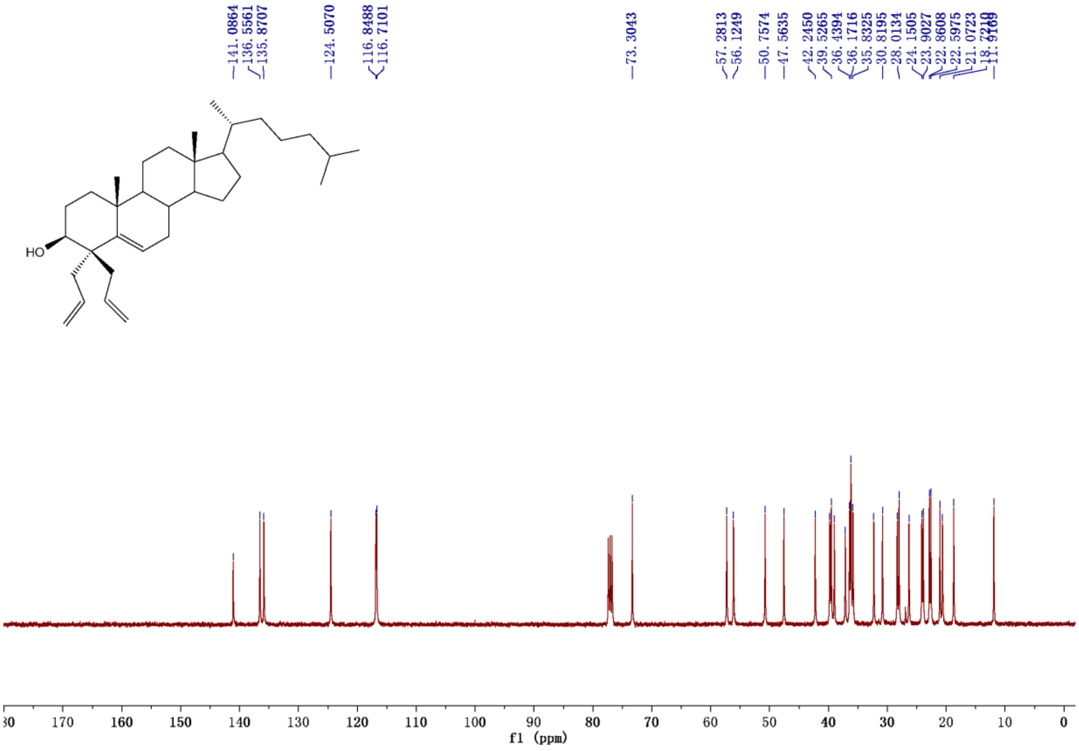

Supplementary Figure 19. Compound 9: ^{13}C-NMR (100 MHz, $CDCl_3$)

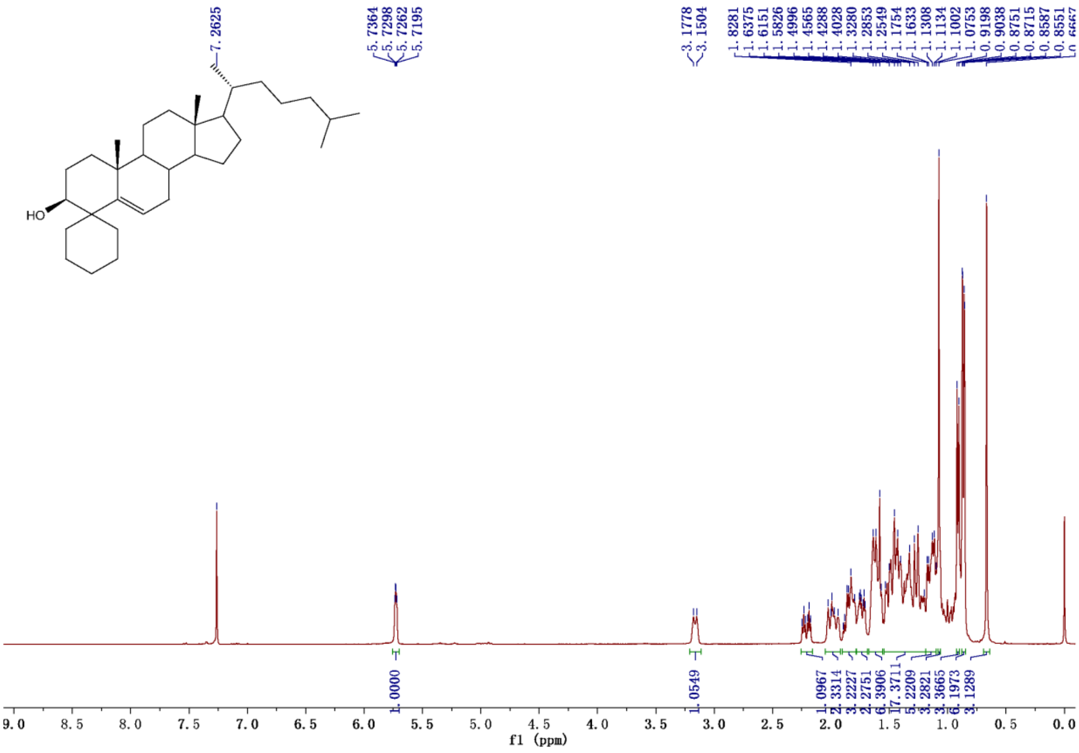

Supplementary Figure 20. Compound 10: ^{1}H-NMR (400 MHz, $CDCl_3$)

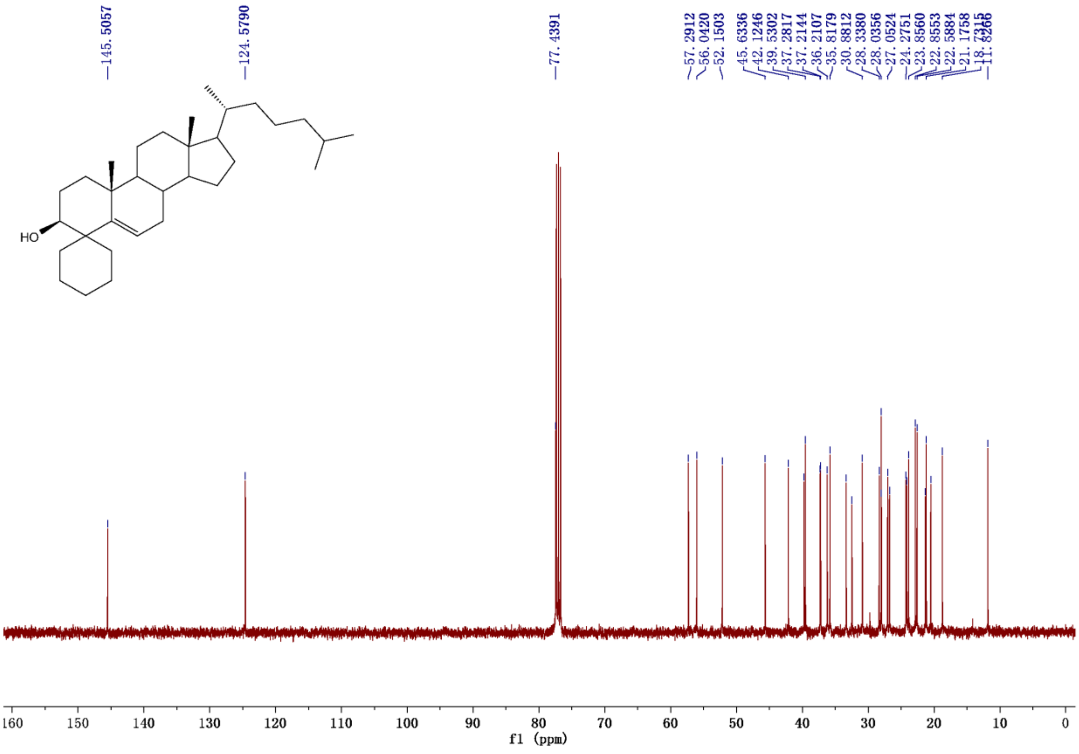

Supplementary Figure 21. Compound 10: ^{13}C-NMR (100 MHz, $CDCl_3$)

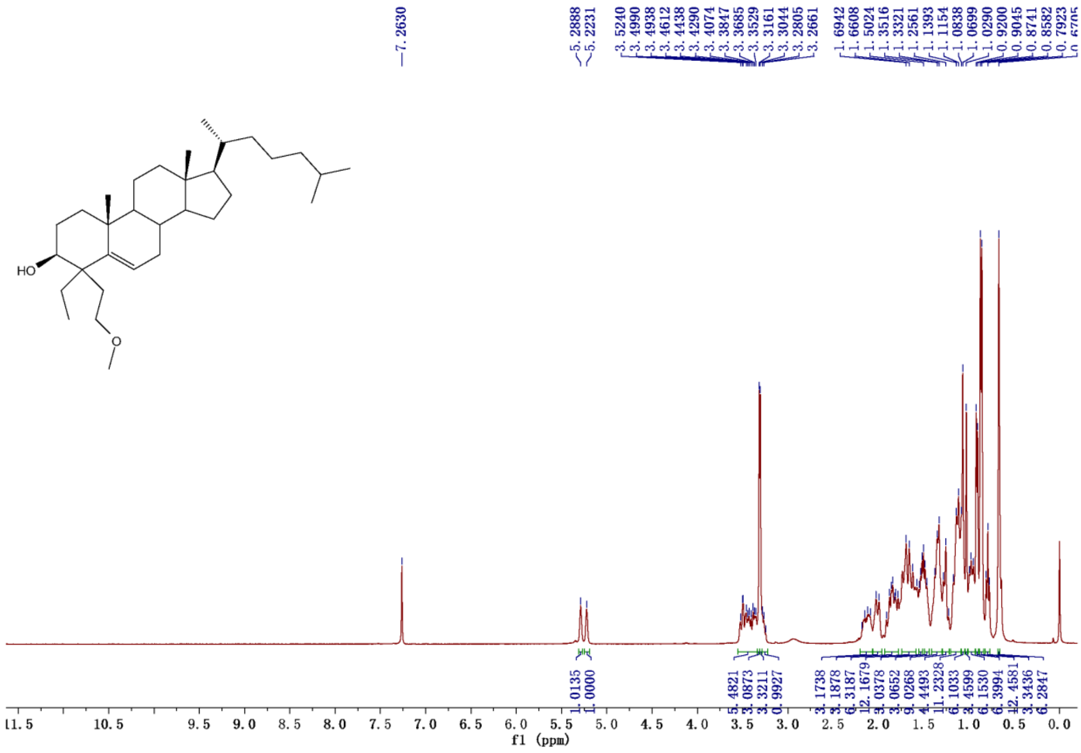

Supplementary Figure 22. Compound 11: ^{1}H-NMR (400 MHz, $CDCl_3$)

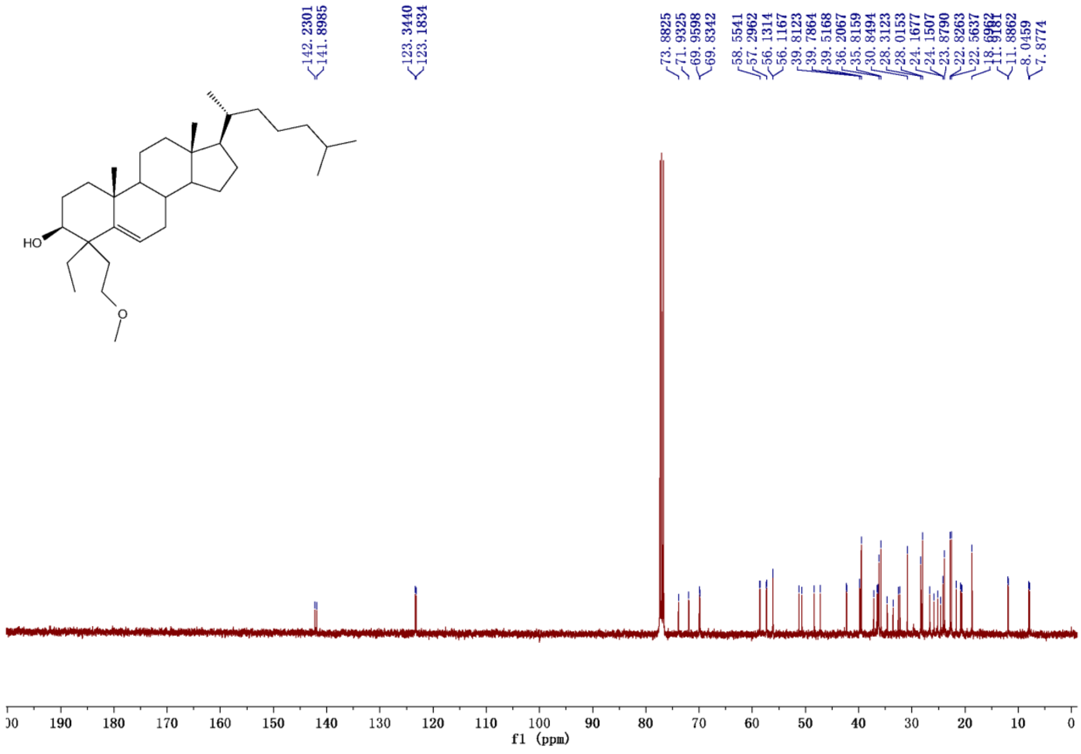

Supplementary Figure 23. Compound 11: ^{13}C-NMR (100 MHz, $CDCl_3$)

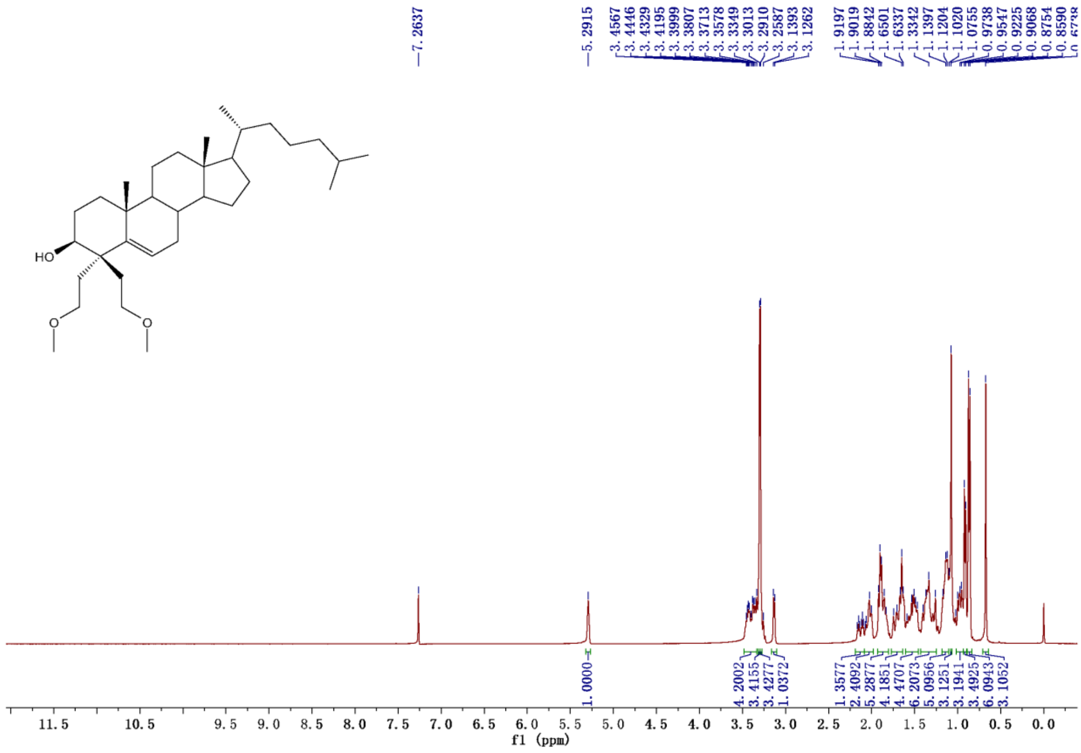

Supplementary Figure 24. Compound 14: ^{1}H-NMR (400 MHz, $CDCl_3$)

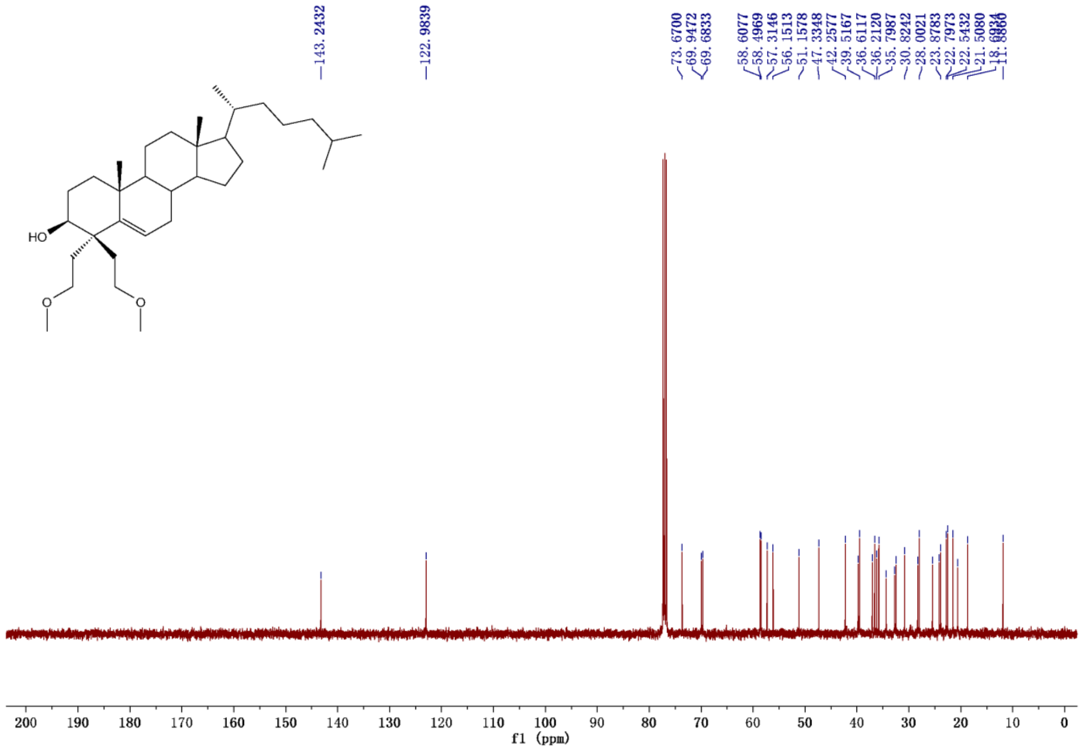

Supplementary Figure 25. Compound 14: ^{13}C-NMR (100 MHz, $CDCl_3$)

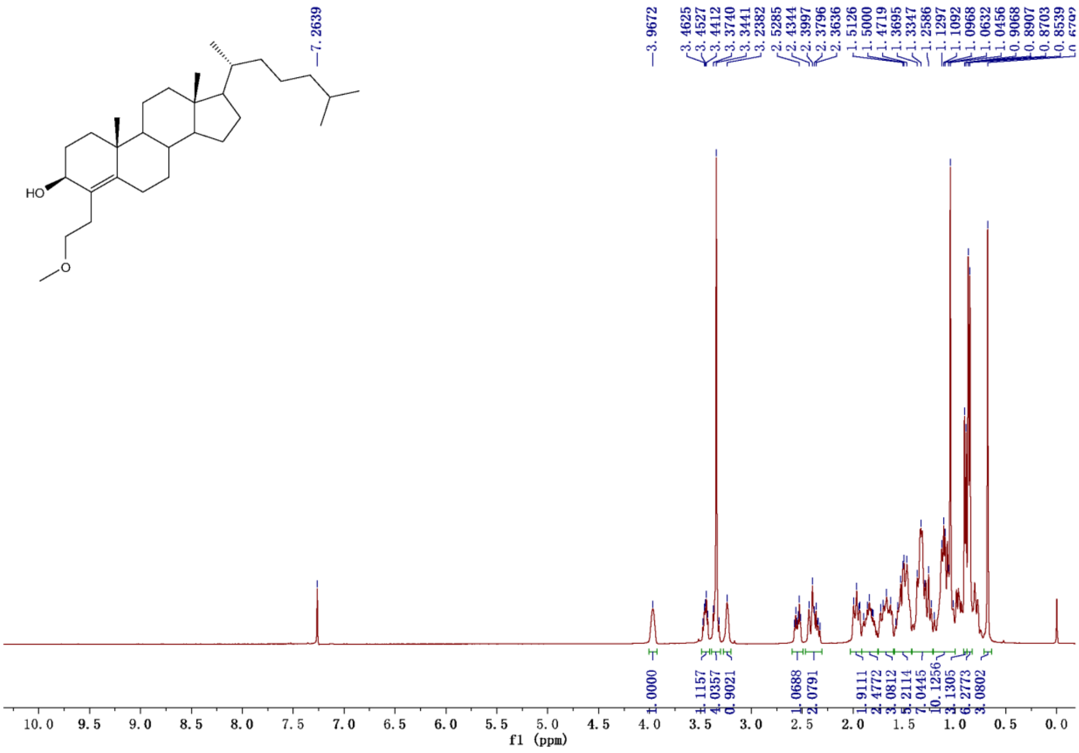

Supplementary Figure 26. Compound 15: ^{1}H-NMR (400 MHz, $CDCl_3$)

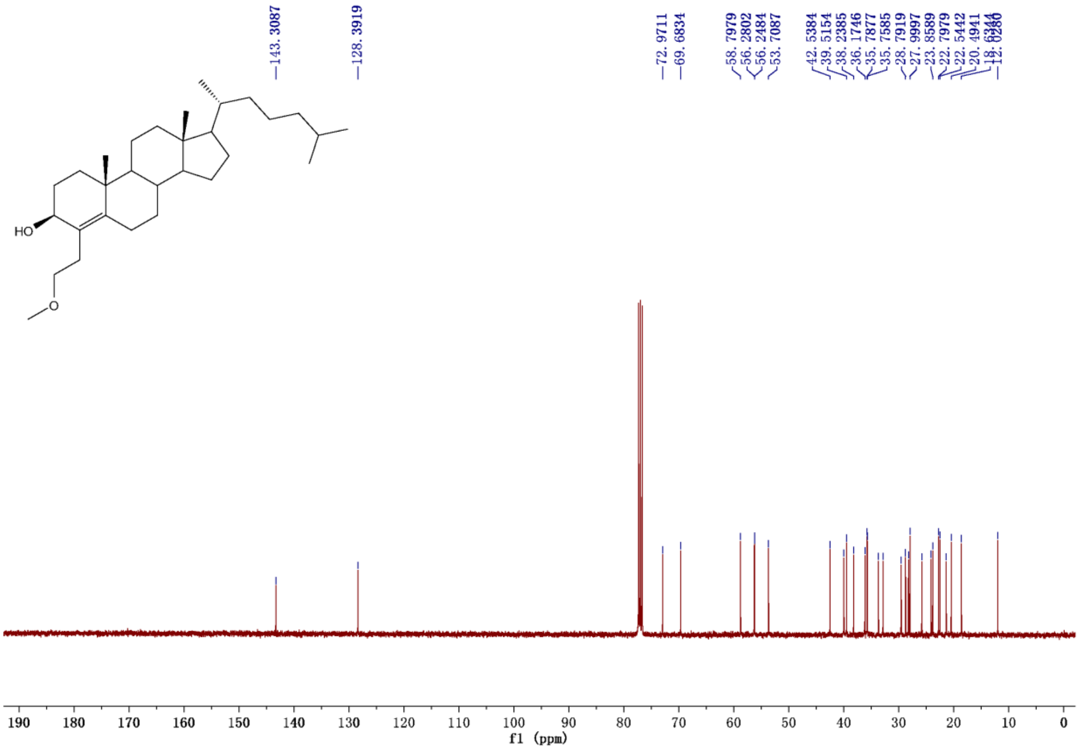

Supplementary Figure 27. Compound 15: ^{13}C-NMR (100 MHz, $CDCl_3$)

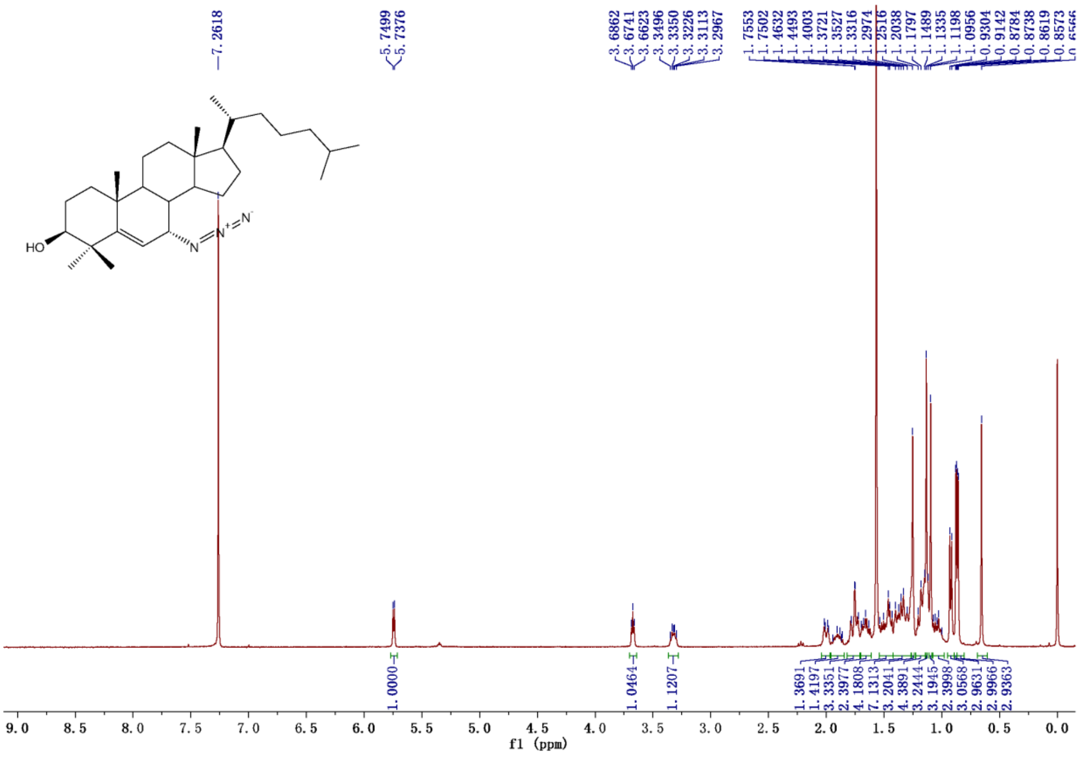

Supplementary Figure 28. Compound 18: ^{1}H-NMR (400 MHz, $CDCl_3$)

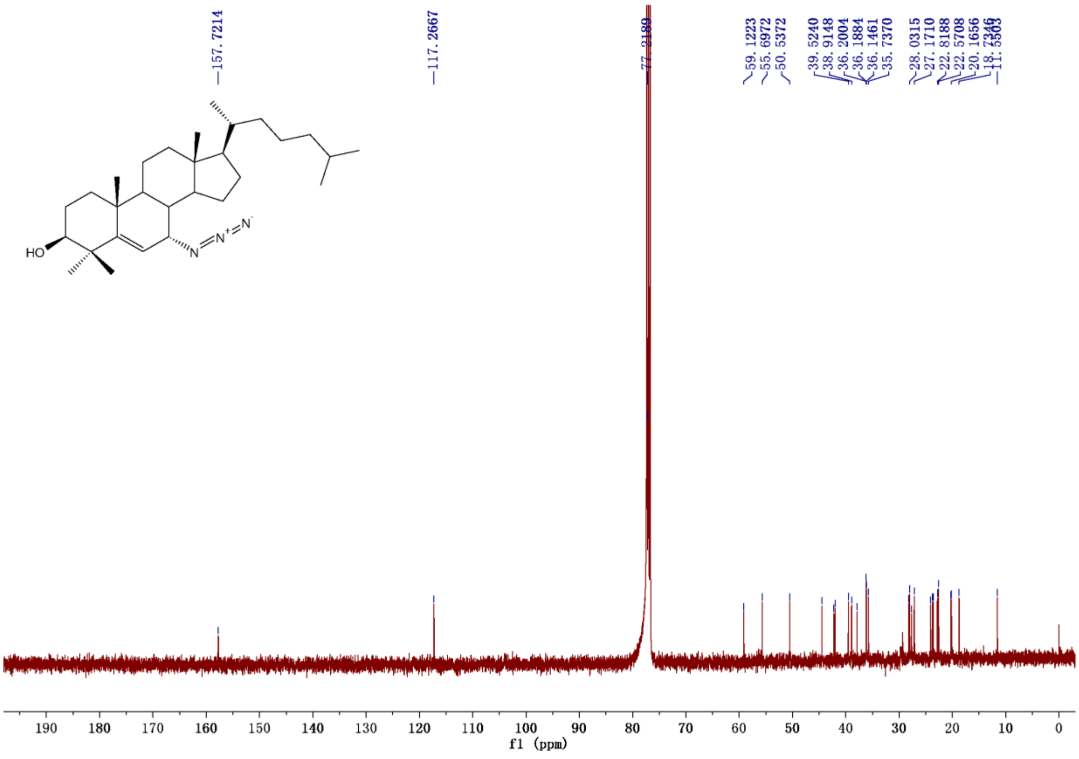

Supplementary Figure 29. Compound 18: ^{13}C-NMR (100 MHz, $CDCl_3$)

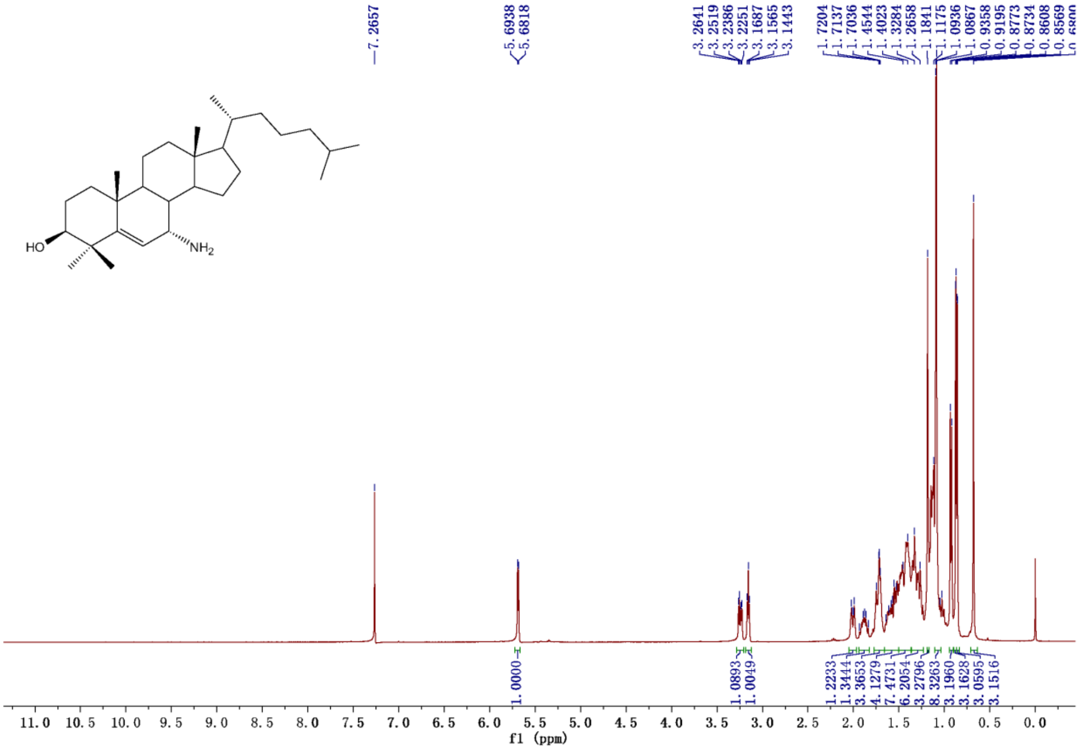

Supplementary Figure 30. Compound 19: ^{1}H-NMR (400 MHz, $CDCl_3$)

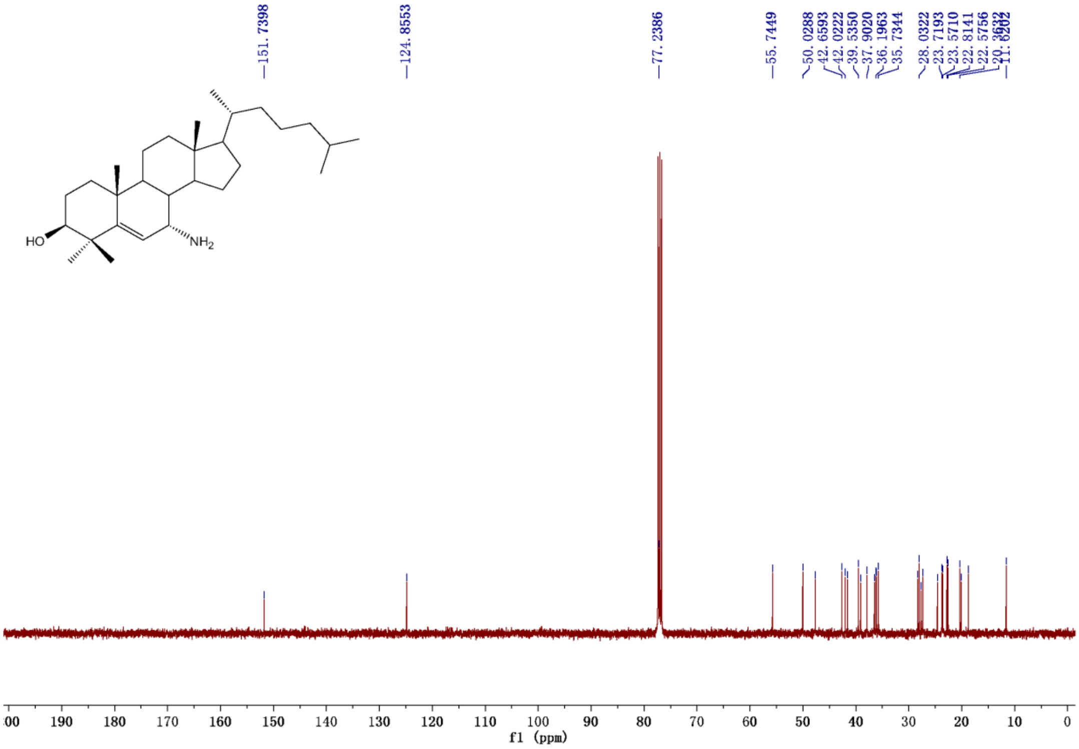

Supplementary Figure 31. Compound 19: ^{13}C-NMR (100 MHz, $CDCl_3$)

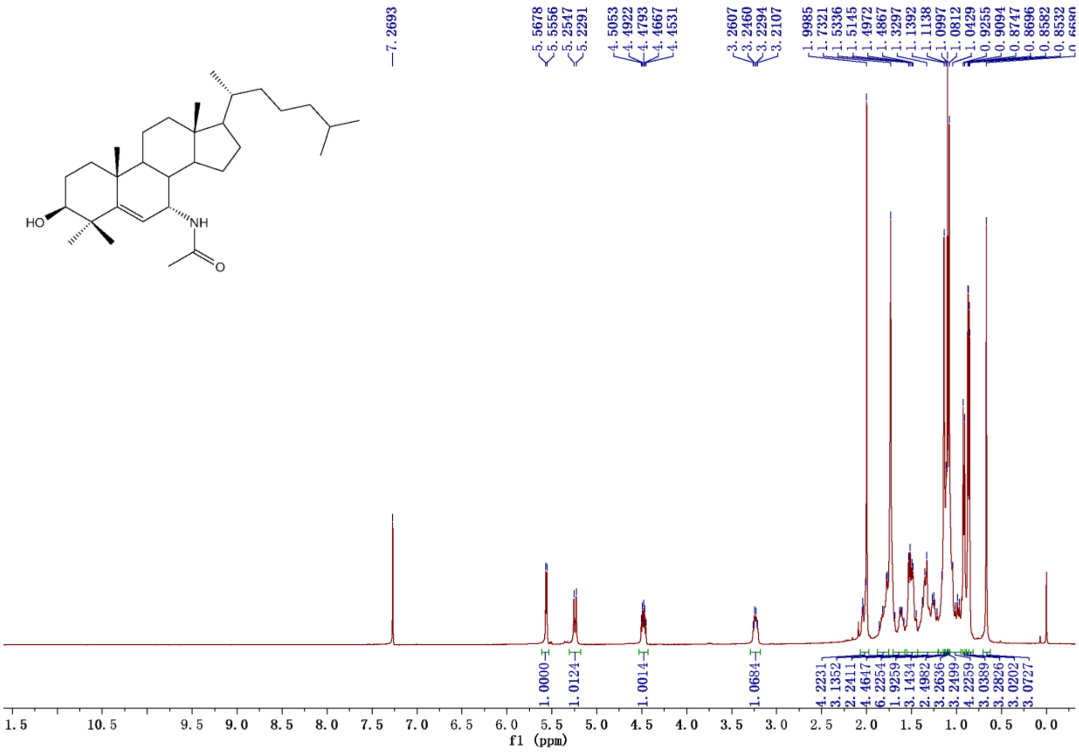

Supplementary Figure 32. Compound 22: ^{1}H-NMR (400 MHz, $CDCl_3$)

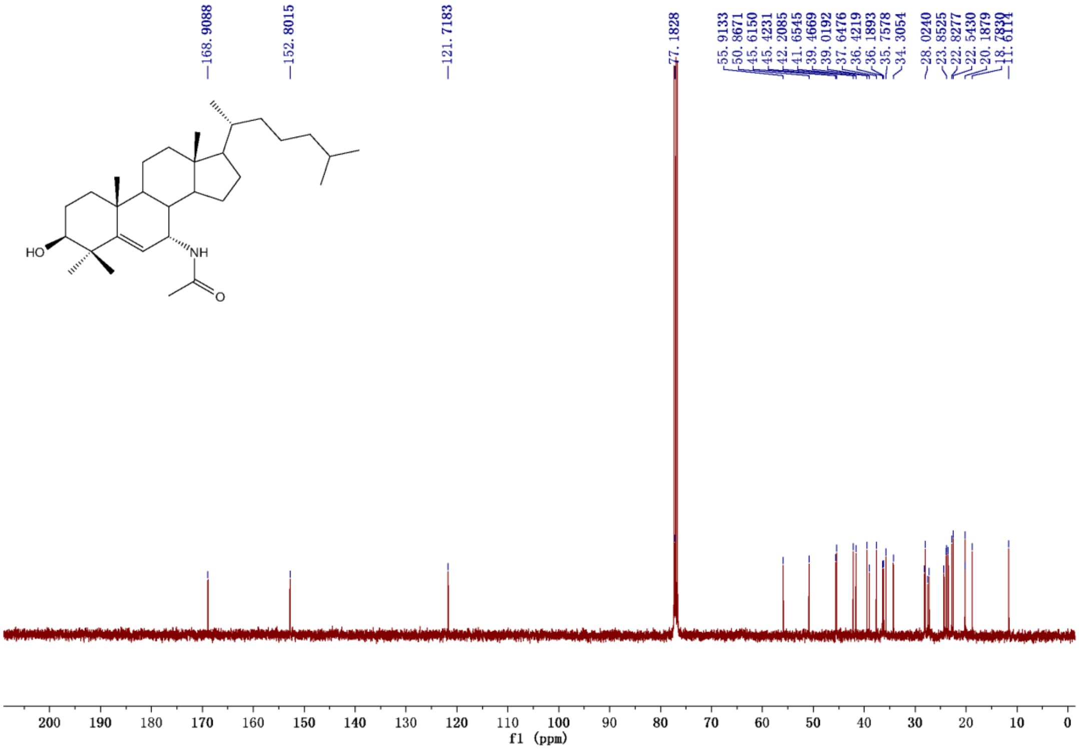

Supplementary Figure 33. Compound 22: ^{13}C-NMR (100 MHz, $CDCl_3$)

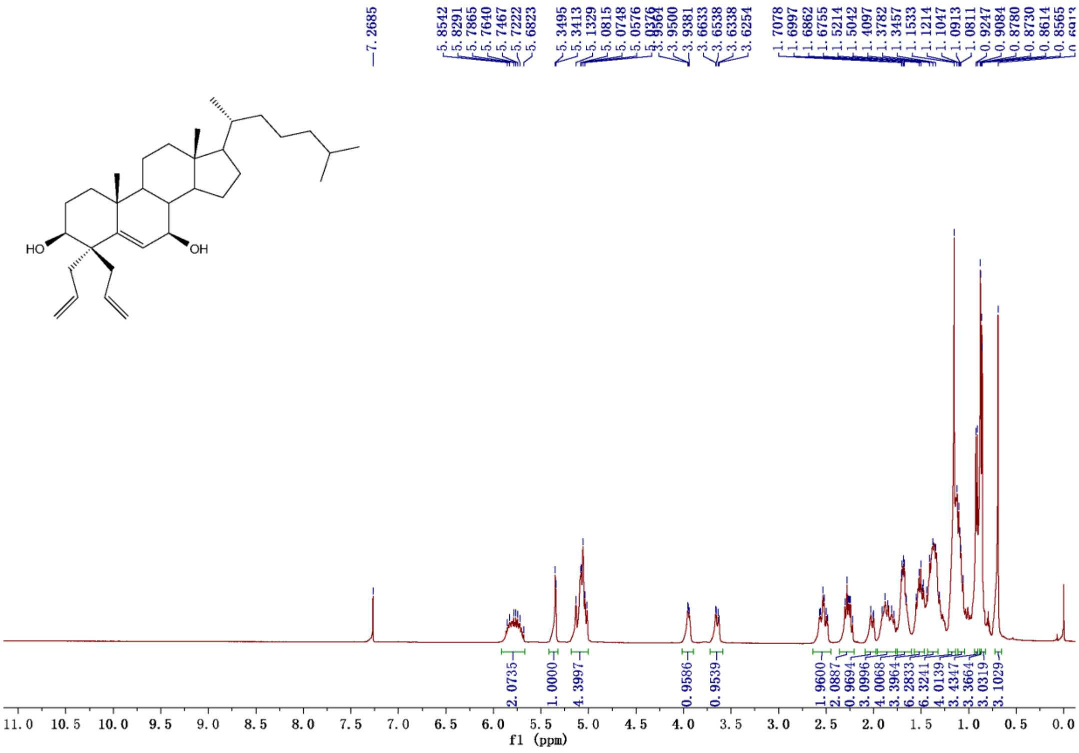

Supplementary Figure 34. Compound 26: ^{1}H-NMR (400 MHz, $CDCl_3$)

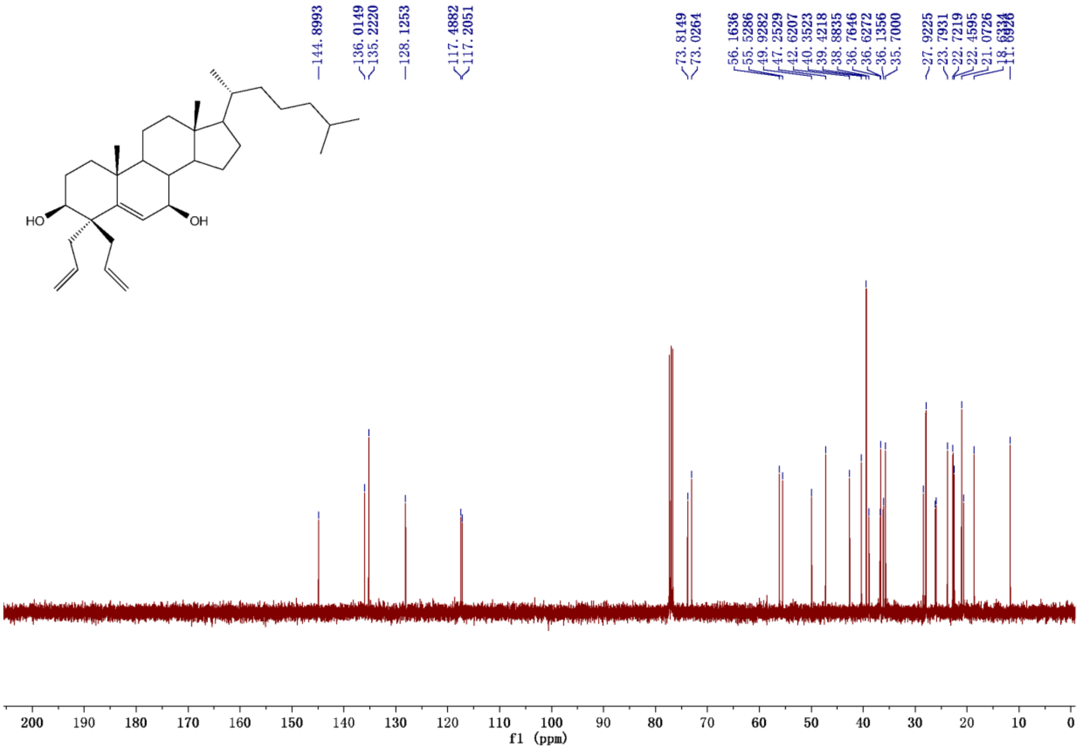

Supplementary Figure 35. Compound 26: ^{13}C-NMR (100 MHz, $CDCl_3$)

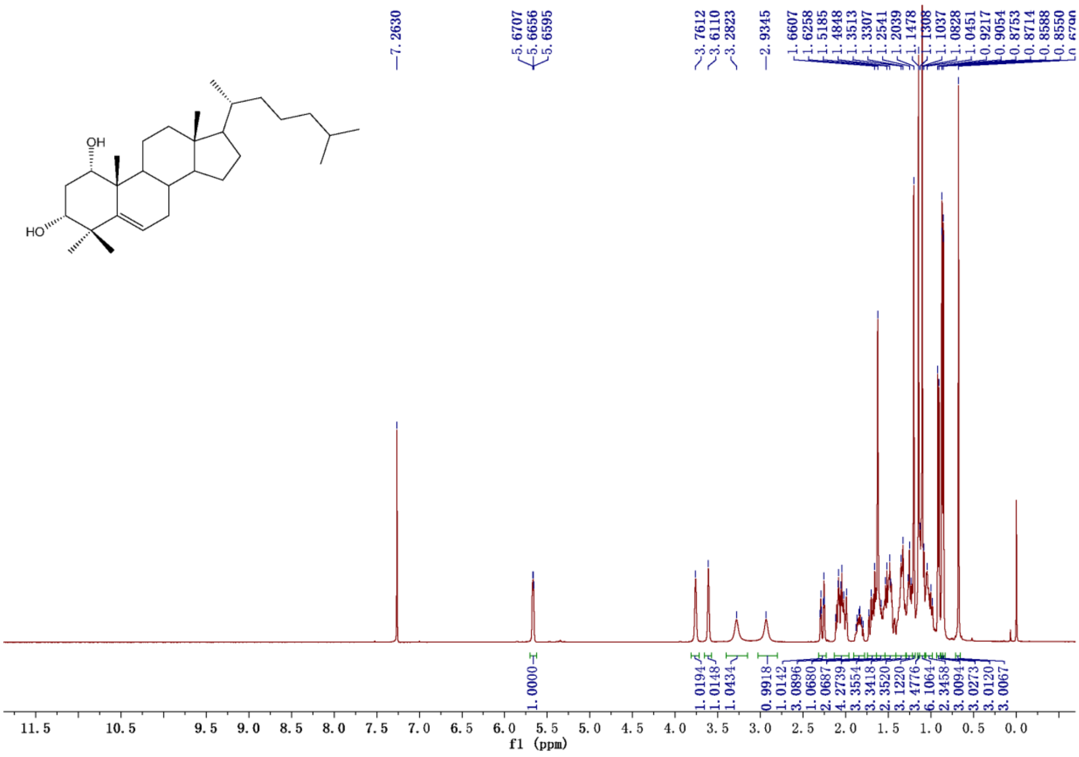

Supplementary Figure 36. Compound 30: ^{1}H-NMR (400 MHz, $CDCl_3$)

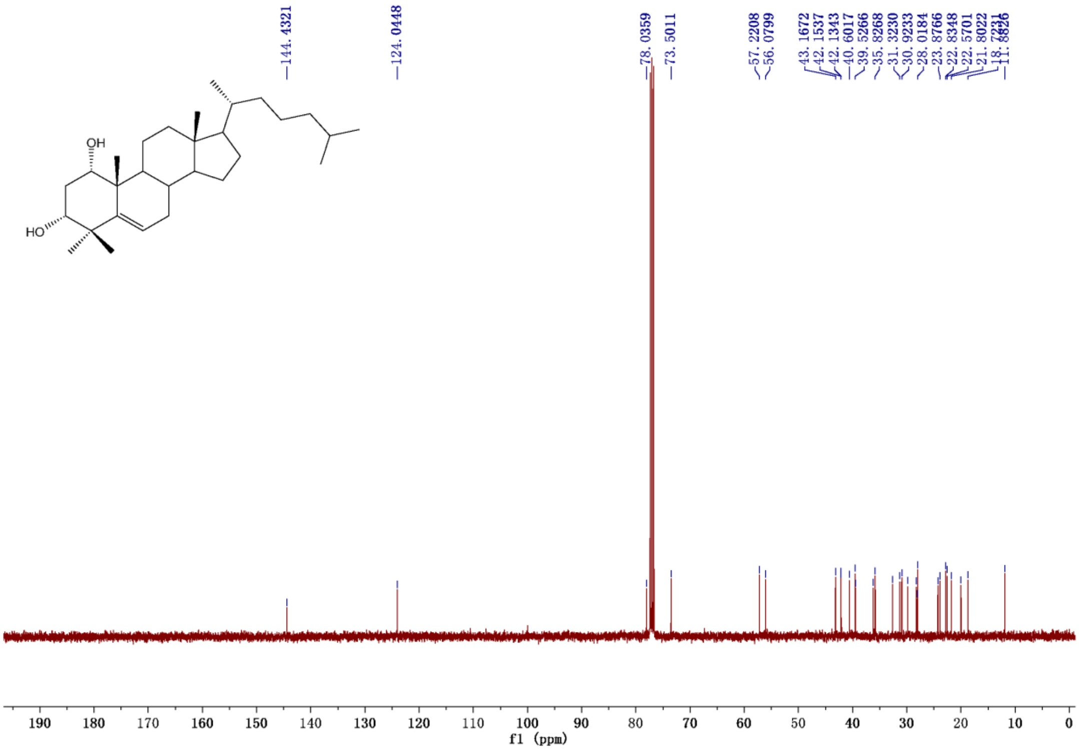

Supplementary Figure 37. Compound 30: ^{13}C-NMR (100 MHz, $CDCl_3$)

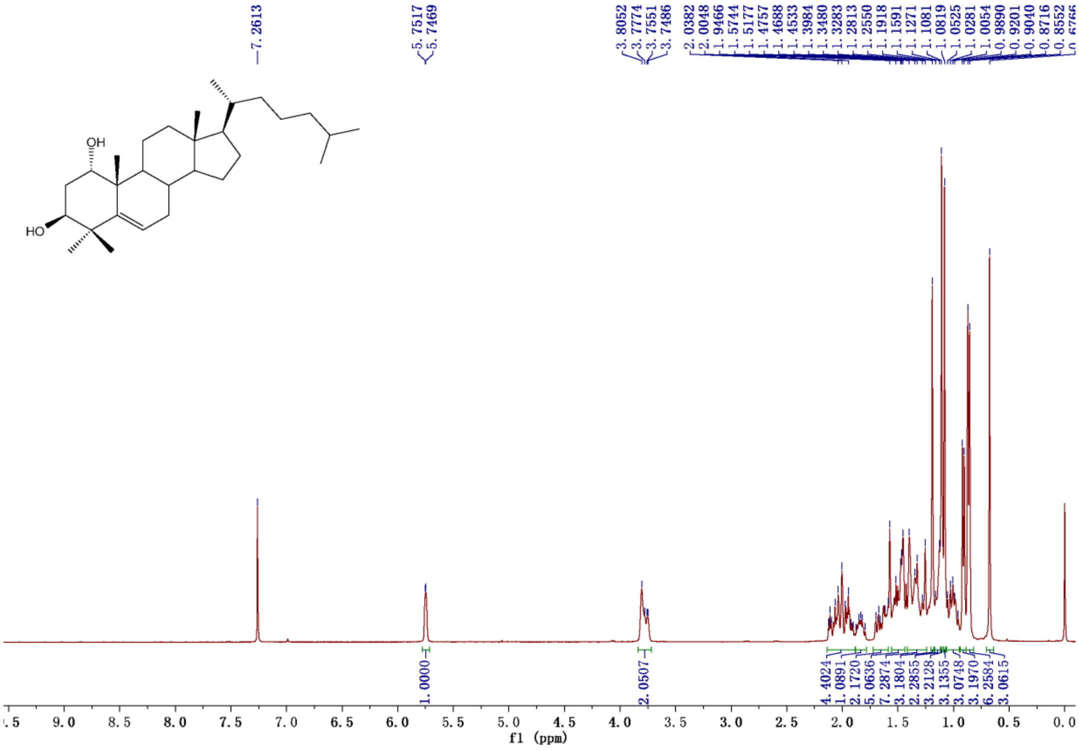

Supplementary Figure 38. Compound 31: ^{1}H-NMR (400 MHz, CDCl$_3$)

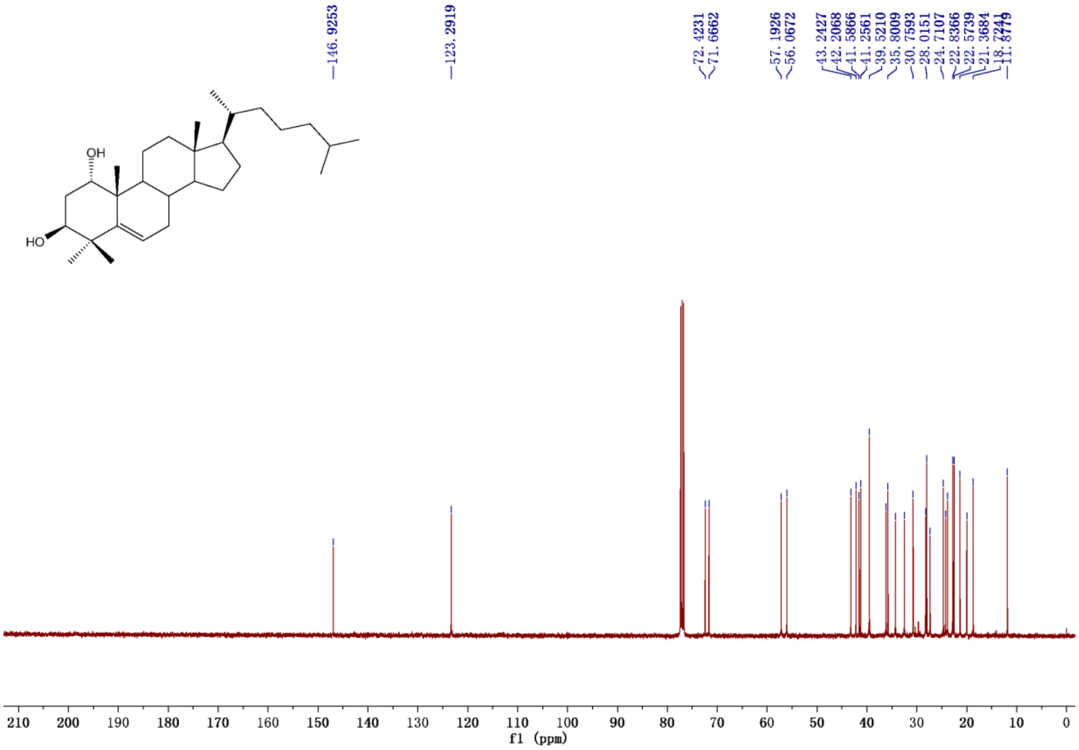

Supplementary Figure 39. Compound 31: ^{13}C-NMR (100 MHz, CDCl$_3$)

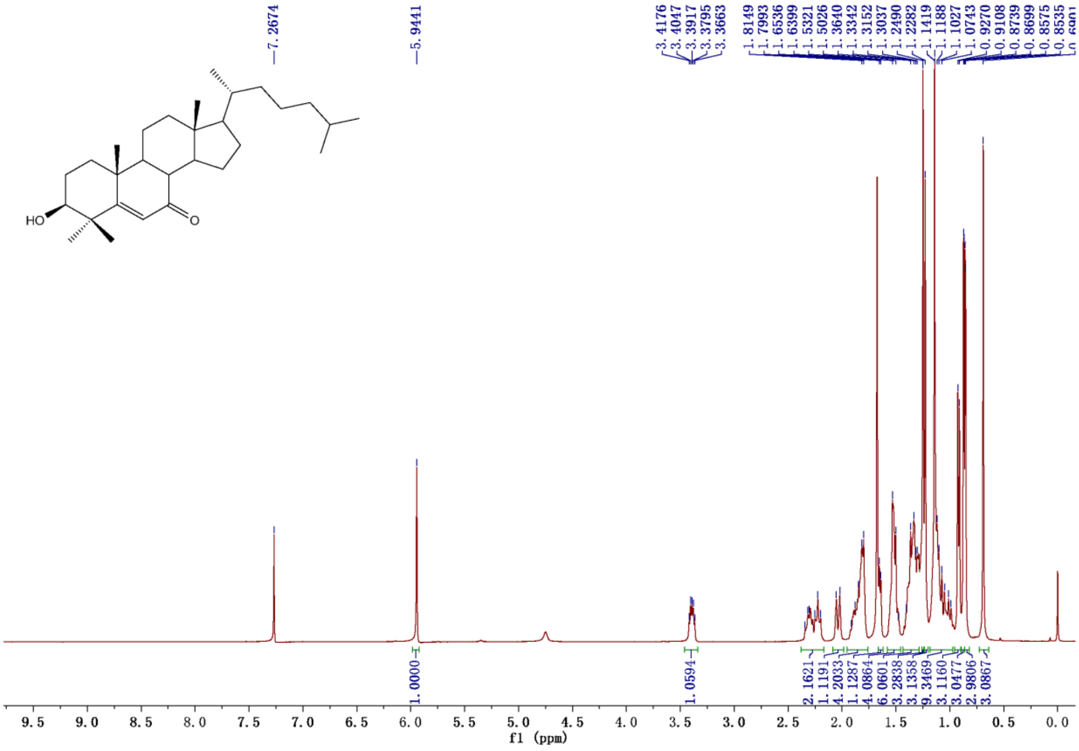

Supplementary Figure 40. Compound 33: ^{1}H-NMR (400 MHz, $CDCl_3$)

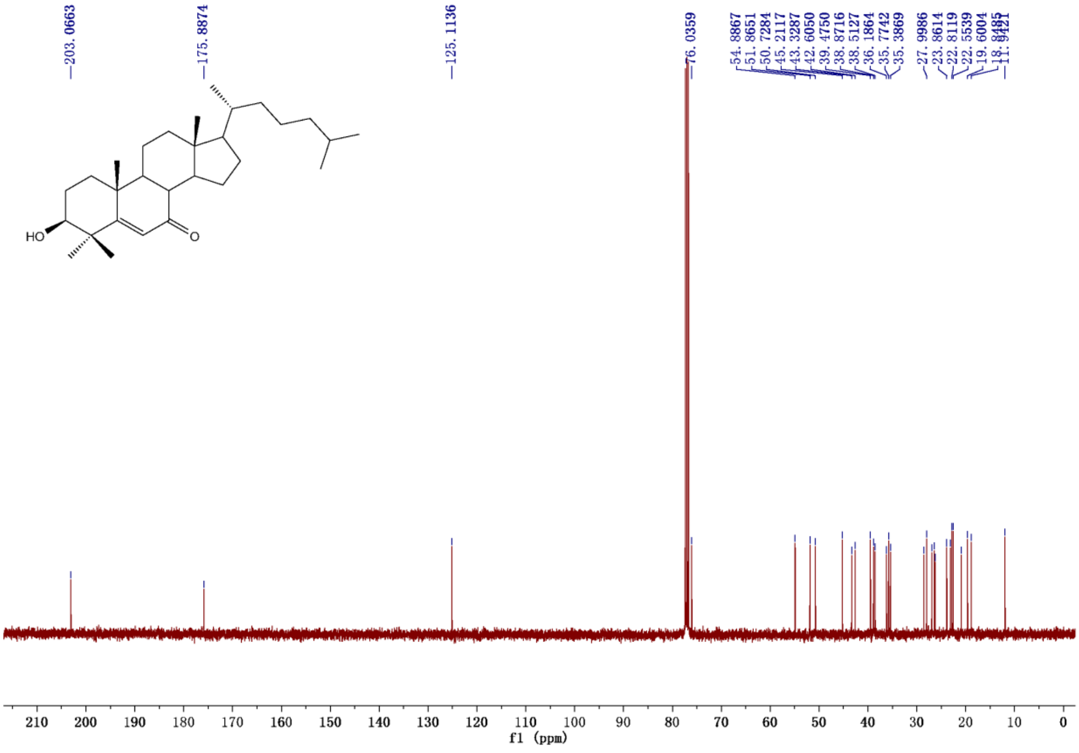

Supplementary Figure 41. Compound 33: ^{13}C-NMR (100 MHz, $CDCl_3$)

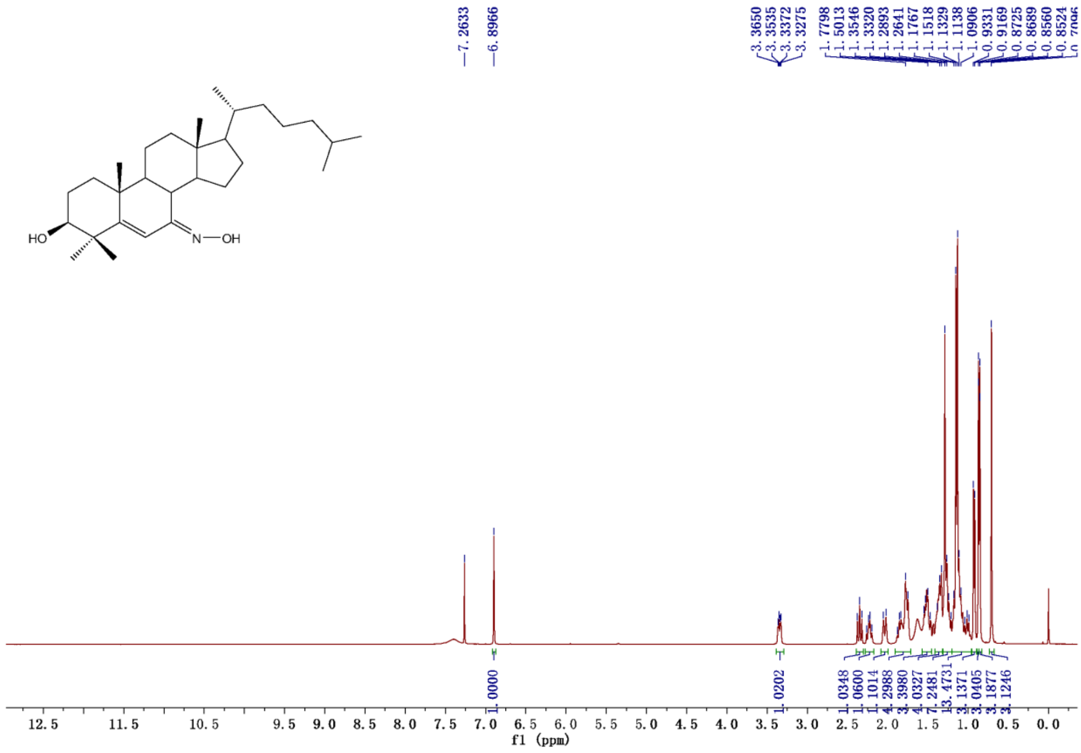

Supplementary Figure 42. Compound 34: ^{1}H-NMR (400 MHz, $CDCl_3$)

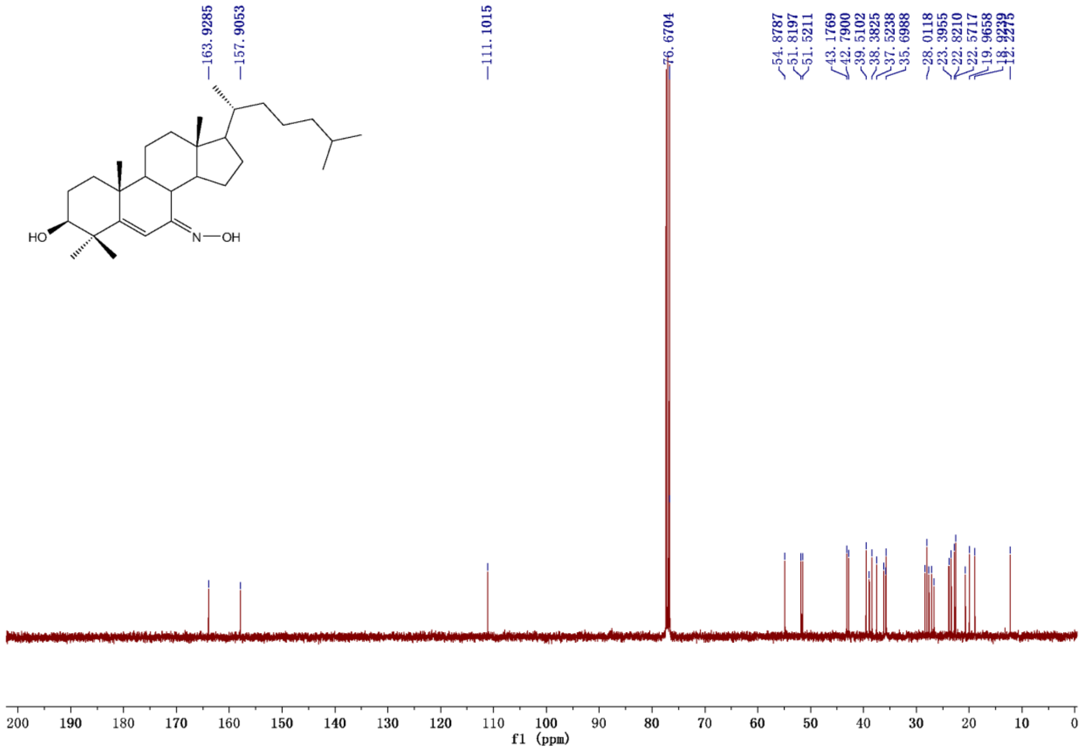

Supplementary Figure 43. Compound 34: ^{13}C-NMR (100 MHz, $CDCl_3$)

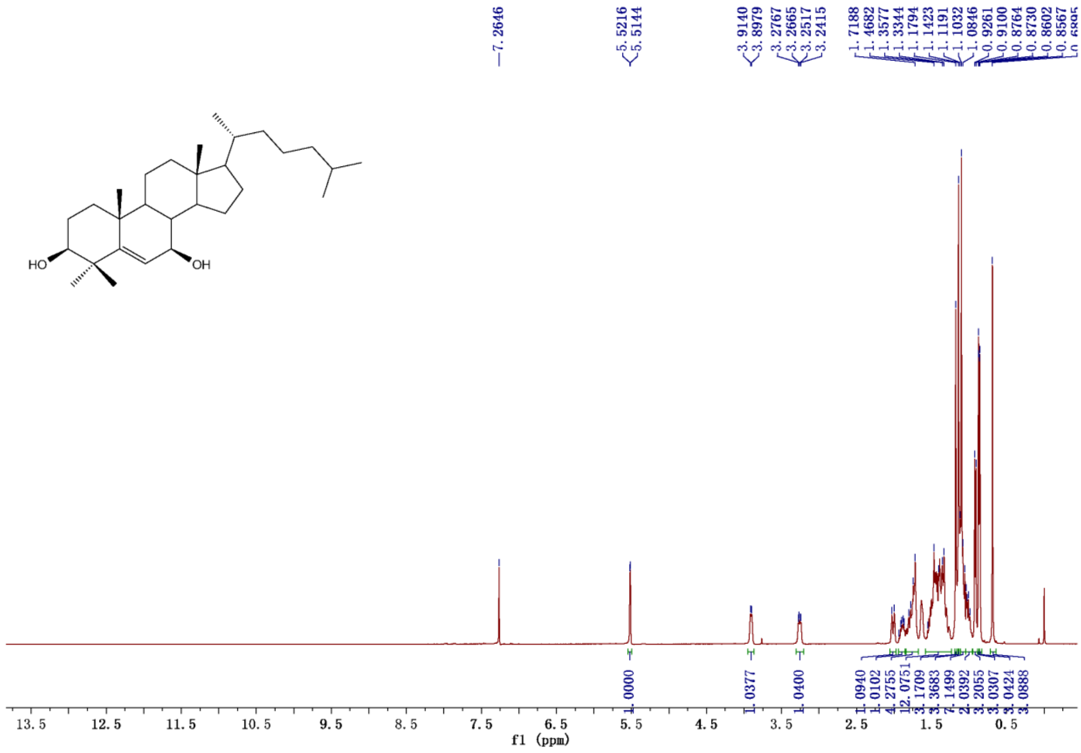

Supplementary Figure 44. Compound 35: ^{1}H-NMR (400 MHz, $CDCl_3$)

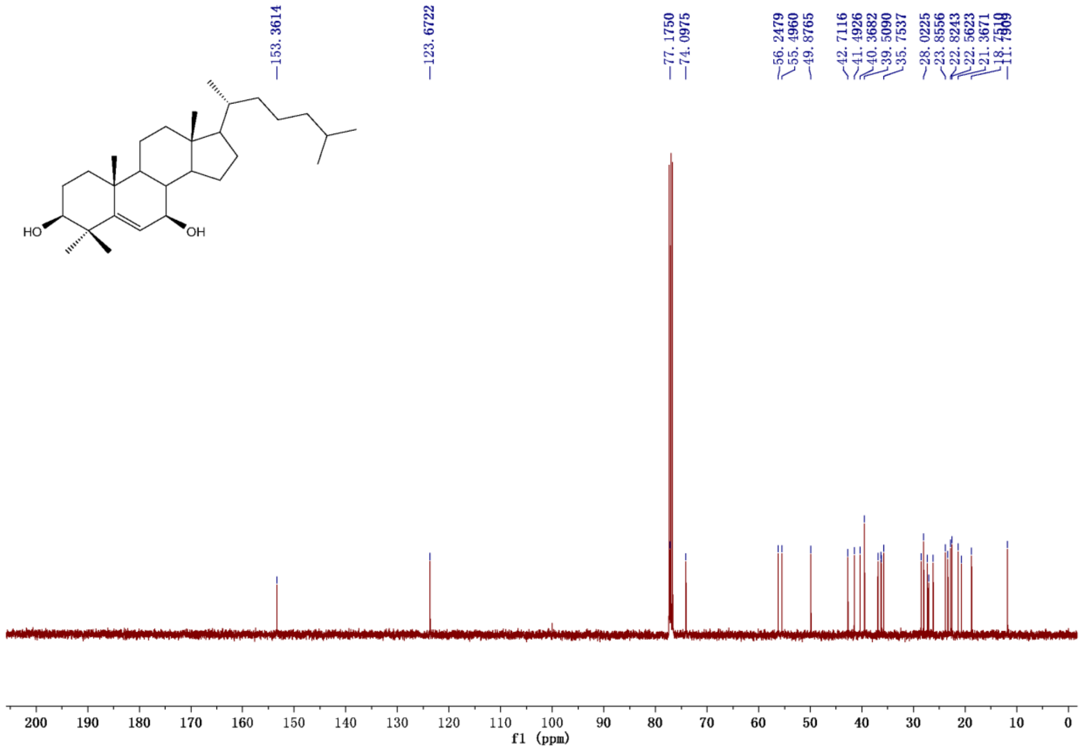

Supplementary Figure 45. Compound 35: ^{13}C-NMR (100 MHz, $CDCl_3$)

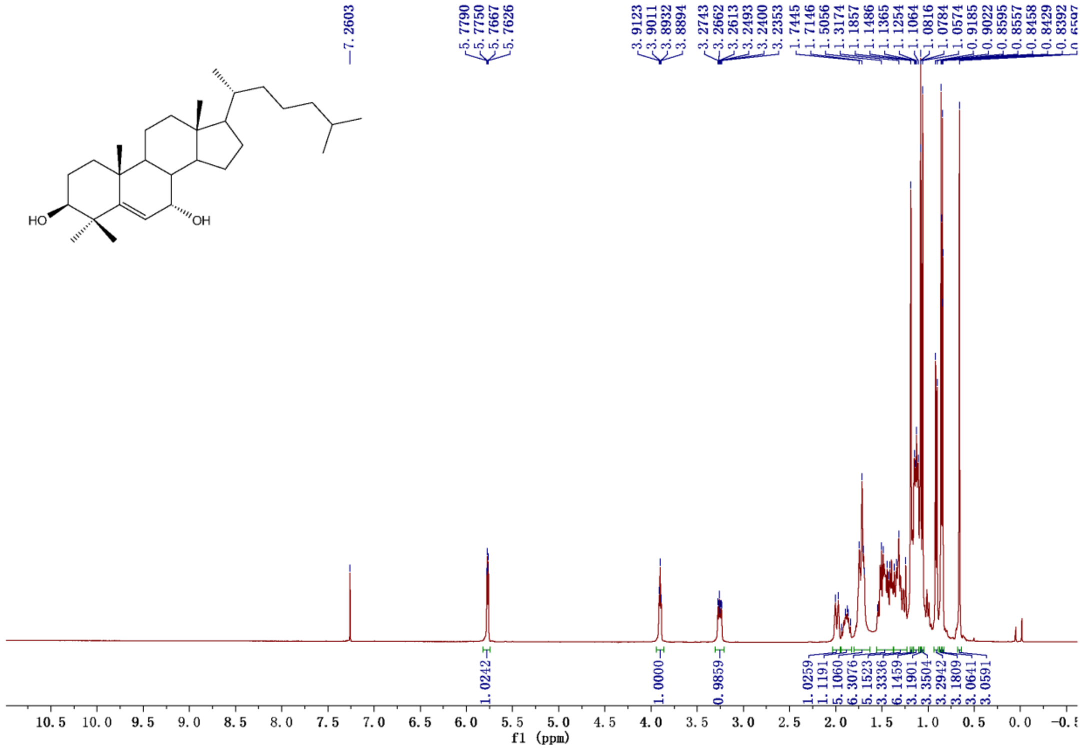

Supplementary Figure 46. Compound 36: ^{1}H-NMR (400 MHz, $CDCl_3$)

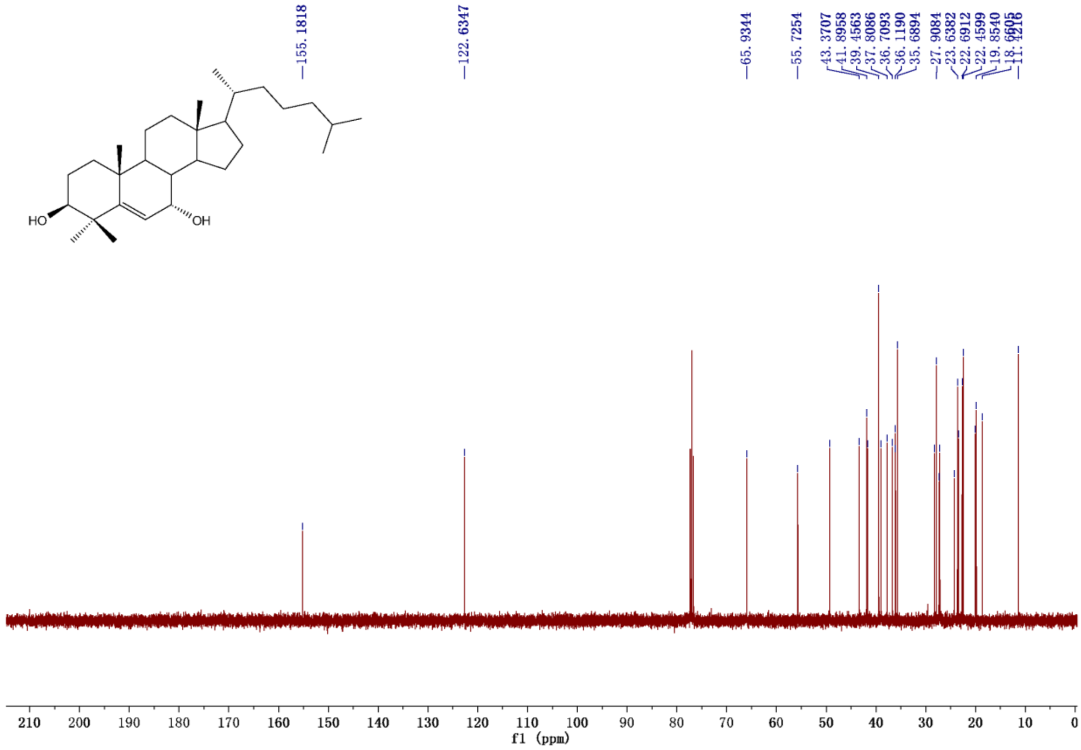

Supplementary Figure 47. Compound 36: ^{13}C-NMR (100 MHz, $CDCl_3$)

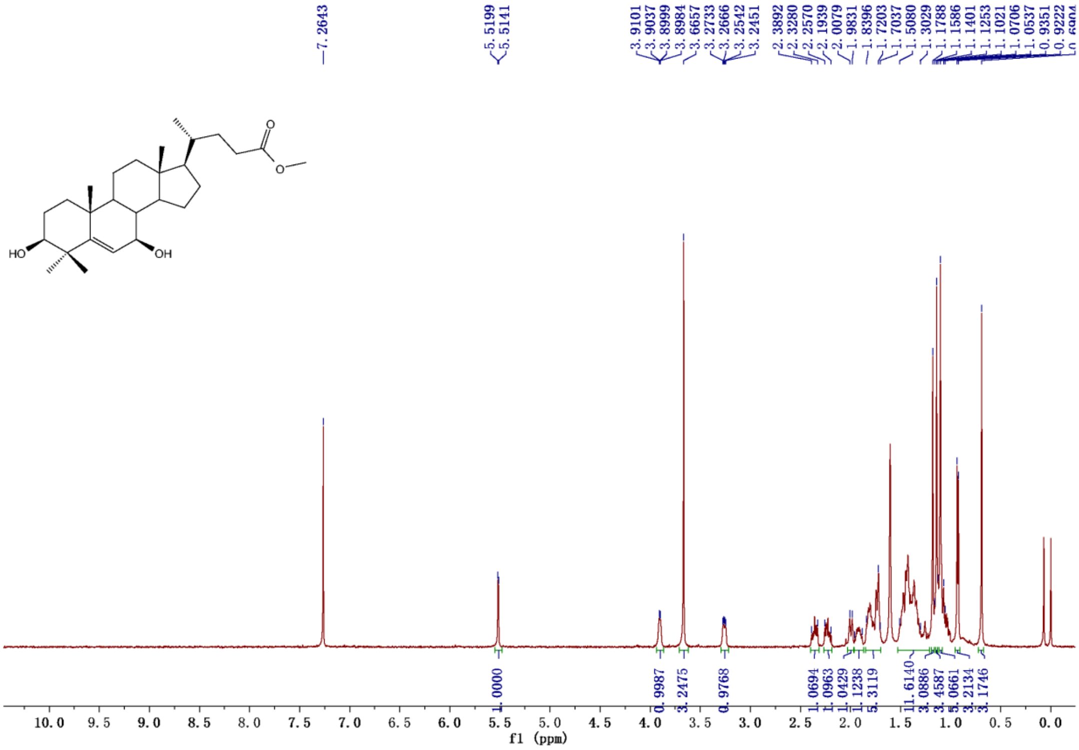

Supplementary Figure 48. Compound 49: ^{1}H-NMR (500 MHz, $CDCl_3$)

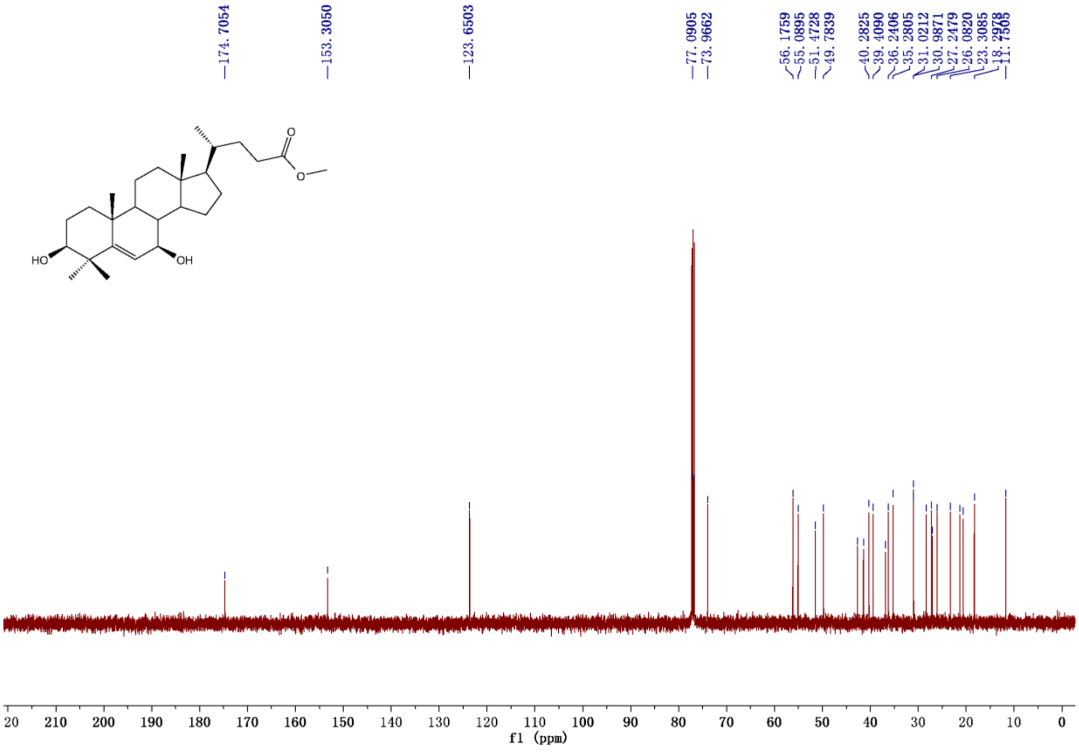

Supplementary Figure 49. Compound 49: ^{13}C-NMR (125 MHz, $CDCl_3$)

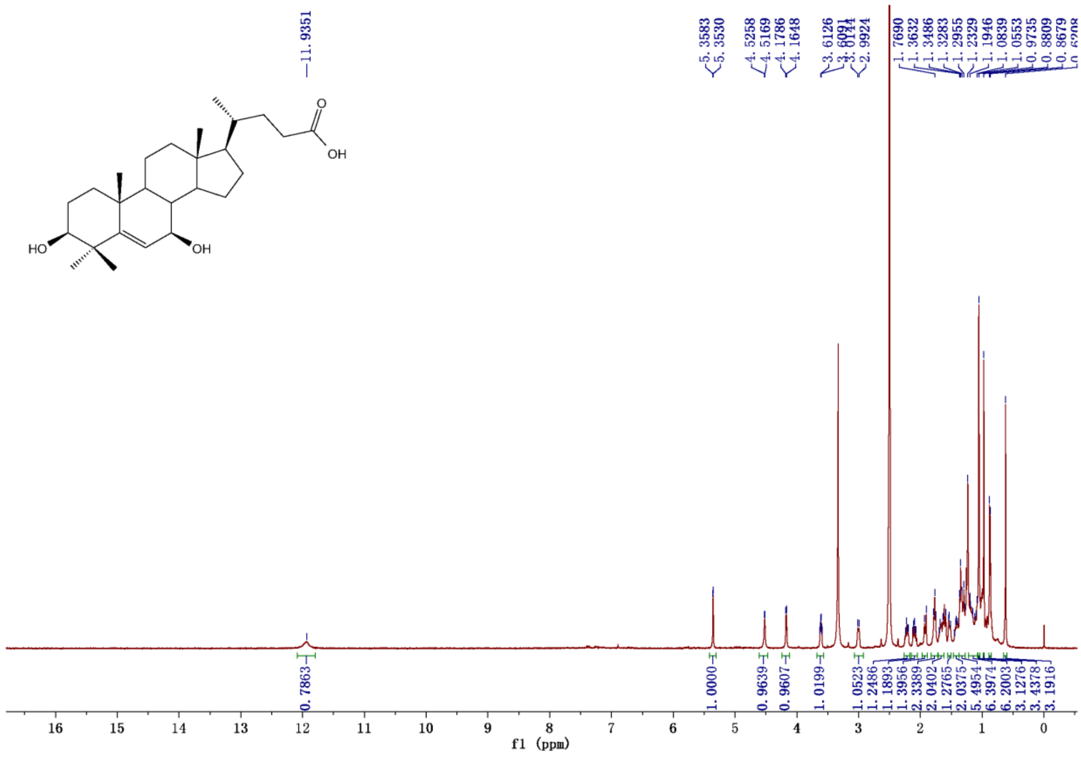

Supplementary Figure 50. Compound 50: ^{1}H-NMR (500 MHz, DMSO-d_6)

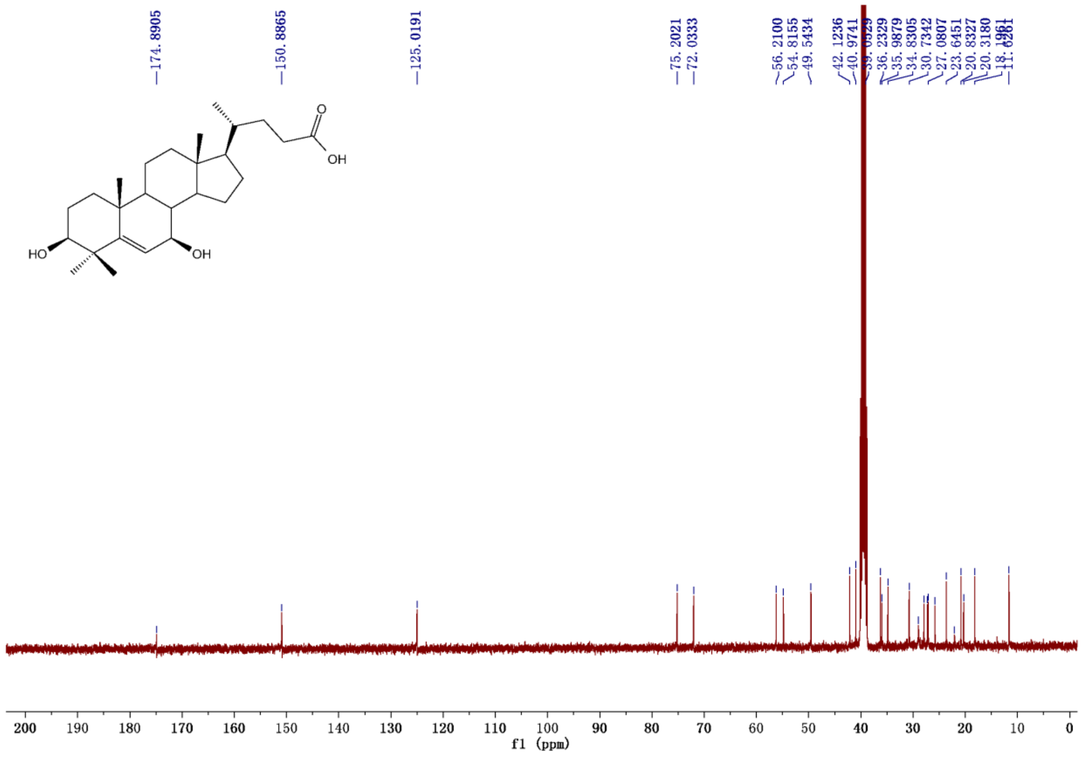

Supplementary Figure 51. Compound 50: ^{13}C-NMR (100 MHz, DMSO-d_6)

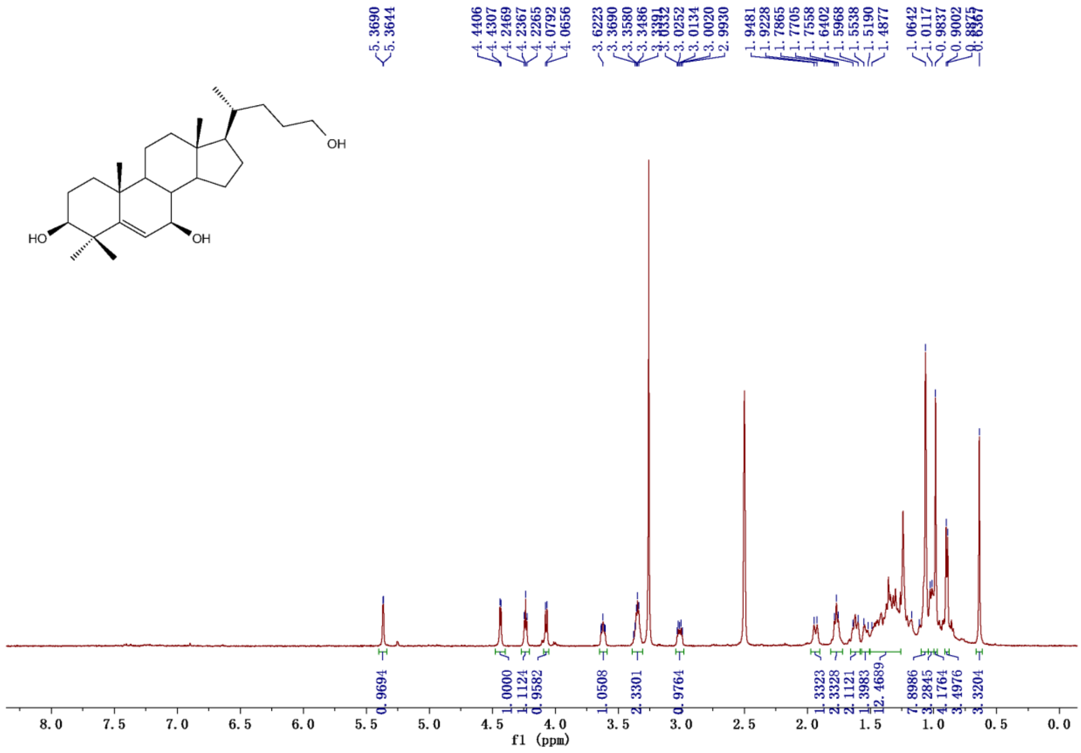

Supplementary Figure 52. Compound 51: ^{1}H-NMR (500 MHz, DMSO-d_6)

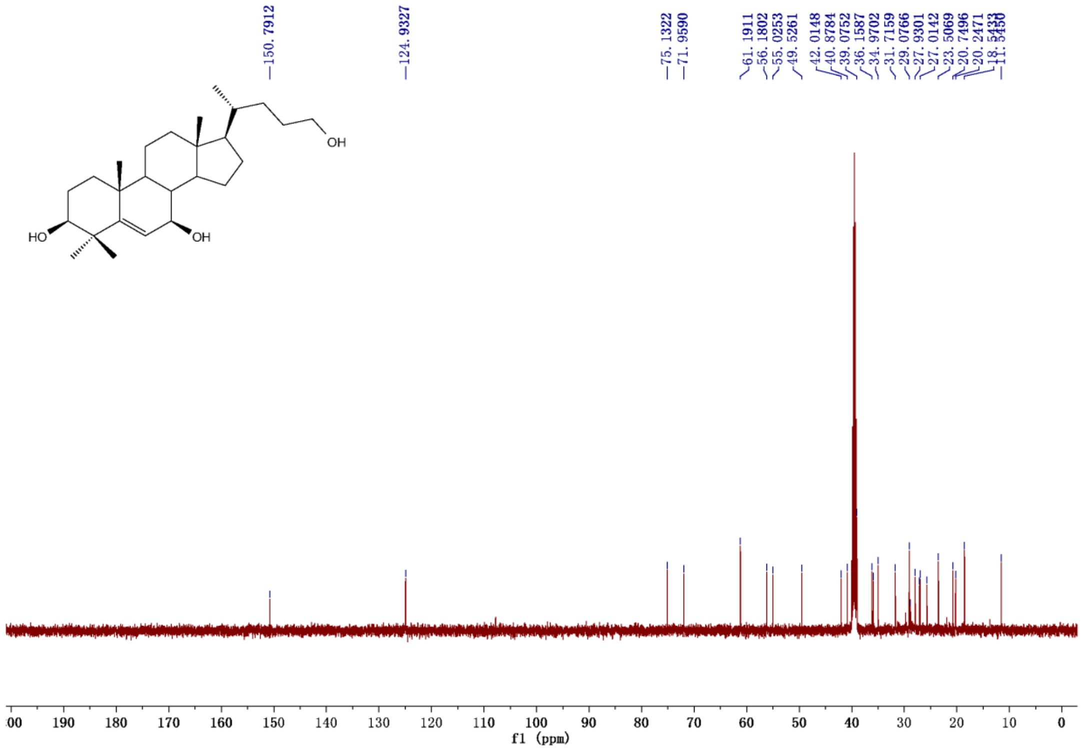

Supplementary Figure 53. Compound 51: ^{13}C-NMR (125 MHz, DMSO-d_6)

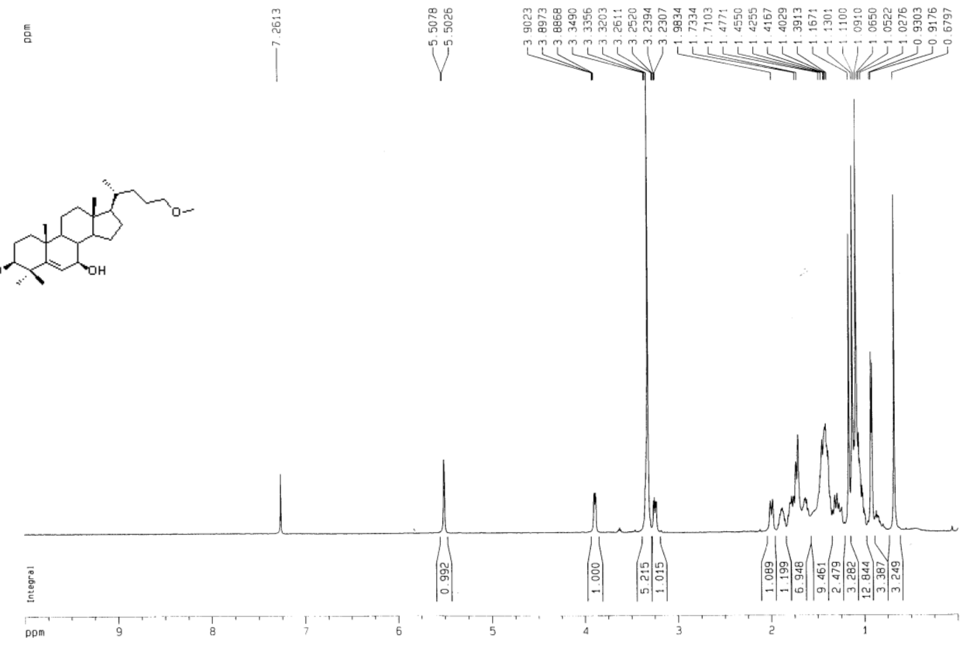

Supplementary Figure 54. Compound 55: ^{1}H-NMR (500 MHz, $CDCl_3$)

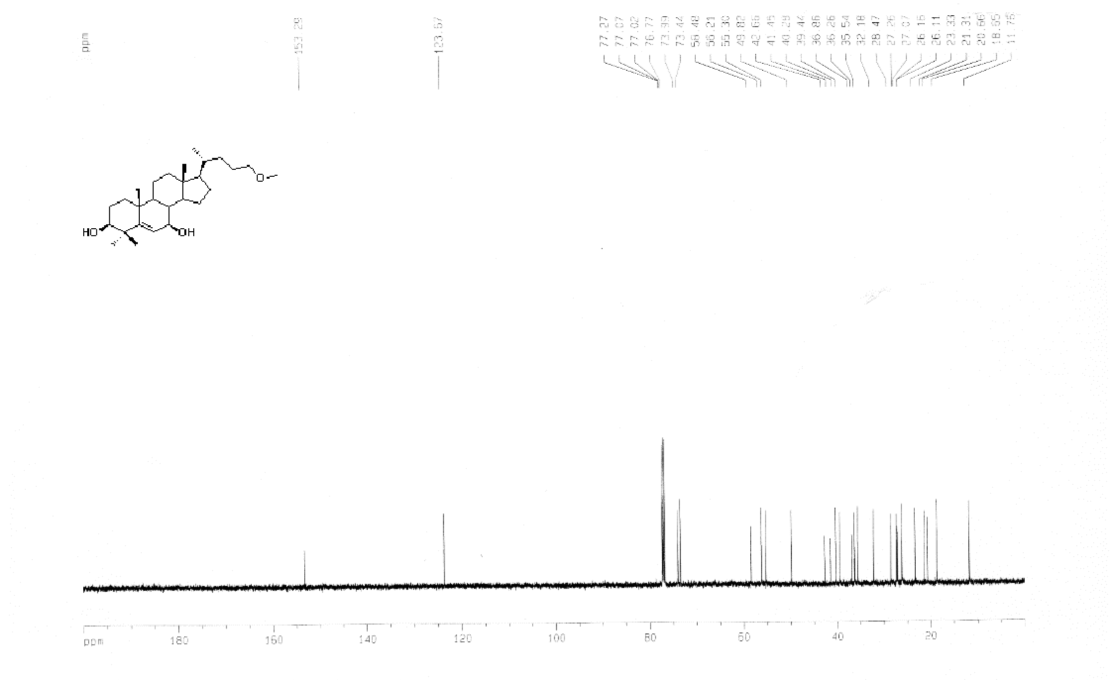

Supplementary Figure 55. Compound 55: ^{13}C-NMR (125 MHz, $CDCl_3$)

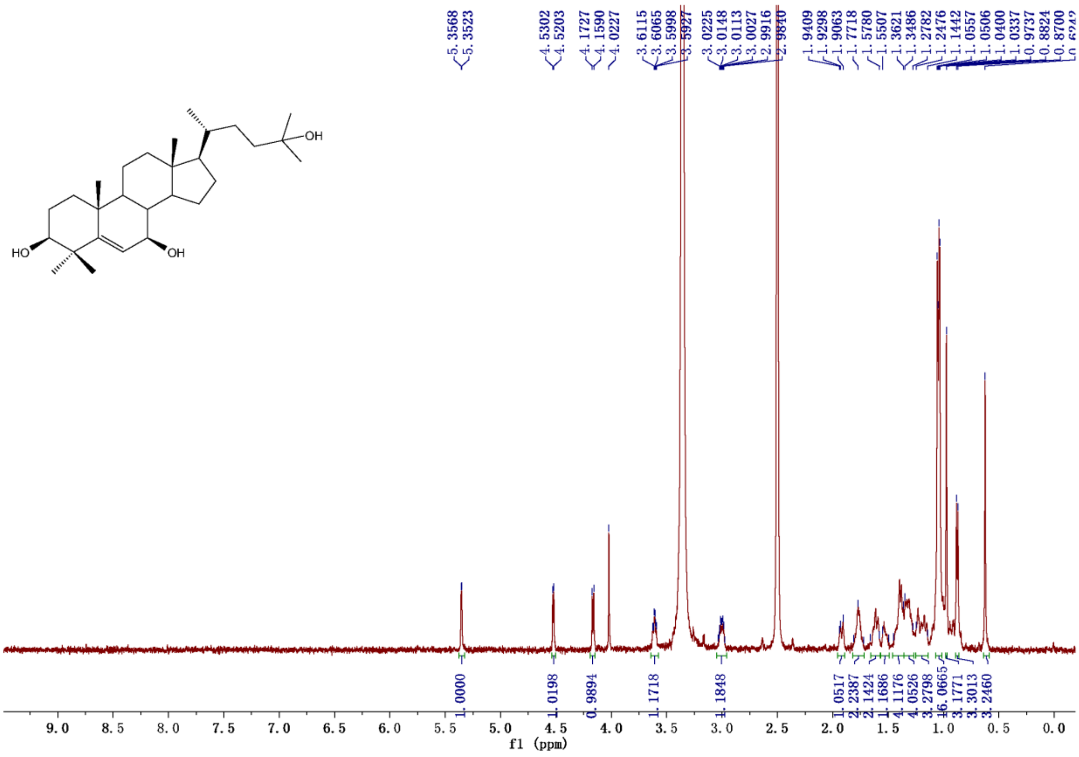

Supplementary Figure 56. Compound 56: ^{1}H-NMR (500 MHz, DMSO-d_6)

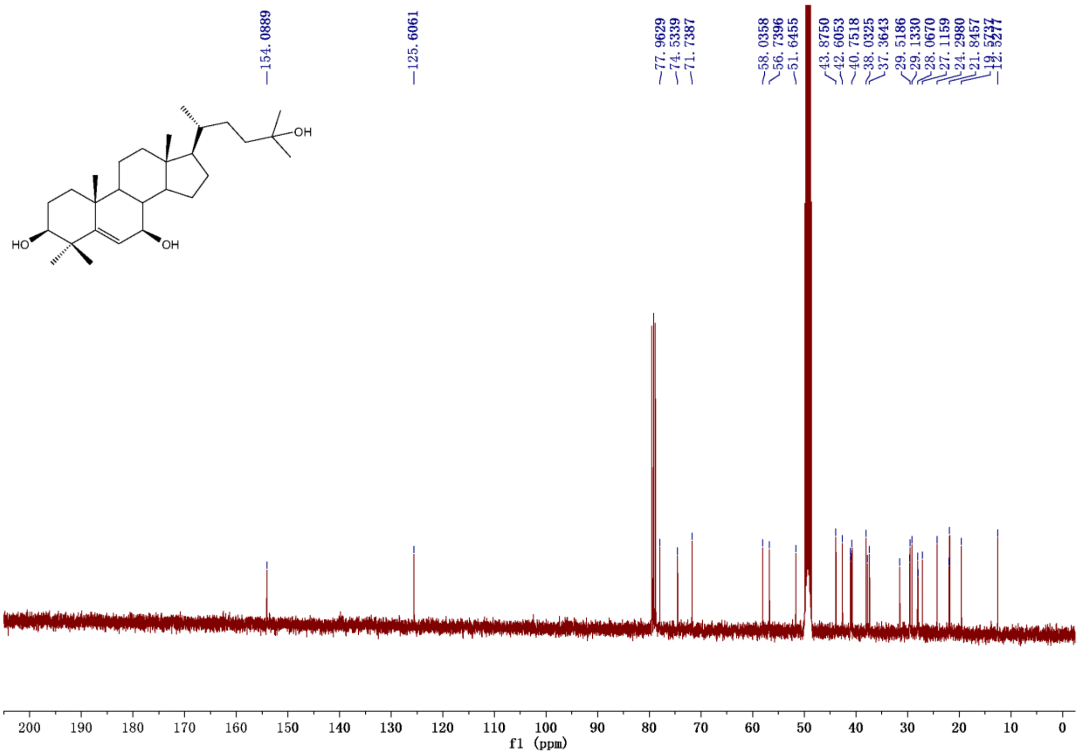

Supplementary Figure 57. Compound 56: ^{13}C-NMR (100 MHz, CD_3OD-d_4 + $CDCl_3$)

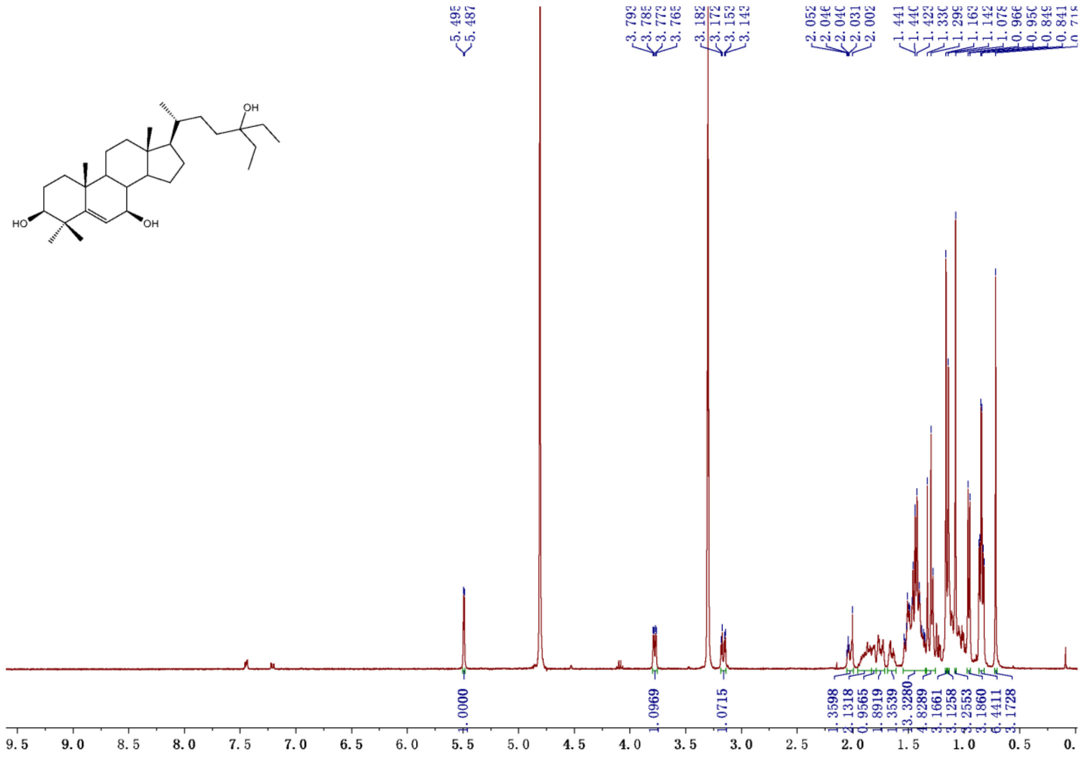

Supplementary Figure 58. Compound 57: ^{1}H-NMR (400 MHz, CD_3OD-d_4)

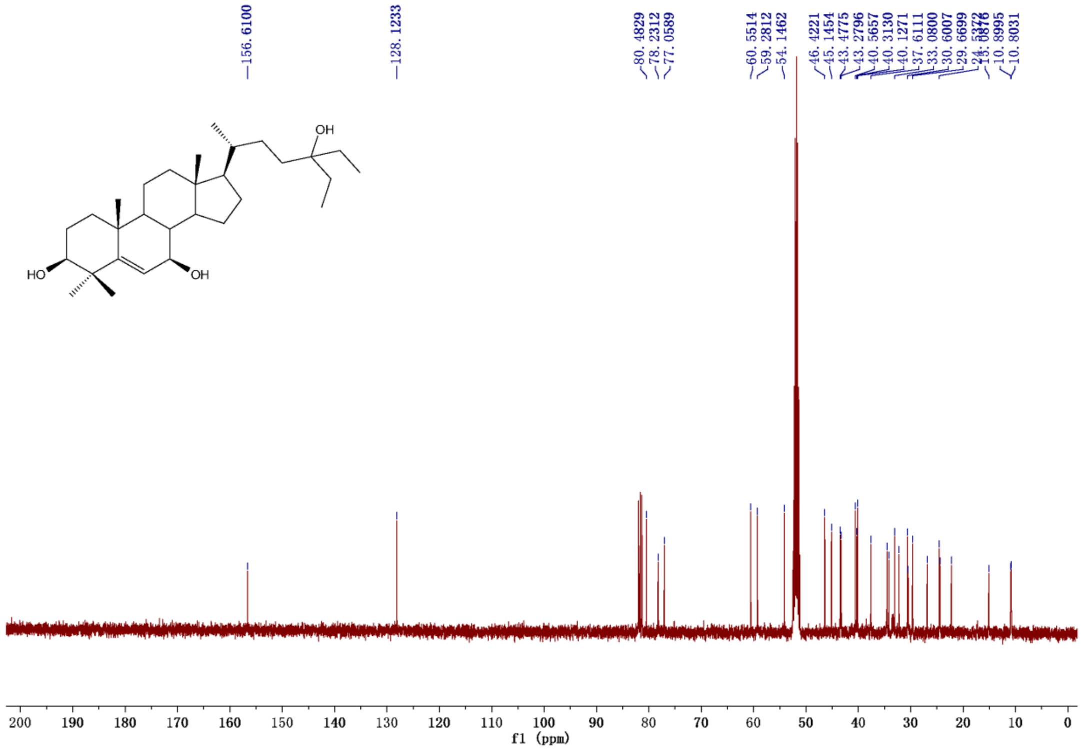

Supplementary Figure 59. Compound 57: ^{13}C-NMR (100 MHz, CD_3OD-d_4 + $CDCl_3$)

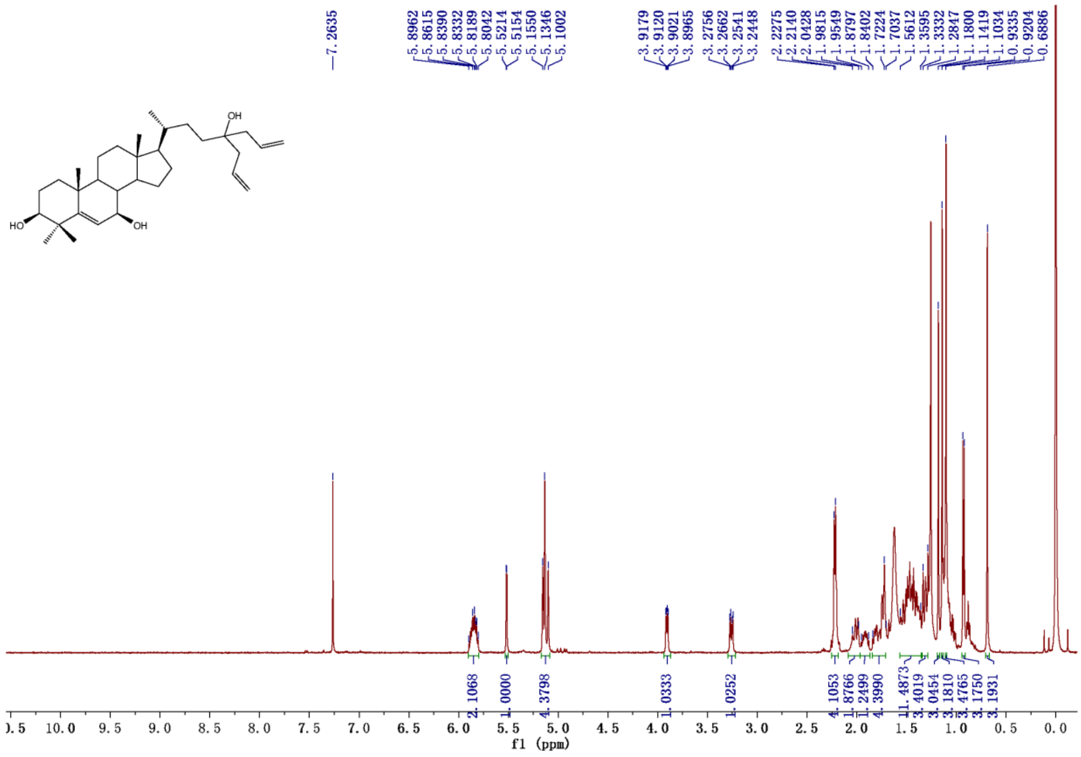

Supplementary Figure 60. Compound 58: ^{1}H-NMR (500 MHz, $CDCl_3$)

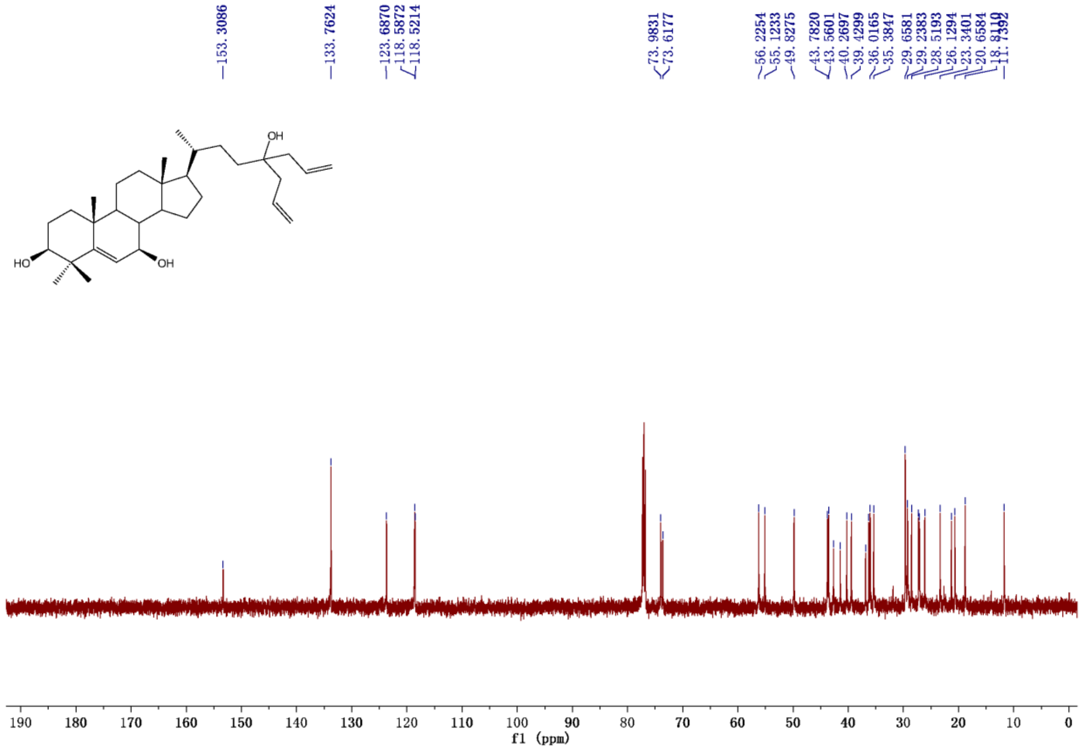

Supplementary Figure 61. Compound 58: ^{13}C-NMR (125 MHz, $CDCl_3$)

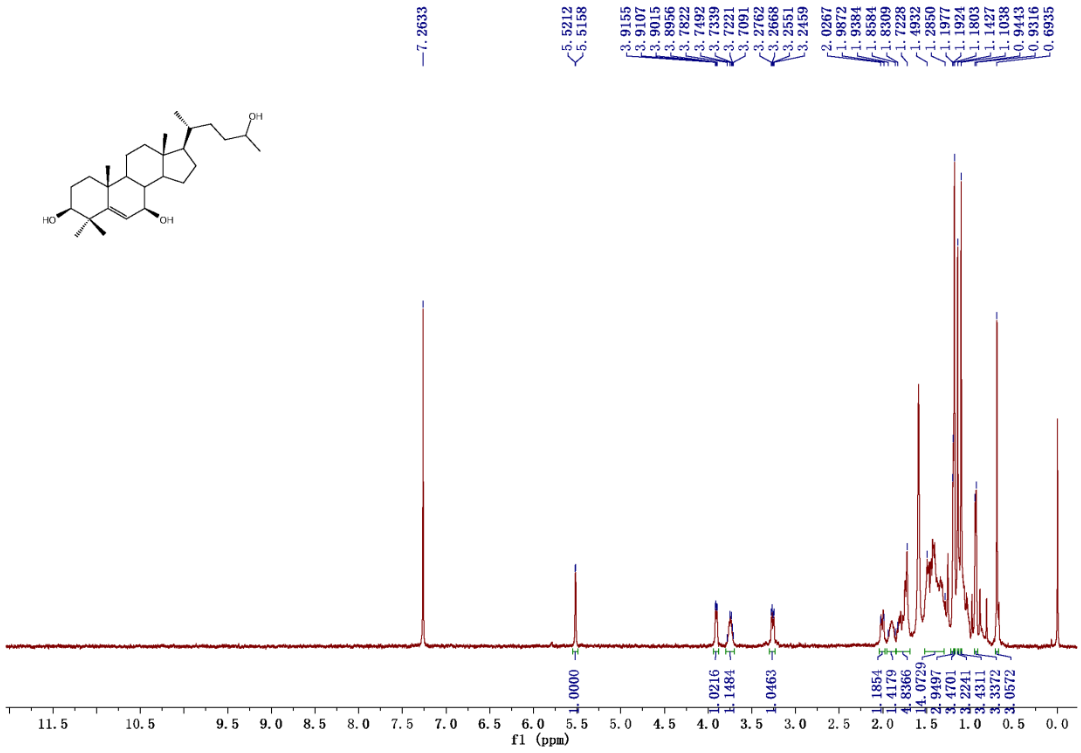

Supplementary Figure 62. Compound 63: ^{1}H-NMR (500 MHz, $CDCl_3$)

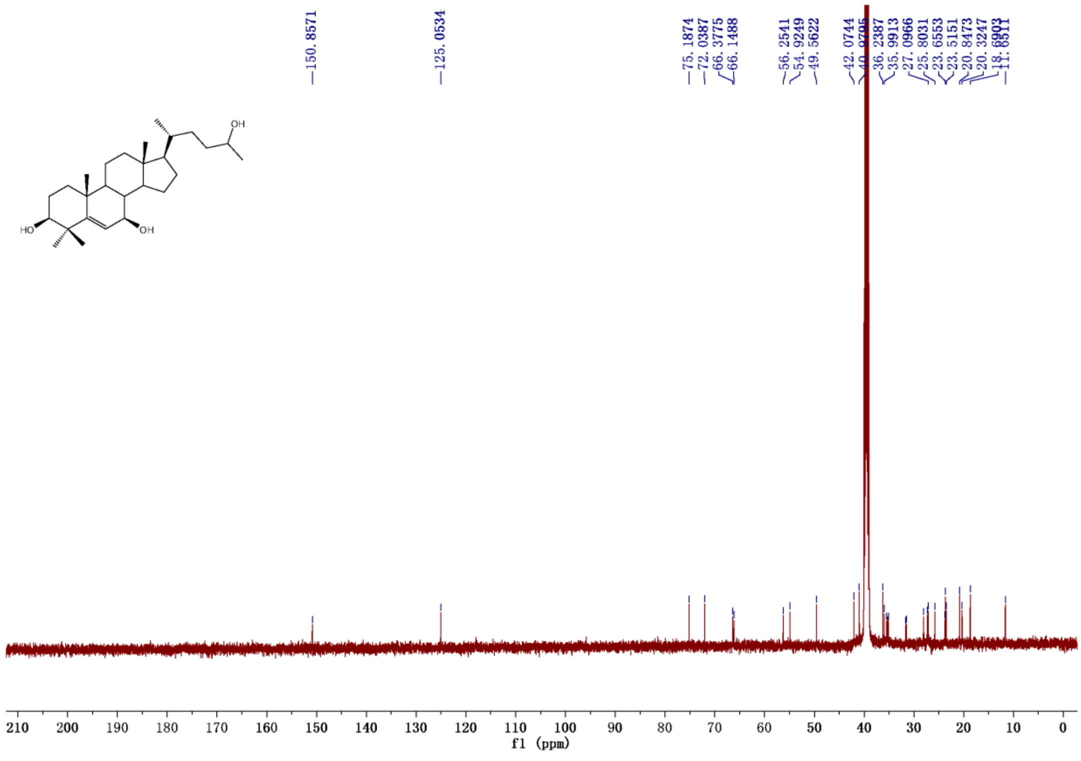

Supplementary Figure 63. Compound 63: ^{13}C-NMR (125 MHz, DMSO-d_6)

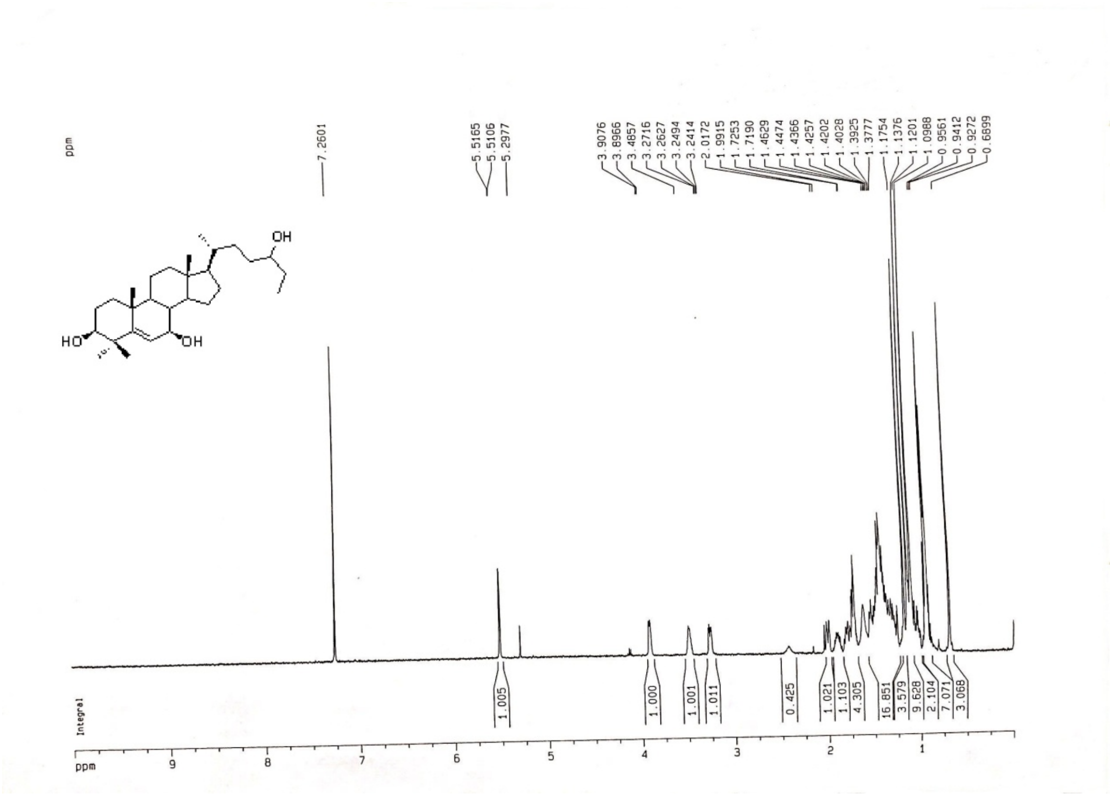

Supplementary Figure 64. Compound 64: ^{1}H-NMR (500 MHz, $CDCl_3$)

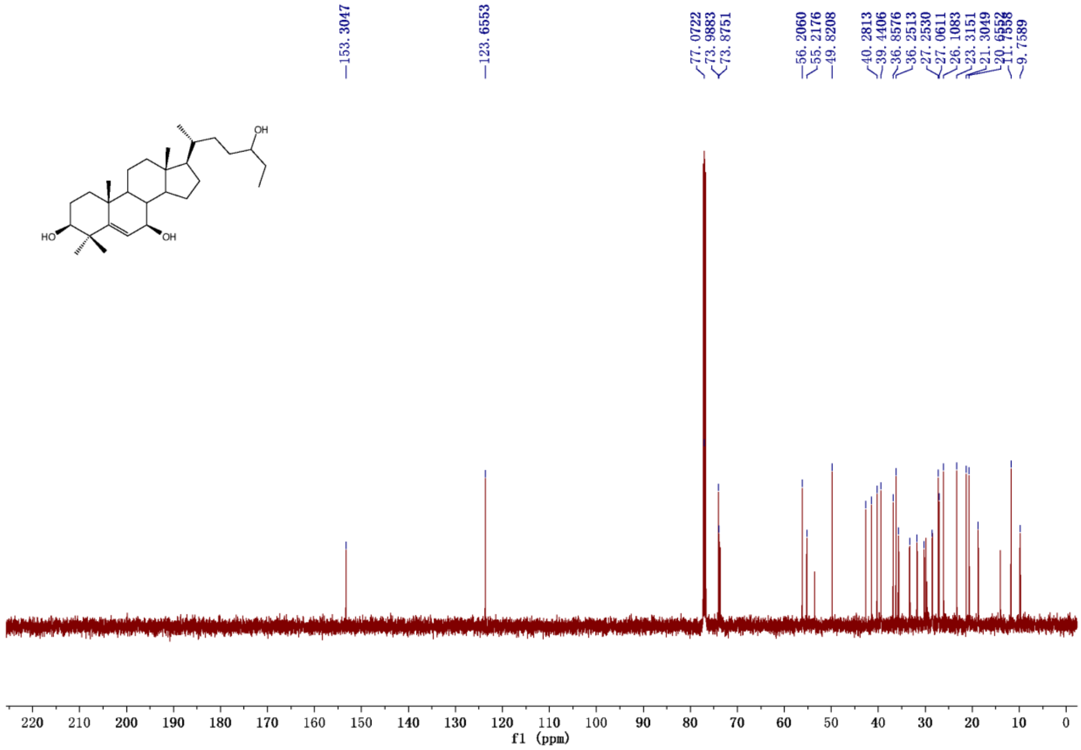

Supplementary Figure 65. Compound 64: ^{13}C-NMR (125 MHz, $CDCl_3$)

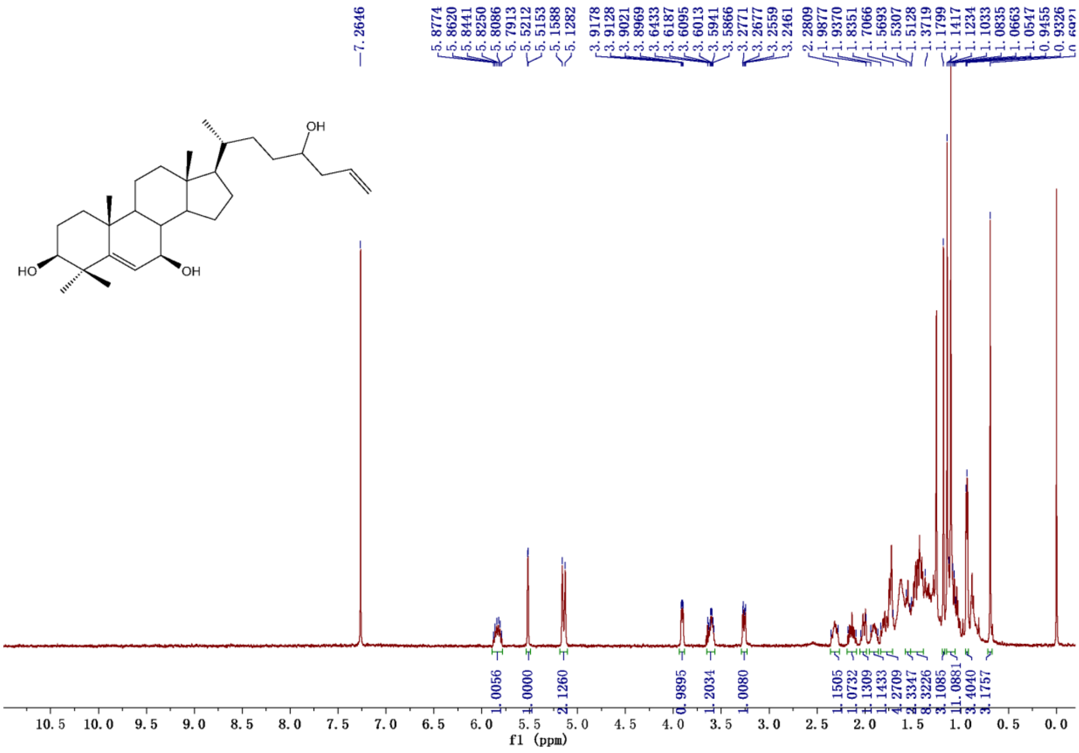

Supplementary Figure 66. Compound 65: ^{1}H-NMR (500 MHz, $CDCl_3$)

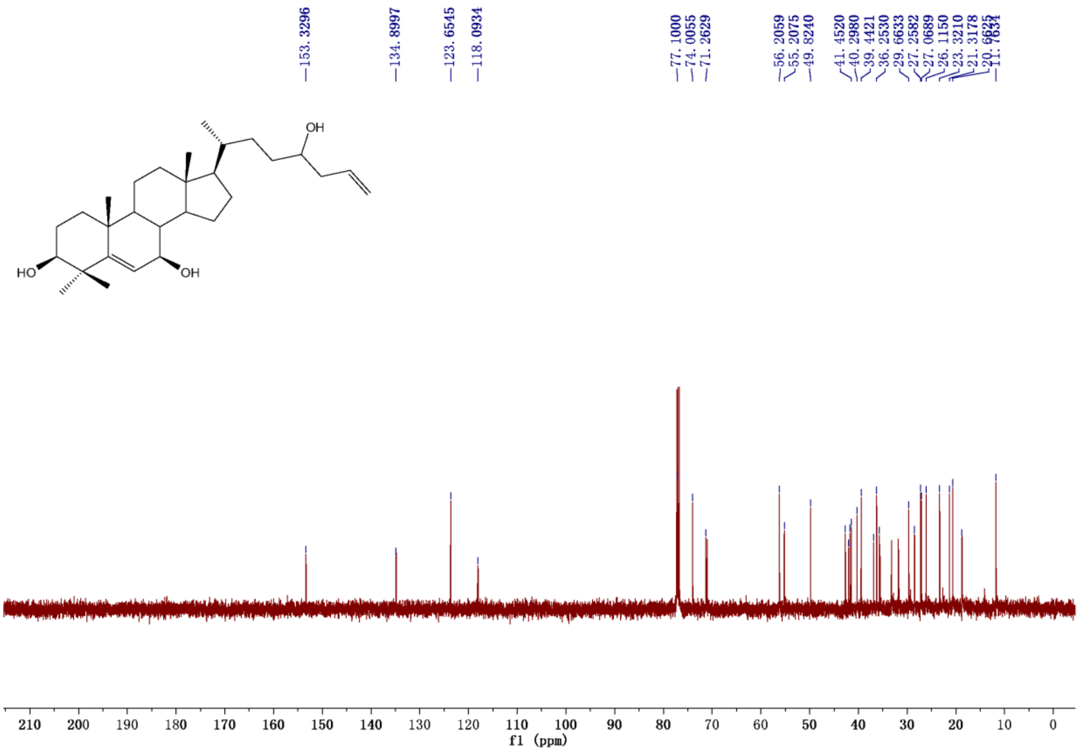

Supplementary Figure 67. Compound 65: ^{13}C-NMR (125 MHz, $CDCl_3$)

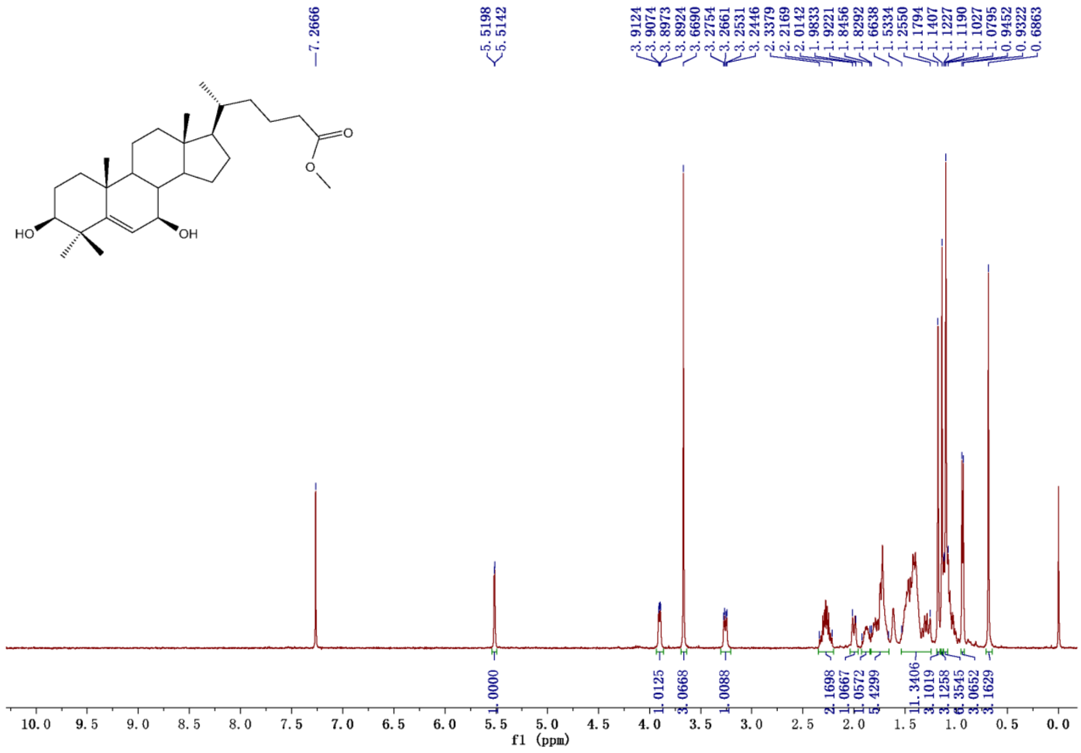

Supplementary Figure 68. Compound 72: ^{1}H-NMR (500 MHz, $CDCl_3$)

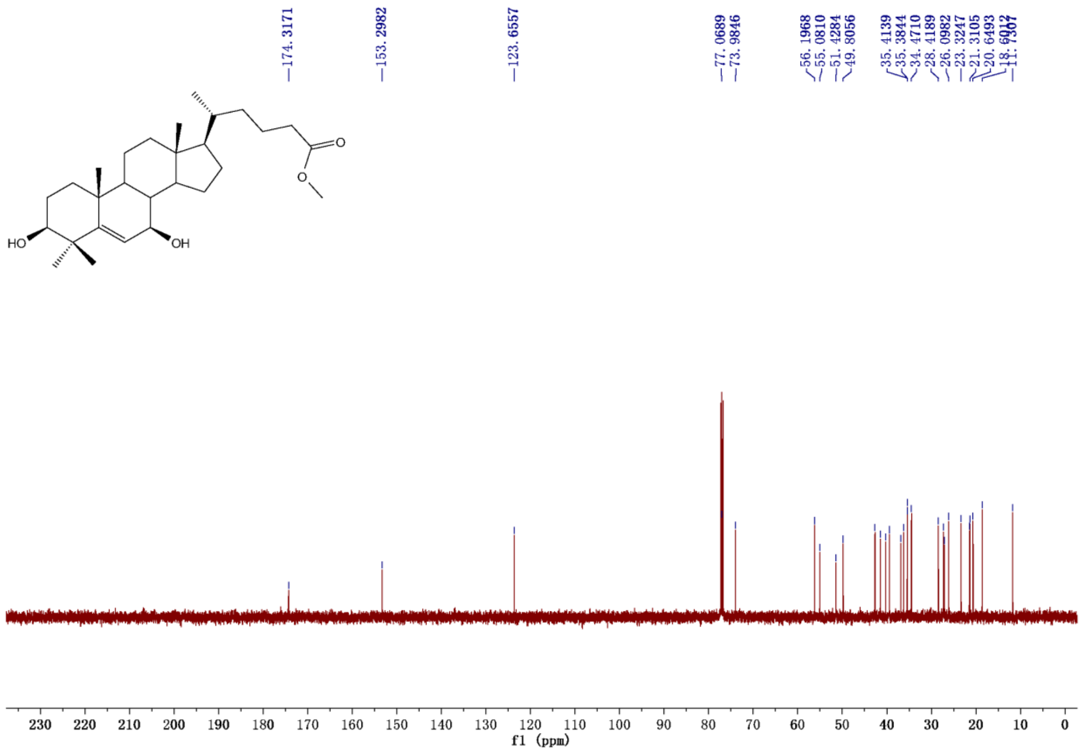

Supplementary Figure 69. Compound 72: ^{13}C-NMR (125 MHz, $CDCl_3$)

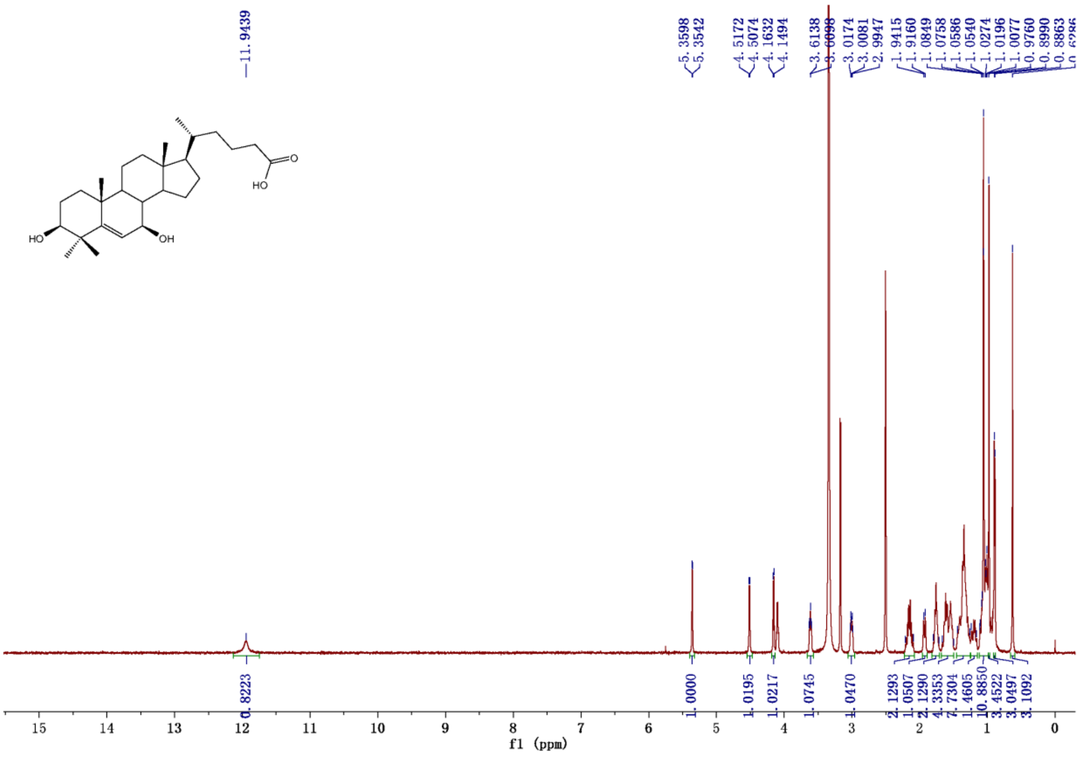

Supplementary Figure 70. Compound 73: ^{1}H-NMR (500 MHz, DMSO-d_6)

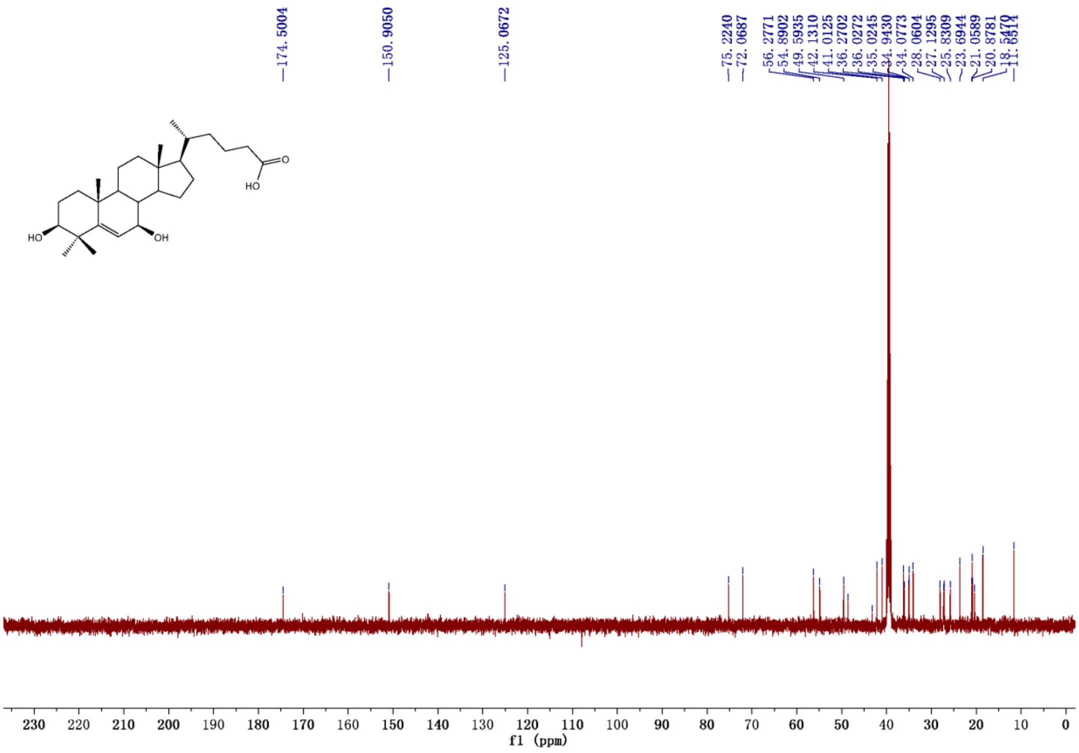

Supplementary Figure 71. Compound 73: ^{13}C-NMR (125 MHz, DMSO-d_6)

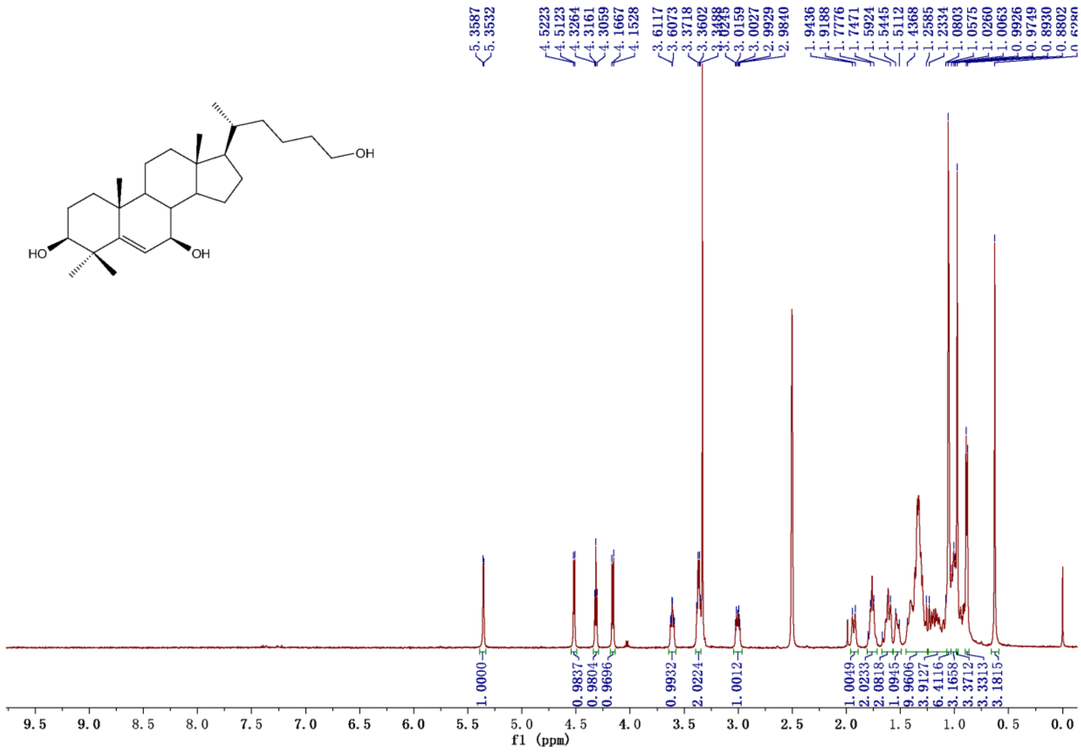

Supplementary Figure 72. Compound 74: ^{1}H-NMR (500 MHz, DMSO-d_6)

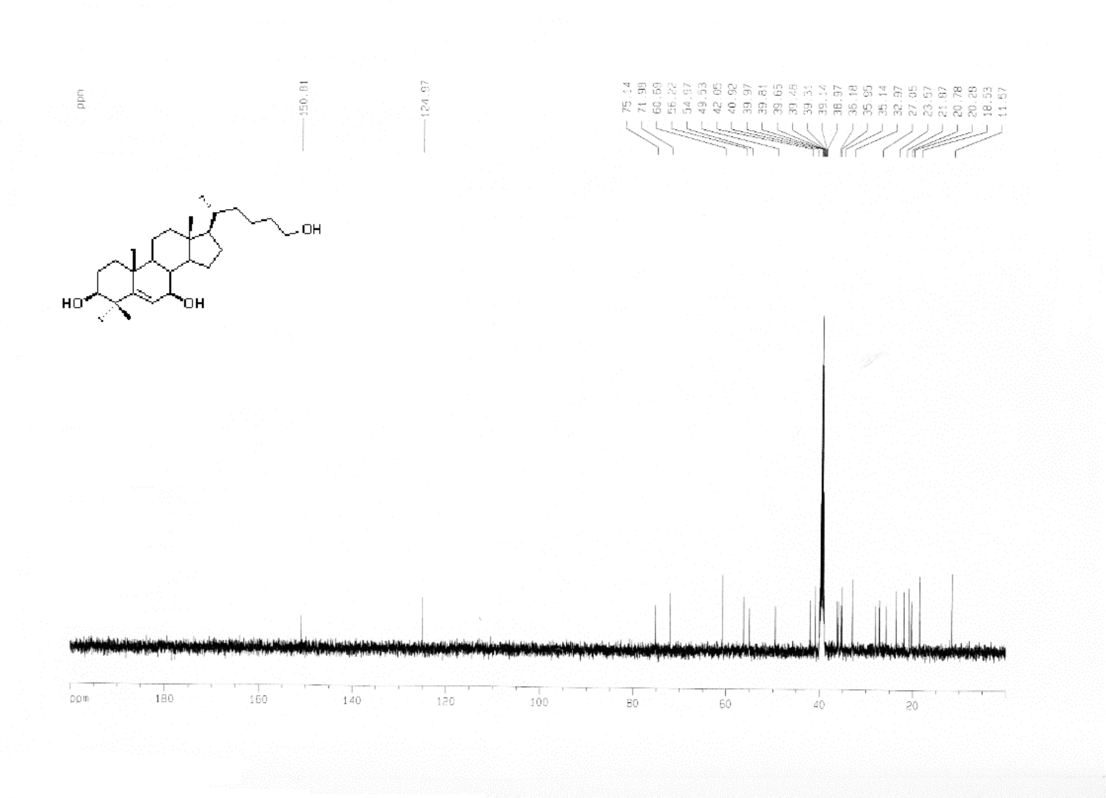

Supplementary Figure 73. Compound 74: ^{13}C-NMR (125 MHz, DMSO-d_6)

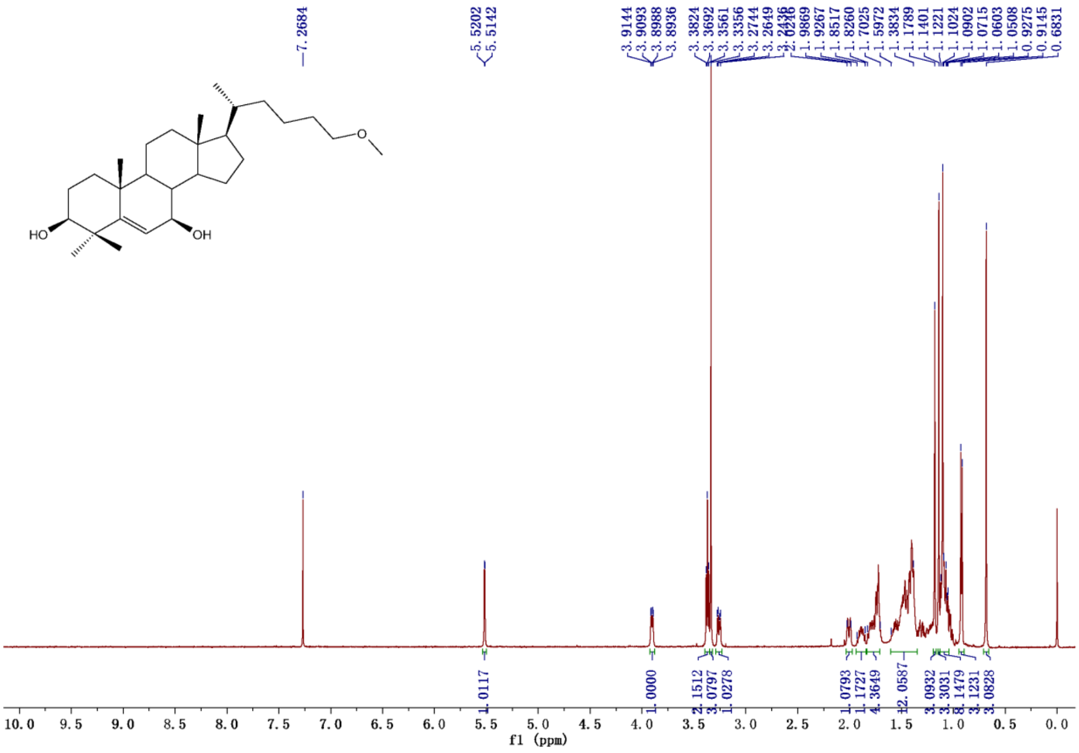

Supplementary Figure 74. Compound 78: ^{1}H-NMR (500 MHz, $CDCl_3$)

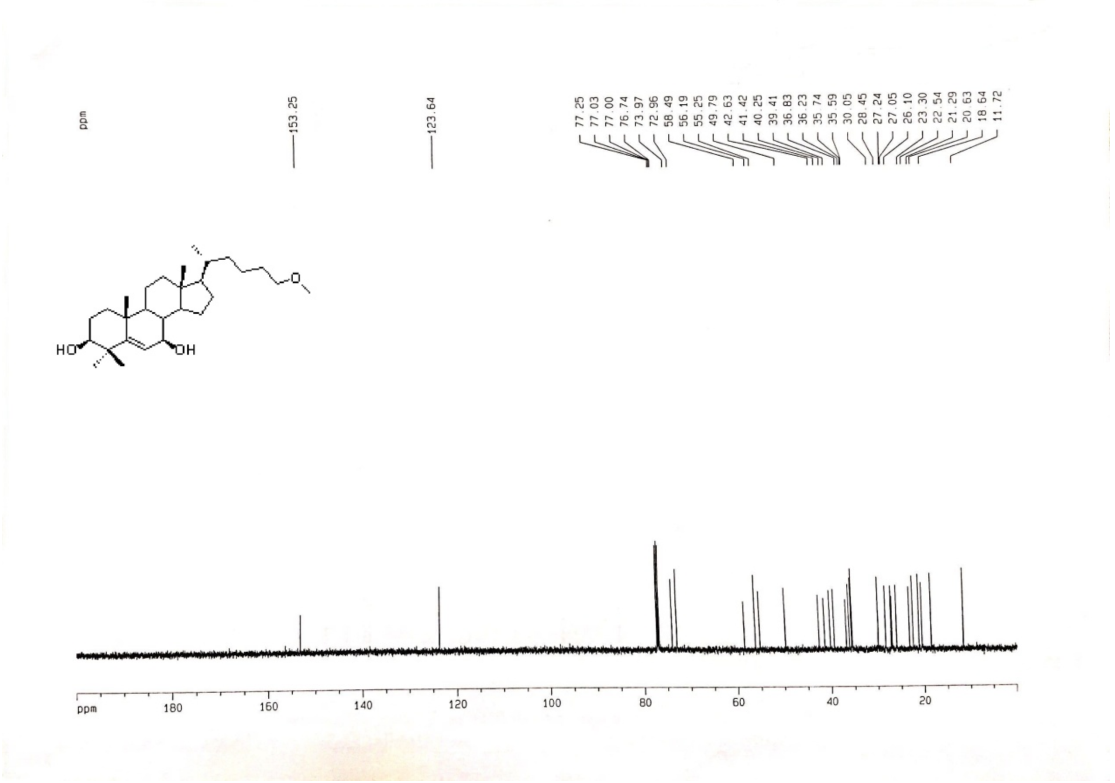

Supplementary Figure 75. Compound 78: ^{13}C-NMR (125 MHz, $CDCl_3$)

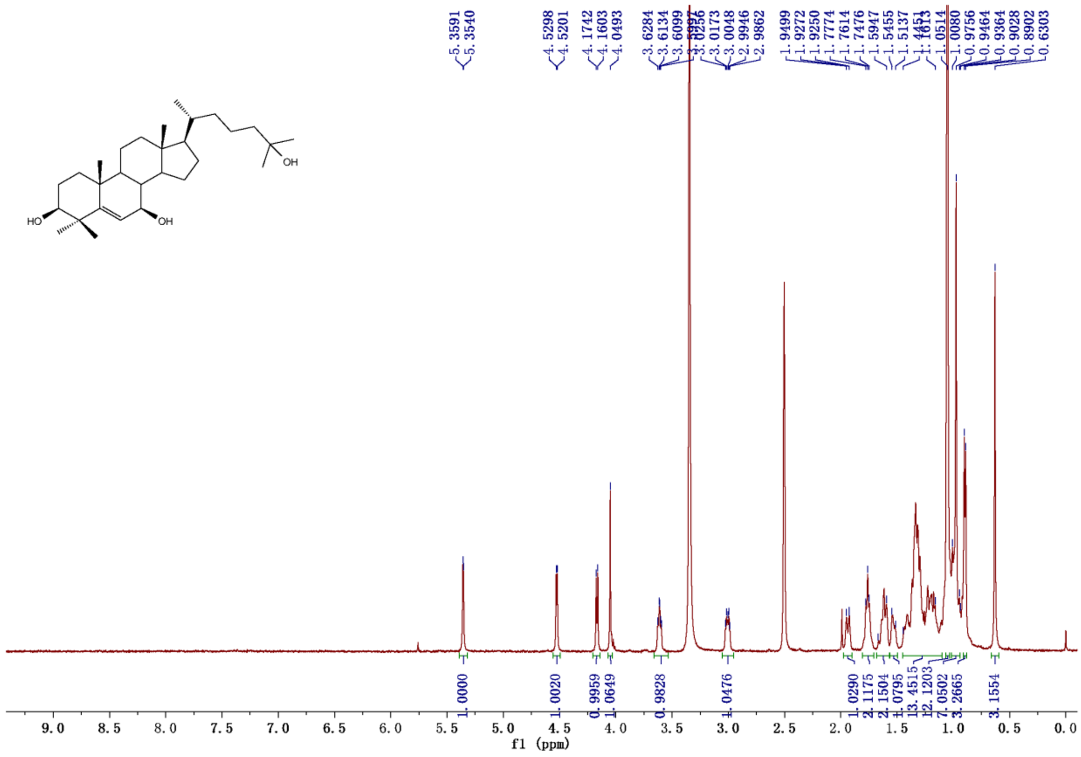

Supplementary Figure 76. Compound 79: ^{1}H-NMR (500 MHz, DMSO-d_6)

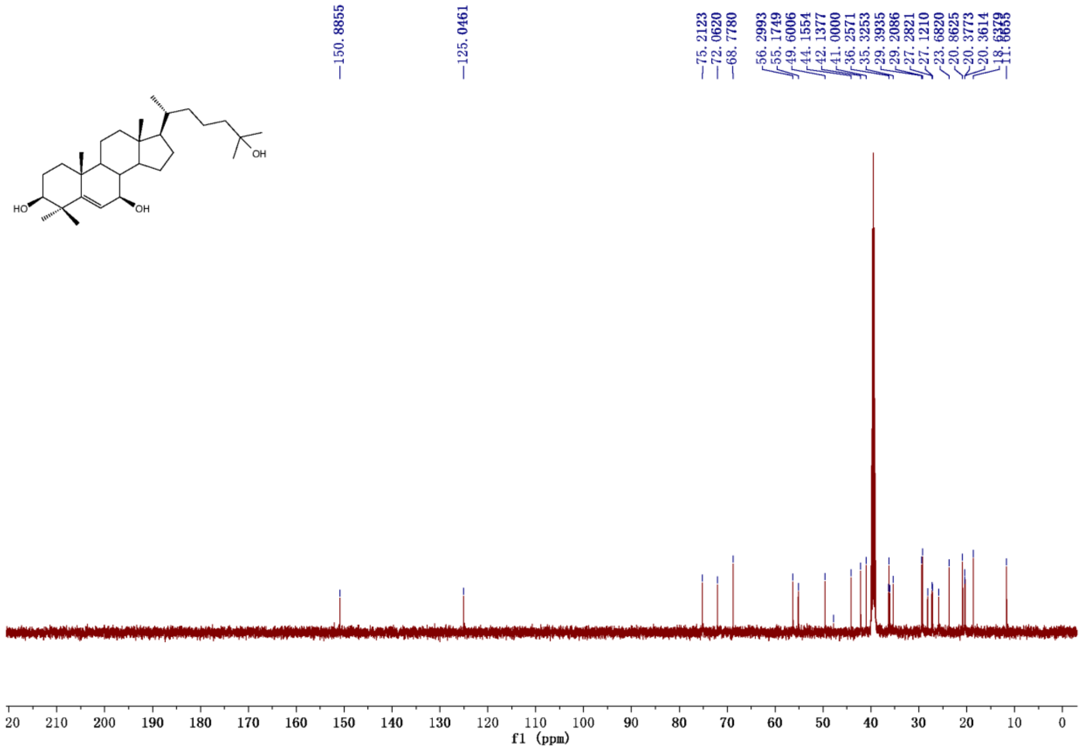

Supplementary Figure 77. Compound 79: ^{13}C-NMR (125 MHz, DMSO-d_6)

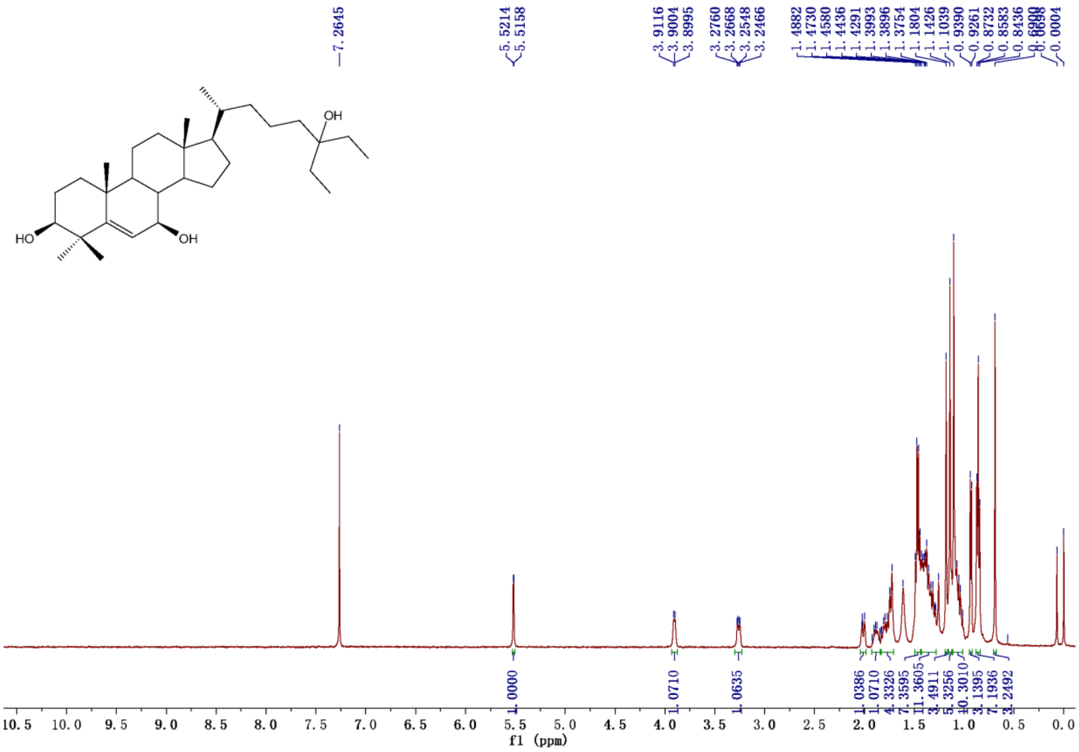

Supplementary Figure 78. Compound 80: ^{1}H-NMR (500 MHz, $CDCl_3$)

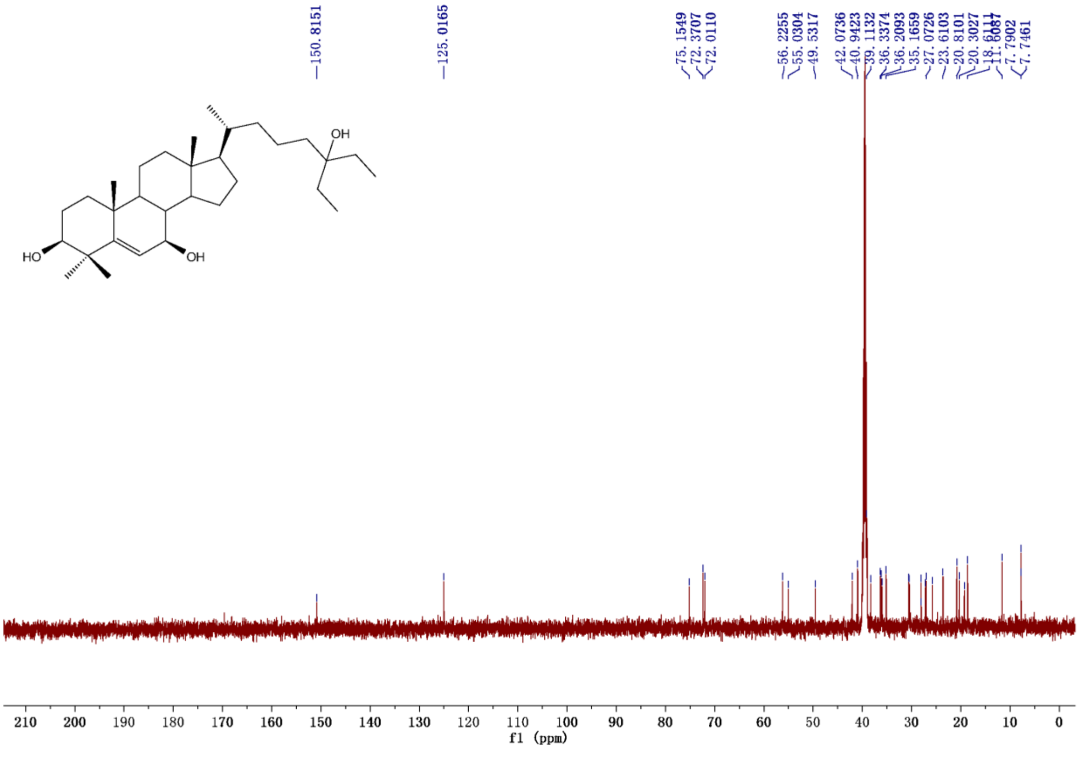

Supplementary Figure 79. Compound 80: ^{13}C-NMR (125 MHz, DMSO-d_6)

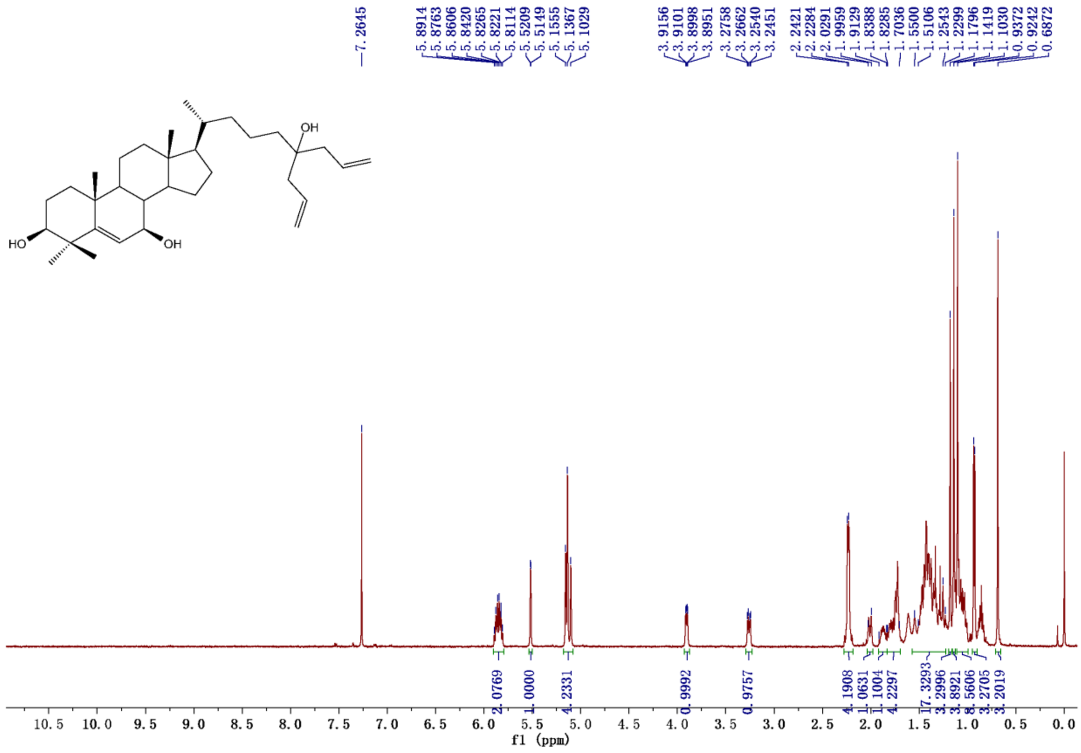

Supplementary Figure 80. Compound 81(HMG499): ^{1}H-NMR (500 MHz, $CDCl_3$)

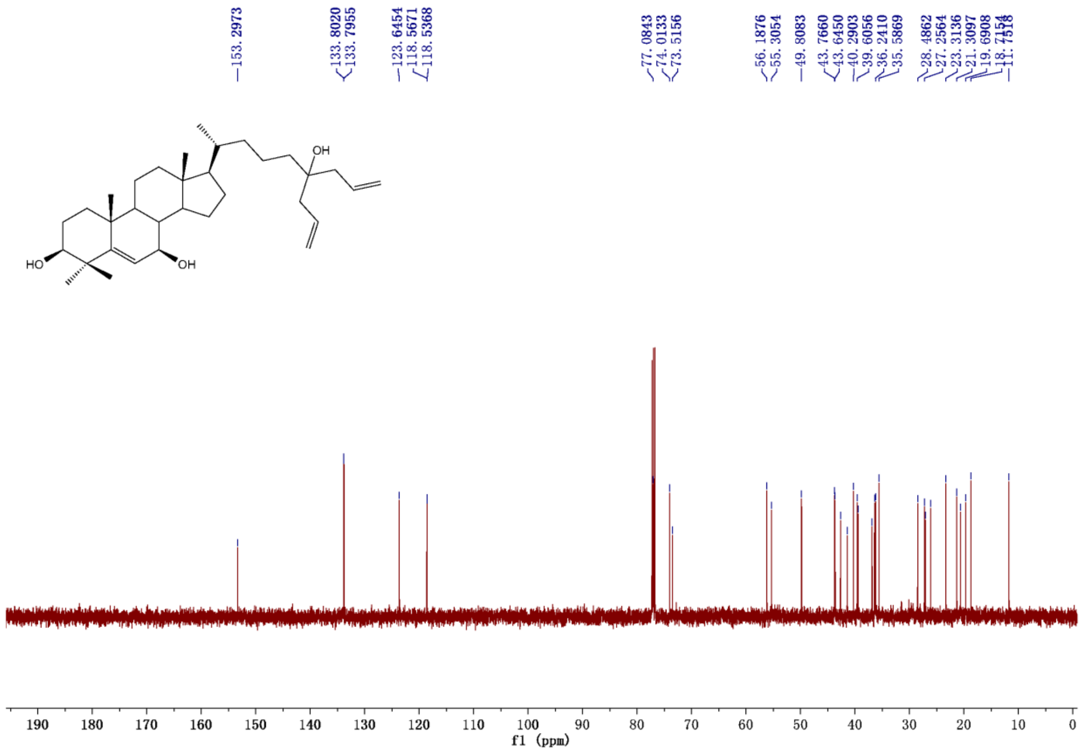

Supplementary Figure 81. Compound 81(HMG499): ^{13}C-NMR (125 MHz, $CDCl_3$)

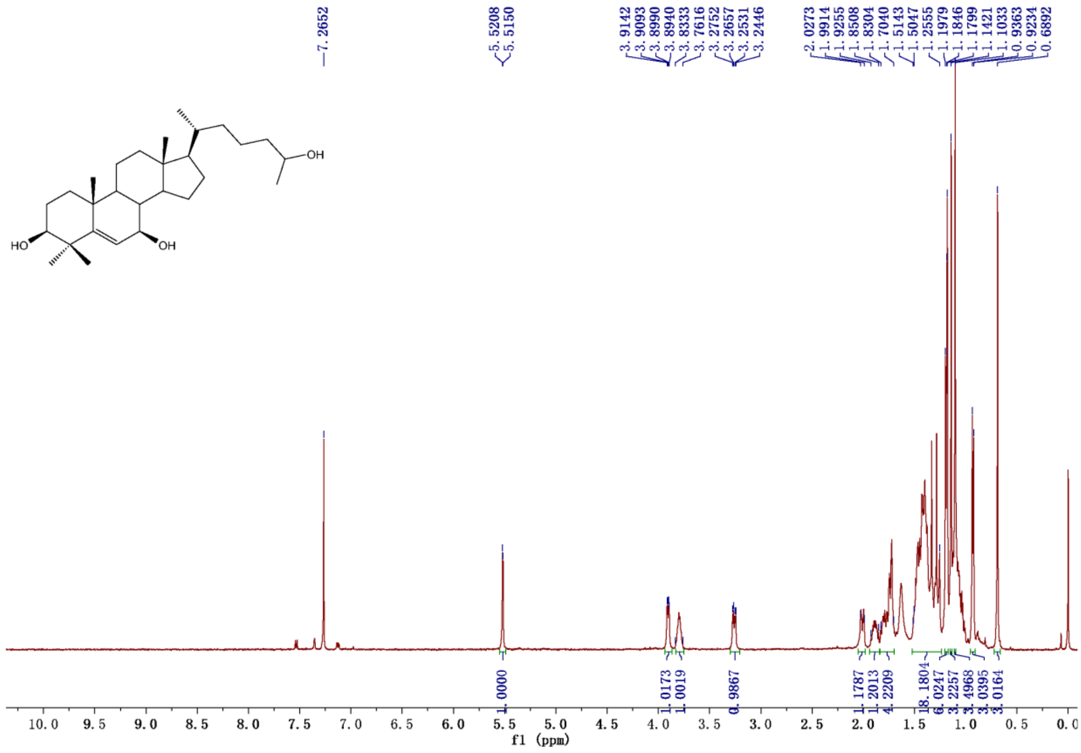

Supplementary Figure 82. Compound 86: ^{1}H-NMR (500 MHz, $CDCl_3$)

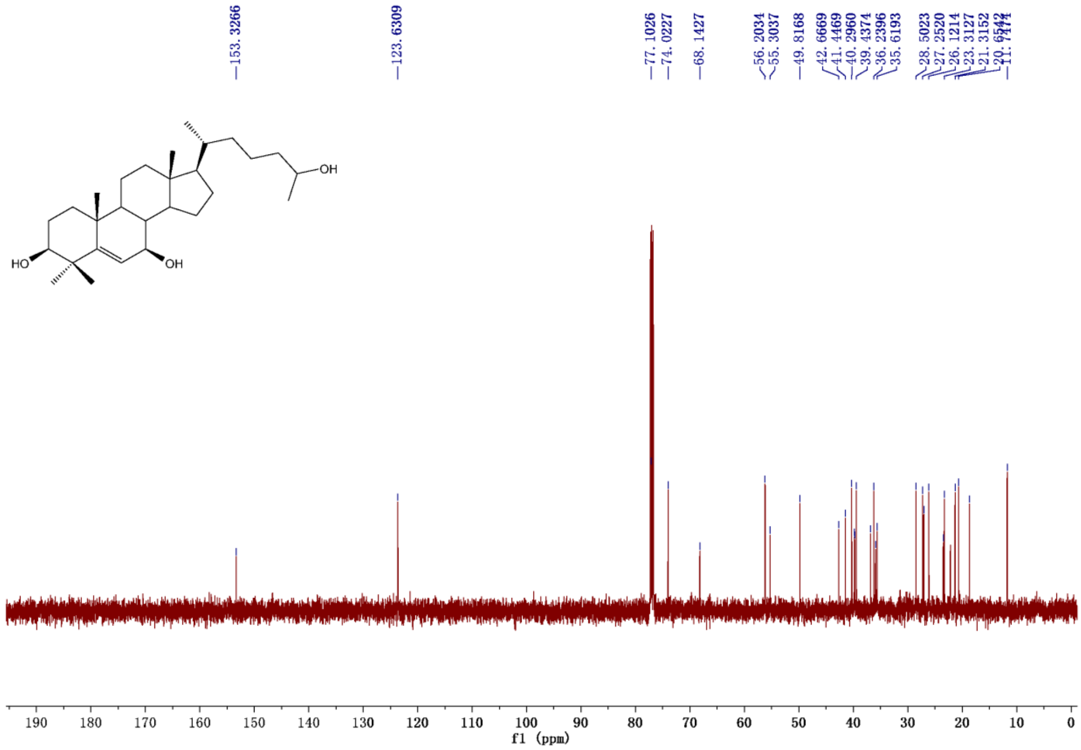

Supplementary Figure 83. Compound 86: ^{13}C-NMR (125 MHz, $CDCl_3$)

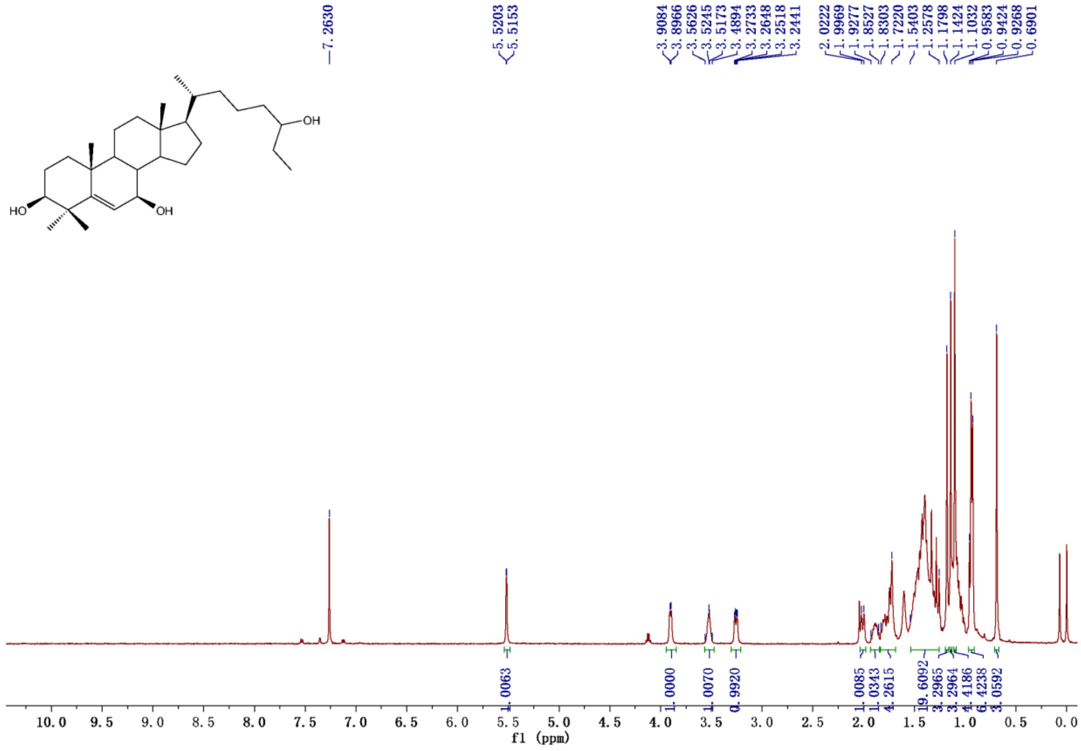

Supplementary Figure 84. Compound 87: ^{1}H-NMR (500 MHz, $CDCl_3$)

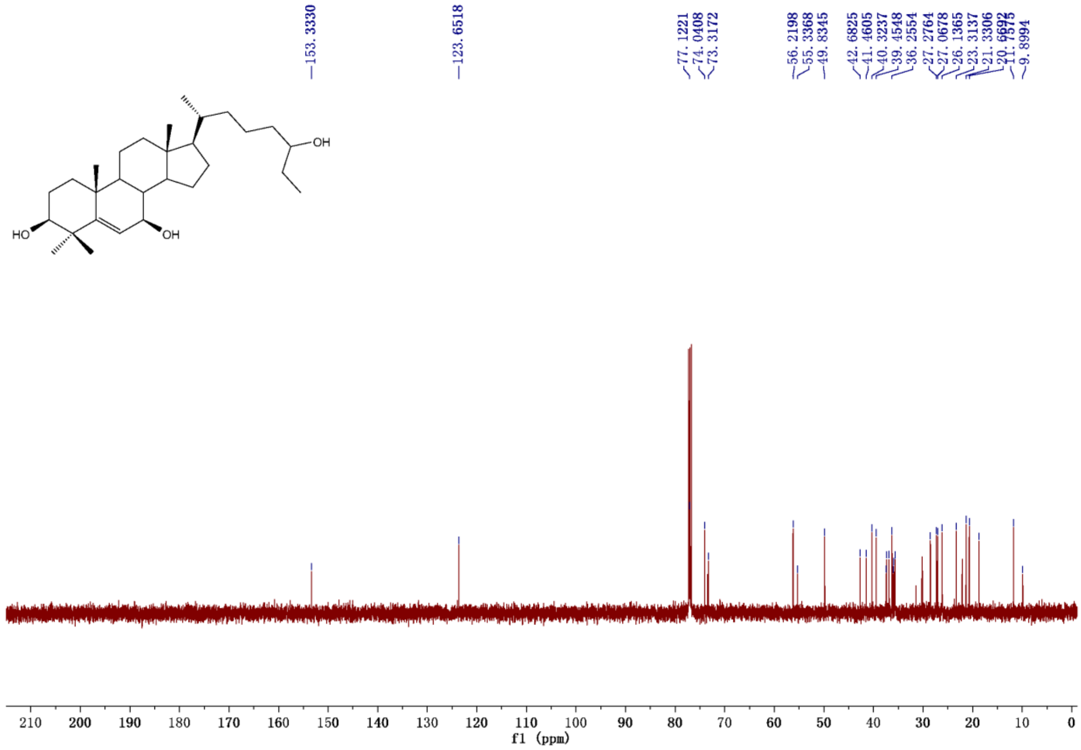

Supplementary Figure 85. Compound 87: ^{13}C-NMR (125 MHz, $CDCl_3$)

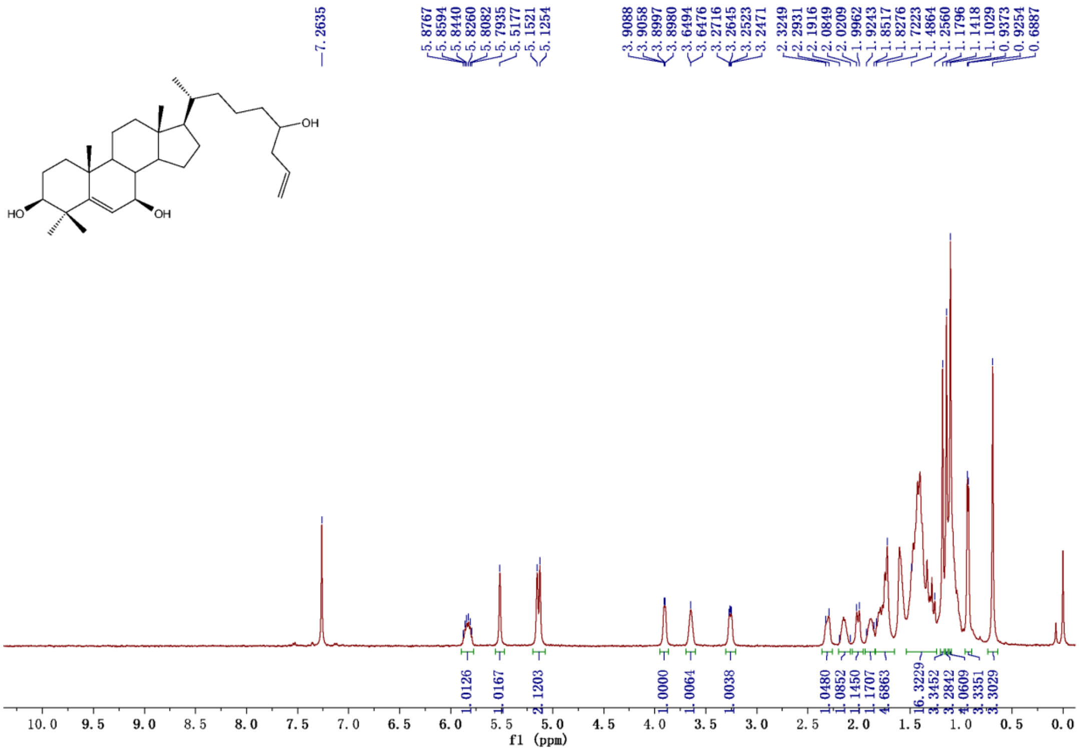

Supplementary Figure 86. Compound 88: ^{1}H-NMR (500 MHz, CDCl₃)

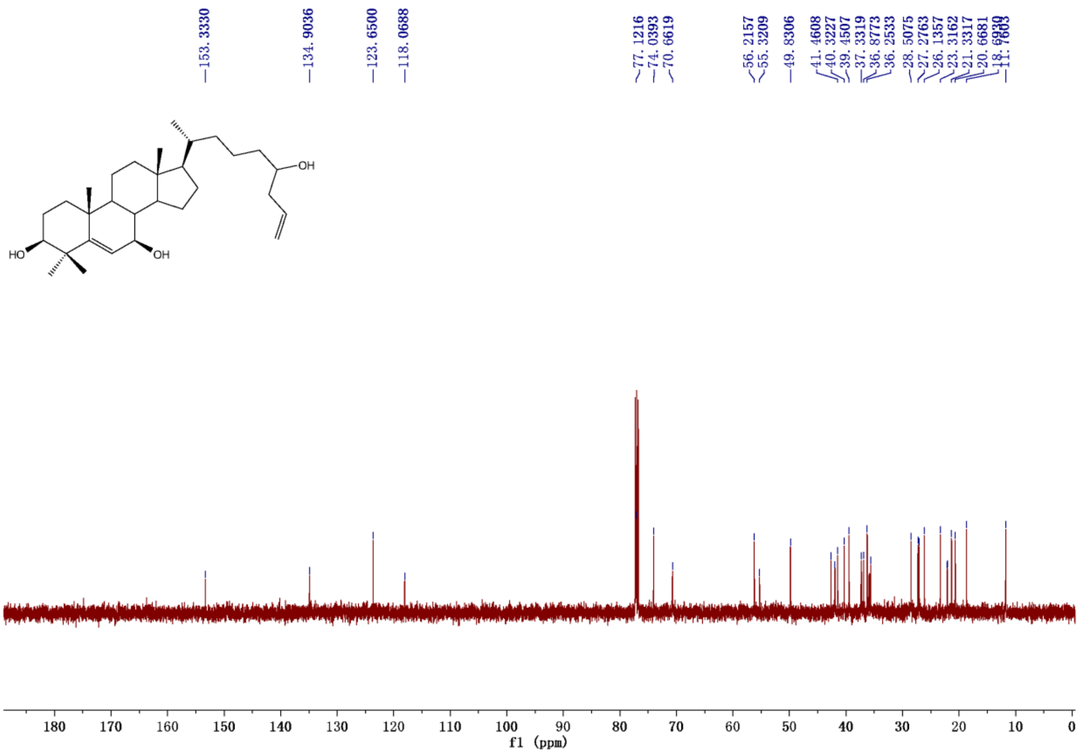

Supplementary Figure 87. Compound 88: ^{13}C-NMR (125 MHz, CDCl₃)

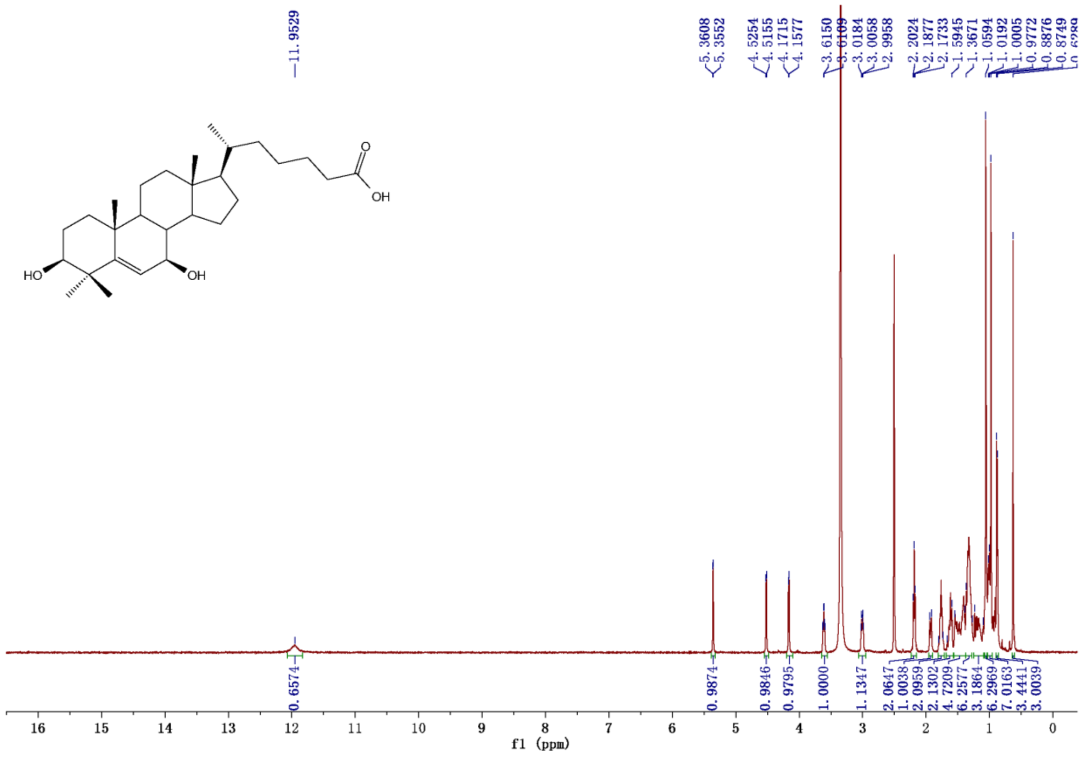

Supplementary Figure 88. Compound 92: ^{1}H-NMR (500 MHz, DMSO-d_6)

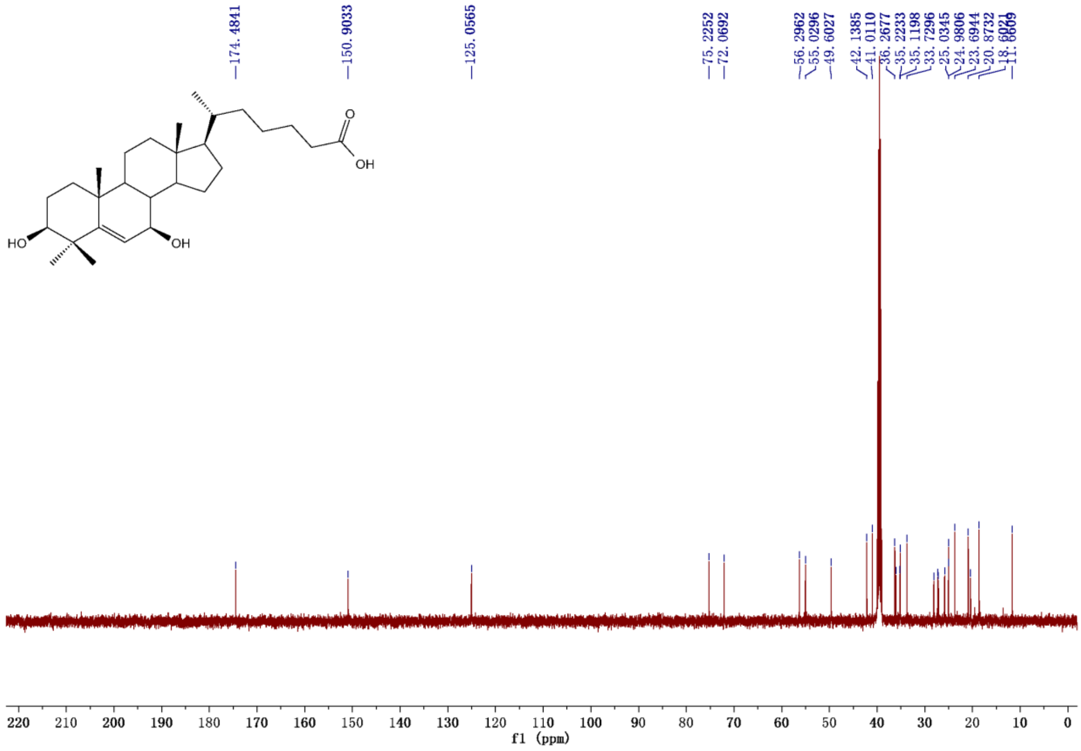

Supplementary Figure 89. Compound 92: ^{13}C-NMR (125 MHz, DMSO-d_6)

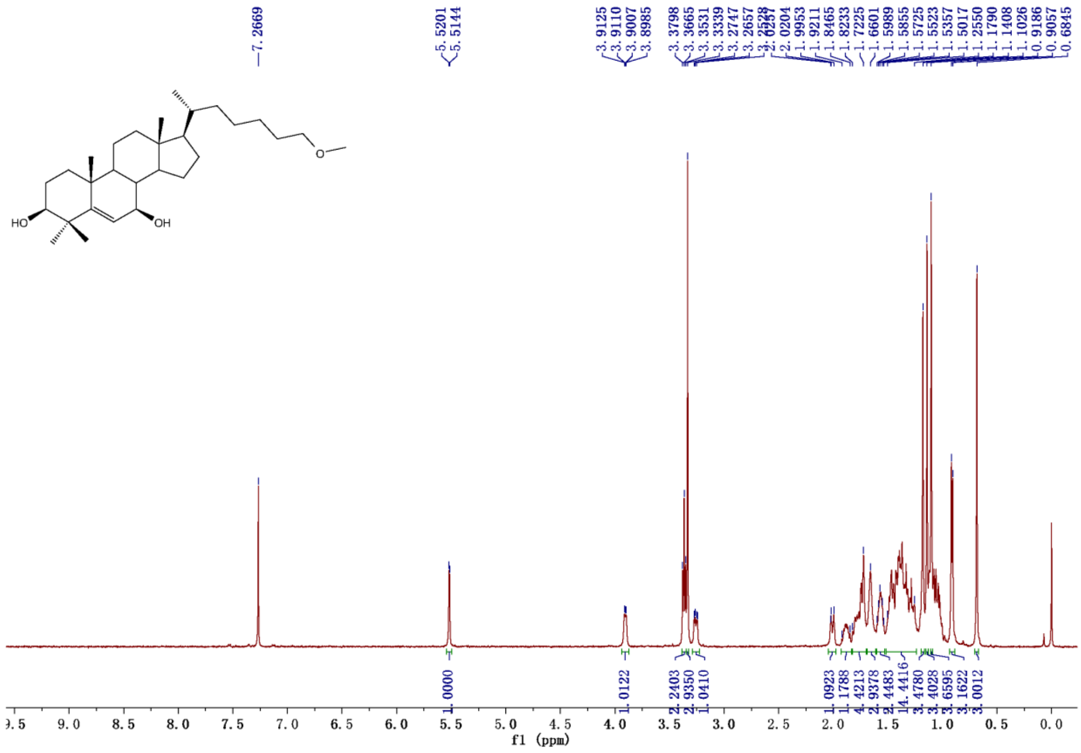

Supplementary Figure 90. Compound 95: ^{1}H-NMR (500 MHz, $CDCl_3$)

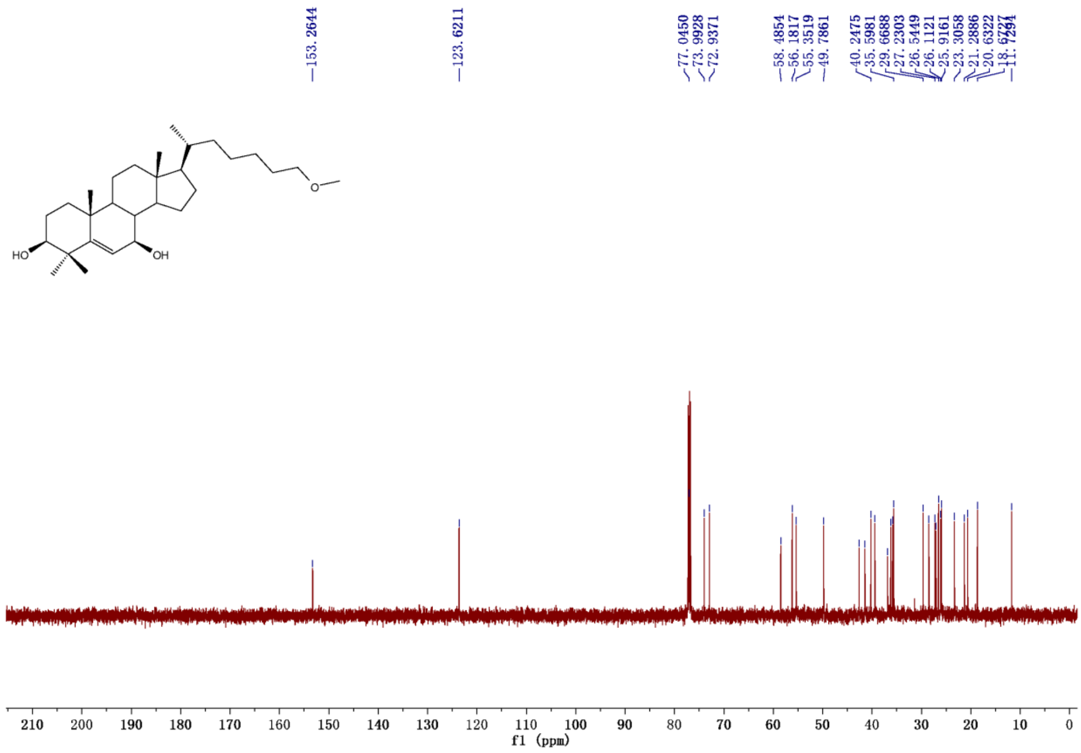

Supplementary Figure 91. Compound 95: ^{13}C-NMR (125 MHz, $CDCl_3$)

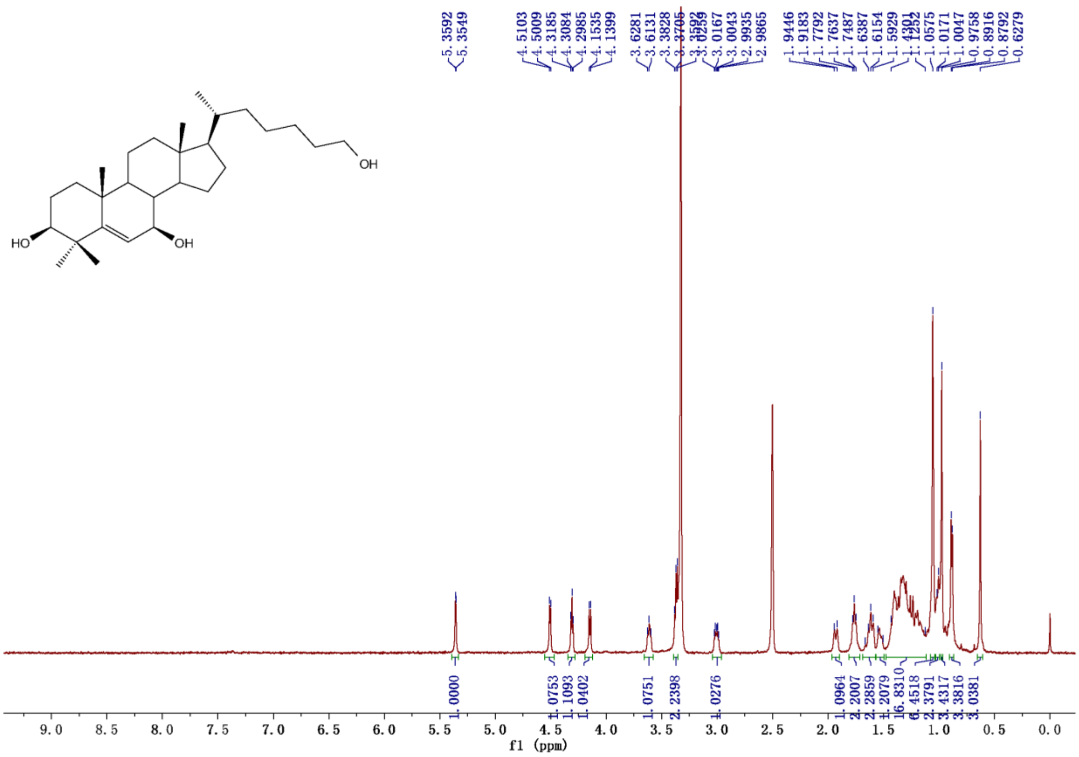

Supplementary Figure 92. Compound 96: ¹H-NMR (500 MHz, DMSO-d_6)

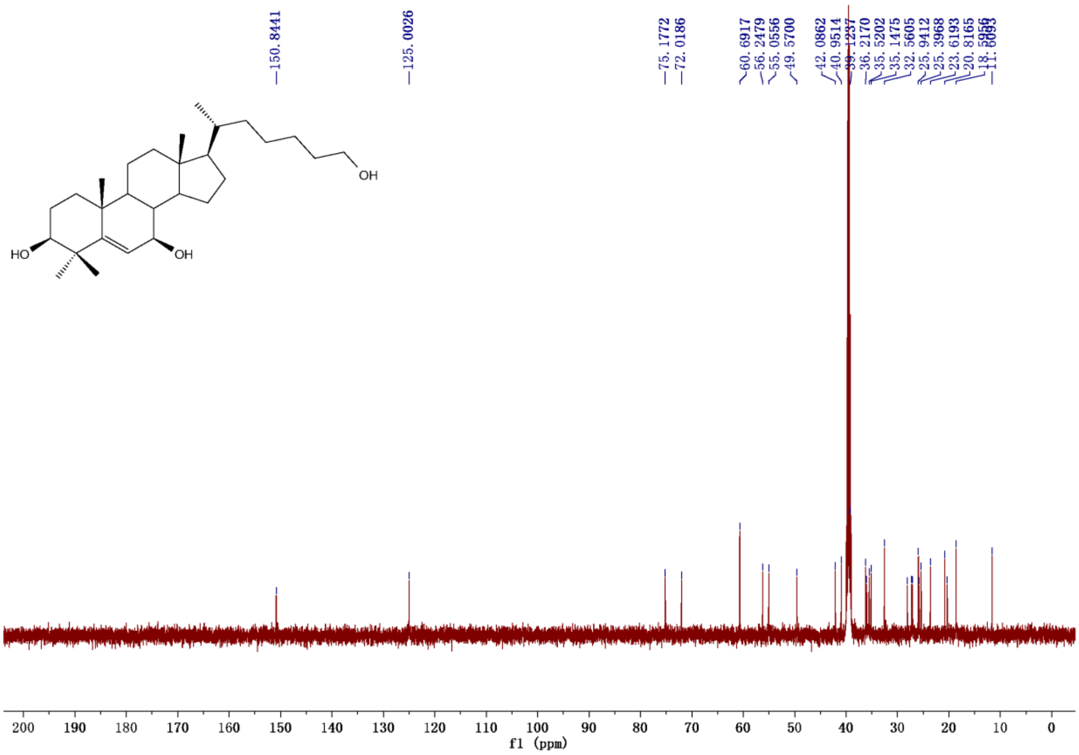

Supplementary Figure 93. Compound 96: ¹³C-NMR (125 MHz, DMSO-d_6)

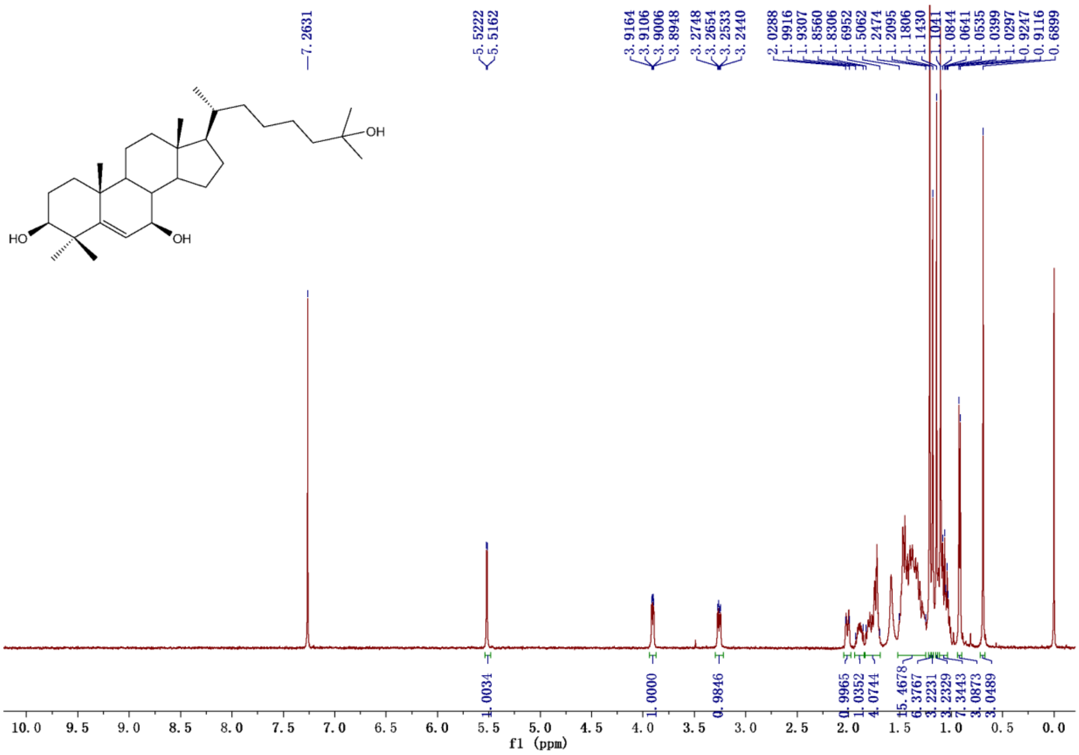

Supplementary Figure 94. Compound 100: ^{1}H-NMR (500 MHz, $CDCl_3$)

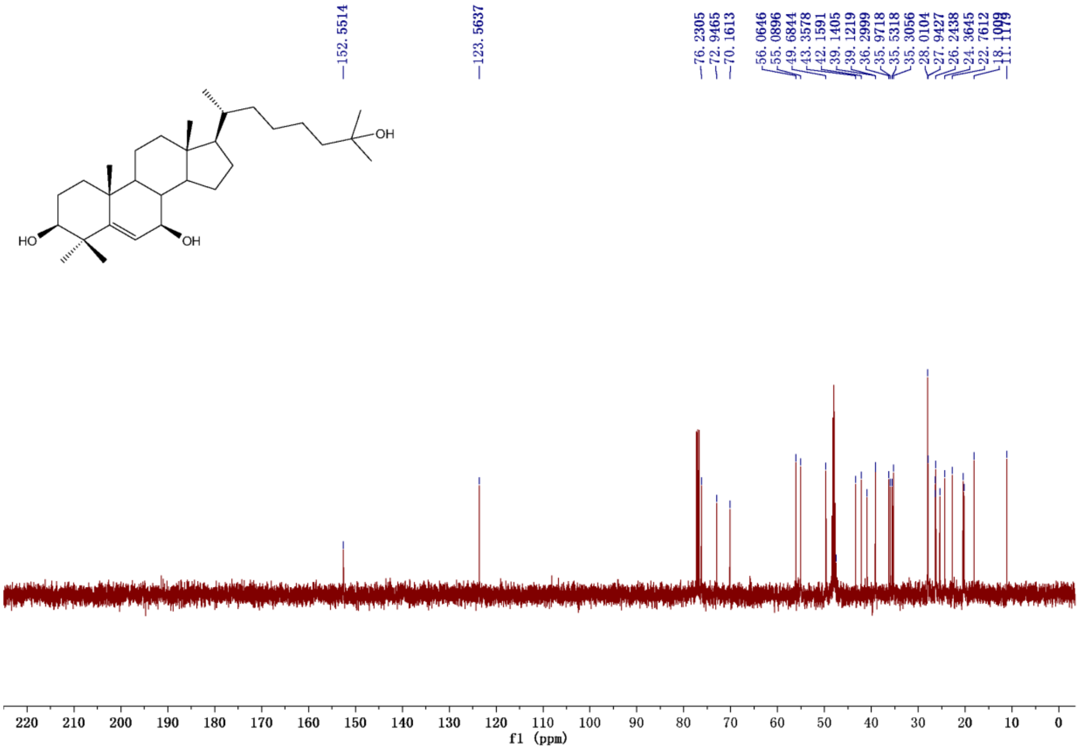

Supplementary Figure 95. Compound 100: ^{13}C-NMR (125 MHz, $CDCl_3$ + CD_3OD-d_4)

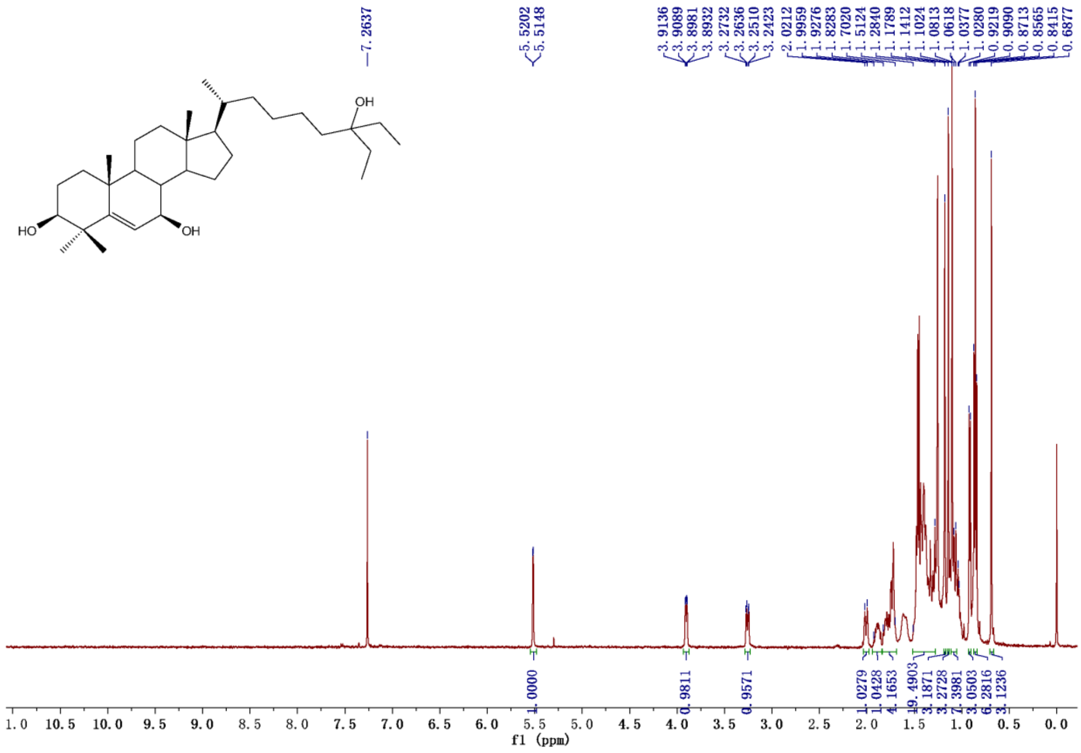

Supplementary Figure 96. Compound 101: ^{1}H-NMR (500 MHz, $CDCl_3$)

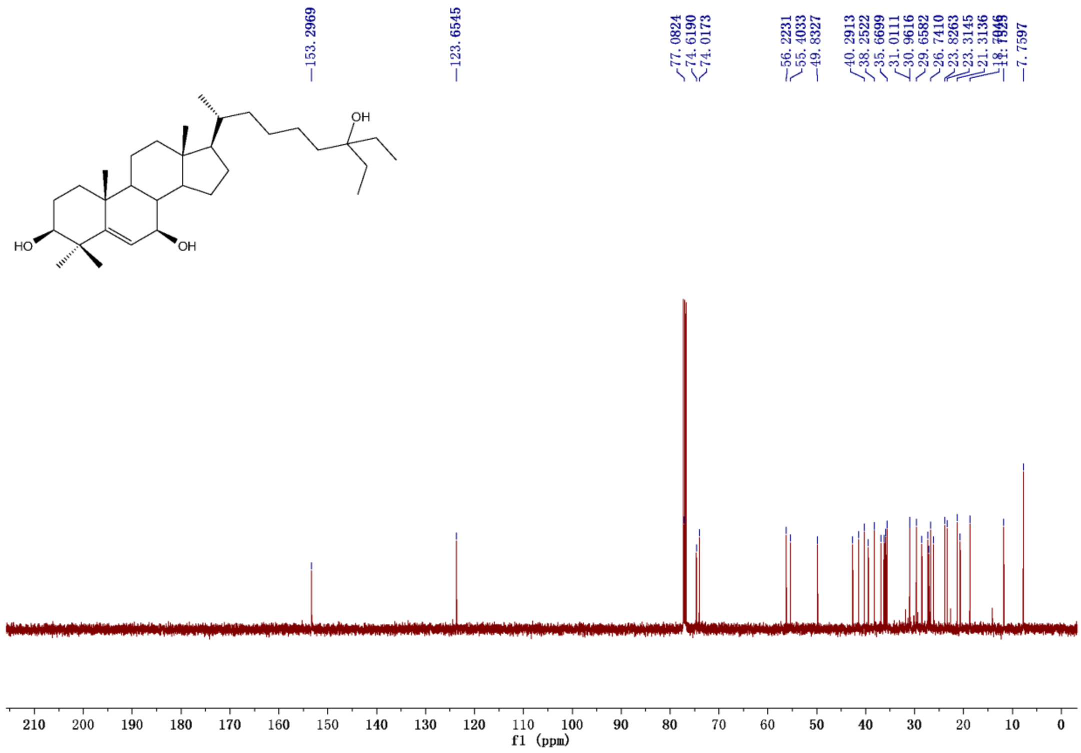

Supplementary Figure 97. Compound 101: ^{13}C-NMR (125 MHz, $CDCl_3$)

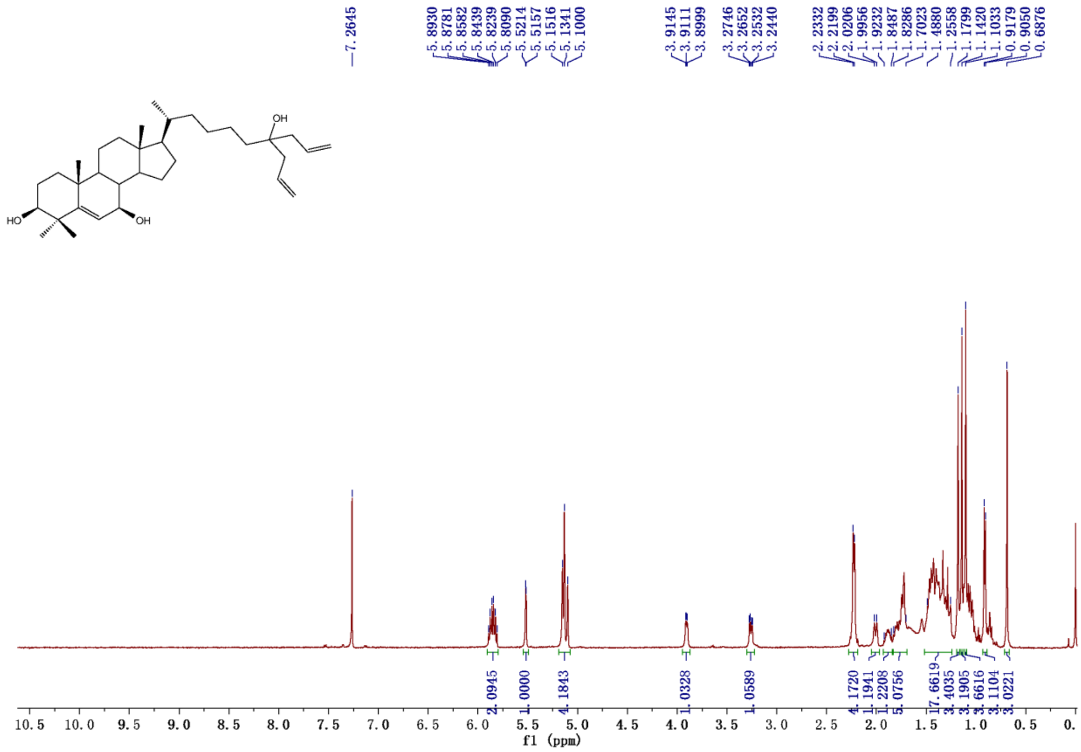

Supplementary Figure 98. Compound 102: ^{1}H-NMR (500 MHz, $CDCl_3$)

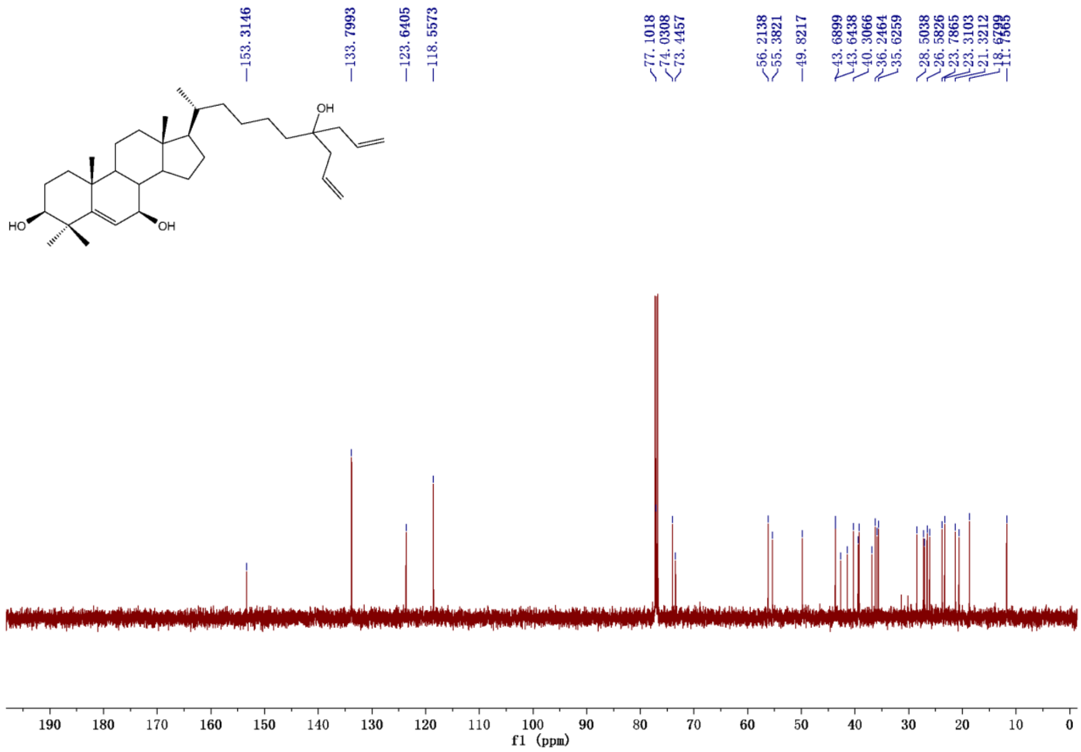

Supplementary Figure 99. Compound 102: ^{13}C-NMR (125 MHz, $CDCl_3$)

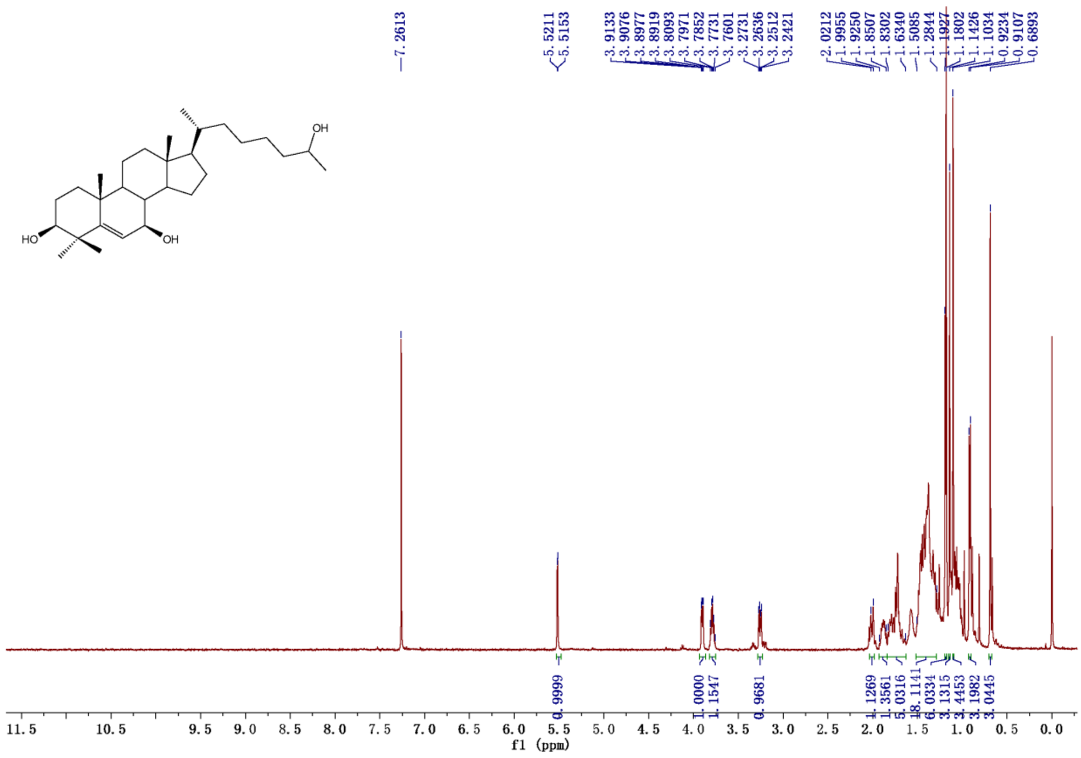

Supplementary Figure 100. Compound 107: ^{1}H-NMR (500 MHz, $CDCl_3$)

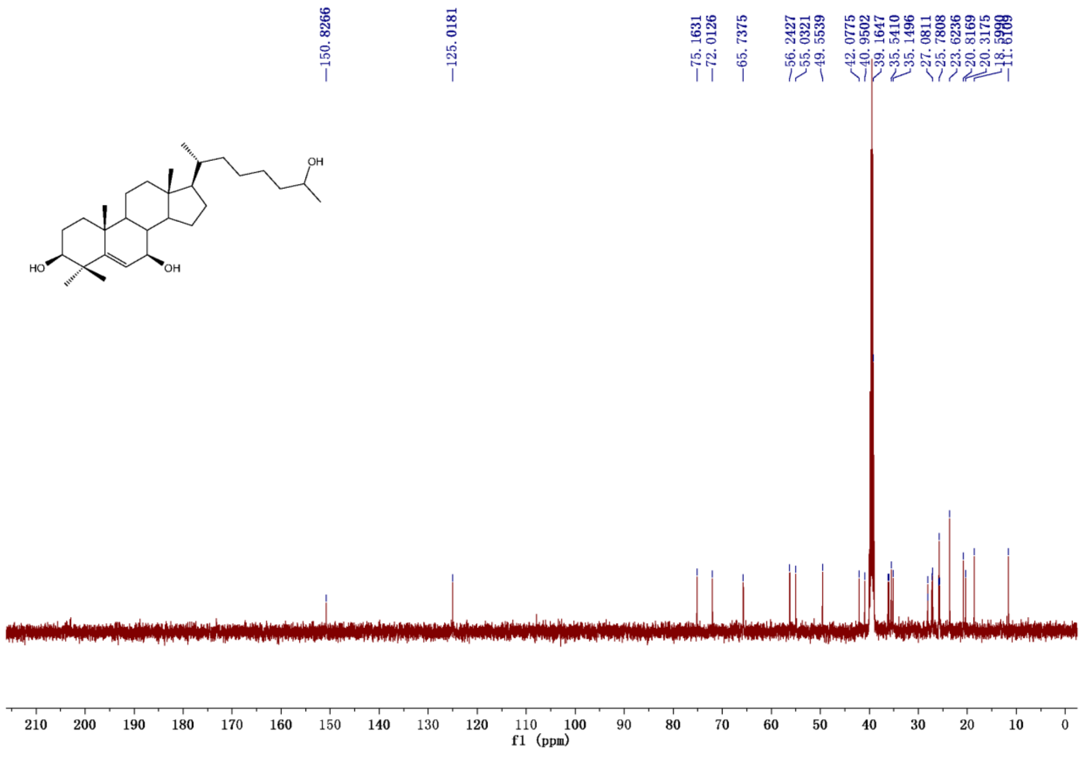

Supplementary Figure 101. Compound 107: ^{13}C-NMR (125 MHz, DMSO-d_6)

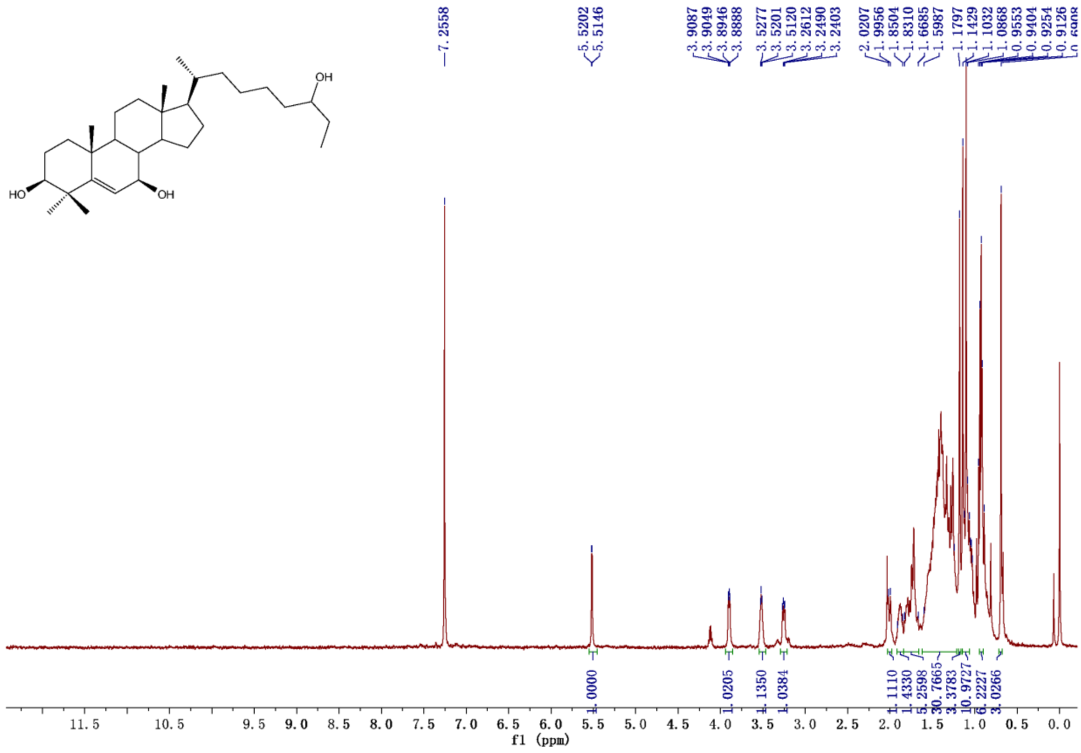

Supplementary Figure 102. Compound 108: ^{1}H-NMR (500 MHz, $CDCl_3$)

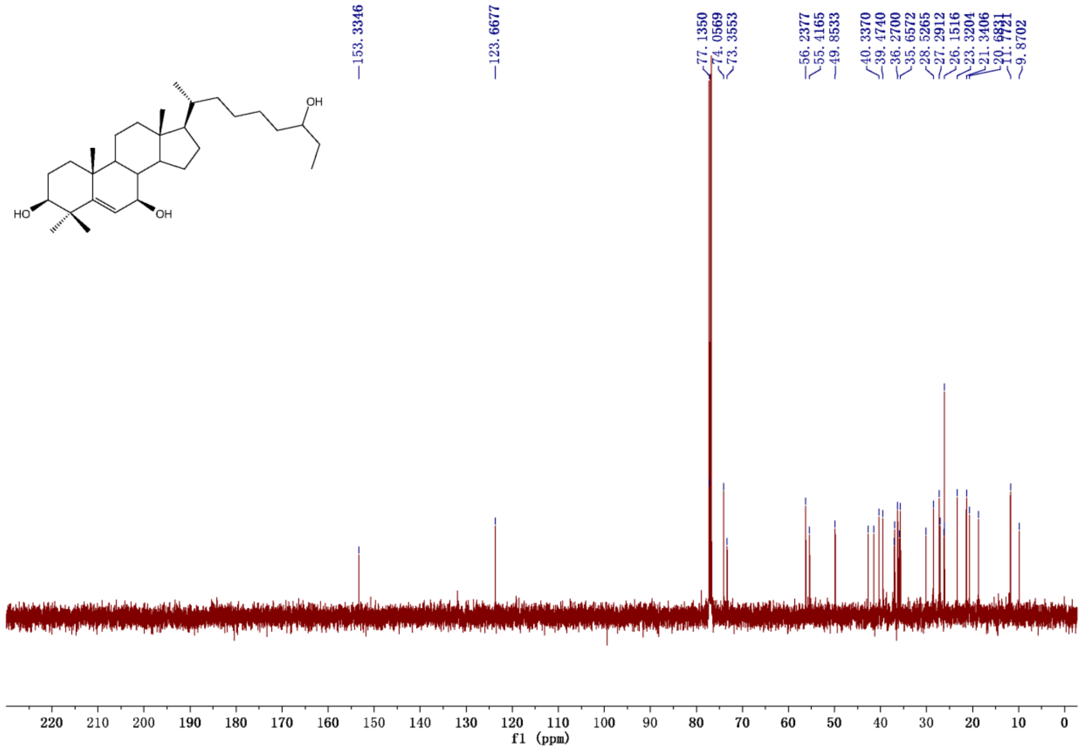

Supplementary Figure 103. Compound 108: ^{13}C-NMR (125 MHz, $CDCl_3$)

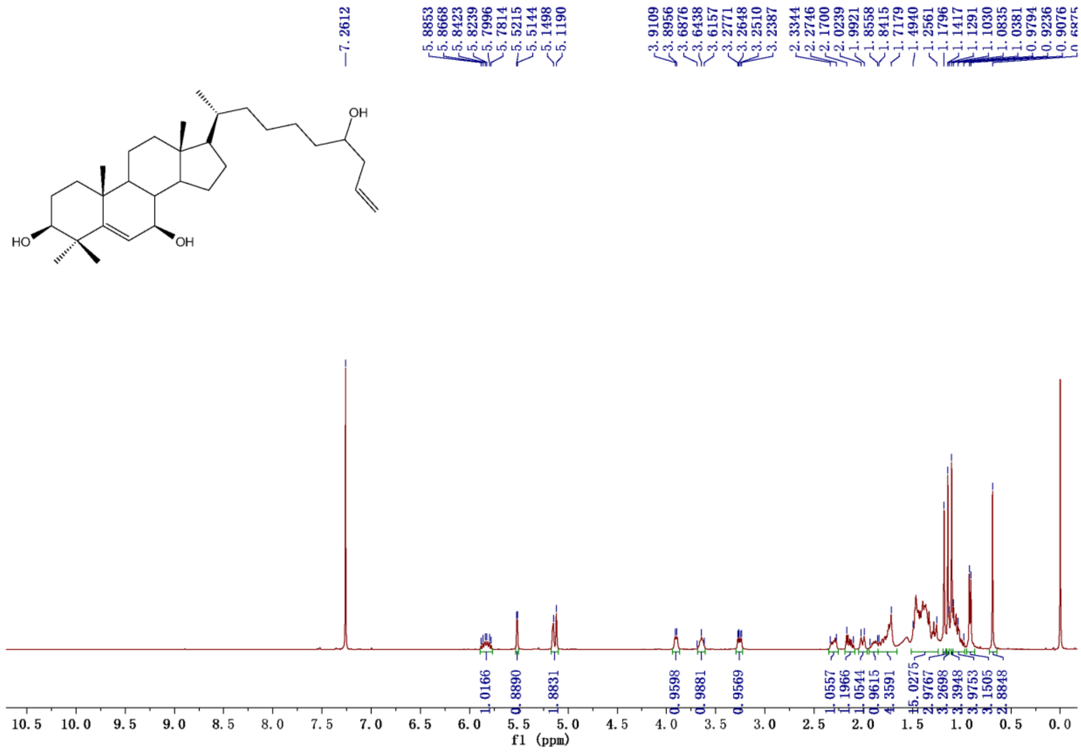

Supplementary Figure 104. Compound 109: ^{1}H-NMR (400 MHz, $CDCl_3$)

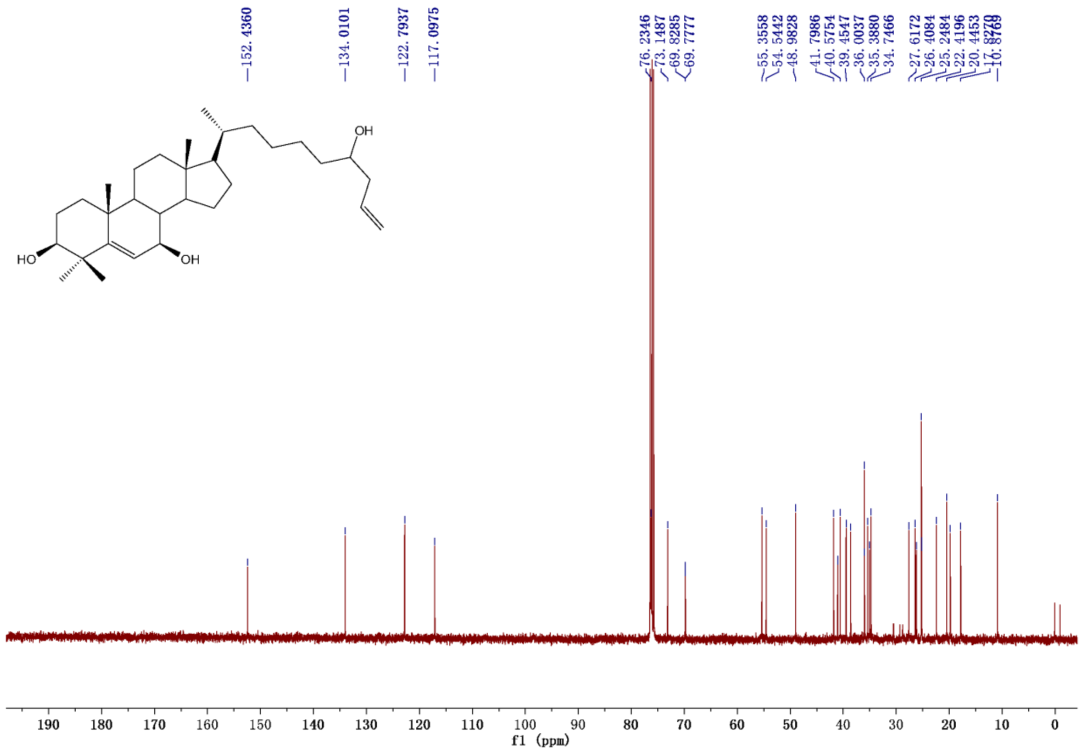

Supplementary Figure 105. Compound 109: ^{13}C-NMR (100 MHz, $CDCl_3$)

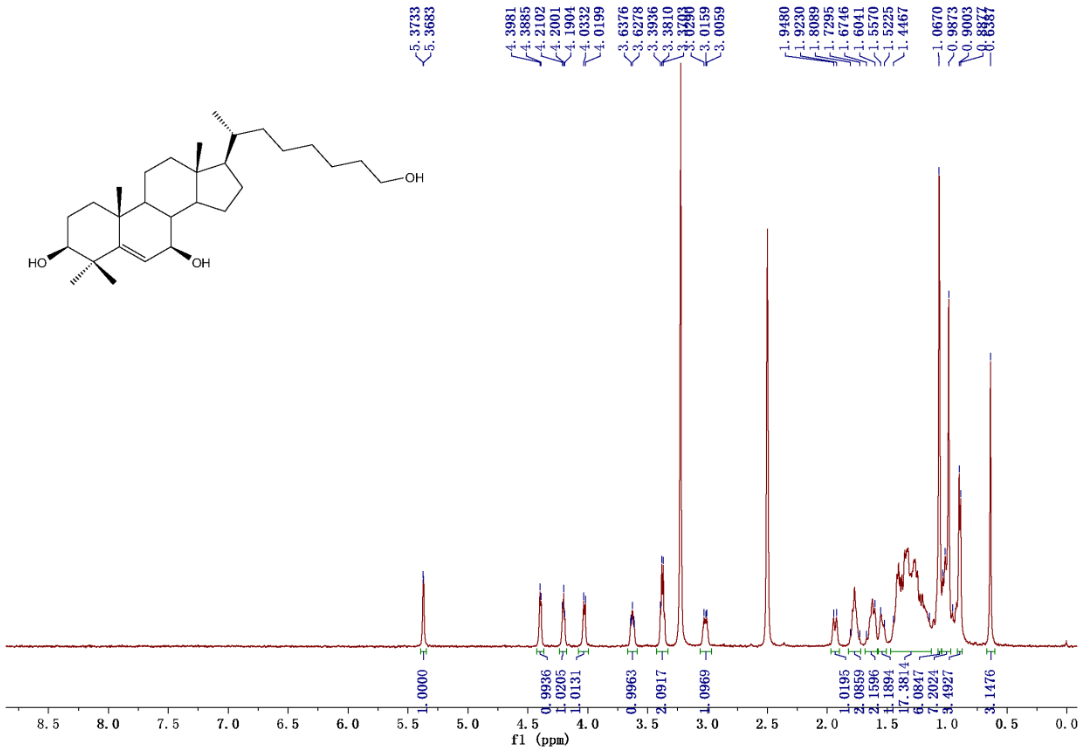

Supplementary Figure 106. Compound 113: ^{1}H-NMR (500 MHz, DMSO-d_6)

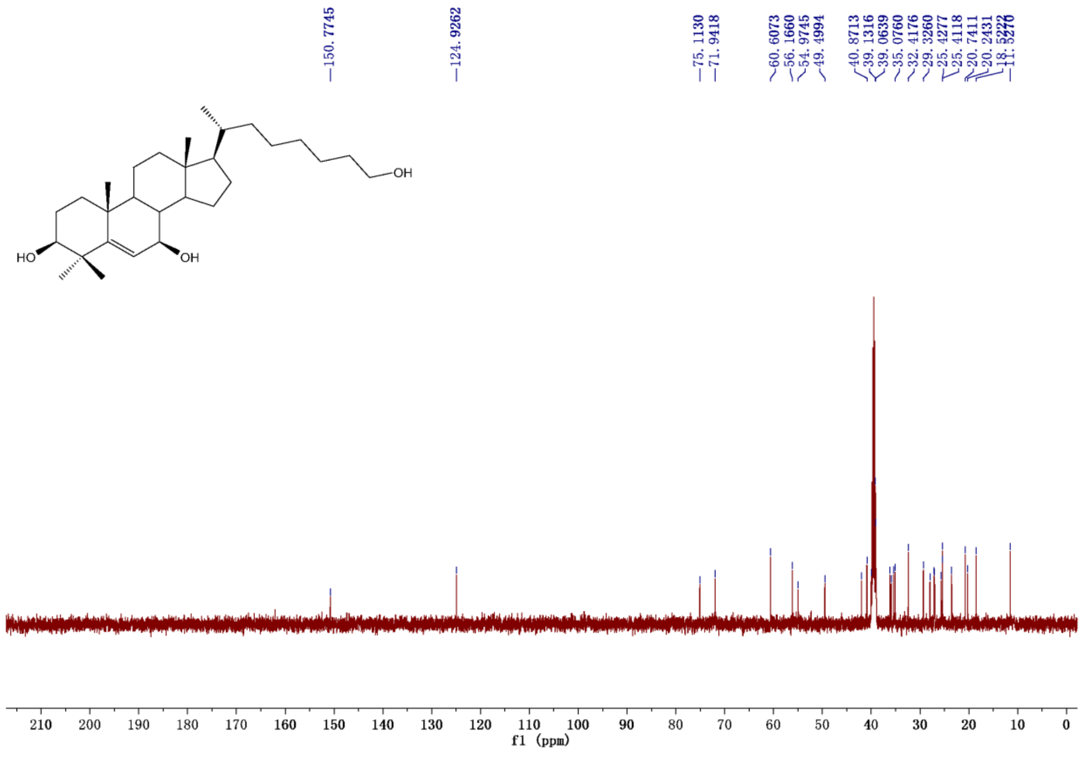

Supplementary Figure 107. Compound 113: ^{13}C-NMR (125 MHz, DMSO-d_6)

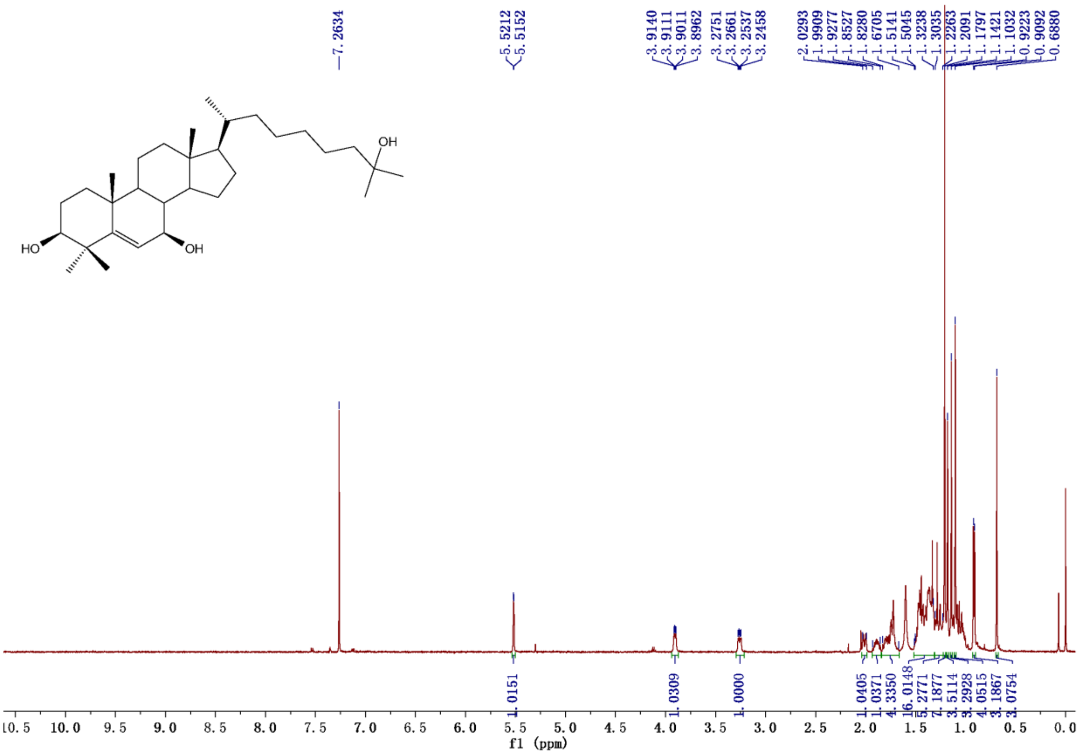

Supplementary Figure 108. Compound 117: ^{1}H-NMR (500 MHz, $CDCl_3$)

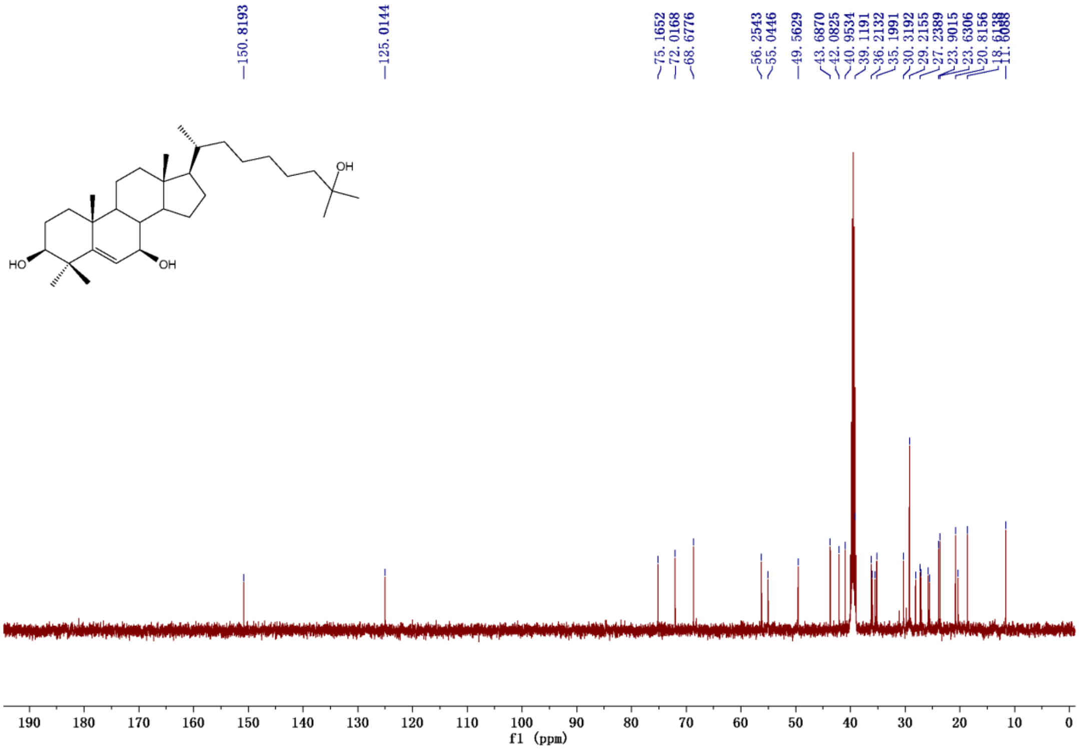

Supplementary Figure 109. Compound 117: ^{13}C-NMR (125 MHz, DMSO-d_6)

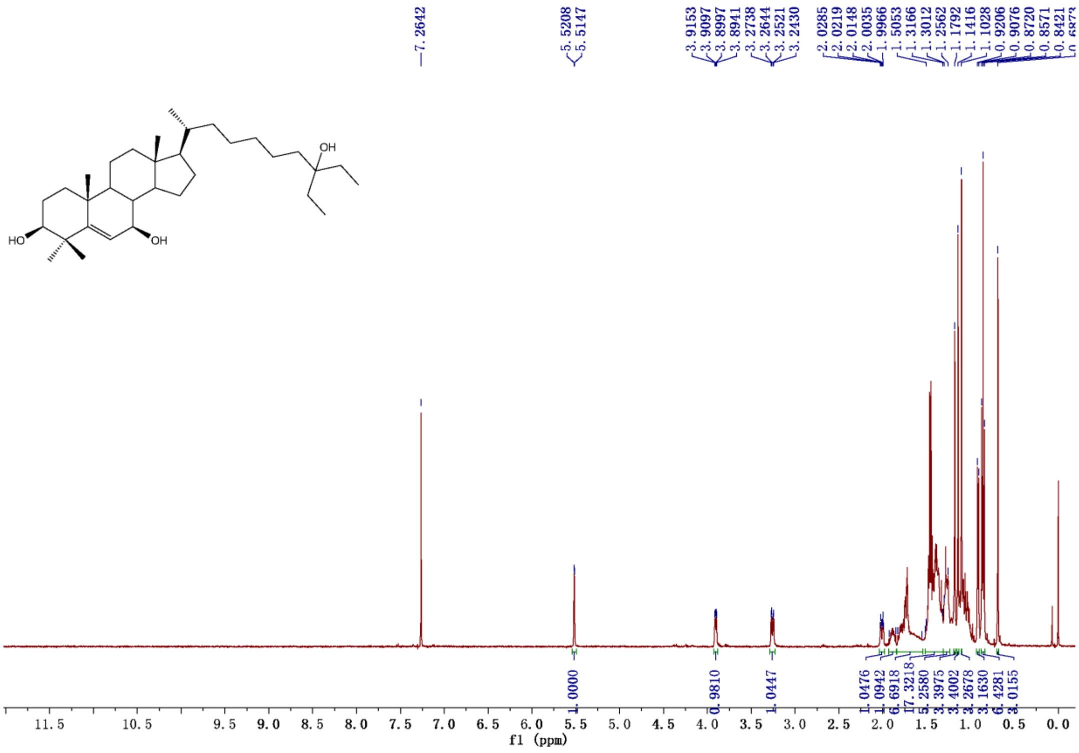

Supplementary Figure 110. Compound 118: ^{1}H-NMR (500 MHz, $CDCl_3$)

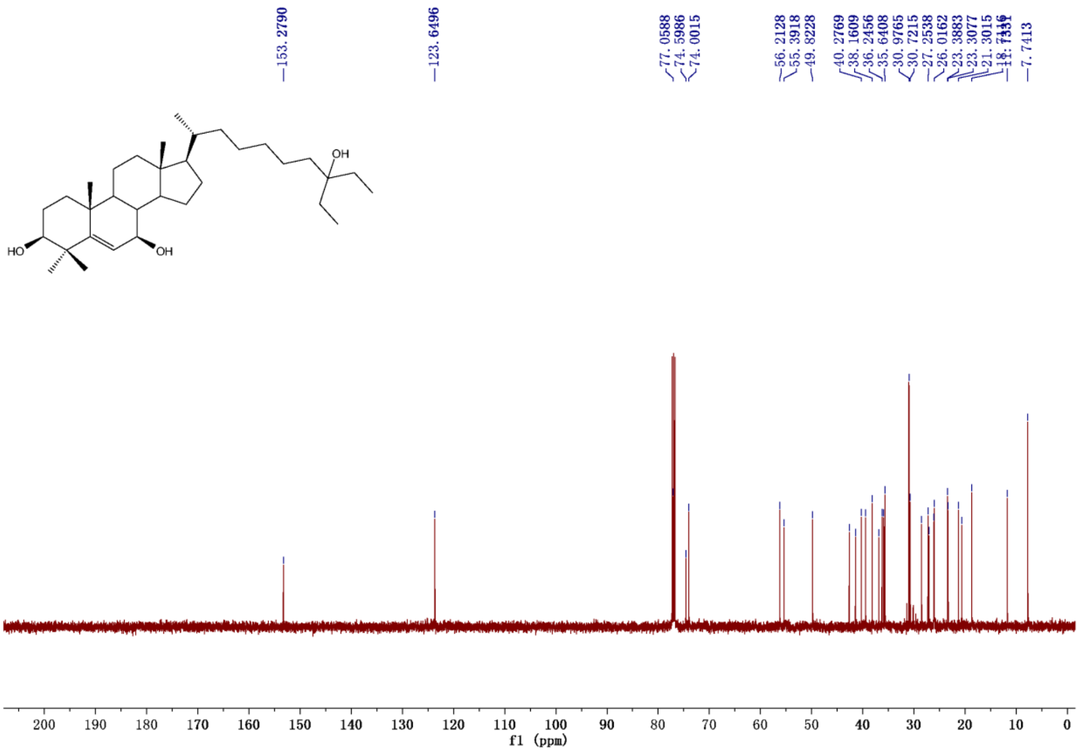

Supplementary Figure 111. Compound 118: ^{13}C-NMR (125 MHz, $CDCl_3$)

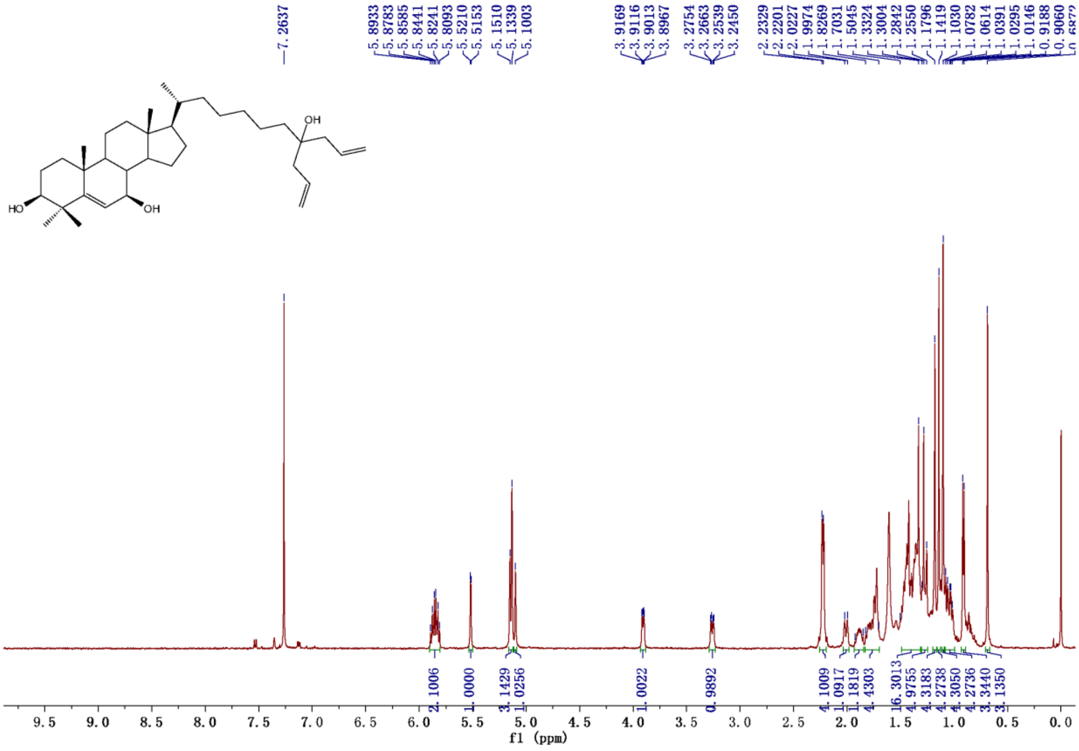

Supplementary Figure 112. Compound 119: ^{1}H-NMR (500 MHz, $CDCl_3$)

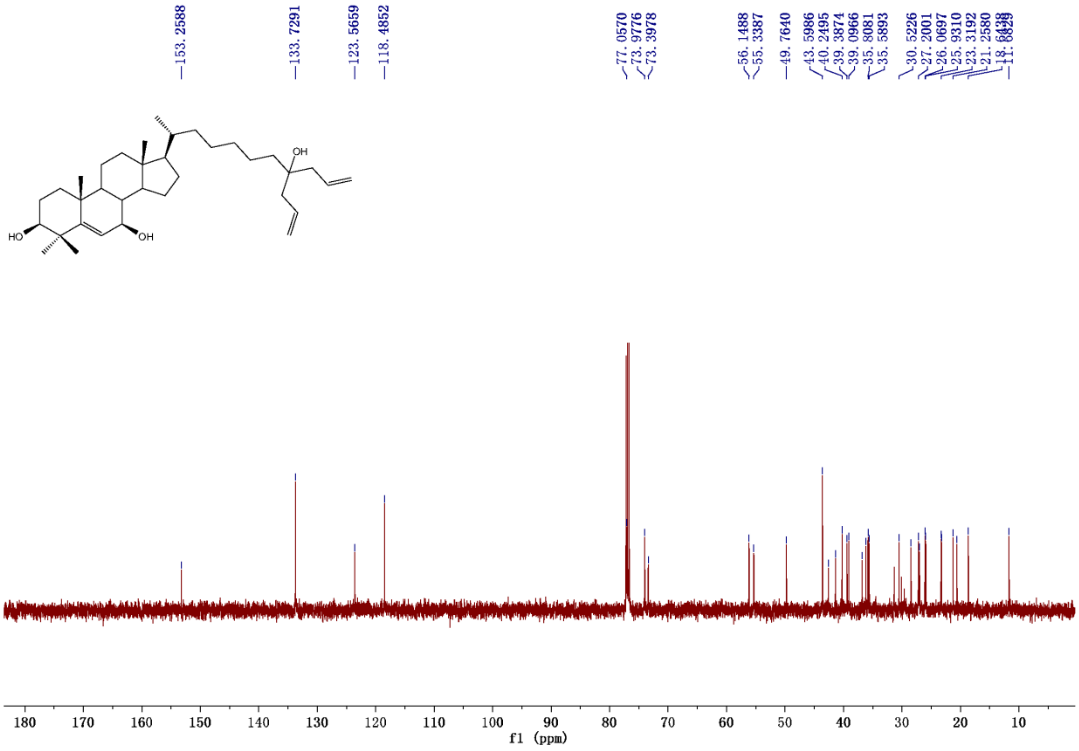

Supplementary Figure 113. Compound 119: ^{13}C-NMR (125 MHz, $CDCl_3$)

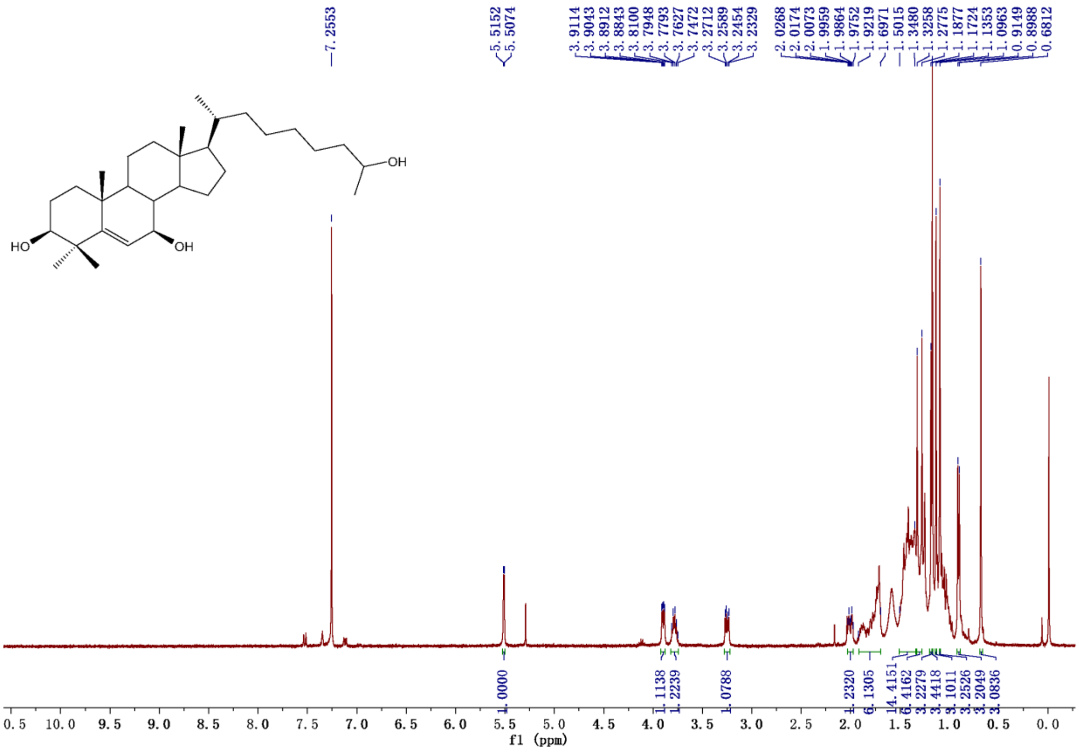

Supplementary Figure 114. Compound 124: ^{1}H-NMR (400 MHz, $CDCl_3$)

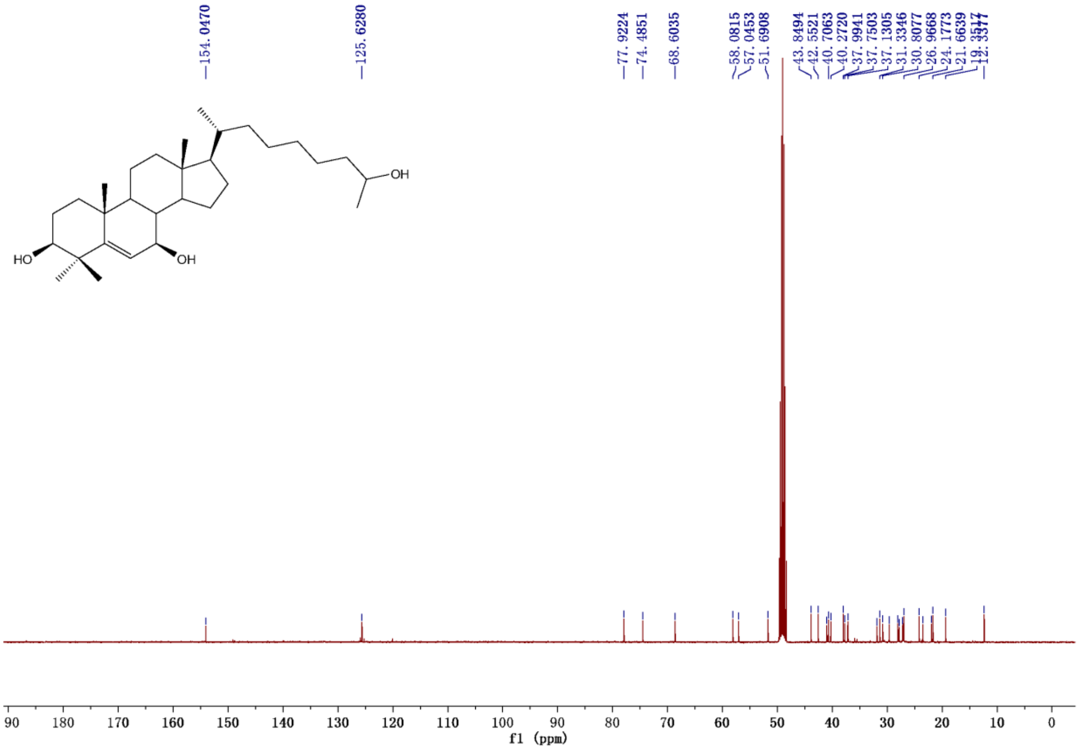

Supplementary Figure 115. Compound 124: ^{13}C-NMR (100 MHz, CD_3OD-d_4)

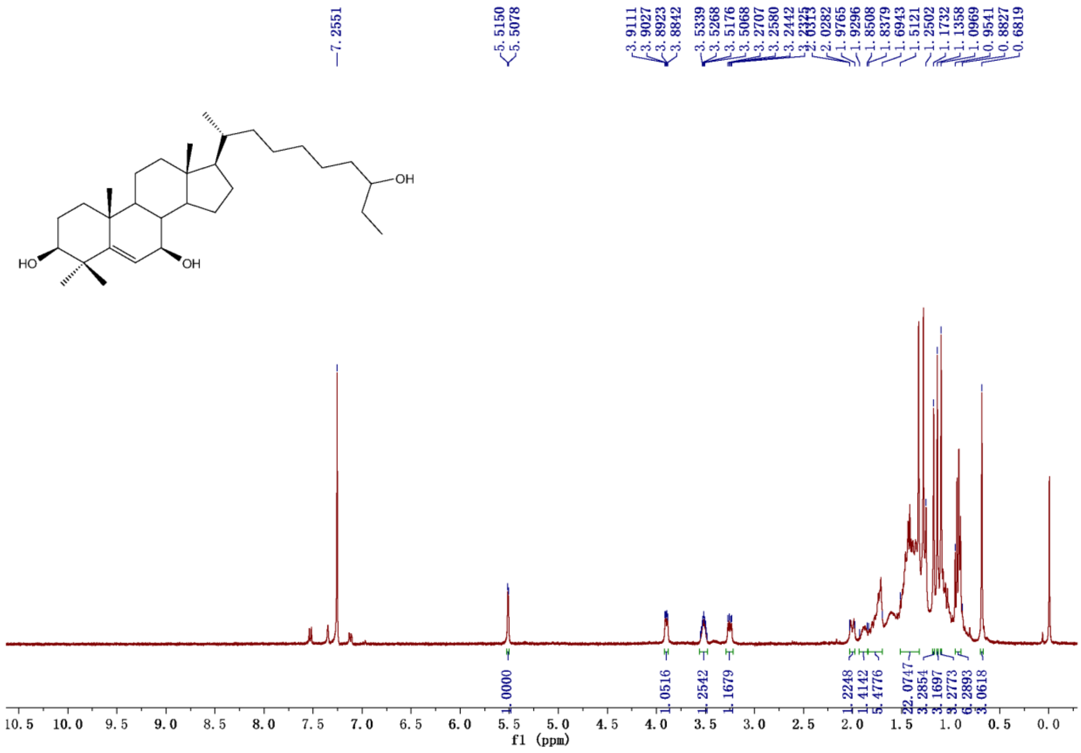

Supplementary Figure 116. Compound 125: ^{1}H-NMR (400 MHz, $CDCl_3$)

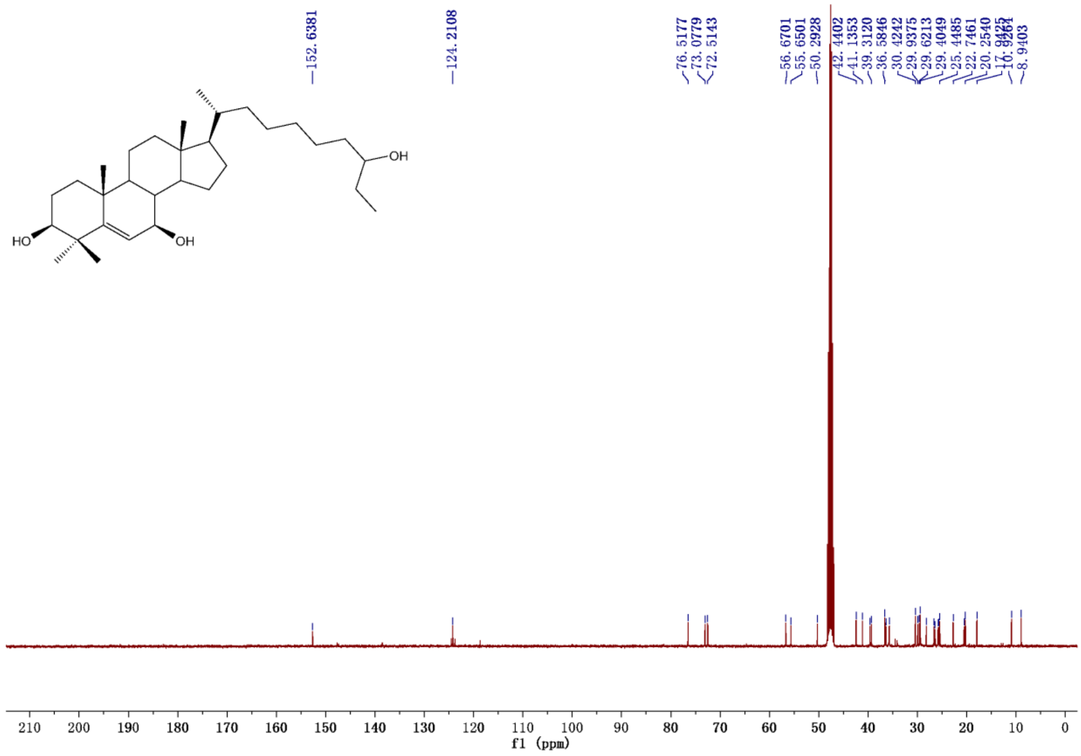

Supplementary Figure 117. Compound 125: ^{13}C-NMR (100 MHz, CD_3OD-d_4)

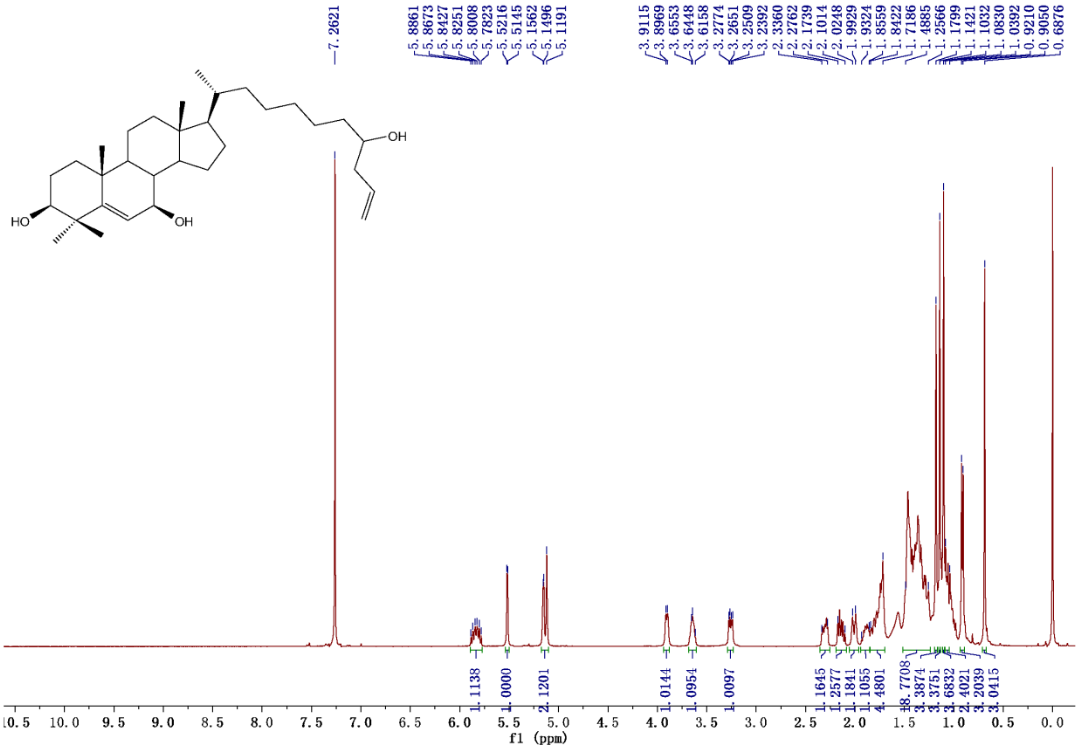

Supplementary Figure 118. Compound 126: ^{1}H-NMR (400 MHz, $CDCl_3$)

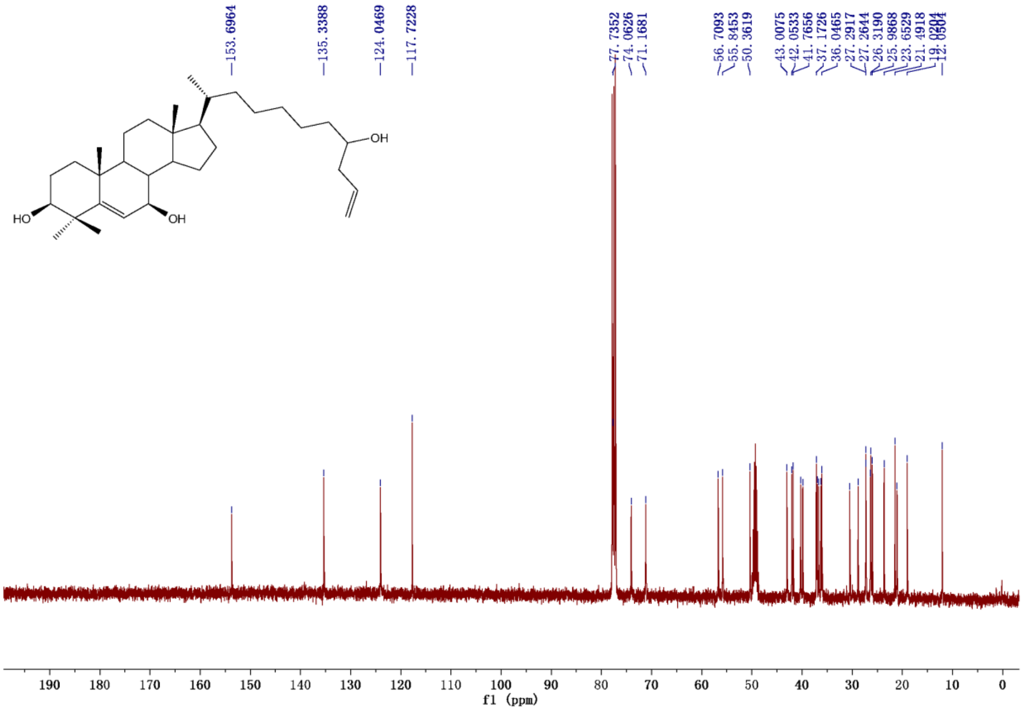

Supplementary Figure 119. Compound 126: ^{13}C-NMR (100 MHz, CD_3OD-*d_4*)

Compound 7

Chromatogram

Detector A: Ch2 205 nm

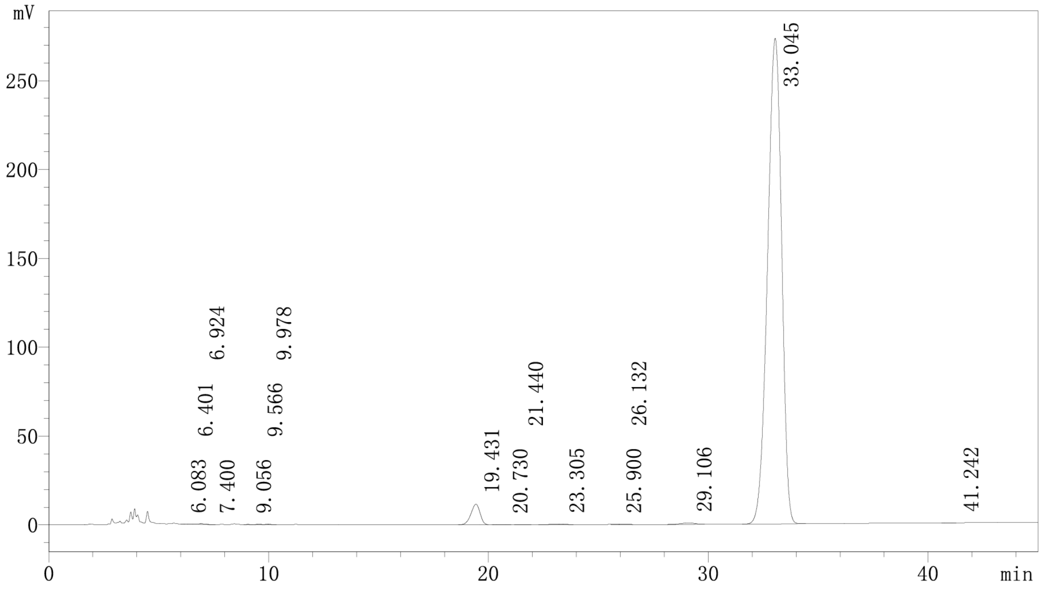

Peaks information

Detector A: Ch1 205 nm

Number	Time (min)	Area	Height	Area %	
1	6.083	479	58	0.004	
2	6.401	3275	257	0.026	
3	6.924	7730	488	0.061	
4	7.400	139	22	0.001	
5	9.056	1165	116	0.009	
6	9.566	4799	391	0.038	
7	9.978	4719	293	0.037	
8	19.431	330835	11531	2.626	
9	20.730	2045	84	0.016	
10	21.440	2513	99	0.020	
11	23.305	12536	279	0.100	
12	25.900	2014	140	0.016	
13	26.132	4157	168	0.033	
14	29.106	37047	882	0.294	
15	33.045	12183059	273361	96.708	Compound 7
16	41.242	1238	47	0.010	
Total		12597749	288215	100.000	

Supplementary Figure 120. Compound 7: HPLC (205nm)

Compound 7

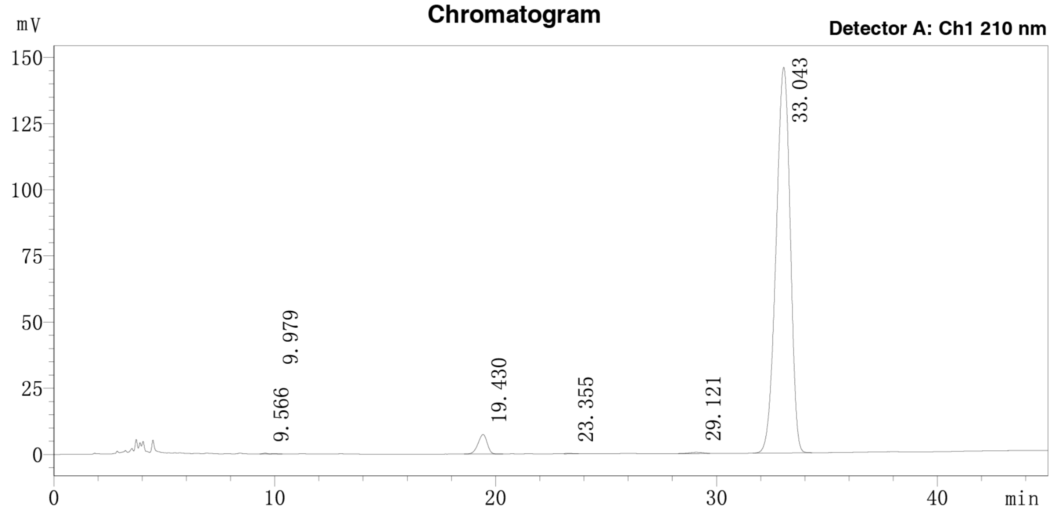

Peaks information

Detector A: Ch1 210 nm

Number	Time (min)	Area	Height	Area %	
1	9.566	3693	305	0.055	
2	9.979	1964	129	0.029	
3	19.430	211989	7375	3.148	
4	23.355	3556	141	0.053	
5	29.121	17134	420	0.254	
6	33.043	6495215	145693	96.460	Compound 7
Total		6733549	154064	100.000	

Supplementary Figure 121. Compound 7: HPLC (210nm)

Compound 35

Chromatogram

Detector A: Ch1 205 nm

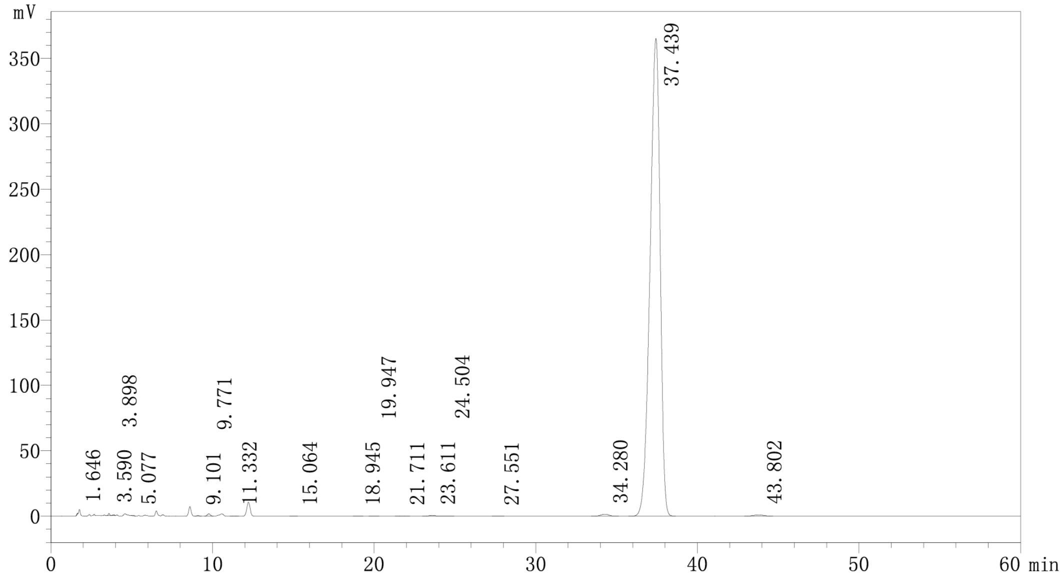

Peaks information

Detector A: Ch1 205 nm

Number	Time (min)	Area	Height	Area %	
1	1.646	3905	707	0.024	
2	3.590	11474	1792	0.071	
3	3.898	6051	734	0.038	
4	5.077	3015	320	0.019	
5	9.101	4877	450	0.030	
6	9.771	22655	1753	0.141	
7	11.332	3852	280	0.024	
8	15.064	1195	76	0.007	
9	18.945	2147	114	0.013	
10	19.947	1056	53	0.007	
11	21.711	6364	261	0.040	
12	23.611	21786	709	0.136	
13	24.504	2784	95	0.017	
14	27.551	2909	98	0.018	
15	34.280	61404	1628	0.382	
16	37.439	15856951	365315	98.741	Compound 35
17	43.802	46632	1018	0.290	
Total		16059056	375402	100.000	

Supplementary Figure 122. Compound 35: HPLC (205nm)

Compound 35

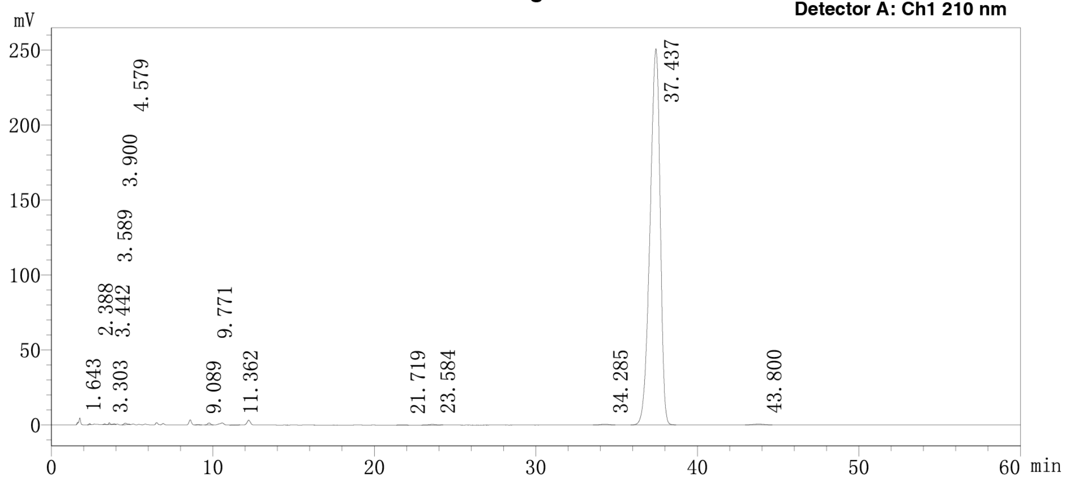

Peaks information

Detector A: Ch1 210 nm

Number	Time (min)	Area	Height	Area %	
1	1.643	3485	652	0.032	
2	2.388	5421	823	0.049	
3	3.303	3454	615	0.031	
4	3.442	154	47	0.001	
5	3.589	8457	1277	0.076	
6	3.900	4352	565	0.039	
7	4.579	15797	1052	0.143	
8	9.089	2796	245	0.025	
9	9.771	17048	1299	0.154	
10	11.362	1788	103	0.016	
11	21.719	2585	123	0.023	
12	23.584	14093	433	0.127	
13	34.285	18008	491	0.163	
14	37.437	10936094	250801	98.889	**Compound 35**
15	43.800	25390	556	0.230	
**Total**		11058920	259083	100.000	

Supplementary Figure 123. Compound 35: HPLC (210nm)

Compound 79

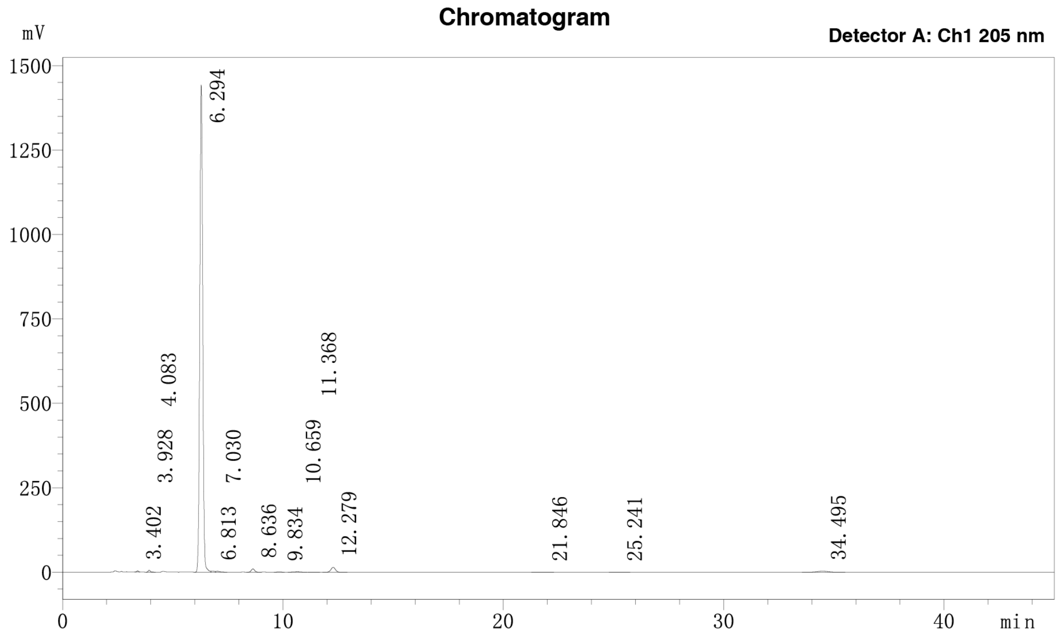

Peaks information

Detector A: Ch1 205 nm

Number	Time (min)	Area	Height	Area %	
1	3.402	20193	3658	0.144	
2	3.928	40296	6146	0.287	
3	4.083	3232	697	0.023	
4	6.294	13382565	1443726	95.269	**Compound 79**
5	6.813	36099	3593	0.257	
6	7.030	39759	2998	0.283	
7	8.636	104140	9889	0.741	
8	9.834	12256	963	0.087	
9	10.659	30389	1604	0.216	
10	11.368	5843	430	0.042	
11	12.279	215375	14764	1.533	
12	21.846	14497	597	0.103	
13	25.241	6780	232	0.048	
14	34.495	135646	3575	0.966	
**Total**		14047071	1492874	100.000	

Supplementary Figure 124. Compound 79: HPLC (205nm)

Compound 79

Chromatogram

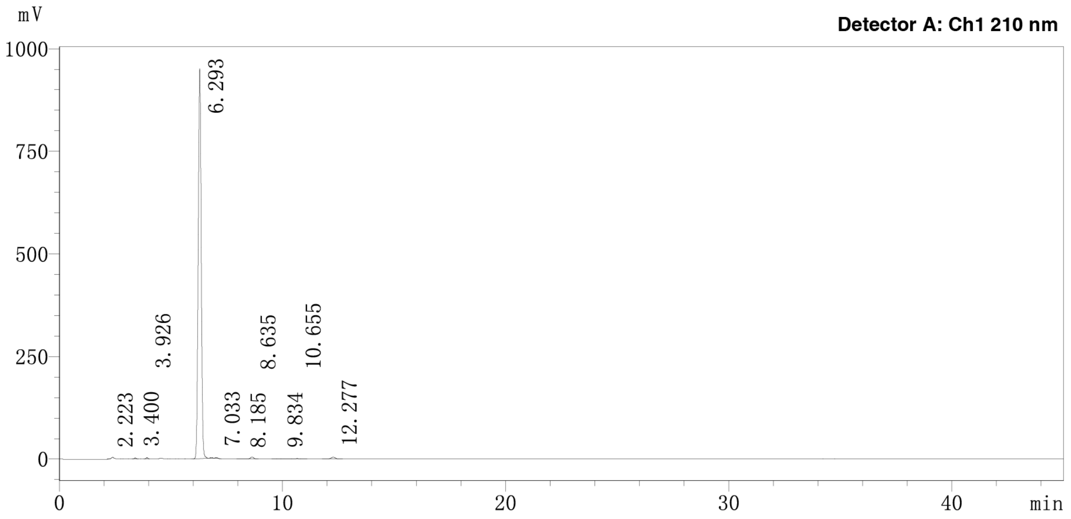

Peaks information

Detector A: Ch1 210 nm

Number	Time (min)	Area	Height	Area %	
1	2.223	3243	724	0.036	
2	3.400	13292	2449	0.146	
3	3.926	19260	3239	0.212	
4	6.293	8895612	950506	97.729	**Compound 79**
5	7.033	15167	2016	0.167	
6	8.185	3960	337	0.044	
7	8.635	50523	4656	0.555	
8	9.834	8423	716	0.093	
9	10.655	27826	1271	0.306	
10	12.277	65063	4454	0.715	
**Total**		9102370	970368	100.000	

Supplementary Figure 125. Compound 79: HPLC (210nm)

Compound 80

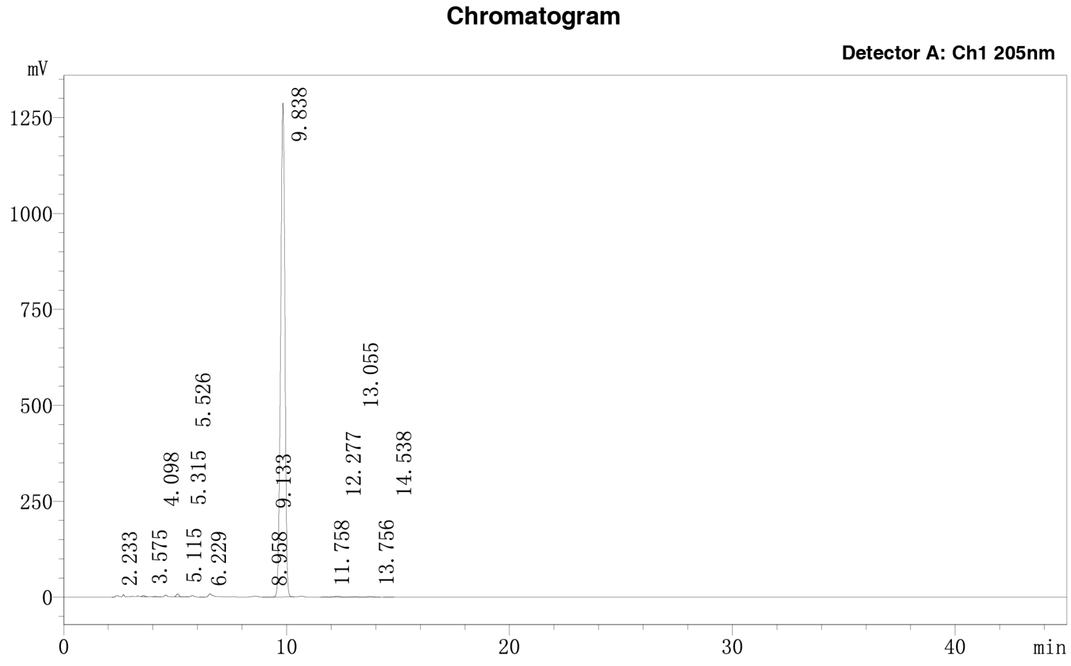

Peaks information

Detector A: Ch1 205nm

Number	Time (min)	Area	Height	Area %	
1	2.233	1703	448	0.010	
2	3.575	24891	3230	0.142	
3	4.098	7605	1002	0.043	
4	5.115	68348	7703	0.390	
5	5.315	860	204	0.005	
6	5.526	2540	372	0.015	
7	6.229	1753	210	0.010	
8	8.958	52	9	0.000	
9	9.133	78	13	0.000	
10	9.838	17309064	1287617	98.824	**Compound 80**
11	11.758	4835	342	0.028	
12	12.277	42623	2444	0.243	
13	13.055	21900	1130	0.125	
14	13.756	27661	1582	0.158	
15	14.538	1068	70	0.006	
**Total**		17514981	1306376	100.000	

Supplementary Figure 126. Compound 80: HPLC (205nm)

Compound 80

Chromatogram

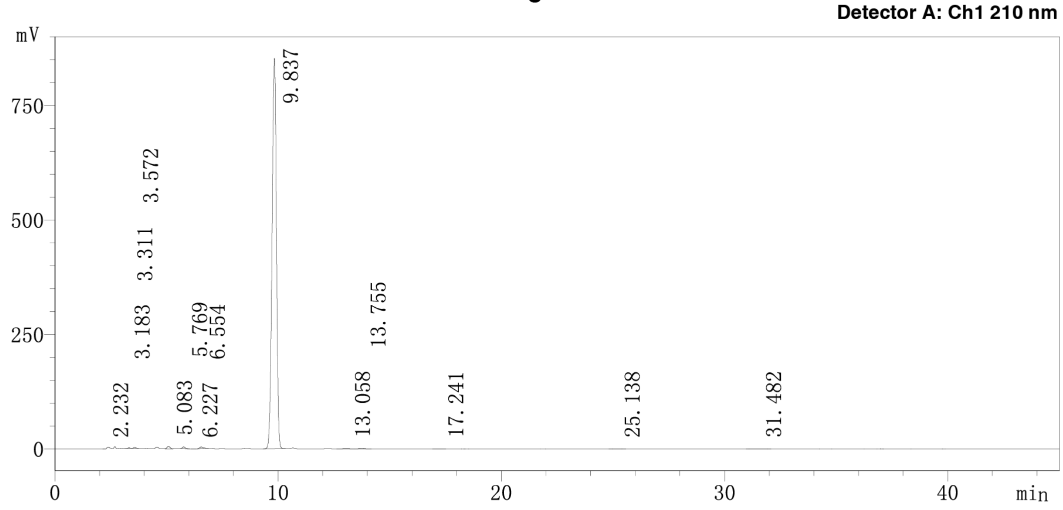

Peaks information

Detector A: Ch1 210 nm

Number	Time (min)	Area	Height	Area %	
1	2.232	1713	445	0.014	
2	3.183	83	38	0.001	
3	3.311	10475	1628	0.089	
4	3.572	19430	2358	0.164	
5	5.083	42689	4885	0.361	
6	5.769	33911	4087	0.287	
7	6.227	1104	141	0.009	
8	6.554	44283	3838	0.375	
9	9.837	11613606	852141	98.245	**Compound 80**
10	13.058	17470	1017	0.148	
11	13.755	25344	1501	0.214	
12	17.241	3996	225	0.034	
13	25.138	2135	85	0.018	
14	31.482	4800	138	0.041	
**Total**		11821038	872527	100.000	

Supplementary Figure 127. Compound 80: HPLC (210nm)

Cmpd 81

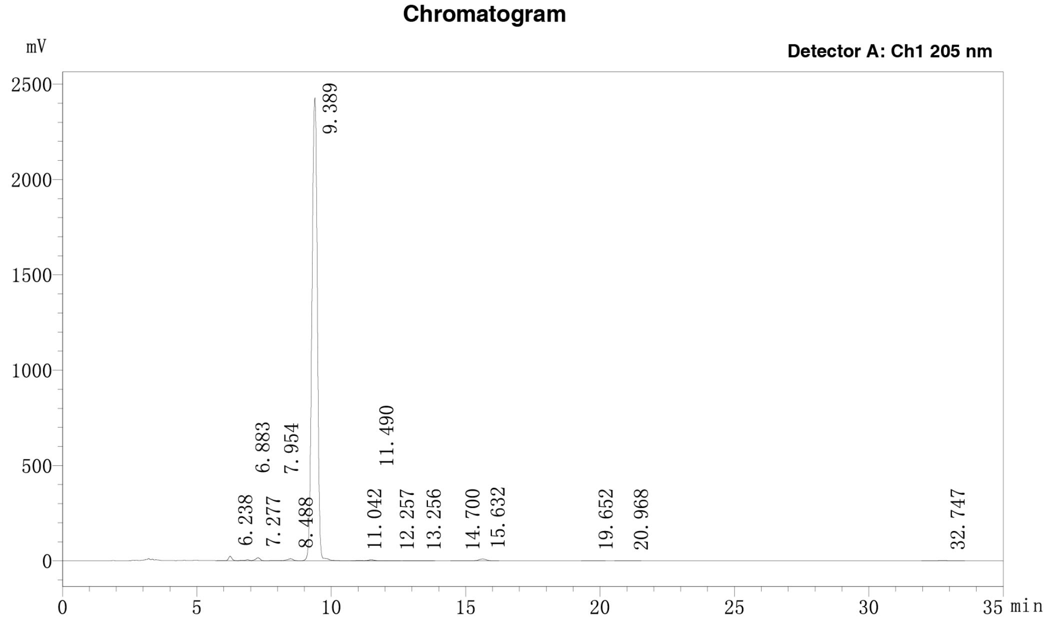

Peaks information

Detector A: Ch1 205 nm

Number	Time (min)	Area	Height	Area %	
1	6.238	214256	22102	0.609	
2	6.883	72510	4854	0.206	
3	7.277	188230	15830	0.535	
4	7.954	16882	860	0.048	
5	8.488	160800	10405	0.457	
6	9.389	34128321	2418566	97.064	Cmpd 81
7	11.042	21797	1470	0.062	
8	11.490	86315	5548	0.245	
9	12.257	5613	321	0.016	
10	13.256	10575	438	0.030	
11	14.700	4702	131	0.013	
12	15.632	191865	9642	0.546	
13	19.652	5796	279	0.016	
14	20.968	6768	270	0.019	
15	32.747	46245	1281	0.132	
Total		35160676	2491996	100.000	

Supplementary Figure 128. Compound 81: HPLC (205nm)

Cmpd 81

Chromatogram

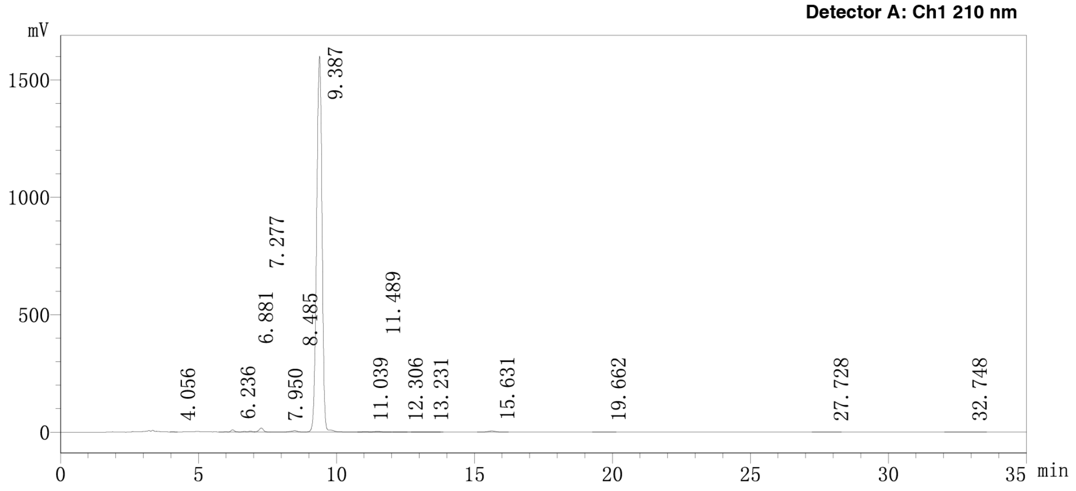

Peaks information

Detector A: Ch1 210 nm

Number	Time (min)	Area	Height	Area %	
1	4.056	4987	730	0.022	
2	6.236	87748	8829	0.390	
3	6.881	52707	3351	0.234	
4	7.277	184604	16450	0.820	
5	7.950	10556	521	0.047	
6	8.485	93075	5715	0.413	
7	9.387	21876104	1592954	97.164	Cmpd 81
8	11.039	19732	1371	0.088	
9	11.489	44716	2855	0.199	
10	12.306	3373	218	0.015	
11	13.231	6309	275	0.028	
12	15.631	90787	4635	0.403	
13	19.662	4795	228	0.021	
14	27.728	6927	236	0.031	
15	32.748	28107	790	0.125	
**Total**		22514527	1639158	100.000	

Supplementary Figure 129. Compound 81: HPLC (210nm)

Supplementary Tables

Supplementary Table 1. Nucleotide sequences of primers used for PCR to construct pCMV-HMGCR (TM1-8)-GFP.

EcoRI-HMGCR	CCGGAATTCATGTTGTCACGACTTTTCCG
HMGCR-BamHI	CGCGGATCCCGTAAAGACAGTGTGGACTCTG

Supplementary Table 2. Nucleotide sequences of primers used for Real-Time PCR in hamster-derived cells.

Genes	Sequences of forward and reverse primers
Gapdh	GCAAGTTCAAAGGCACAGTCAA
	CGCTCCTGGAAGATGGTGAT
Hmgcr	AGATACTGGAGAGTGCCGAGAAA
	TTTGTAGGCTGGGATGTGCTT
Srebp-2	TAAGCAAGTACCTGGCGGTG
	GCTTGTGATTGACCTGCTGC
Insig-1	GGCTTGTGGTGGACATTCG
	GGCGATGGTGATCCCAAGT
Hmgcs	CCTGGGTCACTTCCTTTGAATG
	GATCTCAAGGGCAACGATTCC
Ss	AATCAGACCAGTCGCAGCTT
	ACTCCAAGGAGATCGTGGGG
Fdps	ACCTACTGAACTCCGAGGCT
	TTGTAGGGAACCAAGCCACC
Ldlr	AGACACATGCGACAGGAATGAG
	GACCCACTTGCTGGCGATA

Supplementary Table 3. Nucleotide sequences of primers used for Real-Time PCR in mice.

Genes	Sequences of forward and reverse primers
Cyclophilin	TGGAGAGCACCAAGACAGACA
	TGCCGGAGTCGACAATGAT
Hmgcr	CTTGTGGAATGCCTTGTGATTG
	AGCCGAAGCAGCACATGAT
Srebp-2	GCGTTCTGGAGACCATGGA
	ACAAAGTTGCTCTGAAAACAAATCA
Insig-1	TCACAGTGACTGAGCTTCAGCA
	TCATCTTCATCACACCCAGGAC
Hmgcs	GCCGTGAACTGGGTCGAA
	GCATATATAGCAATGTCTCCTGCAA
Ss	CCAACTCAATGGGTCTGTTCCT
	TGGCTTAGCAAAGTCTTCCAACT
Fdps	ATGGAGATGGGCGAGTTCTTC
	CCGACCTTTCCCGTCACA
Ldlr	AGGCTGTGGGCTCCATAGG
	TGCGGTCCAGGGTCATCT

Supplementary Table 4. Nucleotide sequences of primers used for Real-Time PCR in human cells.

Genes	Sequences of forward and reverse primers
GAPDH	TGCACCACCAACTGCTTAGC
	GGCATGGACTGTGGTCATGAG
SREBP-1c	GCGCCTTGACAGGTGAAGTC
	GCCAGGGAAGTCACTGTCTTG
ABCA1	AATCCTGACCGGGTTGTTCCC
	CCGCCTTCACGTGCTTCTCA
ABCG8	GACAGCTTCACAGCCCACAA
	GCCTGAAGATGTCAGAGCGA

Supplementary Methods

General Information

All reagents and chemicals were purchased from commercial suppliers and used without further purification unless otherwise stated. When needed, the reactions were carried out in flame or oven-dried glassware under a positive pressure of dry N_2. Column chromatography was performed on silica gel (QinDao, 200-300 mesh) using the indicated eluents. Thin-layer chromatography was carried out on silica gel plates (QinDao) with a layer thickness of 0.25mm. Melting points were determined using the MEL-TEMP 3.0 apparatus and uncorrected. ^{1}H (400 and 500 MHz) and ^{13}C (100 and 125 MHz) NMR spectra were recorded on Bruker AM-400, JEOL-400 and Bruker AM-500 spectrometer with $CDCl_3$, DMSO-d_6 or CD_3OD-d_4 as solvent and tetramethylsilane (TMS) as the internal standard. All chemical shift values were reported in units of δ (ppm). The following abbreviations were used to indicate the peak multiplicity: *s* = singlet; *d* = doublet; *t* = triplet; *m* = multiplet; *br* = broad. High-resolution mass data were obtained on a Bruker microOTOF-Q II spectrometer. HPLC analysis was conducted for the representative compounds such as 7, 35, 79, 80 and 81 according to the following method with the retention time expressed in min at UV detection of 210 and 205 nm. For HPLC method, Shimadzu LC-20A series HPLC instrument was used, with chromatography performed on Inertsustain 250 mm × 4.6 mm, 5 μm C18 column with mobile phase gradient of 15% H_2O in CH_3CN, with a flow rate of 1.0 ml/min. All the tested compounds showed purity greater than 95% at 210 nm and 205 nm using this method.

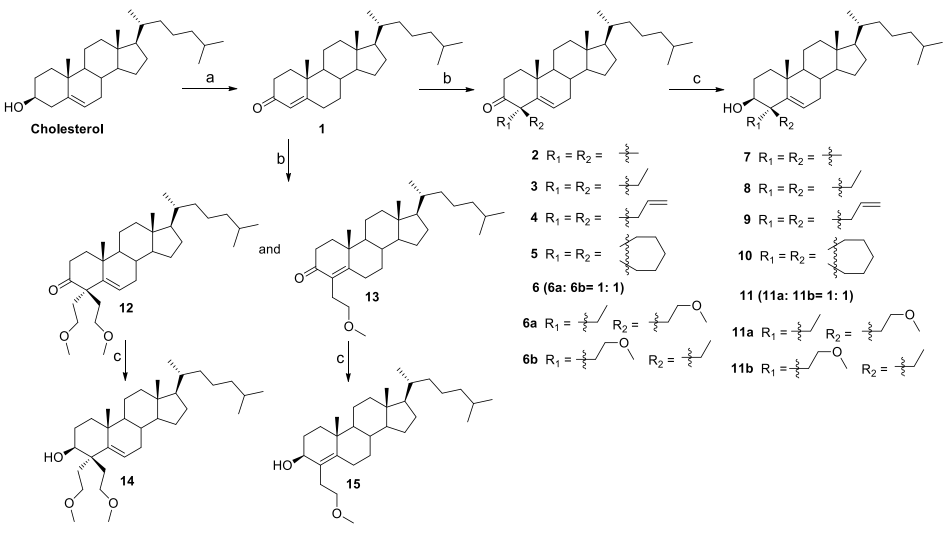

Supplementary Figure 130. The synthesis scheme of compounds 7−11, 14 and 15. Reagents and conditions: (a) Aluminium isopropoxide, cyclohexanone, toluene, reflux, 5 h, 70%; (b) t-BuOK, t-BuOH, alkyl halide, r.t., 24 h, 45% for **2**, 43% for **3**, 49% for **4**, 32% for **5**, 15% for **6** (**6a**: **6b** =1: 1), 10% for **12** and 70% for **13**; (c) $NaBH_4$, MeOH, r.t., 12 h, 90% for **7**, 89% for **8**, 85% for **9**, 84% for **10**, 87% for **11** (**11a**: **11b** =1: 1), 85% for **14** and 86% for **15**.

Synthesis of compound 1

Compound **1**: To a solution of cholesterol (10 g, 25 mmol) and cyclohexanone (60 mL) in toluene (100 mL) was added aluminium isopropoxide (10.2 g, 50 mmol) under N_2 at room temperature. The reaction mixture was heated to reflux for 5 h. After cooling to room temperature, the mixture was poured into 10% HCl (50 mL) and extracted with toluene (50 mL×3). The combined organic extract was washed with H_2O and brine, dried over anhydrous Na_2SO_4 and concentrated. The residue was purified by silica gel chromatography (petroleum ether/AcOEt, 20/1, v/v) to give compound **1** (6.73 g, 70%) as a white solid.

Characterization of compound 1

Compound **1**: ^{1}H NMR (400 MHz, $CDCl_3$) δ 5.72 (s, 1H), 2.47−2.34 (m, 3H), 2.32−2.23 (m, 1H), 2.07−1.98 (m, 2H), 1.90−1.79 (m, 2H), 1.75−1.43 (m, 7H), 1.42−1.22 (m, 5H), 1.18 (s, 3H), 1.17- 1.08 (m, 5H), 1.05–0.96 (m, 3H), 0.91 (d, J = 6.5 Hz, 3H), 0.86 (d, J = 6.6 Hz, 6H), 0.71 (s, 3H).

Procedure for the synthesis of compounds 2–6, 12 and 13

To a solution of **1** (1.15 g, 3 mmol) in dry tert-butanol (20 mL) was added potassium tert-butoxide and halide (30 mmol) under N_2 at 0 °C. The reaction mixture was stirred for 24 h at room temperature and then concentrated. The residue was poured into water and extracted with AcOEt (50 mL×3). The combined organic extract was washed with H_2O and brine, dried over anhydrous Na_2SO_4 and concentrated. The residue was purified by silica gel chromatography (petroleum ether/AcOEt, 50/1 v/v) to afford the desired product.

Characterization of compound 2

Compound **2**: white solid; yield: 45%. ^{1}H NMR (400 MHz, $CDCl_3$) δ 5.55 (dd, J = 5.0, 2.2 Hz, 1H), 2.59–2.42 (m, 2H), 2.14–1.99 (m, 3H), 1.87–1.80 (m, 1H), 1.71–1.58 (m, 3H), 1.54–1.46 (m, 3H), 1.44–1.29 (m, 5H), 1.23 (s, 6H), 1.20–0.99 (m, 9H), 0.92 (d, J = 6.5 Hz, 3H), 0.88–0.85 (m, 9H), 0.69 (s, 3H).

Characterization of compound 3

Compound **3**: white solid; yield: 43%. ^{1}H NMR (400 MHz, $CDCl_3$) δ 5.41 (d, J = 2.2 Hz, 1H), 2.51–2.30 (m, 2H), 2.21–2.09 (m, 2H), 2.03 (d, J = 12.5 Hz, 1H), 2.00 - 1.91 (m, 1H), 1.91–1.79 (m, 1H), 1.79–1.68 (m, 2H), 1.67–1.56 (m, 4H), 1.53–1.48 (m, 1H), 1.48–1.31 (m, 5H), 1.28–1.11 (m, 7H), 1.10–0.97 (m, 5H), 0.92 (d, J = 6.5 Hz, 3H), 0.87 (dd, J = 6.6, 1.4 Hz, 6H), 0.83 (s, 3H), 0.75–0.67 (m, 8H).

Characterization of compound 4

Compound **4**: white solid; yield: 49%. ^{1}H NMR (400 MHz, $CDCl_3$) δ 5.70–5.53 (m, 2H), 5.50 (dd, J = 4.5, 2.3 Hz, 1H), 5.01 (d, J = 8.6 Hz, 2H), 4.94 (d, J = 10.3 Hz, 2H), 2.88–2.76 (m, 1H), 2.51–2.34 (m, 3H), 2.34–2.23 (m, 1H), 2.21–2.11 (m, 1H), 2.11–2.00 (m, 2H), 1.97–1.89 (m, 1H), 1.89–1.79 (m, 1H), 1.72–1.62 (m, 2H), 1.58–1.48 (m, 3H), 1.44–1.23 (m, 6H), 1.21–0.99 (m, 9H), 0.92 (d, J = 6.5 Hz, 3H), 0.87 (dd, J = 6.6, 1.4 Hz, 6H), 0.80 (s, 3H), 0.69 (s, 3H).

Characterization of compound 5

Compound **5**: White solid; yield: 32%. ^{1}H NMR (400 MHz, $CDCl_3$) δ 5.59 (d, J = 2.3 Hz, 1H), 2.56–2.47 (m, 1H), 2.44–2.35 (m, 1H), 2.19–1.92 (m, 8H), 1.55 (s, 6H), 1.36 (d, J = 7.1 Hz, 8H), 1.17–1.07 (m, 9H), 0.91 (d, J = 6.1 Hz, 4H), 0.86 (dd, J = 8.2, 4.1 Hz, 12H), 0.67 (s, 3H).

Characterization of compound 6

Compound **6**: colorless oil; yield: 15%. ^{1}H NMR (400 MHz, $CDCl_3$) δ 5.52 (dd, J = 4.0, 2.1 Hz,1H), 5.43 (dd, J = 4.0, 2.4 Hz, 1H), 3.35–3.29 (m, 2H), 3.28 (s, 3H), 3.26–3.20 (m, 1H), 3.22 (s, 3H), 3.20–3.10 (m, 1H), 2.55–2.32 (m, 4H), 2.22–2.00 (m, 4H), 2.00–1.80 (m, 6H), 1.79–1.65 (m,4H), 1.63–1.45 (m, 10H), 1.45–1.22 (m, 12H), 1.22–0.95 (m, 20H), 0.92 (d, J = 6.4 Hz, 6H), 0.87 (d, J = 7.7 Hz, 18H), 0.76–0.71 (s, 6H), 0.70 (s, 6H). ^{13}C NMR (100 MHz, $CDCl_3$) δ 214.48, 213.96, 145.97, 145.12, 121.49, 121.26, 69.63, 69.07, 58.45, 58.40, 56.96, 56.93, 56.18 (2C), 55.45, 55.11, 49.11, 48.96, 42.78, 42.39 (2C), 39.80, 39.51 (2C), 37.07, 36.43, 36.21, 36.19, 35.79 (2C), 34.88 (2C), 34.57, 34.47, 31.79, 31.69, 31.61, 31.52, 31.17 (2C), 29.86, 29.70, 28.28 (2C), 28.01 (2C), 24.17 (2C), 23.86 (2C), 22.81 (2C), 22.55 (2C), 21.38 (2C), 19.44, 19.32, 18.70 (2C), 11.95 (2C), 9.68, 9.28. HRMS (ESI): calcd for $C_{32}H_{54}NaO_2$ $[M+Na]^+$, 493.4016, found 493.4035.

Characterization of compound 12

Compound **12**: white solid; yield: 10%. ^{1}H NMR (400 MHz, $CDCl_3$) δ 5.55–5.50 (m, 1H), 3.25 (s, 3H), 3.33–3.23 (m, 2H), 3.20 (s, 3H), 3.22–3.13 (m, 2H), 2.56–2.38 (m, 3H), 2.21–2.11 (m, 1H), 2.07–1.99 (m, 1H), 1.99–1.89 (m, 3H), 1.89–1.79 (m, 1H), 1.62–1.49 (m, 6H), 1.43–1.22 (m, 8H), 1.20 - 0.99 (m, 10H), 0.91 (d, J = 6.4 Hz, 3H), 0.86 (d, J = 8.5 Hz, 6H), 0.69 (s, 3H).

Characterization of compound 13

Compound **13**: Colorless oil; yield: 70%. ^{1}H NMR (400 MHz, $CDCl_3$) δ 3.31 (s, 3H), 3.30 –3.18 (m, 2H), 2.82–2.74 (m, 1H), 2.62 (t, J = 7.5 Hz, 2H), 2.46–2.31 (m, 2H), 2.17–1.92 (m, 3H), 1.91–1.79 (m, 2H), 1.74–1.48 (m, 6H), 1.43–1.23 (m, 6H), 1.17 (s, 3H), 1.16–1.07 (m, 5H), 1.04–0.93 (m, 3H), 0.91 (d, J = 6.5 Hz, 3H), 0.86 (dd, J = 6.6, 1.5 Hz, 6H), 0.71 (s, 3H).

Procedure for the synthesis of compounds 7–11, 14 and 15

One of compounds **2–6**, **12** and **13** (1 mmol) was dissolved in MeOH (20 mL) and $NaBH_4$ (75.66 mg, 2 mmol) was added in portions. The reaction mixture was stirred for 6 h at room temperature and then concentrated. The residue was poured into H_2O (20 mL) and extracted with AcOEt (30 mL×3). The combined organic extract was washed with brine, dried over anhydrous Na_2SO_4 and concentrated. The residue was purified by silica gel chromatography (petroleum ether/AcOEt, 25/1, v/v) to afford the desired product.

Characterization of compound 7

Compound **7**: white solid; yield: 90%. ^{1}H NMR (400 MHz, $CDCl_3$) δ 5.56 (dd, J = 4.0, 3.1 Hz, 1H), 3.29–3.19 (m, 1H), 2.09 (dt, J = 18.1, 5.3 Hz, 1H), 2.01 (dt, J = 6.2, 2.9 Hz, 1H), 1.89–1.78 (m, 1H), 1.78–1.65 (m, 3H), 1.65–1.43 (m, 6H), 1.42–1.30 (m, 5H), 1.29–1.24 (m, 2H), 1.19–1.10 (m, 8H), 1.07 (d, J = 4.7 Hz, 8H), 0.91 (d, J = 6.5 Hz, 3H), 0.86 (dd, J = 6.6, 1.5 Hz, 6H), 0.67 (s, 3H). ^{13}C NMR (100 MHz, $CDCl_3$) δ 149.79, 120.19, 77.56, 57.30, 56.09, 50.95, 42.23, 41.61, 39.78, 39.53, 36.76, 36.66, 36.21, 35.81, 32.61, 30.88, 28.30, 28.02, 27.46, 27.2, 24.20, 23.86, 23.63, 22.83, 22.57, 21.33, 20.60, 18.72, 11.88. HRMS (ESI): calcd for $C_{29}H_{50}NaO$ $[M+Na]^+$, 437.3754, found 437.3728.

Characterization of compound 8

Compound **8**: white solid; yield: 89%. ^{1}H NMR (400 MHz, $CDCl_3$) δ 5.23 (t, J = 3.6 Hz, 1H), 3.63 (dd, J = 11.7, 3.4 Hz, 1H), 2.21–2.11 (m, 1H), 2.04–1.97 (m, 1H), 1.92 –1.76 (m, 4H), 1.69 (d, J = 39.2 Hz, 5H), 1.54–1.44 (m, 3H), 1.40–1.24 (m, 9H), 1.17–1.05 (m, 7H), 1.01 (s, 3H), 0.91 (d, J = 6.5 Hz, 3H), 0.86 (dd, J = 6.6, 1.7 Hz, 6H), 0.78 (t, J = 7.2 Hz, 3H), 0.69 (t, J = 7.4 Hz, 3H), 0.67 (s, 3H). ^{13}C NMR (100 MHz, $CDCl_3$) δ 139.52, 124.16, 72.55, 57.32, 56.13, 50.76, 47.93, 42.26, 39.83, 39.53, 36.34, 36.28, 36.22, 35.83, 32.32, 30.88, 28.33, 28.02, 26.33, 25.37, 25.20, 24.16, 23.89, 22.84, 22.58, 20.75, 20.66, 18.71, 11.92, 8.01, 7.97. HRMS (ESI): calcd for $C_{31}H_{54}NaO$ $[M+Na]^+$, 465.4067, found 465.4080.

Characterization of compound 9

Compound **9**: white solid; yield: 85%. ^{1}H NMR (400 MHz, $CDCl_3$) δ 5.88–5.78 (m, 1H), 5.72–5.62 (m, 1H), 5.36 (t, J = 3.5 Hz, 1H), 5.11 - 4.99 (m, 4H), 3.63 - 3.59 (m, 1H), 2.53–2.50 (m, 2H), 2.25–2.13 (m, 3H), 2.02–1.99 (m, 1H), 1.90–1.79 (m, 2H), 1.72–1.60 (m, 3H), 1.57–1.45 (m, 6H), 1.40–1.22 (m, 6H), 1.18–1.11 (m, 5H), 1.08 (s, 3H), 1.04–0.95 (m, 3H), 0.91 (d, J = 6.5 Hz, 3H), 0.87 (dd, J = 6.6, 1.6 Hz, 6H), 0.67 (s, 3H). ^{13}C NMR (100 MHz, $CDCl_3$) δ 141.09, 136.56, 135.87, 124.51, 116.85, 116.71, 73.30, 57.28, 56.12, 50.76, 47.56, 42.25, 39.78, 39.53, 39.00, 37.14, 36.44, 36.17 (2C), 35.83, 32.29, 30.82, 28.33, 28.01, 26.33, 24.15, 23.90, 22.86, 22.60, 21.07, 20.63, 18.72, 11.92. HRMS (ESI): calcd for $C_{33}H_{54}NaO$ $[M+Na]^+$, 489.4067, found 489.4072.

Characterization of compound 10

Compound **10**: white solid; yield: 84%. ^{1}H NMR (400 MHz, $CDCl_3$) δ 5.73 (dd, J = 4.1, 2.7 Hz, 1H), 3.21–3.11 (m, 1H), 2.26–2.16 (m, 1H), 2.05–1.92 (m, 2H), 1.90–1.79 (m, 3H), 1.78–1.69 (m, 2H), 1.68–1.56 (m, 6H), 1.55–1.19 (m, 17H), 1.19 –1.10 (m, 5H), 1.08 (s, 3H), 0.91 (d, J = 6.4 Hz, 3H), 0.87 (dd, J = 6.6, 1.4 Hz, 6H), 0.67 (s, 3H). ^{13}C NMR (100 MHz, $CDCl_3$) δ 145.51, 124.58, 77.44, 57.29, 56.04, 52.15, 45.63, 42.12, 39.74, 39.53, 37.28, 37.21, 36.21, 35.82, 33.34, 32.47, 30.88, 28.34, 28.04, 28.00, 27.05, 26.77, 24.28, 24.13, 23.86, 22.86, 22.59, 21.33, 21.18, 20.50, 18.73, 11.83. HRMS (ESI): calcd for $C_{32}H_{54}NaO$ $[M+Na]^+$, 477.4067, found 477.4068.

Characterization of compound 11

Compound **11**: white solid; yield: 87%. ^{1}H NMR (400 MHz, $CDCl_3$) δ 5.29 (s, 1H), 5.22 (s, 1H), 3.55–3.34 (m, 5H), 3.32 (s, 3H), 3.30 (s, 3H), 3.28–3.22 (m, 1H), 2.20–2.07 (m, 3H), 2.06–1.96 (m, 3H), 1.93–1.78 (m, 6H), 1.74–1.59 (m, 12H), 1.56 –1.51 (m, 3H), 1.49–1.44 (m, 3H), 1.42–1.30 (m, 9H), 1.29–1.22 (m, 4H), 1.20–1.09 (m, 11H), 1.08 (s, 6H), 1.03 (s, 3H), 1.01–0.93 (m, 6H), 0.91 (d, J = 6.2 Hz, 6H), 0.87 (d, J = 6.4 Hz, 12H), 0.79 (t, J = 7.0 Hz, 3H), 0.67 (s, 6H). ^{13}C NMR (100 MHz, $CDCl_3$) δ 142.23, 141.90, 123.34, 123.18, 73.88, 71.93, 69.96, 69.83, 58.63, 58.55, 57.33, 57.30, 56.13, 56.12, 51.23, 50.68, 48.35, 47.22, 42.28, 42.24, 39.81, 39.79, 39.52 (2C), 37.19, 36.56, 36.38, 36.33, 36.21 (2C), 35.82 (2C), 34.63, 33.54, 32.55, 32.23, 30.85 (2C), 28.31 (2C), 28.02 (2C), 26.63, 25.84, 25.15, 24.60, 24.17, 24.15,

23.88 (2C), 22.83 (2C), 22.56 (2C), 21.66, 20.87, 20.65, 20.60, 18.70 (2C), 11.92, 11.89, 8.05, 7.88. HRMS (ESI): Calcd for $C_{32}H_{56}NaO_2$ $[M+Na]^+$, 495.4173, Found 495.4189.

Characterization of compound 14

Compound **14**: white solid; yield: 85%. ^1H NMR (400 MHz, $CDCl_3$) δ 5.29 (s, 1H), 3.48−3.33 (m, 4H), 3.30 (s, 3H), 3.29 (s, 3H), 3.13 (d, J = 5.2 Hz, 1H), 2.19−2.08 (m, 1H), 2.08−1.98 (m, 2H), 1.93−1.80 (m, 5H), 1.77−1.64 (m, 4H), 1.60−1.46 (m, 4H), 1.43−1.25 (m, 6H), 1.18−1.10 (m, 5H), 1.08 (s, 3H), 1.02−0.93 (m, 3H), 0.91 (d, J = 6.3 Hz, 3H), 0.87 (d, J = 6.6 Hz, 6H), 0.67 (s, 3H). ^{13}C NMR (100 MHz, $CDCl_3$) δ 143.24, 122.98, 73.67, 69.95, 69.68, 58.61, 58.50, 57.31, 56.15, 51.16, 47.33, 42.26, 39.79, 39.52, 37.06, 36.61, 36.21, 35.80, 34.37, 32.73, 32.46, 30.82, 28.29, 28.00, 25.47, 24.15, 23.88, 22.80, 22.54, 21.51, 20.61, 18.69, 11.89. HRMS (ESI): calcd for $C_{33}H_{58}NaO_3$ $[M+Na]^+$, 525.4278, found 525.4308.

Characterization of compound 15

Compound **15**: white solid; yield: 86%. ^1H NMR (400 MHz, $CDCl_3$) δ 3.97 (s, 1H), 3.49−3.41 (m, 1H), 3.34 (s, 3H), 3.39−3.30 (m, 1H), 3.24 (brs, 1H), 2.60−2.49 (m, 1H), 2.47−2.30 (m, 2H), 2.03−1.92 (m, 2H), 1.92−1.76 (m, 2H), 1.75−1.60 (m, 3H), 1.59−1.43 (m, 5H), 1.42−1.22 (m, 7H), 1.05 (s, 3H), 1.21−1.00 (m, 7H), 0.90 (d, J = 6.4 Hz, 3H), 0.86 (d, J = 6.5 Hz, 6H), 0.68 (s, 3H). ^{13}C NMR (100 MHz, $CDCl_3$) δ 143.31, 128.39, 72.97, 69.68, 58.80, 56.28, 56.25, 53.71, 42.54, 40.01, 39.52, 38.24, 36.17, 35.79, 35.76, 33.72, 32.88, 29.58, 28.79, 28.26, 28.00, 25.83, 24.16, 23.86, 22.80, 22.54, 21.39, 20.49, 18.63, 12.03. HRMS (ESI): Calcd for $C_{30}H_{52}NaO_2$ $[M+Na]^+$, 467.3860, Found 467.3897.

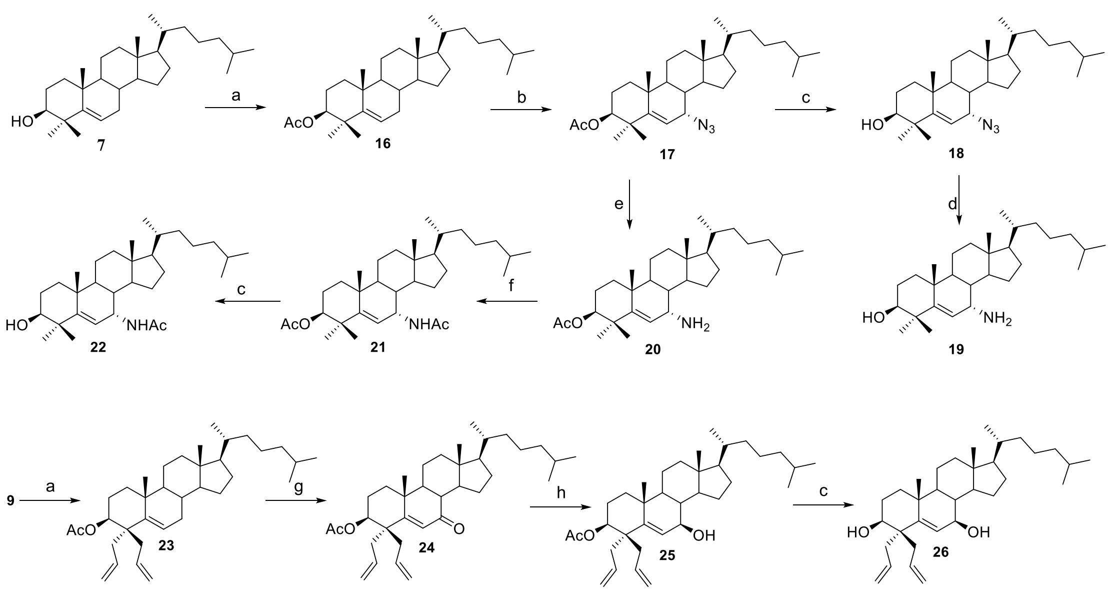

Supplementary Figure 131. The synthesis scheme of compounds 18, 19, 22 and 26. Reagents and conditions: (a) acetic anhydride, pyridine, CH_2Cl_2, r.t., 24 h, 91% (yield of crude) for **16** and 89% for **23**; (b) $TMSN_3$, $Pb(OAc)_4$, CH_2Cl_2, r.t., 4 h, 58% (over two steps); (c) KOH, MeOH, DCM, r.t., 24 h, 88% for **18**, 93% for **22** and 87% for **26**; (d) $LiAlH_4$, THF, reflux, 2 h, 93%; (e) PPh_3, THF, H_2O, reflux, 16 h, 42%; (f) acetic anhydride, pyridine, CH_2Cl_2, r.t., 24 h, 83%; (g) $Na_2Cr_2O_7{\cdot}2H_2O$, NHPI, AcOH, acetone, 50°C, 24 h, 46%; (h) $NaBH_4$, MeOH, THF, r.t., 24 h, 62%.

Synthesis of compound 16

Compound **16**: To a solution of compound **7** (4.14g, 10 mmol) in pyridine (50 mL) was added acetic anhydride (4.4 g, 40 mmol). The reaction mixture was stirred for 24 h at room temperature and then concentrated. CH_2Cl_2 (50 mL) was added to the residue and washed with 10% HCl and brine, then dried over anhydrous Na_2SO_4. The organic layer was concentrated to afford the white solid compound **16** (4.47g, 91%), and used in the next step without purification.

Synthesis of compound 17

Compound **17**: To a solution of compound Me_3SiCl (13.4 g, 0.125 mol) in di-n-butyl ether (30 mL) was added NaN_3 (15 g, 0.225 mol) under N_2 at room temperature. The mixture was heated to 100°C for 48 h, then the mixture was directly distilled and a fraction boiling at a range of 95-96 °C was collected to give Me_3SiN_3 as a colorless

liquid (11.5g, 80%), and used in the next step without purification.

To a solution of compound **16** (0.38 g, 0.83 mmol) and lead (IV) acetate (0.8 g, 1.66 mmol) in CH_2Cl_2 (20mL) was added Me_3SiN_3 (1.6 mL, 11.19 mmol) in drops. The mixture was stirred for 4 h. The solution was diluted with H_2O (10 mL) and the precipitate lead (II) azide was removed by fitration and decomposed with sodium nitrite/dilute hydrochloric acid. The organic layer was dried over anhydrous Na_2SO_4 and concentrated. The residue was purified by silica gel chromatography (petroleum ether/AcOEt, 200/1, v/v) to give compound **17** (0.24g, 58%) as a white solid.

Characterization of compound 17

^{1}H NMR (400 MHz, $CDCl_3$) δ 5.75 (d, J = 5.0 Hz, 1H), 4.61–4.51 (m, 1H), 3.77–3.62 (m, 1H), 2.07 (s, 3H), 2.05–1.97 (m, 1H), 1.94–1.85 (m, 1H), 1.81–1.75 (m, 3H), 1.70–1.62 (m, 2H), 1.55–1.46 (m, 3H), 1.44–1.29 (m, 8H), 1.28–1.22 (m, 3H), 1.20 (s, 3H), 1.13 (s, 3H), 1.12 (s, 3H), 1.07–1.00 (m, 3H), 0.92 (d, J = 6.5 Hz, 3H), 0.88 (d, J = 2.0 Hz, 3H), 0.86 (d, J = 2.0 Hz, 3H), 0.65 (s, 3H).

Synthesis of compound 18

Compound **18**: To a solution of compound **17** (100 mg, 0.20 mmol) in DCM–MeOH mixed solvent (10 mL, 1: 1) was added KOH (160 g, 2.9 mmol). The reaction mixture was stirred for 24 h at room temperature and concentrated. AcOEt (30 mL) was added to the residue and washed with H_2O and brine. The organic layer was dried over anhydrous Na_2SO_4 and concentrated. The residue was purified by silica gel chromatography (petroleum ether/AcOEt, 3/1, v/v) to give compound **18** (70 mg, 88%) as a white solid.

Characterization of compound 18

^{1}H NMR (400 MHz, $CDCl_3$) δ 5.74 (d, J = 4.9 Hz, 1H), 3.67 (t, J = 4.8 Hz, 1H), 3.37–3.28 (m, 1H), 2.04–1.97 (m, 1H), 1.96–1.85 (m, 1H), 1.82–1.71 (m, 3H), 1.70–1.61 (m, 2H), 1.54–1.42 (m, 4H), 1.42–1.27 (m, 7H), 1.25 (s, 3H), 1.23–1.14 (m, 4H), 1.13 (s, 3H), 1.10 (s, 3H), 1.08–0.98 (m, 2H), 0.92 (d, J = 6.5 Hz, 3H), 0.88 (d, J = 1.8 Hz, 3H), 0.86 (d, J = 1.8 Hz, 3H), 0.66 (s, 3H). ^{13}C NMR (100 MHz, $CDCl_3$) δ 157.72, 117.27, 77.22, 59.12, 55.70, 50.54, 44.48, 42.21, 42.03, 39.52, 38.91, 37.87,

36.20, 36.19, 36.15, 35.74, 28.22, 28.03, 27.69, 27.17, 24.08, 23.72, 23.56, 22.82, 22.57, 20.25, 20.17, 18.73, 11.55. HRMS (ESI): calcd for $C_{29}H_{49}N_3NaO$ $[M+Na]^+$, 478.3768, found 478.3782.

Synthesis of compound 19

To a solution of compound **18** (91 mg, 0.2 mmol) in dry THF (20 mL) was added $LiAlH_4$ (38 mg, 1 mmol) under N_2 at room temperature. The reaction mixture was heated to reflux for 2 h. After cooling to room temperature, excessive $Na_2SO_4{\cdot}10H_2O$ was added. Then the reaction mixture was filtered and the filtrate was concentrated to give compound **19** (80 mg, 93%) as a white solid.

Characterization of compound 19

^1H NMR (400 MHz, $CDCl_3$) δ 5.69 (d, J = 4.8 Hz, 1H), 3.29–3.21 (m, 1H), 3.16 (t, J = 4.9 Hz, 1H), 2.05 –1.97 (m, 1H), 1.94–1.82 (m, 1H), 1.72 (dd, J = 10.4, 7.7 Hz, 3H), 1.66–1.50 (m, 4H), 1.50–1.37 (m, 7H), 1.36–1.23 (m, 6H), 1.18 (s, 3H), 1.09 (s, 3H), 1.08 (s, 3H), 1.10–1.04 (m, 2H), 0.93 (d, J = 6.5 Hz, 3H), 0.88 (d, J = 1.6 Hz, 3H), 0.86 (d, J = 1.6 Hz, 3H), 0.68 (s, 3H). ^{13}C NMR (100 MHz, $CDCl_3$) δ 151.74, 124.86, 77.24, 55.74, 50.03, 47.66, 42.66, 42.02, 41.59, 39.54, 39.09, 37.90, 36.50, 36.20, 36.09, 35.73, 28.30, 28.03, 27.67, 27.36, 24.60, 23.72, 23.57, 22.81, 22.58, 20.36, 20.11, 18.76, 11.62. HRMS (ESI): calcd for $C_{29}H_{51}NNaO$ $[M+Na]^+$, 452.3863, found 452.3847.

Synthesis of compound 20

Compound **20**: To a solution of compound **17** (0.25g, 0.50 mmol) in THF (10 mL) was added PPh_3 (0.26 g, 1 mmol) and H_2O (0.25 mL). The reaction mixture was heated to reflux for 16 h, and then concentrated. The residue was purified by silica gel chromatography (petroleum ether/AcOEt, 10/1, v/v) to give **20** (0.1 g, 42%) as a white solid.

Characterization of compound 20

^1H NMR (400 MHz, $CDCl_3$) δ 5.77–5.64 (m, 1H), 4.55–4.42 (m, 1H), 3.22–3.02 (m, 1H), 2.05 (s, 3H), 2.04–1.95 (m, 2H), 1.94–1.82 (m, 1H), 1.80–1.67 (m, 4H), 1.58–1.45 (m, 4H), 1.40–1.23 (m, 8H), 1.23–1.17 (m, 2H), 1.16 (s, 3H), 1.11 (s, 3H),

1.07 (s, 3H), 1.04–0.96 (m, 3H), 0.93 (d, *J* = 6.5 Hz, 3H), 0.87 (d, *J* = 1.8 Hz, 3H), 0.86 (d, *J* = 1.8 Hz, 4H), 0.68 (s, 3H).

Synthesis of compound 21

Compound **21**: To a solution of compound **20** (0.1 g, 0.21 mmol) in CH_2Cl_2 (10 mL) was added acetic anhydride (0.11 mL, 1 mmol) and pyridine (0.08 mL). The reaction mixture was stirred for 24 h at room temperature and washed with 10% HCl, saturated $NaHCO_3$, and brine, then dried overanhydrous Na_2SO_4 and concentrated. The residue was purified by silica gel chromatography (petroleum ether/AcOEt, 3/1, v/v) to give compound **21** (0.09 g, 83%) as a white solid.

Characterization of compound 21

^{1}H NMR (400 MHz, $CDCl_3$) δ 5.56 (d, *J* = 4.9 Hz, 1H), 4.55–4.43 (m, 2H), 2.07 (s, 3H), 2.00 (s, 3H), 1.87−1.69 (m, 7H), 1.69−1.55 (m, 2H), 1.54−1.43 (m, 4H), 1.41−1.30 (m, 5H), 1.22−1.18 (m, 2H), 1.15 (s, 3H), 1.12 (s, 3H), 1.10−1.05 (m, 4H), 1.02 (s, 3H), 0.92 (d, *J* = 6.4 Hz, 3H), 0.87 (d, *J* = 2.0 Hz, 3H), 0.86 (d, *J* = 2.0 Hz, 3H), 0.67 (s, 3H).

Synthesis and characterization of compound 22

By a similar procedure described for **18**, Compound **22** was obtained as a white solid; yield: 93%. ^{1}H NMR (400 MHz, $CDCl_3$) δ 5.56 (d, *J* = 4.9 Hz, 1H), 5.24 (d, *J* = 10.3 Hz, 1H), 4.53–4.43 (m, 1H), 3.29−3.18 (m, 1H), 2.07−1.97 (m, 1H), 2.00 (s, 3H), 1.88−1.75 (m, 3H), 1.71−1.57 (m, 2H), 1.55−1.43 (m, 4H), 1.43−1.21 (m, 6H), 1.20−1.15 (m, 2H), 1.14 (s, 3H), 1.13−1.11 (m, 2H), 1.10 (s, 3H), 1.08 (s, 3H), 1.07–0.95 (m, 4H), 0.92 (d, *J* = 6.4 Hz, 3H), 0.87 (d, *J* = 2.0 Hz, 3H), 0.86 (d, *J* = 2.0 Hz, 3H), 0.67 (s, 3H). ^{13}C NMR (100 MHz, $CDCl_3$) δ 168.91, 152.80, 121.72, 77.18, 55.91, 50.87, 45.62, 45.42, 42.21, 41.65, 39.47, 39.02, 37.65, 36.42, 36.19, 35.76, 34.31, 28.18, 28.02, 27.53, 27.25, 24.35, 23.90, 23.85, 23.51, 22.83, 22.54, 20.23, 20.19, 18.78, 11.61. HRMS (ESI): calcd for $C_{31}H_{53}NNaO_2$ $[M+Na]^+$, 494.3969, found 494.3984.

Synthesis and characterization of compound 23

By a similar procedure described for **16**, **23** was obtained as a white solid; yield: 89%. ^{1}H NMR (400 MHz, $CDCl_3$) δ 5.80−5.60 (m, 2H), 5.41−5.33 (m, 1H), 5.07−4.96 (m, 3H), 4.93−4.84 (m, 1H), 4.76−4.64 (m, 1H), 2.69−2.60 (m, 1H), 2.58−2.49 (m, 1H), 2.24−2.10 (m, 3H), 2.05 (s, 3H), 2.02−1.91 (m, 2H), 1.90−1.75 (m, 4H), 1.75−1.65 (m, 3H), 1.60−1.48 (m, 4H), 1.40−1.30 (m, 5H), 1.23−1.11 (m, 7H), 1.09 (s, 3H), 0.91 (d, J = 6.5 Hz, 3H), 0.87 (d, J = 1.7 Hz, 3H), 0.86 (d, J = 1.7 Hz, 3H), 0.67 (s, 3H).

Synthesis and characterization of compound 24

Compound **24**: To a solution of compound **23** (0.17 g, 0.33 mmol) in acetone (15 mL) was added N-hydroxyphthalimide (NHPI, 0.1 g, 0.61 mmol), AcOH (0.1 mL) and $Na_2Cr_2O_7 \cdot 2H_2O$ (0.1 g, 0.33 mmol). The reaction mixture was stirred for 24 h at 50°C. After cooling to room temperature, the mixture was filtered and concentrated. CH_2Cl_2 (30 mL) was added to the residue and washed with saturated $NaHCO_3$ and brine, then dried over anhydrous Na_2SO_4 and concentrated. The residue was purified by silica gel chromatography (petroleum ether/AcOEt, 10/1, v/v) to give compound **24** (80 mg, 46%) as a white solid. ^{1}H NMR (400 MHz, $CDCl_3$) δ 5.73 (s, 1H), 5.68−5.49 (m, 2H), 5.15−5.01 (m, 3H), 4.98−4.90 (m, 1H), 4.89−4.81 (m, 1H), 2.69−2.54 (m, 2H), 2.40−2.22 (m, 3H), 2.12−2.00 (m, 2H), 2.08 (s, 3H), 1.95−1.82 (m, 3H), 1.81−1.72 (m, 1H), 1.63−1.44 (m, 5H), 1.44−1.29 (m, 8H), 1.26 (s, 3H), 1.18−1.10 (m, 4H), 0.92 (d, J = 6.4 Hz, 3H), 0.87 (d, J = 1.4 Hz, 3H), 0.86 (d, J = 1.4 Hz, 3H), 0.69 (s, 3H).

Synthesis and characterization of compound 25

Compound **25**: To a solution of compound **24** (80 mg, 0.15 mmol) in MeOH−THF mixed solvent (10 mL, 1:1) was added $NaBH_4$ (23 mg, 0.61 mmol). The reaction mixture was stirred for 24 h at room temperature and concentrated. H_2O (20 mL) was added to the residue and extracted with AcOEt (10 mL×3). The combined organic extract was washed with brine, dried over anhydrous Na_2SO_4 and concentrated. The residue was purified by silica gel chromatography (petroleum ether/AcOEt, 10/1, v/v) to give compound **25** (50 mg, 62%) as a white solid. ^{1}H NMR (400 MHz, $CDCl_3$) δ 5.79−5.65 (m, 2H), 5.34 (d, J = 3.3 Hz, 1H), 5.11−4.97 (m, 3H), 4.94−4.86 (m, 1H), 4.77−4.69 (m, 1H), 4.00−3.90 (m, 1H), 2.68−2.53 (m, 2H), 2.27−2.18 (m, 1H), 2.06 (s, 3H), 2.04−1.95 (m, 2H), 1.95−1.85 (m, 1H), 1.85−1.76 (m, 3H), 1.55−1.45 (m, 3H),

1.45–1.24 (m, 9H), 1.17 (s, 3H), 1.16–1.05 (m, 7H), 0.92 (d, *J* = 6.5 Hz, 3H), 0.88 (d, *J* = 1.9 Hz, 3H), 0.86 (d, *J* = 1.9 Hz, 3H), 0.69 (s, 3H).

Synthesis and characterization of compound 26

By a similar procedure described for **18**, **26** was obtained as a white solid; yield: 87%. ^{1}H NMR (400 MHz, $CDCl_3$) δ 5.92–5.67 (m, 2H), 5.35 (d, *J* = 3.3 Hz, 1H), 5.18–5.00 (m, 4H), 3.95 (dd, *J* =7.3, 2.6 Hz, 1H), 3.64 (dd, *J* = 11.8, 3.8 Hz, 1H), 2.64–2.45 (m, 2H), 2.36–2.21 (m, 2H), 2.09–1.98 (m, 1H), 1.96–1.77 (m, 3H), 1.75–1.60 (m, 4H), 1.57–1.47 (m, 3H), 1.43–1.32 (m, 6H), 1.15 (s, 6H), 1.11–1.04 (m, 4H), 0.92 (d, *J* = 6.5 Hz, 3H), 0.88 (d, *J* = 2.0 Hz, 3H), 0.86 (d, *J* = 2.0 Hz, 3H), 0.69 (s, 3H). ^{13}C NMR (100 MHz, $CDCl_3$) δ 144.90, 136.01, 135.22, 128.13, 117.49, 117.21, 73.81, 73.03, 56.16, 55.53, 49.93, 47.25, 42.62, 40.35, 39.42 (3C), 38.88, 36.76, 36.63, 36.14, 35.70, 28.43, 27.92, 26.15, 25.96, 23.79, 22.72, 22.46, 21.07, 20.67, 18.63, 11.69. HRMS (ESI): calcd for $C_{33}H_{54}NaO_2$ $[M+Na]^+$, 505.4016, found 505.4001.

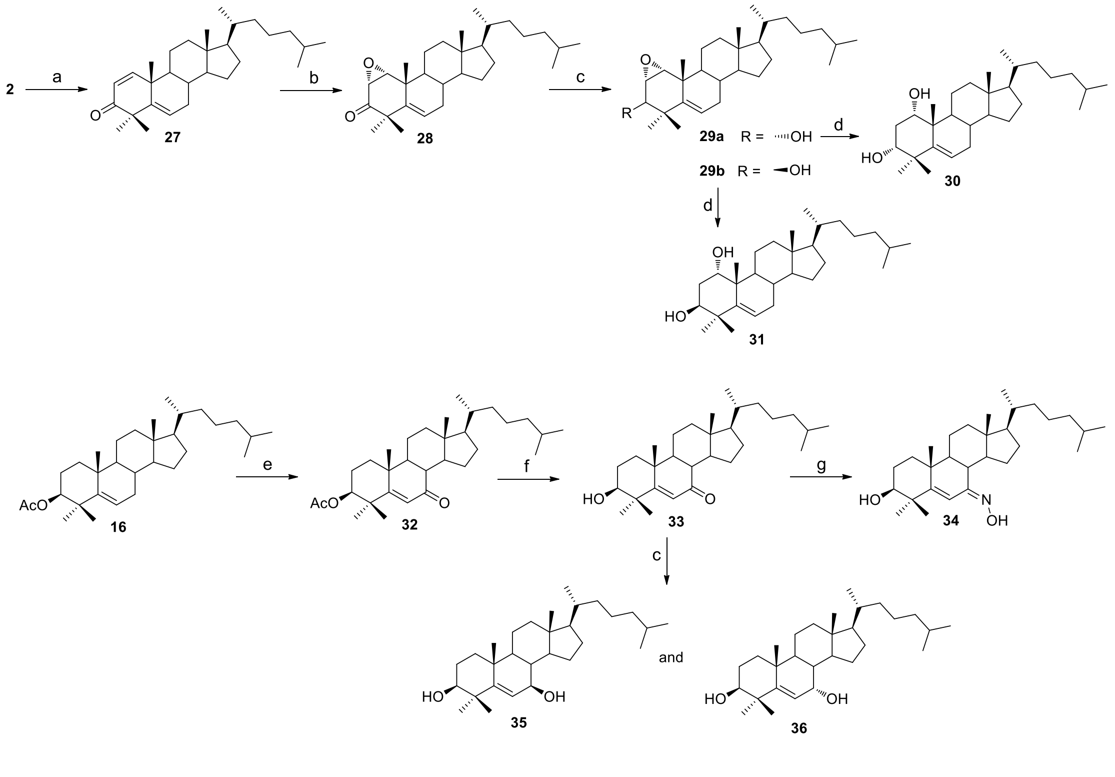

Supplementary Figure 132. The synthesis scheme of compunds 30, 31 and 33-36. Reagents and conditions: (a) IBX, TsOH, DMSO, toluene, 80°C, 6 h, 70%; (b) 30% H_2O_2, THF, MeOH, 20% NaOH, r.t., 4 h, 87%; (c) $NaBH_4$, MeOH, THF, r.t., 12 h, 60% for **29a**, 30% for **29b**, 72% for **35** and 2% for **36**; (d) $LiAlH_4$, THF, reflux, 5 h, 71%for **30** and 87% for **31**; (e) $Na_2Cr_2O_7 \cdot 2H_2O$, NHPI, AcOH, acetone, 50°C, 48 h,

61%; (f) KOH, MeOH, r.t., 6 h, 92%; (g) $NH_2OH·HCl$, pyridine, r.t., 24 h, 56%.

Synthesis and characterization of compound 27

Compound **27**: To a solution of compound **2** (2.27 g, 5.5 mmol) in DMSO-toluene mixed solvent (100 mL, 1: 1) was added IBX (9.24 g, 33 mmol) and TsOH (0.52 g, 2.75 mmol). The reaction mixture was stirred for 6 h at 80°C. After cooling to room temperature, the mixture was filtered. The filtrate was poured into water and extracted with AcOEt (50 mL×3). The combined organic extract was washed with H_2O and brine, dried over anhydrous Na_2SO_4 and concentrated. The residue was purified by silica gel chromatography (petroleum ether/AcOEt, 150/1, v/v) to give compound **27** (1.6 g, 70%) as a white solid. ^1H NMR (400 MHz, $CDCl_3$) δ 6.91 (d, J = 10.3 Hz, 1H), 5.89 (d, J = 10.3 Hz, 1H), 5.66–5.58 (m, 1H), 2.26–2.12 (m, 1H), 2.11–2.03 (m, 1H), 1.92−1.79 (m, 1H), 1.75–1.64 (m, 3H), 1.64–1.46 (m, 4H), 1.45–1.35 (m, 2H), 1.34 (s, 3H), 1.29 (s, 3H), 1.27 (s, 3H), 1.24–1.05 (m, 8H), 1.05–0.96 (m, 2H), 0.92 (d, J = 6.5 Hz, 3H), 0.88 (d, J = 1.2 Hz, 3H), 0.86 (d, J = 1.3 Hz, 3H), 0.72 (s, 3H).

Synthesis and characterization of compound 28

Compound **28**: To a solution of compound **27** (1.1 g, 2.68 mmol) in THF−MeOH mixed solvent (20 mL, 1:1) was added 30% H_2O_2 (1.51 mL, 15 mmol) and 20% NaOH (2.68 mL, 13.4 mmol) in drops at 0°C. After stirring for 0.5 h at 0°C, the reaction mixture was stirred for another 4 h at room temperature. The THF−MeOH mixed solvent were removed and H_2O (30 mL) was added. The organic phase was washed with sodium sulfite solution, 10% hydrochloric acid solution, saturated sodium bicarbonate solution and brine, dried over anhydrous Na_2SO_4 and concentrated. The residue was purified by silica gel chromatography (petroleum ether/AcOEt, 10/1 v/v) to give compound **28** (1.0 g, 87%) as a white solid. ^1H NMR (400 MHz, $CDCl_3$) δ 5.68 (dd, J = 5.0, 2.0 Hz, 1H), 3.60 (d, J = 4.7 Hz, 1H), 3.49 (d, J = 4.7 Hz, 1H), 2.16–2.04 (m, 2H), 1.92−1.80 (m, 1H), 1.76−1.66 (m, 2H), 1.58−1.45 (m, 4H), 1.45−1.31 (m, 4H), 1.30 (s, 3H), 1.28−1.22 (m, 2H), 1.19 (s, 3H), 1.17−1.05 (m, 6H), 1.05−0.98 (m, 1H), 0.95 (s, 3H), 0.93 (d, J = 6.5 Hz, 3H), 0.88 (d, J = 1.6 Hz, 3H), 0.86 (d, J = 1.6 Hz, 3H), 0.70 (s, 3H).

Synthesis and characterization of compound 29a

By a similar procedure described for **7a–11**, **29a** and **29b** were obtained as white solids. Compound **29a**: yield: 60%. ^{1}H NMR (400 MHz, $CDCl_3$) δ 5.52–5.44 (m, 1H), 3.61–3.53 (m, 2H), 3.21–3.15 (m, 1H), 2.16–2.07 (m, 1H), 2.07–2.00 (m, 1H), 1.96–1.89 (m, 1H), 1.89–1.80 (m, 1H), 1.75–1.64 (m, 2H), 1.64 - 1.60 (m, 1H), 1.58–1.47 (m, 3H), 1.43–1.31 (m, 4H), 1.30–1.23 (m, 2H), 1.21 (s, 3H), 1.17–1.09 (m, 4H), 1.08 (s, 3H), 1.06 (s, 3H), 1.04–0.97 (m, 2H), 0.92 (d, J = 6.5 Hz, 3H), 0.88 (d, J = 1.6 Hz, 3H), 0.86 (d, J = 1.6 Hz, 3H), 0.70 (s, 3H).

Characterization of compound 29b

Compound **29b**: yield: 30%. ^{1}H NMR (400 MHz, $CDCl_3$) δ 5.53–5.46 (m, 1H), 3.37 (s, 1H), 3.22 (d, J = 3.7 Hz, 1H), 3.06 (d, J = 3.8 Hz, 1H), 2.16–2.08 (m, 1H), 2.06–1.99 (m, 1H), 1.94 (brs, J = 1.0 Hz, 1H), 1.89–1.78 (m, 1H), 1.74–1.63 (m, 2H), 1.58–1.48 (m, 3H), 1.41–1.29 (m, 5H), 1.29–1.25 (m, 2H), 1.24 (s, 3H), 1.23 - 1.19 (m, 1H), 1.18–1.11 (m, 3H), 1.09 (s, 3H), 1.08 (s, 3H), 1.06 - 0.95 (m, 3H), 0.92 (d, J = 6.5 Hz, 3H), 0.87 (d, J = 1.7 Hz, 3H), 0.86 (d, J = 1.7 Hz, 3H), 0.70 (s, 3H).

Synthesis and characterization of compound 30

Compound **30**: To a solution of compound **29a** (0.77g, 1.8 mmol) in dry THF (20 mL) was added $LiAlH_4$ (0.136 g, 3.6 mmol) under N_2. The reaction mixture was heated to reflux for 5 h. After cooling to room temperature, excessive $Na_2SO_4 \cdot 10H_2O$ was added to the reaction. Then the reaction mixture was filtered and the filtrate was concentrated to give compound **30** (0.55 g, 71%) as a white solid. ^{1}H NMR (400 MHz, $CDCl_3$) δ 5.70–5.63 (m, 1H), 3.76 (s, 1H), 3.61 (s, 1H), 3.28 (brs, 1H), 2.93 (brs, 1H), 2.32–2.23 (m, 1H), 2.14–1.96 (m, 3H), 1.91–1.78 (m, 1H), 1.74–1.64 (m, 2H), 1.61 (d, J = 16.3 Hz, 4H), 1.54–1.42 (m, 3H), 1.41–1.30 (m, 3H), 1.29–1.22 (m, 2H), 1.20 (s, 3H), 1.15 (s, 3H), 1.10 (s, 3H),1.13–1.07 (m, 3H), 1.06–0.98 (m, 2H), 0.91 (d, J = 6.5 Hz, 3H), 0.87 (d, J = 1.5 Hz, 3H), 0.86 (d, J = 1.5 Hz, 3H), 0.68 (s, 3H). ^{13}C NMR (100 MHz, $CDCl_3$) δ 144.43, 124.04, 78.04, 73.50, 57.22, 56.08, 43.17, 42.15, 42.13, 40.60, 39.53, 39.50, 36.20, 35.83, 32.65, 31.32, 30.92, 29.84, 28.26, 28.12, 28.02, 24.27, 23.88, 22.83, 22.57, 21.80, 20.00, 18.72, 11.88. HRMS (ESI): calcd for $C_{29}H_{50}NaO_2$ $[M+Na]^+$, 453.3703, found 453.3693.

Synthesis and characterization of compound 31

By a similar procedure described for **30**, **31** was obtained as a white solid; yield: 87%. ^{1}H NMR (400 MHz, $CDCl_3$) δ 5.78–5.71 (m, 1H), 3.84–3.72 (m, 2H), 2.14–1.88 (m, 4H), 1.88–1.78 (m, 1H), 1.72–1.59 (m, 2H), 1.56–1.44 (m, 5H), 1.42–1.24 (m, 7H), 1.19 (s, 3H), 1.17–1.12 (m, 2H), 1.11 (s, 3H), 1.08 (s, 3H), 1.06–0.95 (m, 3H), 0.91 (d, J = 6.5 Hz, 3H), 0.86 (d, J = 6.5 Hz, 6H), 0.68 (s, 3H). ^{13}C NMR (100 MHz, $CDCl_3$) δ 146.93, 123.29, 72.42, 71.67, 57.19, 56.07, 43.24, 42.21, 41.59, 41.26, 39.52 (2C), 36.19, 35.80, 34.31, 32.51, 30.76, 28.25, 28.02, 27.36, 24.71, 24.26, 23.86, 22.84, 22.57, 21.37, 20.02, 18.72, 11.88. HRMS (ESI): calcd for $C_{29}H_{50}NaO_2$ $[M+Na]^+$ 453.3703, found 453.3697.

Synthesis and characterization of compound 32

By a similar procedure described for **24**, **32** was obtained as a white solid; yield: 61%. ^{1}H NMR (500 MHz, $CDCl_3$) δ 5.93 (s, 1H), 4.60 (t, J = 8.0 Hz, 1H), 2.30–2.25 (m, 1H), 2.24–2.15 (m, 1H), 2.08 (s, 3H), 2.06–2.01 (m, 1H), 1.87–1.76 (m, 4H), 1.61–1.55 (m, 3H), 1.57–1.46 (m, 4H), 1.55–1.41 (m, 4H), 1.42–1.28 (m, 3H), 1.27 (s, 3H), 1.21 (s, 3H), 1.20-1.05 (m, 3H), 1.11 (s, 3H), 0.92 (d, J = 6.5 Hz, 3H), 0.87 (d, J = 2.0 Hz, 3H), 0.85 (d, J = 2.5 Hz, 3H), 0.69 (s, 3H).

Synthesis and characterization of compound 33

By a similar procedure described for **18**, **33** was obtained as a white solid; yield: 92%. ^{1}H NMR (400 MHz, $CDCl_3$) δ 5.94 (s, 1H), 3.46–3.34 (m, 1H), 2.38–2.17 (m, 2H), 2.09–1.99 (m, 1H), 1.95–1.76 (m, 4H), 1.66–1.62 (m, 1H), 1.58–1.45 (m, 4H), 1.44–1.29 (m, 6H), 1.25 (s, 3H), 1.23 (s, 3H), 1.14 (s, 3H), 1.18 - 0.97 (m, 6H), 0.92 (d, J = 6.5 Hz, 3H), 0.87 (d, J = 1.6 Hz, 3H), 0.86 (d, J = 1.6 Hz, 3H), 0.69 (s, 3H). ^{13}C NMR (100 MHz, $CDCl_3$) δ 203.07, 175.89, 125.11, 76.04, 54.89, 51.87, 50.73, 45.21, 43.33, 42.61, 39.48, 38.87, 38.51, 36.19, 35.77, 35.39, 28.57, 28.00, 26.94, 26.41, 26.25, 23.86, 23.15, 22.81, 22.55, 20.91, 19.60, 18.85, 11.94. HRMS (ESI): calcd for $C_{29}H_{48}NaO_2$ $[M+Na]^+$, 451.3547, found 451.3538.

Synthesis and characterization of compound 34

Compound **34**: To a solution of compound **33** (0.26 g, 0.61 mmol) in pyridine (15 mL) was added $NH_2OH{\cdot}HCl$ (0.31 g, 4.5 mmol). The reaction mixture was stirred for 24 h at room temperature and then concentrated. H_2O (20 mL) was added to the residue

and extracted with AcOEt (20 mL×3). The combined organic extract was washed with 10% hydrochloric acid, brine, then dried over anhydrous Na_2SO_4 and concentrated. The residue was purified by silica gel chromatography (petroleum ether/AcOEt, 3/1 v/v) to give compound **34** (0.15 g, 56%) as a white solid. ^{1}H NMR (400 MHz, $CDCl_3$) δ 6.90 (s, 1H), 3.35 (dd, *J* = 10.8, 4.3 Hz, 1H), 2.35 (t, *J* = 11.2 Hz, 1H), 2.28–2.17 (m, 1H), 2.08–1.99 (m, 1H), 1.91–1.72 (m, 4H), 1.57–1.45 (m, 3H), 1.41–1.32 (m, 4H), 1.29 (s, 3H), 1.32–1.20 (m, 4H), 1.15 (s, 3H), 1.11 (s, 3H), 1.20–0.96 (m, 7H), 0.93 (d, *J* = 6.5 Hz, 3H), 0.87 (d, *J* = 1.5 Hz, 3H), 0.85 (d, *J* = 1.4 Hz, 3H), 0.71 (s, 3H). ^{13}C NMR (100 MHz, $CDCl_3$) δ 163.93, 157.91, 111.10, 76.67, 54.88, 51.82, 51.52, 43.18, 42.79, 39.51, 38.90, 38.38, 37.52, 36.17, 35.77, 35.70, 28.37, 28.01, 27.61, 27.11, 26.64, 23.84, 23.40, 22.82, 22.57, 20.71, 19.97, 18.92, 12.23. HRMS (ESI): calcd for $C_{29}H_{49}NNaO_2$ $[M+Na]^+$, 466.3656, found 466.3639.

Synthesis and characterization of compound 35

By a similar procedure described for **7−11**, **35** and **36** were obtained as white solids.

Compound **35**: yield: 72%. ^{1}H NMR (400 MHz, $CDCl_3$) *δ* 5.52 (d, *J* = 2.9 Hz, 1H), 3.91 (d, *J* = 6.4 Hz, 1H), 3.26 (dd, *J* = 10.0, 4.1 Hz, 1H), 2.06−1.97 (m, 1H), 1.94−1.85 (m, 1H), 1.84−1.68 (m, 4H), 1.59−1.24 (m, 12H), 1.18 (s, 3H), 1.14 (s, 3H), 1.10 (s, 3H), 1.12−1.05 (m, 4H), 1.05−0.96 (m, 2H), 0.92 (d, *J* = 6.5 Hz, 3H), 0.87 (d, *J* = 1.3 Hz, 3H), 0.86 (d, *J* = 1.4 Hz, 3H), 0.69 (s, 3H). ^{13}C NMR (100 MHz, $CDCl_3$) *δ* 153.36, 123.67, 77.18, 74.10, 56.25, 55.50, 49.88, 42.71, 41.49, 40.37, 39.51 (2C), 36.92, 36.29, 36.22, 35.75, 28.54, 28.02, 27.31, 27.10, 26.19, 23.86, 23.34, 22.82, 22.56, 21.37, 20.71, 18.75, 11.79. HRMS (ESI): calcd for $C_{29}H_{50}NaO_2$ $[M+Na]^+$, 453.3703, found 453.3691.

Characterization of compound 36

Compound **36**: yield: 2%. ^{1}H NMR (400 MHz, CDCl3) *δ* 5.77 (dd, *J* = 4.9, 1.6 Hz, 1H), 3.94−3.86 (m, 1H), 3.31−3.21 (m, 1H), 2.04−1.95 (m, 1H), 1.94−1.83 (m, 1H), 1.80−1.63 (m, 5H), 1.56−1.38 (m, 6H), 1.37−1.23 (m, 5H), 1.19 (s, 3H), 1.16−1.10 (m, 6H), 1.08 (s, 3H), 1.06 (s, 3H), 0.91 (d, *J* = 6.5 Hz, 3H), 0.87−0.85 (m, 3H), 0.85 - 0.83 (m, 3H), 0.66 (s, 3H). ^{13}C NMR (100 MHz, $CDCl_3$) *δ* 155.18, 122.63, 65.93, 55.73, 49.29, 43.37, 41.90, 41.67, 39.46 (2C), 39.00, 37.81, 36.71, 36.18, 36.12, 35.69,

28.25, 27.91, 27.34, 27.18, 24.27, 23.64, 23.45, 22.69, 22.46, 20.07, 19.85, 18.66, 11.42. HRMS (ESI): calcd for $C_{29}H_{50}NaO_2$ $[M+Na]^+$, 453.3703, found 453.3729.

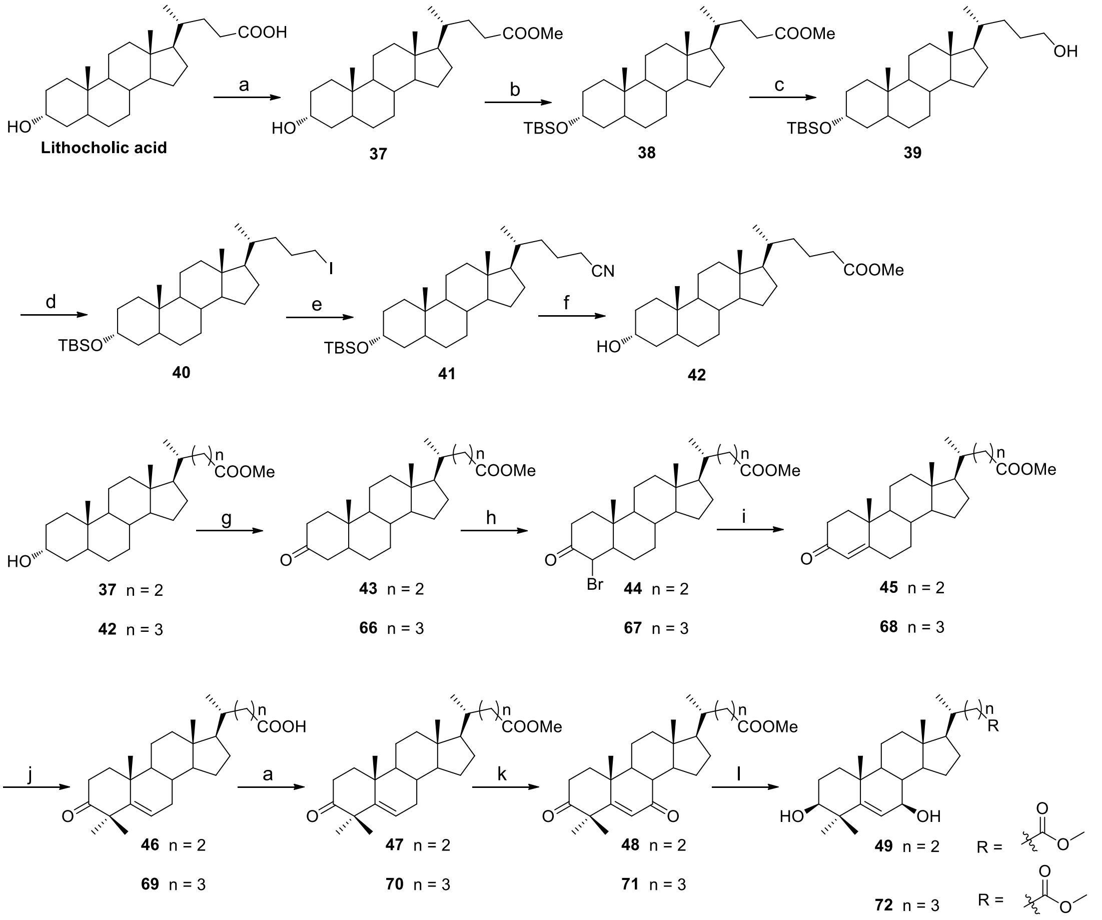

Supplementary Figure 133. The synthesis scheme of compounds 49 and 72. Reagents and conditions: (a) $SOCl_2$, MeOH, r.t., 5 h, 99%; (b) TBSCl, Imidazole, DMF, 80°C, 6 h, 86%; (c) $LiAlH_4$, THF, r.t., 12 h, 96%; (d) I_2, PPh_3, Imidazole, toluene, r.t., 2 h, 98%; (e) NaCN, THF, DMSO, 60°C, 6 h, 97%; (f) HCl/MeOH, reflux, 1 h, 99%; (g) PCC, CH_2Cl_2, r.t., 12 h, 92.3% for **43** and 94% for **66**; (h)Br_2, HOAc, CH_2Cl_2, r.t., 1 h; (i) Li_2CO_3, $LiBr{\cdot}H_2O$, DMF, 90°C, 6 h, 48% for **45** (over two steps) and 50% for **68** (over two steps); (j) CH_3I, t-BuOK, t-BuOH, r.t., 24 h, 50% for **47** (over two steps) and 60% for **70** (over two steps); (k) $Na_2Cr_2O_7{\cdot}2H_2O$, NHPI, AcOH, acetone, 50°C, 24 h, 89% for **48** and 85% for **71**; (l) $NaBH_4$, MeOH, r.t., 12 h, 80% for **49** and 81% for **72** .

Synthesis and characterization of compound 37

Compound **37**: To a solution of Lithocholic acid (3.76 g, 10 mmol) in dry MeOH (150 mL) was added $SOCl_2$ (4.76 g, 40 mmol) at 0°C. The reaction mixture was stirred for 5 h at room temperature and then concentrated. AcOEt (250 mL) was added to the residue, and then washed with saturated $NaHCO_3$ and brine, dried over anhydrous Na_2SO_4 and concentrated. The residue was purified by silica gel chromatography (petroleum ether/AcOEt, 3/1 v/v) to give compound **37** (3.88 g, 99%) as a white solid. ^{1}H NMR (500 MHz, $CDCl_3$) δ 3.67 (s, 3H), 3.59–3.65 (m, 1H), 2.30–2.40 (m, 1H), 2.20–2.26 (m, 1H), 1.93–1.99 (m, 1H), 1.74–1.90 (m, 5H), 1.63–1.73 (m, 2H), 1.57–1.60 (m, 1H), 1.48–1.54 (m, 1H), 1.23–1.44 (m, 11H), 1.02–1.17 (m, 5H), 0.92 (s, 3H), 0.91 (d, J = 6.8 Hz, 3H), 0.64 (s, 3H).

Synthesis and characterization of compound 38

Compound **38**: To a solution of compound **37** (8.69 g, 22.25 mmol) in dry DMF (30 mL) was added TBSCl (8.38 g, 56 mmol) and imidazole (7.57 g, 111 mmol) under N_2. The reaction mixture was stirred for 6 h at 80°C and then poured into ice water (40 mL), extracted with AcOEt (80 mL×3). The combined organic extract was washed with H_2O and brine, dried over anhydrous Na_2SO_4 and concentrated. The residue was purified by silica gel chromatography (petroleum ether/AcOEt, 20/1 v/v) to give compound **38** (9.66 g, 86%) as a white solid. ^{1}H NMR (500 MHz, $CDCl_3$) δ 3.66 (s, 3H), 3.54–3.62 (m, 1H), 2.30–2.40 (m, 1H), 2.16–2.27 (m, 1H), 1.91–1.99 (m, 1H), 1.71–1.89 (m, 5H), 1.51–1.63 (m, 3H), 1.31–1.48 (m, 9H), 1.19–1.29 (m, 3H), 1.01–1.17 (m, 5H), 0.88–0.93 (m, 15H), 0.63 (s, 3H), 0.06 (s, 6H).

Synthesis and characterization of compound 39

Compound **39**: To a solution of compound **38** (5.05 g, 10 mmol) in dry THF (50 mL) was added $LiAlH_4$ (759 mg, 20 mmol) under N_2 at 0°C. The reaction mixture was stirred 12 h at room temperature and added excessive $Na_2SO_4 \cdot 10H_2O$. Then the reaction mixture was filtered and the filtrate was concentrated. The residue was purified by silica gel chromatography (petroleum ether/AcOEt, 5/1 v/v) to give compound **39** (4.58 g, 96%) as a white solid. ^{1}H NMR (500 MHz, $CDCl_3$) δ 3.50–3.68 (m, 3H), 1.92–1.99 (m, 1H), 1.72–1.87 (m, 4H), 1.59–1.69 (m, 1H), 1.51–1.57 (m, 2H), 1.31–1.49 (m, 10H), 1.18–1.28 (m, 4H), 1.00–1.16 (m, 6H), 0.92 (d, J = 6.5 Hz, 3H), 0.89 (s, 12H), 0.64 (s, 3H), 0.06 (s, 6H).

Synthesis and characterization of compound 40

Compound **40**: To a solution of compound **39** (8.2 g, 17.2 mmol), PPh_3 (22.55 g, 86 mmol) and imidazole (11.71 g, 172 mmol) in dry toluene (250 mL) was added I_2 (21.85 g, 86 mmol). The reaction mixture was stirred for 2 h at room temperature. The reaction mixture was filtered and the filtrate was concentrated. The residue was purified by silica gel chromatography (petroleum ether/AcOEt, 25/1 v/v) to give compound **40** (9.89 g, 98%) as a white solid. ^{1}H NMR (500 MHz, $CDCl_3$) δ 3.53–3.62 (m, 1H), 3.08–3.24 (m, 2H), 1.91–1.97 (m, 1H), 1.67–1.91 (m, 6H), 1.52–1.59 (m, 2H), 1.31–1.48 (m, 9H), 1.20–1.29 (m, 3H), 1.00–1.20 (m, 7H), 0.88–0.92 (m, 15H), 0.63 (s, 3H), 0.06 (s, 6H).

Synthesis and characterization of compound 41

Compound **41**: To a solution of compound **40** (13.49 g, 22.9 mmol) in THF-DMSO mixed solvent (100 mL, 2:3) was added NaCN (3.37 g, 68.7 mmol). The reaction mixture was stirred for 6 h at 60°C. After cooling to room temperature, the mixture was poured into H_2O (50 mL) and extracted with AcOEt (100 mL×3). The combined organic extract was washed with H_2O and brine, dried over anhydrous Na_2SO_4 and concentrated. The residue was purified by silica gel chromatography (petroleum ether/AcOEt, 20/1 v/v) to give compound **41** (10.8 g, 97%) as a white solid. ^{1}H NMR (500 MHz, $CDCl_3$) δ 3.53–3.64 (m, 1H), 2.24–2.37 (m, 2H), 1.90–1.98 (m, 1H), 1.68–1.87 (m, 5H), 1.47–1.59 (m, 5H), 1.31–1.47 (m, 8H), 1.16–1.28 (m, 4H), 1.01–1.16 (m, 5H), 0.92 (d, J = 6.6 Hz, 3H), 0.90 (s, 3H), 0.89 (s, 9H), 0.64 (s, 3H), 0.06 (s, 6H).

Synthesis and characterization of compound 42

Compound **42**: To a solution of HCl/MeOH (2.0 M, 20 mL) was added compound **41** (4.86 g, 10 mmol). The reaction mixture was heated to reflux for 1 h, then concentrated. The residue was dissolved in AcOEt (100 mL). The organic layer was washed with saturated $NaHCO_3$ and brine, dried over anhydrous Na_2SO_4 and concentrated. The residue was purified by silica gel chromatography (petroleum ether/AcOEt, 3/1 v/v) to give compound **42** (4.0 g, 99%) as a white solid. ^{1}H NMR (500 MHz, $CDCl_3$) δ 3.65 (s, 3H), 3.57–3.64 (m, 1H), 2.19–2.33 (m, 2H), 1.92–1.98

(m, 1H), 1.62−1.88 (m, 6H), 1.43−1.57 (m, 3H), 1.28−1.43 (m, 8H), 1.17−1.28 (m, 3H), 1.00−1.16 (m, 6H), 0.93−1.00 (m, 1H), 0.91 (d, *J* = 6.2 Hz, 3H), 0.90 (s, 3H), 0.62 (s, 3H). ^{13}C NMR (125 MHz, $CDCl_3$) δ 174.33, 71.82, 56.47, 55.99, 51.40, 42.66, 42.08, 40.41, 40.14, 36.43, 35.82, 35.50, 35.37, 35.33, 34.54, 34.50, 30.52, 28.22, 27.18, 26.40, 24.18, 23.35, 21.57, 20.79, 18.52, 11.98. HRMS (ESI): calcd for $C_{26}H_{44}NaO_3$ $[M+Na]^+$, 427.3183, found 427.3161.

Synthesis and characterization of compound 43

Compound **43**: To a solution of compound **37** (3.88 g, 10 mmol) in CH_2Cl_2 (120 mL) was added PCC (4.32 g, 20 mmol). The reaction mixture was stirred for 12 h at room temperature, then filtered and the filtrate was concentrated. The residue was dissolved in AcOEt (200 mL). The organic layer was washed with saturated $NaHSO_3$ and brine, dried over anhydrous Na_2SO_4 and concentrated. The residue was purified by silica gel chromatography (petroleum ether/AcOEt, 7/1 v/v) to give compound **43** (3.56 g, 92.3%) as a white solid. ^{1}H NMR (500 MHz, $CDCl_3$) δ 3.67 (s, 3H), 2.70 (t, *J* = 14.2 Hz, 1H), 2.29−2.40 (m, 2H), 2.20−2.26 (m, 1H), 2.13−2.20 (m, 1H), 1.99−2.06 (m, 3H), 1.76−1.92 (m, 4H), 1.57−1.63 (m, 1H), 1.28−1.54 (m, 10H), 1.19−1.24 (m, 1H), 1.05−1.16 (m, 4H), 1.02 (s, 3H), 0.92 (d, *J* = 6.5 Hz, 3H), 0.68 (s, 3H).

Synthesis and characterization of compound 66

By a similar procedure described for **43**, **66** was obtained as a white solid; yield: 94%. ^{1}H NMR (500 MHz, $CDCl_3$) δ 3.67 (s, 3H), 2.65−2.74 (m, 1H), 2.20−2.38 (m, 3H), 2.12−2.19 (m, 1H), 1.99−2.07 (m, 3H), 1.77−1.93 (m, 3H), 1.65−1.77 (m, 1H), 1.56−1.61 (m, 1H), 1.33−1.54 (m, 9H), 1.03−1.29 (m, 8H), 1.02 (s, 3H), 0.93 (d, *J* = 6.6 Hz, 3H), 0.68 (s, 3H).

Synthesis and characterization of compound 44

Compound **44**: To a solution of compound **43** (3.56 g, 9.2 mmol) in CH_2Cl_2 (150 mL) and HOAc (20 mL) was added bromine (1.62 g, 0.5 mL, 10.1 mmol) at 0 °C. The reaction mixture was stirred for 1 h at room temperature and then poured into H_2O (50 mL). After the separation of organic layer, the aqueous phase was extracted with CH_2Cl_2 (30 mL×3). The combined organic extract was washed with saturated

$NaHSO_3$ and brine, dried over anhydrous Na_2SO_4 and concentrated to give crude product **44** as a light yellow oil, which was used without further purification.

Synthesis and characterization of compound 45

Compound **45**: To a solution of compound **44** in dry DMF (60 mL) was added Li_2CO_3 (2.72 g, 36.8 mmol) and $LiBr{\cdot}H_2O$ (1.93 g, 18.4 mmol). The resulting mixture was stirred for 6 h at 90 °C. After cooling to room temperature, insoluble material was filtered out. The filtrate was dilute with AcOEt (200 mL). The organic layer was washed with brine, dried over anhydrous Na_2SO_4 and concentrated. The residue was purified by silica gel chromatography (petroleum ether/AcOEt, 6/1 v/v) to give compound **45** (1.83 g, 48% over two steps) as a white solid. ^{1}H NMR (500 MHz, $CDCl_3$) δ 5.72 (s, 1H), 3.67 (s, 3H), 2.30–2.46 (m, 4H), 2.18–2.30 (m, 2H), 1.98–2.06 (m, 2H), 1.75–1.93 (m, 3H), 1.65–1.73 (m, 1H), 1.57–1.63 (m, 1H), 1.48–1.56 (m, 2H), 1.38–1.47 (m, 2H), 1.23–1.37 (m, 3H), 1.18 (s, 3H), 1.15–1.17 (m, 1H), 1.07–1.14 (m, 2H), 0.97–1.07 (m, 2H), 0.92 (d, J = 6.5 Hz, 3H), 0.71 (s, 3H). ^{13}C NMR (125 MHz, $CDCl_3$) δ 199.52, 174.60, 171.46, 123.71, 55.77, 55.68, 53.68, 51.42, 42.36, 39.51, 38.51, 35.62, 35.52, 35.25, 33.92, 32.85, 31.93, 30.95, 30.86, 27.98, 24.07, 20.94, 18.17, 17.31, 11.89.

Characterization of compound 68

By similar procedures described for **45**, **68** was obtained as a white solid; yield: 50% (over two steps). ^{1}H NMR (500 MHz, $CDCl_3$) δ 5.72 (s, 1H), 3.67 (s, 3H), 2.21–2.46 (m, 6H), 1.97–2.06 (m, 2H), 1.80–1.90 (m, 2H), 1.65–1.76 (m, 2H), 1.59–1.64 (m, 1H), 1.47–1.56 (m, 3H), 1.34–1.48 (m, 3H), 1.21–1.34 (m, 2H), 1.18 (s, 3H), 0.97–1.17 (m, 6H), 0.93 (d, J = 6.5 Hz, 3H), 0.71 (s, 3H). ^{13}C NMR (125 MHz, $CDCl_3$) δ 199.59, 174.26, 171.59, 123.71, 55.79, 55.69, 53.71, 51.41, 42.34, 39.53, 38.54, 35.63, 35.54, 35.42, 35.28, 34.44, 33.94, 32.88, 31.96, 28.05, 24.10, 21.46, 20.96, 18.48, 17.33, 11.88.

Synthesis and characterization of compound 46

Compound **46**: To a solution of compound **45** (1.83 g, 4.7 mmol) in dry tert-butanol (30 mL) was added potassium tert-butoxide and CH_3I (6.76 g, 47.0 mmol) under N_2 at 0°C. The reaction mixture was stirred for 24 h at room temperature and then

concentrated. The residue was poured into ice water (50 mL) and extracted with AcOEt (50 mL×3). The combined organic extract was washed with H_2O and brine, dried over anhydrous Na_2SO_4 and concentrated to give crude product **46** as a yellow oil, which was used without further purification.

Synthesis and characterization of compound 47

By a similar procedure described for **37**, **47** was obtained as a white solid; yield: 50% (over two steps). ^{1}H NMR (400 MHz, $CDCl_3$) δ 5.56 (dd, J = 2.0, 4.8 Hz, 1H), 3.67 (s, 3H), 2.43–2.63 (m, 2H), 2.32–2.42 (m, 1H), 2.18–2.28 (m, 1H), 2.06–2.15 (m, 1H), 1.98–2.05 (m, 2H), 1.75–1.94 (m, 2H), 1.65–1.72 (m, 1H), 1.29–1.62 (m, 8H), 1.23 (s, 6H), 1.00–1.20 (m, 5H), 0.93 (d, J = 6.4 Hz, 3H), 0.85 (s, 3H), 0.69 (s, 3H).

By similar procedure described for **47**, **70** was obtained as a white solid; yield: 60% (over two steps). ^{1}H NMR (500 MHz, $CDCl_3$) δ 5.55 (dd, J = 2.4, 5.1 Hz, 1H), 3.67 (s, 3H), 2.41–2.60 (m, 2H), 2.20–2.36 (m, 2H), 2.06–2.14 (m, 1H), 1.98–2.05 (m, 2H), 1.80–1.89 (m, 1H), 1.59–1.74 (m, 3H), 1.46–1.56 (m, 3H), 1.34–1.45 (m, 3H), 1.24–1.30 (m, 2H), 1.23 (s, 6H), 1.15–1.21 (m, 1H), 0.98–1.14 (m, 5H), 0.94 (d, J = 6.5 Hz, 3H), 0.85 (s, 3H), 0.68 (s, 3H).

Synthesis and characterization of compound 48

By a similar procedure described for **24**, **48** and **71** were obtained as white solids.

Compound **48**: yield: 89%. ^{1}H NMR (500 MHz, $CDCl_3$) δ 5.91 (s, 1H), 3.67 (s, 3H), 2.54–2.69 (m, 2H), 2.32–2.45 (m, 2H), 2.19–2.31 (m, 2H), 2.10–2.17 (m, 1H), 2.02–2.08 (m, 1H), 1.91–1.99 (m, 1H), 1.77–1.87 (m, 2H), 1.57–1.65 (m, 2H), 1.49–1.57 (m, 1H), 1.39–1.48 (m, 1H), 1.32 (s, 6H), 1.28–1.39 (m, 4H), 1.14–1.21 (m, 1H), 1.08–1.14 (m, 1H), 1.07 (s, 3H), 0.94 (d, J = 6.5 Hz, 3H), 0.70 (s, 3H).

Characterization of compound 71

Compound **71**: yield: 85%. ^{1}H NMR (500 MHz, $CDCl_3$): δ 5.89 (s, 1 H), 3.66 (s, 3 H), 2.56–2.62 (m, 2 H), 2.32–2.42 (m, 1H), 2.24–2.31 (m, 3H), 2.10–2.14 (m, 1H), 2.00–2.08 (m, 1H), 1.83–1.90 (m, 1H), 1.77–1.82 (m, 1H), 1.60–1.75 (m, 2H), 1.45–1.55 (m, 2H), 1.32–1.42 (m, 3H), 1.30 (s, 6H), 1.24–1.29 (m, 3H), 1.06–1.17 (m, 3H), 1.06 (s, 3 H), 0.94 (d, J = 7.0 Hz, 3 H), 0.68 (s, 3 H).

Synthesis and characterization of compound 49

Compound **49**: To a solution of compound **48** (393 mg, 0.91 mmol) in dry MeOH (40 mL) was added $NaBH_4$ (138 mg, 3.6 mmol). The reaction mixture was stirred 12 h at room temperature and then concentrated. The residue was poured into water (20 mL) and extracted with AcOEt (30 mL×3). The combined organic extract was washed with brine, dried over anhydrous Na_2SO_4 and concentrated. The residue was purified by silica gel chromatography (petroleum ether/AcOEt, 3/1 v/v) to give compound **49** (316 mg, 80%) as a white solid. ^1H NMR (500 MHz, $CDCl_3$) δ 5.52 (d, J = 3.0 Hz, 1H), 3.90 (dd, J = 2.9, 7.9 Hz, 1H), 3.67 (s, 3H), 3.26 (dd, J = 4.7, 10.7 Hz, 1H), 2.31–2.41 (m, 1H), 2.18–2.27 (m, 1H), 1.97–2.03 (m, 1H), 1.88–1.96 (m, 1H), 1.70–1.86 (m, 5H), 1.30–1.53 (m, 10H), 1.18 (s, 3H), 1.14 (s, 3H), 1.08–1.12 (m, 2H), 1.10 (s, 3H), 0.93 (d, J = 6.5 Hz, 3H), 0.69 (s, 3H). ^{13}C NMR (125 MHz, $CDCl_3$) δ 174.71, 153.31, 123.65, 77.09, 73.97, 56.18, 55.09, 51.47, 49.78, 42.70, 41.45, 40.28, 39.41, 36.85, 36.24, 35.28, 31.02, 30.99, 28.36, 27.25, 27.06, 26.08, 23.31, 21.31, 20.65, 18.30, 11.75. HRMS (ESI): calcd for $C_{27}H_{44}NaO_4$ $[M+Na]^+$, 455.3132, found 455.3170.

Synthesis and characterization of compound 72

By a similar procedure described for **49**, **72** was obtained as a white solid; yield: 81%. ^1H NMR (500 MHz, $CDCl_3$) δ 5.52 (d, J = 2.8 Hz, 1H), 3.90 (dd, J = 2.5, 7.6 Hz, 1H), 3.67 (s, 3H), 3.26 (dd, J = 4.7, 11.2 Hz, 1H), 2.20–2.35 (m, 2H), 1.96–2.04 (m, 1H), 1.84–1.93 (m, 1H), 1.66–1.84 (m, 5H), 1.25–1.54 (m, 11H), 1.18 (s, 3H), 1.14 (s, 3H), 1.10 (s, 3H), 1.08–1.13 (m, 3H), 0.94 (d, J = 6.5 Hz, 3H), 0.69 (s, 3H). ^{13}C NMR (125 MHz, $CDCl_3$) δ 174.32, 153.30, 123.66, 77.07, 73.98, 56.20, 55.08, 51.43, 49.81, 42.66, 41.45, 40.27, 39.41, 36.85, 36.24, 35.41, 35.38, 34.47, 28.42, 27.25, 27.07, 26.10, 23.32, 21.47, 21.31, 20.65, 18.60, 11.73. HRMS (ESI): calcd for $C_{28}H_{46}NaO_4$ $[M+Na]^+$, 469.3288, found 469.3274.

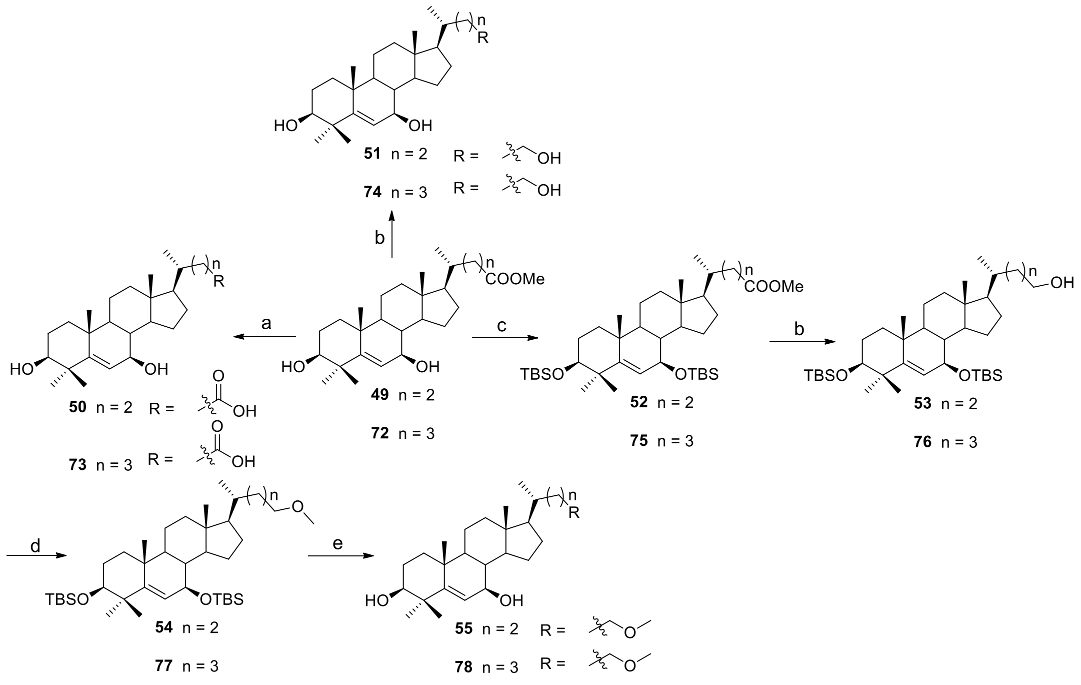

Supplementary Figure 134. The synthesis scheme of compounds 50, 51, 55, 73, 74 and 78. Reagents and conditions: (a) 4 M NaOH, MeOH, r.t., 12 h, 95% for **50** and 93% for **73**; (b) $LiAlH_4$, THF, r.t., 12 h, 94% for **51** , 92% for **74**, 92% for **53** and 93% for **76**; (c) TBSCl, Imidazole, DMF, 80°C, 3 h, 94% for **52** and 92% for **75**; (d) CH_3I, 60% NaH, THF, r.t., 12 h, 91% for **54** and 92% for **77**; (e) TBAF, THF, reflux, 24 h, 90% for **55** and 92% for **78**.

Synthesis and characterization of compound 50

Compound **50**: To a solution of compound **49** (56 mg, 0.13 mmol) in MeOH (10 mL) was added 4 M NaOH (2 mL). The reaction mixture was stirred 12 h at room temperature and then concentrated. H_2O (20 mL) was added to the residue and adjusted the PH value to 3 with 1 M HCl. The mixture was extracted with AcOEt (10 mL×3). The combined organic extract was washed with H_2O and brine, dried over anhydrous Na_2SO_4 and concentrated. The residue was purified by silica gel chromatography (CH_2Cl_2/MeOH, 20/1 v/v) to give compound **50** (52 mg, 95%) as a white solid. ^{1}H NMR (500 MHz, DMSO-d_6) δ 11.94 (brs, 1H), 5.36 (d, J = 2.7 Hz, 1H), 4.50–4.56 (m, 1H), 4.15–4.20 (m, 1H), 3.58–3.64 (m, 1H), 2.97–3.04 (m, 1H), 2.17–2.27 (m, 1H), 2.04–2.14 (m, 1H), 1.89–1.96 (m, 1H), 1.72–1.81 (m, 2H), 1.57–1.71 (m, 4H), 1.50–1.56 (m, 1H), 1.38–1.46 (m, 2H), 1.28–1.38 (m, 6H),

1.14–1.28 (m, 3H), 1.06 (s, 6H), 0.97 (s, 3H), 0.87 (d, J = 6.5 Hz, 3H), 0.62 (s, 3H). ^{13}C NMR (100 MHz, DMSO-d_6) δ 174.89, 150.89, 125.02, 75.20, 72.03, 56.21, 54.82, 49.54, 42.12, 40.97, 39.05, 36.23, 35.99, 34.83, 30.73, 28.98, 27.92, 27.26, 27.08, 25.80, 23.65, 22.06, 20.83, 20.32, 18.20, 11.63. HRMS (ESI): calcd for $C_{26}H_{42}NaO_4$ [M+Na]$^+$, 441.2975, found 441.2999.

Synthesis and characterization of compound 73

By a similar procedure described for **50**, **73** was obtained as a white solid; yield: 93%. ^{1}H NMR (500 MHz, DMSO-d_6) δ 11.94 (brs, 1H), 5.36 (d, J = 2.8 Hz, 1H), 4.47–4.55 (m, 1H), 4.13–4.18 (m, 1H), 3.57–3.66 (m, 1H), 2.96–3.06 (m, 1H), 2.08–2.23 (m, 2H), 1.89–1.96 (m, 1H), 1.71–1.82 (m, 2H), 1.50–1.68 (m, 4H), 1.26–1.46 (m, 8H), 1.15–1.25 (m, 1H), 0.99–1.12 (m, 5H), 1.06 (s, 3H), 1.05 (s, 3H), 0.98 (s, 3H), 0.89 (d, J = 6.4 Hz, 3H), 0.63 (s, 3H). ^{13}C NMR (125 MHz, DMSO-d_6) δ 174.50, 150.91, 125.07, 75.22, 72.07, 56.28, 54.89, 49.59, 48.60, 43.24, 42.13, 41.01, 36.27, 36.03, 35.02, 34.94, 34.08, 28.06, 27.30, 27.13, 25.83, 23.69, 21.06, 20.88, 20.37, 18.55, 11.65. HRMS (ESI): calcd for $C_{27}H_{44}NaO_4$ [M+Na]$^+$, 455.3132, found 455.3172.

Synthesis and characterization of compound 51

By a similar procedure described for **39**, **51** and **74** were obtained as white solids.

Compound **51**: yield: 94%. ^{1}H NMR (500 MHz, DMSO-d_6) δ 5.37 (d, J = 2.3 Hz, 1H), 4.40–4.48 (m, 1H), 4.24 (t, J = 5.1 Hz, 1H), 4.05–4.10 (m, 1H), 3.59–3.65 (m, 1H), 3.31–3.39 (m, 2H), 2.98–3.05 (m, 1H), 1.90–1.98 (m, 1H), 1.72–1.82 (m, 2H), 1.58–1.66 (m, 2H), 1.51–1.57 (m, 1H), 1.15–1.50 (m, 12H), 1.04 (s, 6H), 1.00–1.04 (m, 3H), 0.98 (s, 3H), 0.89 (d, J = 6.4 Hz, 3H), 0.64 (s, 3H). ^{13}C NMR (125 MHz, DMSO-d_6) δ 150.79, 124.93, 75.13, 71.96, 61.19, 56.18, 55.03, 49.53, 42.01, 40.88, 39.08, 36.16, 35.93, 34.97, 31.72, 29.08, 28.83, 27.93, 27.16, 27.01, 25.68, 23.51, 20.75, 20.25, 18.54, 11.55. HRMS (ESI): calcd for $C_{26}H_{44}NaO_3$ [M+Na]$^+$, 427.3183, found 427.3181.

Characterization of compound 74

Compound **74**: yield: 92%. ^{1}H NMR (500 MHz, DMSO-d_6) δ 5.36 (d, J = 2.8 Hz, 1H), 4.49–4.54 (m, 1H), 4.32 (t, J = 5.1 Hz, 1H), 4.14–4.18 (m, 1H), 3.58–3.64 (m, 1H),

3.34–3.39 (m, 2H), 2.96–3.04 (m, 1H), 1.89–1.96 (m, 1H), 1.72–1.81 (m, 2H), 1.57–1.67 (m, 2H), 1.49–1.57 (m, 1H), 1.25–1.45 (m, 10H), 1.07–1.24 (m, 4H), 1.06 (s, 6H), 0.99–1.03 (m, 3H), 0.97 (s, 3H), 0.89 (d, *J* = 6.4 Hz, 3H), 0.63 (s, 3H). ^{13}C NMR (125 MHz, DMSO-d_6): *δ* 150.81, 124.97, 75.14, 71.98, 60.69, 56.22, 54.97, 49.53, 42.05, 40.92, 39.14, 38.97, 36.18, 35.95, 35.34, 35.14, 32.97, 28.02, 27.20, 27.05, 25.73, 23.57, 21.87, 20.78, 20.28, 18.53, 11.57. HRMS (ESI): calcd for $C_{27}H_{46}NaO_3$ [M+Na]$^+$, 441.3339, found 441.3333.

Synthesis and characterization of compound 52

By a similar procedure described for **38**, **52** and **75** were obtained as white solids.

Compound **52**: yield: 94%. ^{1}H NMR (500 MHz, $CDCl_3$) *δ* 5.49 (d, *J* = 3.2 Hz, 1H), 3.99 (dd, *J* = 3.2, 7.5 Hz, 1H), 3.66 (s, 3H), 3.21 (dd, *J* = 4.1, 11.4 Hz, 1H), 2.30–2.39 (m, 1H), 2.17–2.27 (m, 1H), 1.93–1.99 (m, 1H), 1.70–1.89 (m, 4H), 1.62–1.69 (m, 1H), 1.24–1.56 (m, 10H), 1.12 (s, 3H), 1.07 (s, 3H), 1.05–1.09 (m, 3H), 1.05 (s, 3H), 0.85–0.93 (m, 3H), 0.89 (s, 9H), 0.88 (s, 9H), 0.67 (s, 3H), 0.08 (s, 3H), 0.07 (s, 3H), 0.04 (s, 3H), 0.02 (s, 3H).

Characterization of compound 75

Compound **75**: yield: 92%. ^{1}H NMR (500 MHz, $CDCl_3$) *δ* 5.49 (d, *J* = 3.1 Hz, 1H), 3.99 (dd, *J* = 3.1, 7.5 Hz, 1H), 3.67 (s, 3H), 3.21 (dd, *J* = 4.1, 11.5 Hz, 1H), 2.20-2.33 (m, 2H), 1.93–2.00 (m, 1H), 1.77–1.86 (m, 1H), 1.63–1.77 (m, 4H), 1.43–1.58 (m, 4H), 1.31–1.43 (m, 5H), 1.20–1.29 (m, 2H), 1.12 (s, 3H), 1.08 (s, 3H), 1.05 (s, 3H), 1.07–0.98 (m, 4H), 0.93 (d, *J* = 6.5 Hz, 3H), 0.89 (s, 9H), 0.88 (s, 9H), 0.67 (s, 3H), 0.08 (s, 3H), 0.07 (s, 3H), 0.04 (s, 3H), 0.02 (s, 3H).

Synthesis and characterization of compound 53

By a similar procedure described for **39**, **53** and **76** were obtained as white solids.

Compound **53**: yield: 92%. ^{1}H NMR (500 MHz, $CDCl_3$) *δ* 5.49 (d, *J* = 3.2 Hz, 1H), 4.00 (dd, *J* = 3.1, 7.6 Hz, 1H), 3.57–3.66 (m, 2H), 3.21 (dd, *J* = 4.1, 11.5 Hz, 1H), 1.95–2.01 (m, 1H), 1.78–1.88 (m, 1H), 1.70–1.78 (m, 2H), 1.61–1.69 (m, 2H), 1.49–1.57 (m, 2H), 1.20–1.49 (m, 10H), 1.12 (s, 3H), 1.07 (s, 3H), 1.11–1.06 (m, 3H),

1.05 (s, 3H), 0.93 (d, J = 6.5 Hz, 3H), 0.89 (s, 9H), 0.88 (s, 9H), 0.68 (s, 3H), 0.08 (s, 3H), 0.07 (s, 3H), 0.04 (s, 3H), 0.02 (s, 3H).

Characterization of compound 76

Compound **76**: yield: 93%. ^{1}H NMR (500 MHz, $CDCl_3$) δ 5.49 (d, J = 2.4 Hz, 1H), 3.99 (dd, J = 2.5, 7.4 Hz, 1H), 3.60–3.68 (m, 2H), 3.21 (dd, J = 3.6, 11.2 Hz, 1H), 1.94–2.01 (m, 1H), 1.78–1.87 (m, 1H), 1.69–1.78 (m, 2H), 1.62–1.69 (m, 1H), 1.31–1.56 (m, 10H), 1.19–1.30 (m, 4H), 1.12 (s, 3H), 1.08 (s, 3H), 1.05 (s, 3H), 1.02–1.09 (m, 4H), 0.92 (d, J = 6.5 Hz, 3H), 0.89 (s, 9H), 0.88 (s, 9H), 0.67 (s, 3H), 0.08 (s, 3H), 0.07 (s, 3H), 0.04 (s, 3H), 0.02 (s, 3H). HRMS (ESI): calcd for $C_{39}H_{74}NaO_3Si_2$ $[M+Na]^+$, 669.5069, found 669.5078.

Synthesis and characterization of compound 54

Compound **54**: To a solution of compound **53** (100 mg, 0.16 mmol) in dry THF (10 mL) was added 60% NaH (64 mg, 1.6 mmol) under N_2 at 0ºC. After stirring for 0.5 h, CH_3I (0.1 mL, 1.6 mmol) was added and the reaction mixture was stirred for 12 h at room temperature. The reaction mixture was concentrated and H_2O (20 mL) was added, then the aqueous phase was extracted with AcOEt (20 mL× 3). The combined organic extract was washed with brine, dried over anhydrous Na_2SO_4 and concentrated. The residue was purified by silica gel chromatography (petroleum ether/AcOEt, 20/1 v/v) to give compound **54** (94 mg, 91%) as a white solid. ^{1}H NMR (500 MHz, $CDCl_3$) δ 5.49 (d, J = 3.1 Hz, 1H), 4.00 (dd, J = 3.1, 7.5 Hz, 1H), 3.36 (t, J = 6.7 Hz, 2H), 3.33 (s, 3H), 3.22 (dd, J = 4.0, 11.5 Hz, 1H), 1.96–1.99 (m, 1H), 1.83–1.93 (m, 1H), 1.70–1.82 (m, 2H), 1.64–1.67 (m, 2H), 1.52–1.58 (m, 2H), 1.34–1.48 (m, 7H), 1.24–1.29 (m, 2H), 1.02–1.11 (m, 4H), 1.12 (s, 3H), 1.08 (s, 3H), 1.05 (s, 3H), 0.93 (d, J = 6.5 Hz, 3H), 0.90 (s, 9H), 0.88 (s, 9H), 0.68 (s, 3H), 0.09 (s, 3H), 0.08 (s, 3H), 0.04 (s, 3H), 0.02 (s, 3H).

Synthesis and characterization of compound 77

By a similar procedure described for **54**, **77** was obtained as a white solid; yield: 92%. ^{1}H NMR (500 MHz, $CDCl_3$) δ 5.49 (d, J = 3.2 Hz, 1H), 3.99 (dd, J = 3.2, 7.5 Hz, 1H), 3.37 (t, J = 6.6 Hz, 2H), 3.34 (s, 3H), 3.21 (dd, J = 4.2, 11.6 Hz, 1H), 1.94–2.01 (m, 1H), 1.78–1.87 (m, 1H), 1.69–1.78 (m, 2H), 1.63–1.69 (m, 1H), 1.43–1.57 (m, 5H),

1.34–1.42 (m, 5H), 1.16–1.28 (m, 3H), 1.12 (s, 3H), 1.07 (s, 3H), 1.05 (s, 3H), 1.00-1.10 (m, 5H), 0.91 (d, *J* = 6.8 Hz, 3H), 0.89 (s, 9H), 0.88 (s, 9H), 0.66 (s, 3H), 0.08 (s, 3H), 0.07 (s, 3H), 0.04 (s, 3H), 0.02 (s, 3H).

Synthesis and characterization of compound 54

Compound **55**: To a solution of compound **54** (81 mg, 0.126 mmol) in THF (10 mL) was added TBAF (239 mg, 0.756 mmol). The reaction mixture was heated to reflux for 24 h, then concentrated and saturated aqueous NH_4Cl (10 mL) was added. The aqueous phase was extracted with AcOEt (20 mL×3). The combined organic extract was washed with brine, dried over anhydrous Na_2SO_4 and concentrated. The residue was purified by silica gel chromatography (petroleum ether/AcOEt, 3/1 v/v) to give compound **55** (48 mg, 90%) as a white solid. ^{1}H NMR (500 MHz, $CDCl_3$) δ 5.51 (d, *J* = 2.6 Hz, 1H), 3.89 (dd, *J* = 2.5, 7.8 Hz, 1H), 3.34 (t, *J* = 6.7 Hz, 2H), 3.32 (s, 3H), 3.25 (dd, *J* = 4.6, 10.9 Hz, 1H), 1.98–2.01 (m, 1H), 1.83–1.93 (m, 1H), 1.60–1.82 (m, 7H), 1.37–1.59 (m, 10H), 1.21–1.32 (m, 2H), 1.17 (s, 3H), 1.13 (s, 3H), 1.07 (s, 3H), 0.92 (d, *J* = 6.4 Hz, 3H), 0.68 (s, 3H). ^{13}C NMR (125 MHz, $CDCl_3$) δ 153.29, 123.67, 77.07, 73.99, 73.44, 58.48, 56.21, 55.30, 49.82, 42.66, 41.45, 40.28, 39.44, 36.86, 36.26, 35.54, 32.18, 28.47, 27.26, 27.07, 26.16, 26.11, 23.33, 21.31, 20.66, 18.65, 11.76. HRMS (ESI): calcd for $C_{27}H_{46}NaO_3$ $[M+Na]^+$, 441.3339, found 441.3324.

Synthesis and characterization of compound 78

By a similar procedure described for **55**, **78** was obtained as a white solid; yield: 92%. ^{1}H NMR (500 MHz, $CDCl_3$) δ 5.52 (d, *J* = 3.0 Hz, 1H), 3.90 (dd, *J* = 2.6, 7.8 Hz, 1H), 3.37 (t, *J* = 6.6 Hz, 2H), 3.34 (s, 3H), 3.26 (dd, *J* = 4.8, 10.8 Hz, 1H), 1.97–2.04 (m, 1H), 1.84–1.94 (m, 1H), 1.71–1.83 (m, 4H), 1.35–1.61 (m, 12H), 1.18 (s, 3H), 1.14 (s, 3H), 1.10 (s, 3H), 1.04–1.13 (m, 5H), 0.92 (d, *J* = 6.5 Hz, 3H), 0.68 (s, 3H). ^{13}C NMR (125 MHz, $CDCl_3$): δ 153.25, 123.64, 77.03, 73.97, 72.96, 58.49, 56.19, 55.25, 49.79, 42.63, 41.42, 40.25, 39.41, 36.83, 36.23, 35.74, 35.59, 30.05, 28.45, 27.24, 27.05, 26.10, 23.30, 22.54, 21.29, 20.63, 18.64, 11.72. HRMS (ESI): calcd for $C_{28}H_{48}NaO_3$ $[M+Na]^+$, 455.3496, found 455.3486.

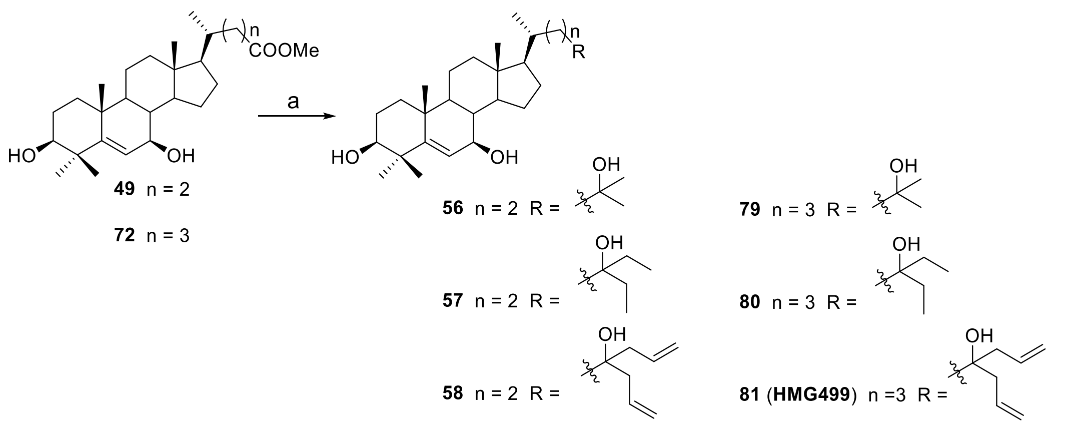

Supplementary Figure 135. The synthesis scheme of compounds 56–58 and 79–81 by Grignard reaction. Reagents and conditions: (a) CH_3MgCl (1.0 M in THF) or C_2H_5MgCl (2.0 M in THF) or C_3H_5MgCl (1.0 M in THF), THF, r.t.,1 h (80 - 89% for **56- 58** and **79- 81**.

General procedure for Grignard Reaction

To a solution of compound **49** or **72** (0.2 mmol) in dry THF (12 mL) was added Grignard reagent (2 mmol, 1 M in THF) under N_2 at 0°C. The reaction mixture was stirred for 1 h at room temperature and then poured into saturated NH_4Cl (20 mL) and extracted with AcOEt (50 mL×3). The combined organic extract was washed with brine, dried over anhydrous Na_2SO_4 and concentrated. The residue was purified by silica gel chromatography (petroleum ether/AcOEt, 1/1 v/v) to give the desired product.

Characterization of compound 56

Compound **56**: white solid; yield: 80%. ^{1}H NMR (500 MHz, DMSO-d_6) δ 5.35 (d, J = 2.3 Hz, 1H), 4.51–4.54 (m, 1H), 4.15–4.19 (m, 1H), 4.02 (s, 1H), 3.57–3.64 (m, 1H), 2.96–3.05 (m, 1H), 1.89–1.95 (m, 1H), 1.72–1.82 (m, 2H), 1.57–1.66 (m, 2H), 1.49–1.57 (m, 1H), 1.36–1.46 (m, 4H), 1.27–1.36 (m, 4H), 1.14–1.25 (m, 3H), 1.02–1.07 (m, 4H), 1.06 (s, 3H), 1.05 (s, 3H), 1.04 (s, 3H), 1.03 (s, 3H), 0.97 (s, 3H), 0.88 (d, J = 6.2 Hz, 3H), 0.62 (s, 3H). ^{13}C NMR (100 MHz, CD_3OD-d_4 + $CDCl_3$) δ 154.09, 125.61, 77.96, 74.53, 71.74, 58.04, 56.74, 51.65, 43.87, 42.61, 41.05, 40.96, 40.75, 38.03, 37.78, 37.36, 31.50, 29.58, 29.52, 29.13, 28.07, 27.94, 27.12, 24.30, 21.99, 21.85, 19.57, 12.53. HRMS (ESI): calcd for $C_{28}H_{48}NaO_3$ [M+Na]$^+$, 455.3496,

found 455.3630.

Characterization of compound 57

Compound **57**: white solid; yield: 85%. ^{1}H NMR (400 MHz, CD_3OD-d_4) δ 5.49 (d, J = 3.2 Hz, 1H), 3.78 (dd, J = 3.0, 8.0 Hz, 1H), 3.16 (dd, J = 4.2, 11.8 Hz, 1H), 2.00–2.05 (m, 1H), 1.84–1.95 (m, 2H), 1.80–1.83 (m, 1H), 1.72–1.78 (m, 2H), 1.62–1.69 (m, 1H), 1.35–1.54 (m, 13H), 1.28–1.33 (m, 5H), 1.16 (s, 3H), 1.14 (s, 3H), 1.08 (s, 3H), 0.96 (d, J = 6.5 Hz, 3H), 0.82–0.87 (m, 6H), 0.72 (s, 3H). ^{13}C NMR (100 MHz, $CDCl_3$ + CD_3OD-d_4) δ 156.61, 128.12, 80.48, 78.23, 77.06, 60.55, 59.28, 54.15, 46.42, 45.15, 43.48, 43.28, 40.57, 40.31, 40.13, 37.61, 34.53, 34.17, 33.08, 32.25, 30.60, 30.50, 29.67, 26.86, 24.54, 24.41, 22.23, 15.09, 10.90, 10.80. HRMS (ESI): calcd for $C_{30}H_{52}NaO_3$ $[M+Na]^+$, 483.3809, found 483.3803.

Characterization of compound 58

Compound **58**: white solid; yield: 87%. ^{1}H NMR (500 MHz, $CDCl_3$) δ 5.80–5.90 (m, 2H), 5.52 (d, J = 3.0 Hz, 1H), 5.08–5.17 (m, 4H), 3.91 (dd, J = 3.0, 7.9 Hz, 1H), 3.26 (dd, J = 4.7, 10.8 Hz, 1H), 2.22 (d, J = 6.7 Hz, 4H), 1.97–2.09 (m, 2H), 1.87–1.97 (m, 1H), 1.71–1.84 (m, 4H), 1.35–1.57 (m, 11H), 1.29–1.34 (m, 3H), 1.18 (s, 3H), 1.14 (s, 3H), 1.10 (s, 3H), 0.93 (d, J = 6.6 Hz, 3H), 0.69 (s, 3H). ^{13}C NMR (125 MHz, $CDCl_3$) δ 153.31, 133.76 (2C), 123.69, 118.59, 118.52, 73.98, 73.62, 56.23, 55.12, 49.83, 43.78, 43.56, 42.66, 41.45, 40.27, 39.43, 36.86, 36.27, 36.02, 35.38, 29.66, 29.24, 28.52, 27.25, 27.09, 26.13, 23.34, 21.30, 20.66, 18.81, 11.74. HRMS (ESI): calcd for $C_{32}H_{52}NaO_3$ $[M+Na]^+$, 507.3809, found 507.3822.

Characterization of compound 79

Compound **79**: white solid; yield: 89%. ^{1}H NMR (500 MHz, DMSO-d_6) δ 5.36 (d, J = 2.6 Hz, 1H), 4.49–4.56 (m, 1H), 4.14–4.20 (m, 1H), 4.05 (s, 1H), 3.53–3.66 (m, 1H), 2.95–3.06 (m, 1H), 1.90–1.97 (m, 1H), 1.71–1.81 (m, 2H), 1.57–1.68 (m, 2H), 1.50–1.57 (m, 1H), 1.10–1.45 (m, 13H), 1.05 (s, 12H), 0.94–1.02 (m, 4H), 0.98 (s, 3H), 0.90 (d, J = 6.3 Hz, 3H), 0.63 (s, 3H). ^{13}C NMR (125 MHz, DMSO-d_6) δ 150.89, 125.05, 75.21, 72.06, 68.78, 56.30, 55.17, 49.60, 47.83, 44.16, 42.14, 41.00, 39.17, 36.29, 36.26, 36.04, 35.33, 29.39, 29.21, 28.17, 27.28, 27.12, 25.83, 23.68, 20.86, 20.38, 20.36, 18.64, 11.67. HRMS (ESI): calcd for $C_{29}H_{50}NaO_3$ $[M+Na]^+$, 469.3652,

found 469.3657.

Characterization of compound 80

Compound **80**: white solid; yield: 87%. ^{1}H NMR (500 MHz, $CDCl_3$) δ 5.52 (d, J = 2.8 Hz, 1H), 3.90 (dd, J = 2.4, 7.8 Hz,1H), 3.26 (dd, J = 4.3, 10.4 Hz, 1H), 1.98–2.04 (m, 1H), 1.84–1.93 (m, 1H), 1.71–1.83 (m, 4H), 1.44–1.49 (m, 7H), 1.28–1.43 (m, 11H), 1.18 (s, 3H), 1.14 (s, 3H), 1.10 (s, 3H), 1.01–1.14 (m, 3H), 0.93 (d, J = 6.5 Hz, 3H), 0.84-0.88 (m, 6H), 0.69 (s, 3H). ^{13}C NMR (125 MHz, DMSO-d_6) δ 150.82, 125.02, 75.15, 72.37, 72.01, 56.23, 55.03, 49.53, 42.07, 40.94, 39.11, 38.30, 36.34, 36.21, 35.97, 35.17, 30.58, 30.45, 28.09, 28.07, 27.23, 27.07, 25.78, 23.61, 20.81, 20.30, 19.26, 18.61, 11.61, 7.79, 7.75. HRMS (ESI): calcd for $C_{31}H_{54}NaO_3$ $[M+Na]^+$, 497.3965, found 497.3948.

Characterization of compound 81

Compound **81** (**HMG499**): white solid; yield: 81%. ^{1}H NMR (500 MHz, $CDCl_3$) δ 5.80-5.90 (m, 2H), 5.52 (d, J = 3.0 Hz, 1H), 5.08–5.18 (m, 4H), 3.91 (dd, J = 2.5, 7.7 Hz, 1H), 3.26 (dd, J = 4.7, 10.7 Hz, 1H), 2.18–2.28 (m, 4H), 1.98–2.04 (m, 1H), 1.83–1.92 (m, 1H), 1.70–1.83 (m, 4H), 1.23–1.55 (m, 17H), 1.18 (s, 3H), 1.14 (s, 3H), 1.10 (s, 3H), 0.93 (d, J = 6.5 Hz, 3H), 0.69 (s, 3H). ^{13}C NMR (125 MHz, $CDCl_3$) δ 153.30, 133.80 (2C), 123.65, 118.55 (2C), 77.08, 74.01, 73.52, 56.19, 55.31, 49.81, 43.77, 43.65, 42.67, 41.44, 40.29, 39.61, 39.43, 36.86, 36.41, 36.24, 35.59, 28.49, 27.26, 27.06, 26.12, 23.31, 21.31, 20.65, 19.69, 18.72, 11.75. HRMS (ESI): calcd for $C_{33}H_{54}NaO_3$ $[M+Na]^+$, 521.3965, found 521.3993.

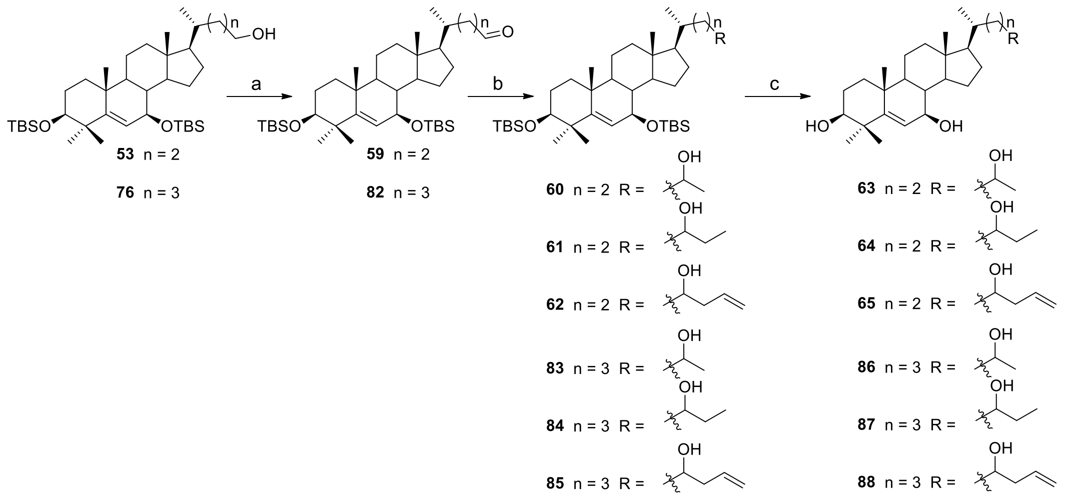

Supplementary Figure 136. The synthesis scheme of compounds 63–65 and 86–88. Reagents and conditions: (a) IBX, DMSO, 50°C, 3 h, 89% for **59** and 87% for **82**; (b) CH_3MgCl (1.0 M in THF) or C_2H_5MgCl (2.0 M in THF) or C_3H_5MgCl (1.0 M in THF), THF, r.t., 2 h, 60- 83.2% for **60- 62** and **83- 85**; (c) TBAF, THF, reflux, 24 h, 70- 90% for **63- 65** and **86- 88**.

Synthesis and characterization of compound 59

Compound **59**: To a solution of compound **53** (2.4 g, 3.75 mmol) in DMSO (50 mL) was added IBX (2.1 g, 7.5 mmol). The reaction mixture was stirred for 3 h at 50 °C. After cooling to temperature, H_2O (100 mL) and AcOEt (50 mL) was added. After separation of the organic layer, the aqueous phase was extracted with AcOEt (50 mL×3). The combined organic extract was washed with brine, dried over anhydrous Na_2SO_4 and concentrated. The residue was purified by silica gel chromatography (petroleum ether/AcOEt, 25/1 v/v) to give compound **59** (2.1 g, 89%) as a white solid. ^{1}H NMR (500 MHz, $CDCl_3$) δ 9.76 (s, 1H), 5.49 (d, J = 3.0 Hz, 1H), 4.00 (dd, J = 3.0, 7.4 Hz, 1H), 3.21 (dd, J = 4.1, 11.5 Hz, 1H), 2.40–2.50 (m, 1H), 2.30–2.39 (m, 1H), 1.93–2.01 (m, 1H), 1.70–1.90 (m, 4H), 1.62–1.68 (m, 1H), 1.24–1.55 (m, 9H), 1.12 (s, 3H), 1.08 (s, 3H), 1.06–1.11 (m, 2H), 1.05 (s, 3H), 1.02–1.06 (m, 2H), 0.92 (d, J = 6.6 Hz, 3H), 0.90 (s, 9H), 0.88 (s, 9H), 0.68 (s, 3H), 0.08 (s, 3H), 0.07 (s, 3H), 0.04 (s, 3H), 0.02 (s, 3H).

Synthesis and characterization of compound 82

By a similar procedure described for **59**, **82** was obtained as a white solid; yield: 87%. ^{1}H NMR (500 MHz, $CDCl_3$) δ 9.76 (s, 1H), 5.49 (d, J = 3.2 Hz, 1H), 3.99 (dd, J = 3.1, 7.5 Hz, 1H), 3.21 (dd, J = 4.2, 11.5 Hz, 1H), 2.32–2.46 (m, 2H), 1.92–2.01 (m, 1H), 1.78–1.86 (m, 1H), 1.69–1.78 (m, 3H), 1.62–1.68 (m, 1H), 1.44–1.56 (m, 4H), 1.33–1.43 (m, 4H), 1.19–1.30 (m, 2H), 1.12 (s, 3H), 1.08 (s, 3H), 1.05 (s, 3H), 0.99–1.10 (m, 5H), 0.94 (d, J = 6.5 Hz, 3H), 0.89 (s, 9H), 0.88 (s, 9H), 0.67 (s, 3H), 0.08 (s, 3H), 0.07 (s, 3H), 0.04 (s, 3H), 0.02 (s, 3H).

Characterization of compound 60

Compound **60**: white solid; yield: 60%. ^{1}H NMR (500 MHz, $CDCl_3$) δ 5.49 (d, J = 3.1 Hz, 1H), 4.00 (dd, J = 3.0, 7.6 Hz, 1H), 3.70–3.81 (m, 1H), 3.21 (dd, J = 4.2, 11.5 Hz,

1H), 1.94–2.00 (m, 1H), 1.78–1.90 (m, 1H), 1.70–1.78 (m, 2H), 1.63–1.69 (m, 1H), 1.47–1.55 (m, 3H), 1.28–1.47 (m, 10H), 1.19 (d, J = 6.2 Hz, 3H), 1.12 (s, 3H), 1.08 (s, 3H), 1.05 (s, 3H), 1.03–1.10 (m, 3H), 0.92 (d, J = 6.8 Hz, 3H), 0.89 (s, 9H), 0.88 (s, 9H), 0.68 (s, 3H), 0.08 (s, 3H), 0.07 (s, 3H), 0.04 (s, 3H), 0.02 (s, 3H).

Characterization of compound 61

Compound **61**: white solid; yield: 75%. ^{1}H NMR (500 MHz, $CDCl_3$) δ 5.49 (d, J = 3.1 Hz, 1H), 4.00 (dd, J = 3.0, 7.5 Hz, 1H), 3.42–3.52 (m, 1H), 3.21 (dd, J = 4.0, 11.4 Hz, 1H), 1.94–2.02 (m, 1H), 1.78–1.90 (m, 1H), 1.70–1.78 (m, 2H), 1.63–1.69 (m, 1H), 1.49–1.58 (m, 5H), 1.34–1.49 (m, 7H), 1.28–1.34 (m, 3H), 1.12 (s, 3H), 1.08 (s, 3H), 1.05 (s, 3H), 0.99-1.06 (m, 3H), 0.91-0.96 (m, 6H), 0.89 (s, 9H), 0.88 (s, 9H), 0.67 (s, 3H), 0.08 (s, 3H), 0.07 (s, 3H), 0.04 (s, 3H), 0.02 (s, 3H).

Characterization of compound 62

Compound **62**: white solid; yield: 83%. ^{1}H NMR (400 MHz, $CDCl_3$) δ 5.70–5.92 (m, 1H), 5.49 (d, J = 3.6 Hz, 1H), 5.04–5.25 (m, 2H), 4.00 (dd, J = 3.5, 9.2 Hz, 1H), 3.49–3.67 (m, 1H), 3.21 (dd, J = 4.8, 14.2 Hz, 1H), 2.25–2.35 (m, 1H), 2.10–2.19 (m, 1H), 1.93–2.00 (m, 1H), 1.28–1.89 (m, 15H), 1.12 (s, 3H), 1.08 (s, 3H), 1.05 (s, 3H), 1.00–1.10 (m, 5H), 0.83–0.94 (m, 3H), 0.89 (s, 9H), 0.88 (s, 9H), 0.67 (s, 3H), 0.08 (s, 3H), 0.07 (s, 3H), 0.04 (s, 3H), 0.02 (s, 3H).

Characterization of compound 83

Compound **83**: white solid; yield: 75%. ^{1}H NMR (500 MHz, $CDCl_3$) δ 5.49 (d, J = 3.0 Hz, 1H), 4.00 (dd, J = 3.2, 7.7 Hz, 1H), 3.84–3.74 (m, 1H), 3.21 (dd, J = 4.1, 11.5 Hz, 1H), 1.93–2.02 (m, 1H), 1.78–1.88 (m, 1H), 1.69–1.78 (m, 2H), 1.62–1.69 (m, 1H), 1.51–1.55 (m, 1H), 1.31–1.50 (m, 8H), 1.24–1.31 (m, 3H), 1.19 (d, J = 5.6 Hz, 3H), 1.12 (s, 3H), 1.08 (s, 3H), 1.05 (s, 3H), 1.02–1.08 (m, 6H), 0.88–0.92 (m, 3H), 0.89 (s, 9H), 0.88 (s, 9H), 0.67 (s, 3H), 0.08 (s, 3H), 0.07 (s, 3H), 0.04 (s, 3H), 0.02 (s, 3H).

Characterization of compound 84

Compound **84**: white solid; yield: 78%. ^{1}H NMR (500 MHz, $CDCl_3$) δ 5.49 (d, J = 3.1 Hz, 1H), 3.99 (dd, J = 3.1, 7.5 Hz, 1H), 3.46–3.58 (m, 1H), 3.21 (dd, J = 4.1, 11.5 Hz, 1H), 1.94–2.02 (m, 1H), 1.78–1.87 (m, 1H), 1.69–1.77 (m, 2H), 1.62–1.68 (m, 1H),

1.22–1.58 (m, 19H), 1.12 (s, 3H), 1.07 (s, 3H), 1.05 (s, 3H), 0.87–1.02 (m, 7H), 0.89 (s, 9H), 0.88 (s, 9H), 0.67 (s, 3H), 0.08 (s, 3H), 0.07 (s, 3H), 0.04 (s, 3H), 0.02 (s, 3H).

Characterization of compound 85

Compound **85**: white solid; yield: 79%. ^{1}H NMR (500 MHz, $CDCl_3$) δ 5.77–5.89 (m, 1H), 5.49 (d, J = 3.0 Hz, 1H), 5.08-5.20 (m, 2H), 4.00 (dd, J = 2.9, 7.5 Hz, 1H), 3.59–3.77 (m, 1H), 3.21 (dd, J = 4.0, 11.5 Hz, 1H), 2.24–2.35 (m, 1H), 2.09–2.19 (m, 1H), 1.93–2.02 (m, 1H), 1.78–1.87 (m, 1H), 1.69–1.78 (m, 2H), 1.62–1.69 (m, 1H), 1.50–1.55 (m, 2H), 1.31–1.49 (m, 9H), 1.23–1.30 (m, 2H), 1.12 (s, 3H), 1.05 (s, 3H), 1.02 (s, 3H), 0.97–1.08 (m, 5H), 0.92 (d, J = 6.4 Hz, 3H), 0.89 (s, 9H), 0.88 (s, 9H), 0.67 (s, 3H), 0.08 (s, 3H), 0.07 (s, 3H), 0.04 (s, 3H), 0.03 (s, 3H).

Synthesis and characterization of compound 63

By a similar procedure described for **55**, **63–65** and **86–88** were obtained as white solids. Compound **63**: yield: 75%. ^{1}H NMR (500 MHz, $CDCl_3$) δ 5.52 (d, J = 2.7 Hz, 1H), 3.91 (dd, J = 2.4, 7.0 Hz, 1H), 3.70–3.80 (m, 1H), 3.26 (dd, J = 4.7, 10.6 Hz, 1H), 1.98–2.04 (m, 1H), 1.85–1.96 (m, 1H), 1.69–1.85 (m, 5H), 1.30–1.52 (m, 14H), 1.20 (d, J = 2.7 Hz, 3H), 1.18 (s, 3H), 1.14 (s, 3H), 1.10 (s, 3H), 0.94 (d, J = 6.5 Hz, 3H), 0.69 (s, 3H). ^{13}C NMR (125 MHz, DMSO-d_6) δ 150.86, 125.05, 75.19, 72.04, 66.38, 56.25, 54.92, 49.56, 42.07, 40.98, 36.24, 35.99, 35.48, 35.10, 31.68, 31.52, 28.04, 27.27, 27.10, 25.80, 23.78, 23.66, 23.52, 20.85, 20.32, 18.69, 11.65. HRMS (ESI): calcd for $C_{27}H_{46}NaO_3$ $[M+Na]^+$, 441.3339, found 441.3336.

Characterization of compound 64

Compound **64**: yield: 80%. ^{1}H NMR (500 MHz, $CDCl_3$): δ 5.51 (d, J = 3.0 Hz, 1 H), 3.90 (m, 1 H), 3.46–3.49 (m, 1 H), 3.25 (dd, J = 4.5, 11.10 Hz, 1 H), 1.99–2.02 (m, 1 H), 1.86–1.96 (m, 1 H), 1.72–1.81 (m, 4 H), 1.30–1.54 (m, 17 H), 1.18 (s, 3 H), 1.14 (s, 3 H), 1.10 (s, 3 H), 0.91-0.96 (m, 6 H), 0.69 (s, 3 H). ^{13}C NMR (125 MHz, $CDCl_3$) δ 153.30, 123.66, 77.07, 73.99, 73.88, 56.21, 55.22, 49.82, 42.66, 41.44, 40.28, 39.44, 36.86, 36.25, 35.75, 33.34, 31.80, 30.22, 28.51, 27.25, 27.06, 26.11, 23.32, 21.30, 20.66, 18.77, 11.76, 9.76. HRMS (ESI): calcd for $C_{28}H_{48}NaO_3$ $[M+Na]^+$, 455.3496, found 455.3489.

Characterization of compound 65

Compound **65**: yield: 88%. ^{1}H NMR (500 MHz, $CDCl_3$) δ 5.78-5.89 (m, 1H), 5.52 (d, *J* = 3.0 Hz, 1H), 5.14 (d, *J* = 15.3 Hz, 2H), 3.91 (dd, *J* = 2.5, 7.9 Hz, 1H), 3.57–3.65 (m, 1H), 3.26 (dd, *J* = 4.7, 10.6 Hz, 1H), 2.27–2.36 (m, 1H), 2.09–2.19 (m, 1H), 1.98–2.05 (m, 1H), 1.86–1.95 (m, 1H), 1.71–1.84 (m, 4H), 1.52–1.58 (m, 2H), 1.39–1.52 (m, 8H), 1.18 (s, 3H), 1.14 (s, 3H), 1.10 (s, 3H), 1.06-1.15 (m, 5H), 0.94 (d, *J* = 6.5 Hz, 3H), 0.69 (s, 3H). ^{13}C NMR (125 MHz, $CDCl_3$) δ 153.33, 134.90, 123.65, 118.09, 77.10, 74.01, 71.26, 56.21, 55.21, 49.82, 42.67, 42.03, 41.71, 41.45, 40.30, 39.44, 36.87, 36.25, 35.72, 29.66, 28.47, 27.26, 27.07, 26.12, 23.32, 21.32, 20.66, 18.76, 11.76. HRMS (ESI): calcd for $C_{29}H_{48}NaO_3$ $[M+Na]^+$, 467.3496, found 467.3438.

Characterization of compound 86

Compound **86**: yield: 70%. ^{1}H NMR (500 MHz, $CDCl_3$) δ 5.52 (d, *J* = 2.9 Hz, 1H), 3.90 (dd, *J* = 7.6, 2.5 Hz, 1H), 3.75-3.83 (m, 1H), 3.26 (dd, *J* = 10.8, 4.5 Hz, 1H), 1.98-2.05 (m, 1H), 1.84–1.94 (m, 1H), 1.70–1.84 (m, 4H), 1.24–1.52 (m, 17H), 1.17–1.21 (m, 6H), 1.14 (s, 3H), 1.10 (s, 3H), 0.93 (d, *J* = 6.5 Hz, 3H), 0.69 (s, 3H). ^{13}C NMR (125 MHz, $CDCl_3$) δ 153.33, 123.63, 77.10, 74.02, 68.14, 56.20, 55.30, 49.82, 42.67, 41.45, 40.30, 39.83, 39.73, 39.44, 36.86, 36.24, 35.87, 35.62, 28.50, 27.25, 27.06, 26.12, 23.47, 23.31, 21.32, 20.65, 18.67, 11.75. HRMS (ESI): calcd for $C_{28}H_{48}NaO_3$ $[M+Na]^+$, 455.3496, found 455.3516.

Characterization of compound 87

Compound **87**: yield: 73%. ^{1}H NMR (500 MHz, $CDCl_3$) δ 5.52 (d, *J* = 2.5 Hz, 1H), 3.90 (dd, *J* = 2.5, 7.4 Hz, 1H), 3.57–3.47 (m, 1H), 3.26 (dd, *J* = 4.1, 10.6 Hz, 1H), 1.98–2.03 (m, 1H), 1.84–1.93 (m, 1H), 1.69–1.84 (m, 4H), 1.26–1.54 (m, 19H), 1.18 (s, 3H), 1.14 (s, 3H), 1.10 (s, 3H), 0.91–0.97 (m, 6H), 0.69 (s, 3H). ^{13}C NMR (125 MHz, $CDCl_3$) δ 153.33, 123.65, 77.12, 74.04, 73.32, 56.22, 55.34, 49.83, 42.68, 41.46, 40.32, 39.45, 37.48, 37.34, 36.88, 36.26, 36.05, 35.94, 35.63, 28.51, 27.28, 27.07, 26.14, 23.31, 21.33, 20.67, 18.69, 11.76, 9.90. HRMS (ESI): calcd for $C_{29}H_{50}NaO_3$ $[M+Na]^+$, 469.3652, found 469.3663.

Characterization of compound 88

Compound **88**: yield: 80%. ^{1}H NMR (500 MHz, $CDCl_3$) δ 5.78–5.90 (m, 1H), 5.52 (d, J = 2.4 Hz 1H), 5.14 (d, J = 13.3 Hz, 2H), 3.90 (dd, J = 1.2, 4.2 Hz, 1H), 3.60–3.69 (m, 1H), 3.26 (dd, J = 3.1, 9.2 Hz, 1H), 2.26–2.36 (m, 1H), 2.08–2.20 (m, 1H), 1.96–2.06 (m, 1H), 1.84–1.94 (m, 1H), 1.65–1.84 (m, 5H), 1.24–1.54 (m, 16H), 1.18 (s, 3H), 1.14 (s, 3H), 1.10 (s, 3H), 0.93 (d, J = 6.0 Hz, 3H), 0.69 (s, 3H). ^{13}C NMR (125 MHz, $CDCl_3$) δ 153.33, 134.90, 123.65, 118.07, 76.12, 74.04, 70.66, 56.22, 55.32, 49.83, 42.68, 42.02, 41.46, 40.32, 39.45, 37.33, 36.88, 36.25, 35.66, 28.51, 27.28, 27.07, 26.14, 23.32, 22.13, 22.05, 21.33, 20.67, 18.69, 11.76. HRMS (ESI): calcd for $C_{30}H_{50}NaO_3$ [M+Na]$^+$, 481.3652, found 481.3719.

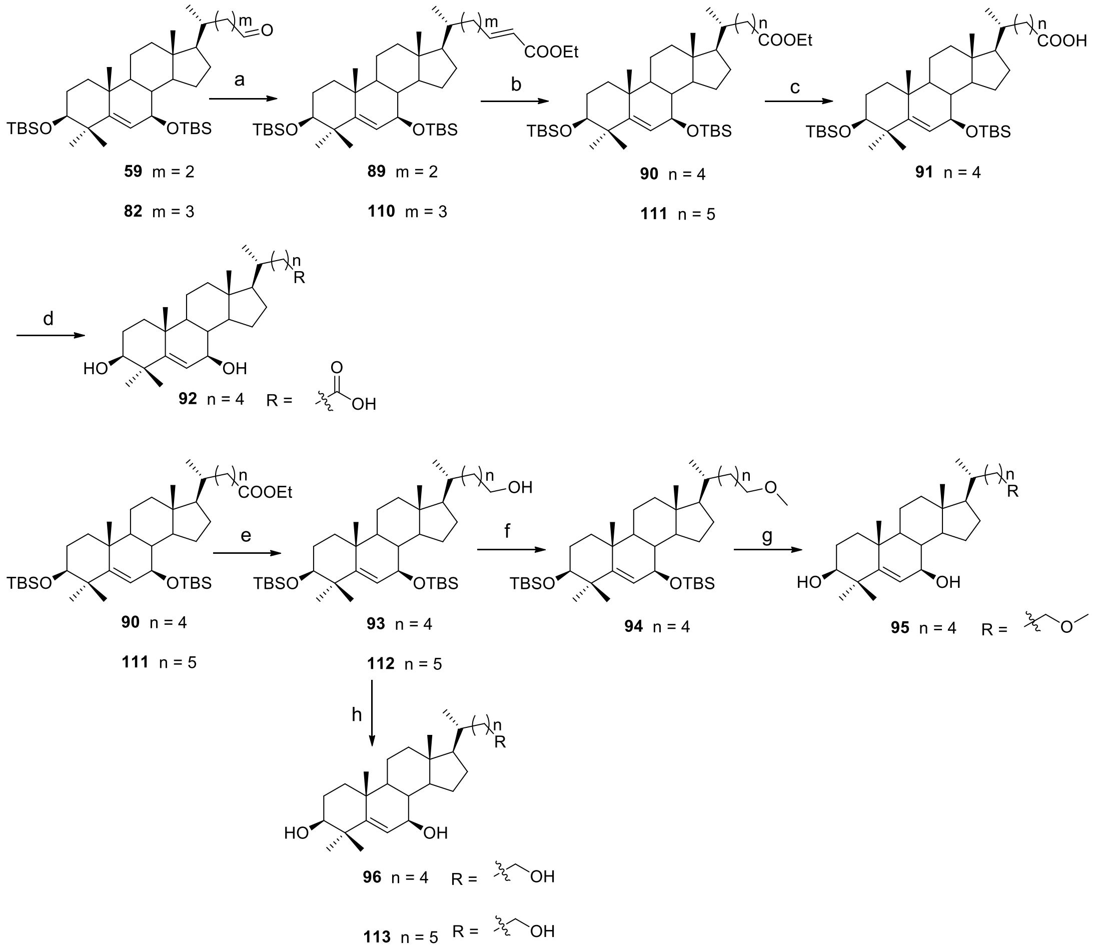

Supplementary Figure 137. The synthesis scheme of compounds 92, 95, 96 and 113. Reagents and conditions: (a) $Ph_3P{=}CHCOOC_2H_5$, toluene, reflux, 6 h, 95% for **89** and 96% for **110**; (b) 10% Pd/C, H_2, MeOH, r.t., 12 h; (c) K_2CO_3, MeOH, THF, r.t., 12 h, 81% for **91** (over two steps); (d) TBAF, THF, reflux, 24 h, 79% for **92**,, 91% for **95**; 75% for **96**, 78% for **113** (e) $LiAlH_4$, THF, r.t., 12 h, 97% for **93** and 92% for **112** (over two steps); (f) CH_3I, NaH, THF, r.t., 12 h, 92% for **94**.

Synthesis and characterization of compound 89

Compound **89**: To a solution of the $Ph_3P=CHCOOC_2H_5$ (696 mg, 2.0 mmol) in dry toluene (10 mL) was added compound **59** (631 mg, 1.0 mmol) under N_2. The reaction mixture was refluxed for 6 h and then concentrated. The residue was purified by silica gel chromatography (petroleum ether/AcOEt, 25/1 v/v) to give compound **89** (666 mg, 95%) as a white solid. ^{1}H NMR (500 MHz, $CDCl_3$) δ 6.90–7.01 (m, 1H), 5.80 (d, J = 15.6 Hz, 1H), 5.49 (d, J = 3.1 Hz, 1H), 4.18 (q, J = 7.1 Hz, 2H), 4.00 (dd, J = 3.2, 7.6 Hz, 1H), 3.21 (dd, J = 4.1, 11.4 Hz, 1H), 2.20–2.30 (m, 1H), 2.03–2.14 (m, 1H), 1.93–2.01 (m, 1H), 1.70–1.87 (m, 3H), 1.62–1.69 (m, 1H), 1.32–1.49 (m, 7H), 1.24–1.32 (m, 10H), 1.12 (s, 3H), 1.08 (s, 3H), 1.05 (s, 3H), 0.93 (d, J = 6.5 Hz, 3H), 0.89 (s, 9H), 0.88 (s, 9H), 0.67 (s, 3H), 0.08 (s, 3H), 0.07 (s, 3H), 0.04 (s, 3H), 0.02 (s, 3H).

Synthesis and characterization of compound 110

By a similar procedure described for **89**, **110** was obtained as a white solid; yield: 96%. ^{1}H NMR (500 MHz, $CDCl_3$) δ 6.90–7.01 (m, 1H), 5.81 (d, J = 15.7 Hz, 1H), 5.49 (d, J = 3.1 Hz, 1H), 4.18 (q, J = 7.1 Hz, 2H), 3.99 (dd, J = 3.3, 7.5 Hz, 1H), 3.21 (dd, J = 4.3, 11.5 Hz, 1H), 2.08–2.23 (m, 2H), 1.94–2.00 (m, 1H), 1.78–1.85 (m, 1H), 1.69–1.78 (m, 2H), 1.63–1.69 (m, 1H), 1.46–1.54 (m, 3H), 1.32–1.46 (m, 6H), 1.29 (t, J = 7.1 Hz, 3H), 1.23–1.31 (m, 3H), 1.12 (s, 3H), 1.08 (s, 3H), 1.05 (s, 3H), 1.00–1.08 (m, 4H), 0.91 (d, J = 6.7 Hz, 3H), 0.89 (s, 9H), 0.88 (s, 9H), 0.67 (s, 3H), 0.08 (s, 3H), 0.07 (s, 3H), 0.04 (s, 3H), 0.02 (s, 3H).

Synthesis and characterization of compound 90

Compound **90**: To a solution of compound **89** (457 mg, 0.652 mmol) in dry MeOH (20 mL) 10% Pd on carbon (90 mg) was added under N_2. The reaction mixture was subjected to 1 atm of H_2 and stirred for 12 h at room temperature. The reaction mixture was filtered and concentrated to give compound **90** (449 mg, 98%) as a white solid, which was used without further purification. ^{1}H NMR (500 MHz, $CDCl_3$) δ 5.49 (d, J = 3.1 Hz, 1H), 4.13 (q, J = 7.1 Hz, 2H), 3.99 (dd, J = 3.1, 7.4 Hz, 1H), 3.21 (dd, J = 4.0, 11.5 Hz, 1H), 2.29 (t, J = 7.6 Hz, 2H), 1.93–2.00 (m, 1H), 1.78–1.86 (m, 1H), 1.70–1.78 (m, 2H), 1.59–1.69 (m, 2H), 1.49–1.55 (m, 2H), 1.30–1.43 (m, 6H),

1.26 (t, J = 7.1 Hz, 3H), 1.21−1.29 (m, 3H), 1.12 (s, 3H), 1.08 (s, 3H), 1.05 (s, 3H), 0.99−1.08 (m, 6H), 0.89 (s, 9H), 0.88 (s, 9H), 0.86–0.91 (m, 3H), 0.67 (s, 3H), 0.08 (s, 3H), 0.07 (s, 3H), 0.04 (s, 3H), 0.02 (s, 3H).

Synthesis and characterization of compound 111

By a similar procedure described for **90**, **111** was obtained as a white solid; yield: 99%. ^{1}H NMR (500 MHz, $CDCl_3$) δ 5.49 (d, J = 3.1 Hz, 1H), 4.12 (q, J = 7.1 Hz, 2H), 4.00 (dd, J = 3.1, 7.5 Hz, 1H), 3.21 (dd, J = 4.1, 11.5 Hz, 1H), 2.28 (t, J = 7.5 Hz, 2H), 1.94−2.01 (m, 1H), 1.78−1.86 (m, 1H), 1.69−1.78 (m, 2H), 1.59−1.69 (m, 3H), 1.43−1.56 (m, 3H), 1.31−1.40 (m, 6H), 1.26 (t, J = 7.1 Hz, 3H), 1.21−1.30 (m, 4H), 1.12 (s, 3H), 1.08 (s, 3H), 1.05 (s, 3H), 1.01−1.09 (m, 5H), 0.87–0.91 (m, 3H), 0.89 (s, 9H), 0.88 (s, 9H), 0.67 (s, 3H), 0.08 (s, 3H), 0.07 (s, 3H), 0.04 (s, 3H), 0.02 (s, 3H).

Synthesis and characterization of compound 91

Compound **91**: To a solution of compound **90** (170 mg, 0.24 mmol) in MeOH-THF mixed solvent (20 mL, 1:1) was added K_2CO_3 (44 mg, 0.32 mmol). The reaction mixture was stirred for 12 h at room temperature and then concentrated. H_2O (20 mL) and CH_2Cl_2 (30 mL) were added to the residue and the PH value was adjusted to 3 with 1 M HCl. After the separation of organic layer, the aqueous phase was extracted with CH_2Cl_2 (20 mL × 3). The combined organic extract was washed with brine, dried over anhydrous Na_2SO_4 and concentrated. The residue was purified by silica gel chromatography (petroleum ether/AcOEt, 5/1 v/v) to give compound **91** (139 mg, 85%) as a white solid. ^{1}H NMR (500 MHz, $CDCl_3$) δ 5.49 (d, J = 3.1 Hz, 1H), 4.00 (dd, J = 3.0, 7.5 Hz, 1H), 3.21 (dd, J = 4.0, 11.5 Hz, 1H), 2.35 (td, J = 2.4, 7.8 Hz, 2H), 1.94−2.01 (m, 1H), 1.78−1.87 (m, 1H), 1.69−1.78 (m, 2H), 1.60−1.69 (m, 2H), 1.50−1.60 (m, 3H), 1.31−1.49 (m, 7H), 1.18−1.31 (m, 3H), 1.12 (s, 3H), 1.08 (s, 3H), 1.05 (s, 3H), 1.01−1.09 (m, 4H), 0.87–0.91 (m, 3H), 0.90 (s, 9H), 0.88 (s, 9H), 0.67 (s, 3H), 0.08 (s, 3H), 0.07 (s, 3H), 0.04 (s, 3H), 0.02 (s, 3H).

Synthesis and characterization of compound 92

By a similar procedure described for **55**, **92** was obtained as a white solid; yield: 79%. ^{1}H NMR (500 MHz, DMSO-d_6) δ 11.95 (brs, 1H), 5.36 (d, J = 2.8 Hz, 1H), 4.48-4.55 (m, 1H), 4.10–4.20 (m, 1H), 3.56–3.64 (m, 1H), 2.95–3.06 (m, 1H), 2.19 (t, J = 7.3

Hz, 2H), 1.90−1.96 (m, 1H), 1.71−1.81 (m, 2H), 1.57−1.68 (m, 2H), 1.38−1.57 (m, 5H), 1.28−1.38 (m, 6H), 1.09−1.25 (m, 3H), 1.06 (s, 6H), 0.96−1.03 (m, 4H), 0.98 (s, 3H), 0.88 (d, J = 6.4 Hz, 3H), 0.63 (s, 3H). ^{13}C NMR (125 MHz, DMSO-d_6) δ 174.48, 150.90, 125.06, 75.23, 72.07, 56.30, 55.03, 49.60, 42.14, 41.01, 39.34, 39.17, 36.27, 36.04, 35.22, 35.12, 33.73, 28.12, 27.29, 27.13, 25.84, 25.03, 24.98, 23.69, 20.87, 20.39, 18.60, 11.66. HRMS (ESI): calcd for $C_{28}H_{46}NaO_4$ [M+Na]$^+$, 469.3288, found 469.3291.

Synthesis and characterization of compound 93

By a similar procedure described for **39**, **93** and **112** were obtained as white solids.

Compound **93**: yield: 97%. ^{1}H NMR (500 MHz, $CDCl_3$) δ 5.49 (d, J = 2.9 Hz, 1H), 4.00 (dd, J = 2.8, 7.3 Hz, 1H), 3.64 (t, J = 6.6 Hz, 2H), 3.21 (dd, J = 3.9, 11.3 Hz, 1H), 1.94–2.01 (m, 1H), 1.78−1.88 (m, 1H), 1.69−1.78 (m, 2H), 1.63−1.69 (m, 1H), 1.43−1.55 (m, 3H), 1.31−1.43 (m, 8H), 1.27−1.31 (m, 2H), 1.16−1.23 (m, 2H), 1.12 (s, 3H), 1.08 (s, 3H), 1.05 (s, 3H), 1.01−1.09 (m, 5H), 0.88–0.91 (m, 3H), 0.90 (s, 9H), 0.88 (s, 9H), 0.67 (s, 3H), 0.08 (s, 3H), 0.07 (s, 3H), 0.04 (s, 3H), 0.02 (s, 3H).

Characterization of compound 112

Compound **112**: yield: 93%. ^{1}H NMR (500 MHz, $CDCl_3$) δ 5.49 (d, J = 3.2 Hz, 1H), 4.00 (dd, J = 3.1, 7.4 Hz, 1H), 3.60−3.68 (m, 2H), 3.21 (dd, J = 4.2, 11.5 Hz, 1H), 1.95−2.00 (m, 1H), 1.78−1.86 (m, 1H), 1.70−1.78 (m, 2H), 1.63−1.69 (m, 1H), 1.43−1.53 (m, 2H), 1.14−1.42 (m, 15H), 1.12 (s, 3H), 1.08 (s, 3H), 1.05 (s, 3H), 1.00−1.09 (m, 5H), 0.87–0.91 (m, 3H), 0.89 (s, 9H), 0.88 (s, 9H), 0.67 (s, 3H), 0.08 (s, 3H), 0.07 (s, 3H), 0.04 (s, 3H), 0.02 (s, 3H).

Characterization of compound 94

By a similar procedure described for **54**, **94** was obtained as a white solid; yield: 92%. ^{1}H NMR (500 MHz, $CDCl_3$) δ 5.49 (d, J = 3.0 Hz, 1H), 4.00 (dd, J = 3.0, 7.4 Hz, 1H), 3.36 (t, J = 6.7 Hz, 2H), 3.33 (s, 3H), 3.21 (dd, J = 4.0, 11.5 Hz, 1H), 1.96−1.98 (m, 1H), 1.83−1.93 (m, 1H), 1.70−1.82 (m, 2H), 1.64−1.67 (m, 1H), 1.45−1.60 (m, 8H), 1.24−1.37 (m, 12H), 1.12 (s, 3H), 1.08 (s, 3H), 1.05 (s, 3H), 0.89 (s, 9H), 0.88 (s, 9H), 0.88-0.91 (m, 3H), 0.67 (s, 3H), 0.08 (s, 3H), 0.07 (s, 3H), 0.04 (s, 3H), 0.02 (s, 3H).

Characterization of compound 95

By a similar procedure described for **55**, **95** was obtained as a white solid; yield: 91%. ^{1}H NMR (500 MHz, $CDCl_3$) δ 5.52 (d, J = 2.9 Hz, 1H), 3.91 (dd, J = 2.9, 7.1 Hz, 1H), 3.37 (t, J = 6.7 Hz, 2H), 3.33 (s, 3H), 3.26 (dd, J = 4.2, 10.7 Hz, 1H), 1.98–2.05 (m, 1H), 1.83−1.93 (m, 1H), 1.70−1.82 (m, 4H), 1.61−1.69 (m, 3H), 1.53−1.60 (m, 2H), 1.24−1.52 (m, 14H), 1.18 (s, 3H), 1.14 (s, 3H), 1.10 (s, 3H), 0.91 (d, J = 6.4 Hz, 3H), 0.68 (s, 3H). ^{13}C NMR (125 MHz, $CDCl_3$) δ 153.26, 123.62, 77.05, 73.99, 72.94, 58.49, 56.18, 55.35, 49.79, 42.63, 41.42, 40.25, 39.41, 36.83, 36.22, 35.84, 35.60, 29.67, 28.48, 27.23, 27.04, 26.54, 26.11, 25.92, 23.31, 21.29, 20.63, 18.67, 11.73. HRMS (ESI): calcd for $C_{29}H_{50}NaO_3$ [M+Na]$^+$, 469.3652, found 469.3646.

Synthesis and characterization of compound 96

By a similar procedure described for **55**, **96** and **113** were obtained as white solids.

Compound **96**: yield: 75%. ^{1}H NMR (500 MHz, DMSO-d_6) δ 5.36 (d, J = 2.2 Hz, 1H), 4.47–4.55 (m, 1H), 4.31 (t, J = 5.1 Hz, 1H), 4.12–4.19 (m, 1H), 3.58–3.66 (m, 1H), 3.35–3.39 (m, 2H), 2.96–3.04 (m, 1H), 1.90−1.97 (m, 1H), 1.71−1.81 (m, 2H), 1.57−1.69 (m, 2H), 1.49−1.57 (m, 1H), 1.12−1.48 (m, 17H), 1.06 (s, 6H), 1.00−1.03 (m, 2H), 0.98 (s, 3H), 0.89 (d, J = 6.2 Hz, 3H), 0.63 (s, 3H). ^{13}C NMR (125 MHz, DMSO-d_6) δ 150.84, 125.00, 75.18, 72.02, 60.69, 56.25, 55.06, 49.57, 42.09, 40.95, 40.00, 39.12, 36.22, 36.00, 35.52, 35.15, 32.56, 28.08, 27.24, 27.08, 25.94, 25.78, 25.40, 23.62, 20.82, 20.33, 18.60, 11.61. HRMS (ESI): calcd for $C_{28}H_{48}NaO_3$ [M+Na]$^+$, 455.3496, found 455.3500.

Characterization of compound 113

Compound **113**: yield: 78%. ^{1}H NMR (500 MHz, DMSO-d_6) δ 5.37 (d, J = 2.5 Hz, 1H), 4.37–4.43 (m, 1H), 4.18–4.23 (m, 1H), 3.99–4.08 (m, 1H), 3.59–3.67 (m, 1H), 3.33–3.43 (m, 2H), 2.97–3.06 (m, 1H), 1.90−1.97 (m, 1H), 1.72−1.82 (m, 2H), 1.58−1.69 (m, 2H), 1.51−1.58 (m, 1H), 1.13−1.47 (m, 17H), 1.07 (s, 6H), 0.97−1.05 (m, 4H), 0.99 (s, 3H), 0.89 (d, J = 6.3 Hz, 3H), 0.64 (s, 3H). ^{13}C NMR (125 MHz, DMSO-d_6) δ 150.77, 124.93, 75.11, 71.94, 60.61, 56.17, 54.97, 49.50, 42.01, 40.87, 39.13, 39.06, 36.15, 35.92, 35.36, 35.08, 32.42, 29.33, 27.97, 27.16, 27.00, 25.68,

25.43, 25.41, 23.52, 20.74, 20.24, 18.52, 11.53. HRMS (ESI): calcd for $C_{29}H_{50}NaO_3$ $[M+Na]^+$, 469.3652, found 469.3643.

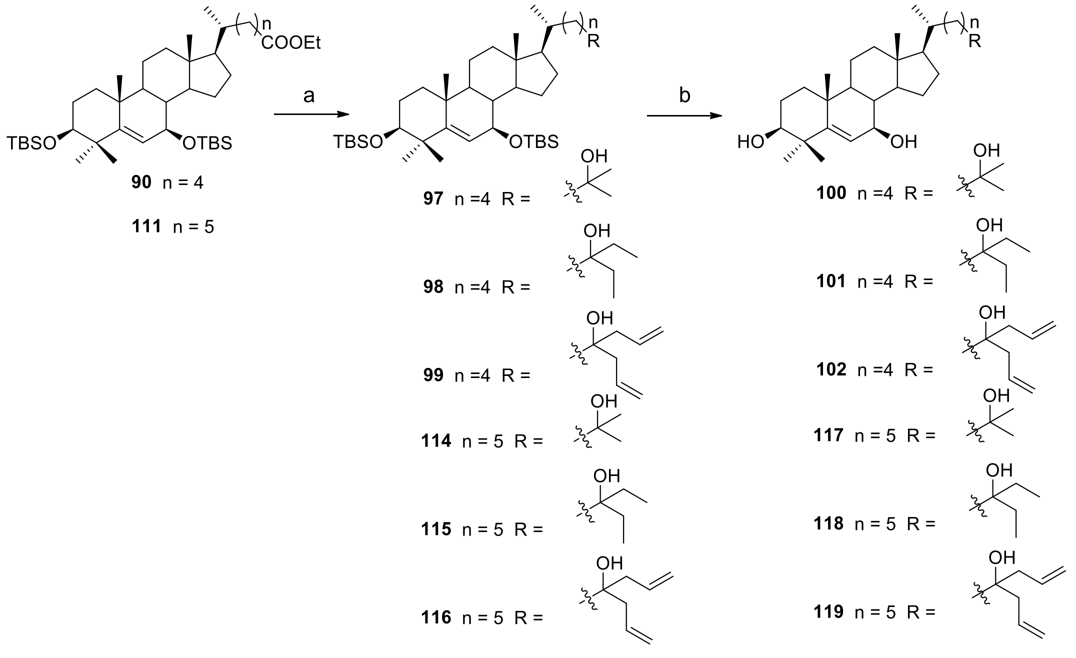

Supplementary Figure 138. The synthesis scheme of compounds 100-102 and 117- 119. Reagents and conditions: (a) CH_3MgCl (1.0 M in THF) or C_2H_5MgCl (2.0 M in THF) or C_3H_5MgCl (1.0 M in THF), THF, r.t., 2 h, 75-90% for **97- 99** and **114-116**); (c) TBAF, THF, reflux, 24 h, 78- 85% for **100- 102** and **117- 119**.

Synthesis and characterization of compound 97

By a similar procedure described for **56**, **97–99** and **114–116** were obtained as white solids. Compound **97**: yield: 75%. ^{1}H NMR (500 MHz, $CDCl_3$) δ 5.49 (d, J = 3.1 Hz, 1H), 4.00 (dd, J = 2.9, 7.5 Hz, 1H), 3.21 (dd, J = 4.1, 11.5 Hz, 1H), 1.93–2.01 (m, 1H), 1.78–1.87 (m, 1H), 1.70–1.78 (m, 2H), 1.63–1.69 (m, 1H), 1.43–1.53 (m, 4H), 1.31–1.42 (m, 8H), 1.27–1.30 (m, 2H), 1.21 (s, 6H), 1.12 (s, 3H), 1.08 (s, 3H), 1.05 (s, 3H), 0.99–1.09 (m, 6H), 0.87–0.90 (m, 3H), 0.89 (s, 9H), 0.88 (s, 9H), 0.67 (s, 3H), 0.08 (s, 3H), 0.07 (s, 3H), 0.04 (s, 3H), 0.02 (s, 3H).

Characterization of compound 98

Compound **98**: yield: 88%. ^{1}H NMR (500 MHz, $CDCl_3$) δ 5.49 (d, J = 3.0 Hz, 1H), 4.00 (dd, J = 2.9, 7.4 Hz, 1H), 3.21 (dd, J = 4.0, 11.5 Hz, 1H), 1.95–2.01 (m, 1H), 1.78–1.87 (m, 1H), 1.70–1.78 (m, 2H), 1.63–1.68 (m, 1H), 1.49–1.59 (m, 2H),

1.42–1.49 (m, 6H), 1.31–1.42 (m, 8H), 1.15–1.22 (m, 2H), 1.12 (s, 3H), 1.08 (s, 3H), 1.08 (s, 3H), 1.00–1.09 (m, 6H), 0.85–0.90 (m, 9H), 0.90 (s, 9H), 0.88 (s, 9H), 0.67 (s, 3H), 0.08 (s, 3H), 0.07 (s, 3H), 0.04 (s, 3H), 0.02 (s, 3H).

Characterization of compound 99

Compound **99**: yield: 81%. ^{1}H NMR (500 MHz, $CDCl_3$) δ 5.79–5.91 (m, 2H), 5.49 (d, J = 3.1 Hz, 1H), 5.08–5.18 (m, 4H), 4.00 (dd, J = 3.0, 7.4 Hz, 1H), 3.21 (dd, J = 4.0, 11.5 Hz, 1H), 2.22 (d, J = 7.0 Hz, 4H), 1.94–2.02 (m, 1H), 1.78–1.87 (m, 1H), 1.69–1.77 (m, 2H), 1.63–1.68 (m, 1H), 1.51–1.55 (m, 2H), 1.25–1.47 (m, 13H), 1.12 (s, 3H), 1.08 (s, 3H), 1.05 (s, 3H), 0.99–1.08 (m, 5H), 0.89 (s, 9H), 0.88 (s, 9H), 0.88–0.91 (m, 3H), 0.67 (s, 3H), 0.08 (s, 3H), 0.07 (s, 3H), 0.04 (s, 3H), 0.02 (s, 3H).

Characterization of compound 114

Compound **114**: yield: 90%. ^{1}H NMR (500 MHz, $CDCl_3$) δ 5.49 (d, J = 3.2 Hz, 1H), 4.00 (dd, J = 3.2, 7.5 Hz, 1H), 3.21 (dd, J = 4.2, 11.5 Hz, 1H), 1.95–2.01 (m, 1H), 1.78–1.87 (m, 1H), 1.69–1.78 (m, 2H), 1.63–1.69 (m, 1H), 1.49–1.58 (m, 2H), 1.43–1.49 (m, 3H), 1.30–1.41 (m, 8H), 1.23–1.30 (m, 4H), 1.21 (s, 6H), 1.12 (s, 3H), 1.08 (s, 3H), 1.05 (s, 3H), 1.02-1.10 (m, 5H), 0.91–0.92 (m, 3H), 0.89 (s, 9H), 0.88 (s, 9H), 0.67 (s, 3H), 0.08 (s, 3H), 0.07 (s, 3H), 0.04 (s, 3H), 0.02 (s, 3H).

Characterization of compound 115

Compound **115**: yield: 78%. ^{1}H NMR (500 MHz, $CDCl_3$) δ 5.49 (d, J = 2.7 Hz, 1H), 4.00 (dd, J = 2.5, 7.0 Hz, 1H), 3.21 (dd, J = 3.9, 11.4 Hz, 1H), 1.95–2.01 (m, 1H), 1.78–1.87 (m, 1H), 1.69–1.77 (m, 2H), 1.63–1.69 (m, 1H), 1.51–1.60 (m, 4H), 1.43–1.48 (m, 5H), 1.34–1.41 (m, 9H), 1.23–1.31 (m, 8H), 1.12 (s, 3H), 1.08 (s, 3H), 1.05 (s, 3H), 0.84–0.91 (m, 9H), 0.89 (s, 9H), 0.88 (s, 9H), 0.67 (s, 3H), 0.08 (s, 3H), 0.07 (s, 3H), 0.04 (s, 3H), 0.02 (s, 3H).

Characterization of compound 116

Compound **116**: yield: 80%. ^{1}H NMR (500 MHz, $CDCl_3$) δ 5.80–5.92 (m, 2H), 5.49 (d, J = 2.1 Hz, 1H), 5.08–5.18 (m, 4H), 4.00 (dd, J = 2.3, 7.0 Hz,1H), 3.21 (dd, J = 3.5, 11.3 Hz, 1H), 2.23 (d, J = 6.7 Hz, 4H), 1.94–2.01 (m, 1H), 1.78–1.87 (m, 1H), 1.69–1.78 (m, 2H), 1.62–1.69 (m, 1H), 1.49–1.56 (m, 3H), 1.40–1.48 (m, 4H),

1.31−1.40 (m, 8H), 1.15−1.30 (m, 7H), 1.12 (s, 3H), 1.07 (s, 3H), 1.05 (s, 3H), 0.88–0.89 (m, 3H), 0.89 (s, 9H), 0.88 (s, 9H), 0.67 (s, 3H), 0.08 (s, 3H), 0.07 (s, 3H), 0.04 (s, 3H),0.02 (s, 3H).

Characterization of compound 100

By a similar procedure described for **55**, **100–102** and **117–119** were obtained as white solids.

Compound **100**: yield: 80%. ^{1}H NMR (500 MHz, $CDCl_3$) δ 5.52 (d, J = 3.0 Hz, 1H), 3.91 (dd, J = 2.9, 7.9 Hz, 1H), 3.26 (dd, J = 4.7, 10.8 Hz, 1H), 1.98–2.04 (m, 1H), 1.85−1.94 (m, 1H), 1.69−1.84 (m, 4H), 1.25−1.52 (m, 15H), 1.21 (s, 6H), 1.18 (s, 3H), 1.14 (s, 3H), 1.10 (s, 3H), 1.04−1.11 (m, 4H), 0.92 (d, J = 6.6 Hz, 3H), 0.69 (s, 3H). ^{13}C NMR (125 MHz, $CDCl_3$+CD_3OD-d_4) δ 152.55, 123.56, 76.23, 72.95, 70.16, 56.06, 55.09, 49.68, 43.36, 42.16, 40.91, 39.14, 39.12, 36.30, 35.97, 35.53, 35.31, 28.01(2C), 27.94, 26.39, 26.34, 26.24, 25.41, 24.36, 22.76, 20.46, 20.27, 18.10, 11.12. HRMS (ESI): calcd for $C_{30}H_{52}NaO_3$ $[M+Na]^+$, 483.3809, found 483.3807.

Characterization of compound 101

Compound **101**: yield: 82%. ^{1}H NMR (500 MHz, $CDCl_3$) δ 5.52 (d, J = 2.7 Hz, 1H), 3.90 (dd, J = 2.4, 7.8 Hz, 1H), 3.26 (dd, J = 4.8, 11.1 Hz, 1H), 1.98–2.04 (m, 1H), 1.84–1.94 (m, 1H), 1.69–1.84 (m, 4H), 1.28–1.52 (m, 19H), 1.18 (s, 3H), 1.14 (s, 3H), 1.05–1.12 (m, 7H), 0.92 (d, J = 6.5 Hz, 3H), 0.86 (t, J = 7.5 Hz, 6H), 0.69 (s, 3H). ^{13}C NMR (125 MHz, $CDCl_3$) δ 153.30, 123.65, 77.08, 74.62, 74.02, 56.22, 55.40, 49.83, 42.66, 41.45, 40.29, 39.46, 38.25, 36.86, 36.25, 35.96, 35.67, 31.01, 30.96, 28.51, 27.26, 27.06, 26.74, 26.13, 23.83, 23.31, 21.31, 20.66, 18.70, 11.75, 7.76 (2C). HRMS (ESI): calcd for $C_{32}H_{56}NaO_3$ $[M+Na]^+$, 511.4122, found 511.4127.

Characterization of compound 102

Compound **102**: yield: 85%. ^{1}H NMR (500 MHz, $CDCl_3$) δ 5.80–5.91 (m, 2H), 5.52 (d, J = 2.9 Hz, 1H), 5.07–5.19 (m, 4H), 3.91 (dd, J = 1.7, 7.3 Hz, 1H), 3.26 (dd, J = 4.6, 10.7 Hz, 1H), 2.23 (d, J = 6.7 Hz, 4H), 1.97–2.05 (m, 1H), 1.84–1.93 (m, 1H), 1.69–1.83 (m, 5H), 1.24–1.52 (m, 18H), 1.18 (s, 3H), 1.14 (s, 3H), 1.10 (s, 3H), 0.91 (d, J = 6.4 Hz, 3H), 0.69 (s, 3H). ^{13}C NMR (125 MHz, $CDCl_3$) δ 153.31, 133.80 (2C),

123.64, 118.56 (2C), 77.10, 74.03, 73.45, 56.21, 55.38, 49.82, 43.69, 43.64, 42.66, 41.45, 40.31, 39.45, 39.23, 36.87, 36.25, 35.89, 35.63, 28.50, 27.27, 27.06, 26.58, 26.13, 23.79, 23.31, 21.32, 20.66, 18.68, 11.76. HRMS (ESI): calcd for $C_{34}H_{56}NaO_3$ $[M+Na]^+$, 535.4122, found 535.4121.

Characterization of compound 117

Compound **117**: yield: 78%. ^{1}H NMR (500 MHz, $CDCl_3$) δ 5.52 (d, J = 3.0 Hz, 1H), 3.91 (dd, J = 2.0, 6.9 Hz, 1H), 3.26 (dd, J = 4.2, 10.4 Hz, 1H), 1.98–2.04 (m, 1H), 1.84−1.94 (m, 1H), 1.66−1.84 (m, 4H), 1.31−1.52 (m, 16H), 1.22−1.31 (m, 5H), 1.21 (s, 6H), 1.18 (s, 3H), 1.14 (s, 3H), 1.10 (s, 3H), 0.92 (d, J = 6.5 Hz, 3H), 0.69 (s, 3H). ^{13}C NMR (125 MHz, DMSO-d_6) δ 150.82, 125.01, 75.17, 72.02, 68.68, 56.25, 55.04, 49.56, 43.69, 42.08, 40.95, 39.12, 36.21 36.00, 35.50, 35.20, 30.32, 29.22 (3C), 28.09, 27.24, 27.09, 25.79, 25.56, 23.90, 23.63, 20.82, 20.34, 18.61, 11.61. HRMS (ESI): calcd for $C_{31}H_{54}NaO_3$ $[M+Na]^+$, 497.3965, found 497.3987.

Characterization of compound 118

Compound **118**: yield: 80%. ^{1}H NMR (500 MHz, $CDCl_3$) δ 5.52 (d, J = 3.1 Hz, 1H), 3.90 (dd, J = 2.8, 7.8 Hz, 1H), 3.26 (dd, J = 4.6, 10.8 Hz, 1H), 1.98–2.04 (m, 1H), 1.85−1.93 (m, 1H), 1.56−1.83 (m, 7H), 1.31−1.52 (m, 17H), 1.23−1.31 (m, 5H), 1.18 (s, 3H), 1.14 (s, 3H), 1.10 (s, 3H), 0.91 (d, J = 6.5 Hz, 3H), 0.86 (t, J = 7.5 Hz, 6H), 0.69 (s, 3H). ^{13}C NMR (125 MHz, $CDCl_3$) δ 153.28, 123.65, 77.06, 74.60, 74.00, 56.21, 55.39, 49.82, 42.64, 41.43, 40.28, 39.44, 38.16, 36.85, 36.25, 35.89, 35.64, 30.98 (2C), 30.72, 28.50, 27.25, 27.06, 26.12, 26.02, 23.39, 23.31, 21.30, 20.65, 18.71, 11.73, 7.74 (2C). HRMS (ESI): calcd for $C_{33}H_{58}NaO_3$ $[M+Na]^+$, 525.4278, found 525.4332.

Characterization of compound 119

Compound **119**: yield: 83%. ^{1}H NMR (500 MHz, $CDCl_3$) δ 5.81−5.89 (m, 2H), 5.52 (d, J = 2.9 Hz, 1H), 5.10−5.15 (m, 4H), 3.91 (dd, J = 2.5, 7.7 Hz, 1H), 3.26 (dd, J = 4.5, 10.7 Hz, 1H), 2.23 (d, J = 6.4 Hz, 4H), 1.98–2.04 (m, 1H), 1.84−1.93 (m, 1H), 1.70−1.83 (m, 4H), 1.31−1.49 (m, 16H), 1.27−1.31 (m, 5H), 1.18 (s, 3H), 1.14 (s, 3H), 1.10 (s, 3H), 0.91 (d, J = 6.4 Hz, 3H), 0.69 (s, 3H). ^{13}C NMR (125 MHz, $CDCl_3$) δ 153.26, 133.73 (2C), 123.57, 118.49 (2C), 77.06, 73.98, 73.40, 56.15, 55.34, 49.76,

43.60 (2C), 42.60, 41.39, 40.25, 39.39, 39.10, 36.80, 36.18, 35.81, 35.59, 30.52, 28.45, 27.20, 26.99, 26.10, 25.93, 23.32, 23.24, 21.26, 20.60, 18.64, 11.68. HRMS (ESI): calcd for $C_{35}H_{58}NaO_3$ $[M+Na]^+$, 549.4278, found 549.4299.

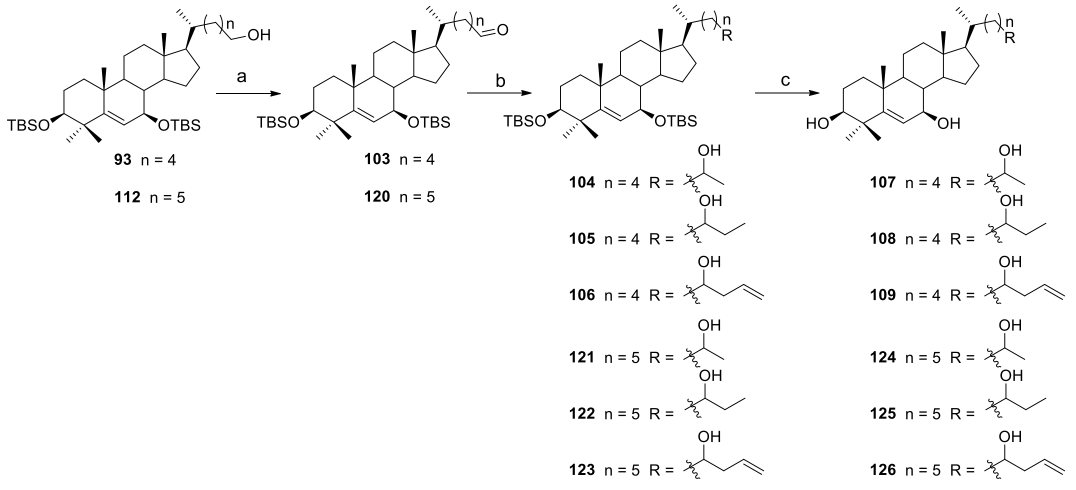

Supplementary Figure 139. The synthesis scheme of compounds 107-109 and 124-126. Reagents and conditions: (a) IBX, DMSO, 50°C, 3 h, 87% for **103** and 88% for **120**; (b) CH_3MgCl (1.0 M in THF) or C_2H_5MgCl (2.0 M in THF) or C_3H_5MgCl (1.0 M in THF), THF, r.t., 2 h, 74-82% for **104- 106** and **121- 123**; TBAF, THF, reflux, 24 h, 70- 85% for **107- 109** and **124- 126**.

Characterization of compound 103

By a similar procedure described for **59**, **103** and **120** were obtained as white solids.

Compound **103**: yield: 87%. ^{1}H NMR (500 MHz, $CDCl_3$) δ 9.76 (s, 1H), 5.49 (d, J = 3.1 Hz, 1H), 3.99 (dd, J = 3.1, 7.4 Hz, 1H), 3.21 (dd, J = 4.0, 11.5 Hz, 1H), 2.42 (t, J = 7.3 Hz, 2H), 1.94–2.01 (m, 1H), 1.69-1.87 (m, 4H), 1.61–1.68 (m, 2H), 1.43–1.58 (m, 6H), 1.31–1.42 (m, 8H), 1.16–1.24 (m, 2H), 1.12 (s, 3H), 1.07 (s, 3H), 1.05 (s, 3H), 0.87–0.90 (m, 3H), 0.89 (s, 9H), 0.88 (s, 9H), 0.67 (s, 3H), 0.08 (s, 3H), 0.07 (s, 3H), 0.04(s, 3H), 0.02 (s, 3H).

Characterization of compound 120

Compound **120**: yield: 88%. ^{1}H NMR (400 MHz, $CDCl_3$) δ 9.76 (s, 1H), 5.49 (d, J = 3.2 Hz, 1H), 4.00 (dd, J = 3.2, 7.5 Hz, 1H), 3.21 (dd, J = 4.1, 11.4 Hz, 1H), 2.39–2.44 (m, 2H), 1.94–2.01 (m, 1H), 1.69–1.86 (m, 4H), 1.59–1.69 (m, 4H), 1.31–1.47 (m, 9H), 1.23–1.29 (m, 4H), 1.12 (s, 3H), 1.08 (s, 3H), 1.05 (s, 3H), 0.86-0.92 (m, 6H),

0.89 (s, 9H), 0.88 (s, 9H), 0.67 (s, 3H), 0.08 (s, 3H), 0.07 (s, 3H), 0.04 (s, 3H), 0.02 (s, 3H).

Characterization of compound 104

By a similar procedure described for **56**, **104–106** and **121–123** were obtained as white solids. Compound **104**: yield: 80%. ^{1}H NMR (500 MHz, $CDCl_3$) δ 5.49 (s, 1H), 4.00 (dd, J = 2.3, 7.1 Hz, 1H), 3.75–3.84 (m, 1H), 3.21 (dd, J = 3.4, 11.4 Hz, 1H), 1.95–2.01 (m, 1H), 1.72–1.85 (m, 4H), 1.63–1.67 (m, 1H), 1.41–1.60 (m, 10H), 1.28–1.34 (m, 4H), 1.18 (d, J = 6.1 Hz, 3H), 1.12 (s, 3H), 1.08 (s, 3H), 1.05 (s, 3H), 1.03–1.09 (m, 5H), 0.88–0.90 (m, 3H), 0.90 (s, 9H), 0.88 (s, 9H), 0.67 (s, 3H), 0.08 (s, 3H), 0.07 (s, 3H), 0.04 (s, 3H), 0.02 (s, 3H).

Characterization of compound 105

Compound **105**: yield: 78%. ^{1}H NMR (500 MHz, $CDCl_3$) δ 5.49 (d, J = 3.1 Hz, 1H), 4.00 (dd, J = 3.1, 7.5 Hz, 1H), 3.46–3.56 (m, 1H), 3.21 (dd, J = 4.1, 11.5 Hz, 1H), 1.94–2.00 (m, 1H), 1.69–1.86 (m, 3H), 1.59–1.68 (m, 1H), 1.31–1.58 (m, 17H), 1.12 (s, 3H), 1.08 (s, 3H), 1.05 (s, 3H), 0.99-1.10 (m, 5H), 0.93 (t, J = 7.5 Hz, 3H), 0.87-0.90 (m, 3H), 0.89 (s, 9H), 0.88 (s, 9H), 0.67 (s, 3H), 0.08 (s, 3H), 0.07 (s, 3H), 0.04 (s, 3H), 0.02 (s, 3H).

Characterization of compound 106

Compound **106**: yield: 76%. ^{1}H NMR (400 MHz, $CDCl_3$) δ 5.78–5.89 (m, 1H), 5.49 (d, J = 3.2 Hz, 1H), 5.12–5.16 (m, 2H), 4.00 (dd, J = 3.2, 7.5 Hz, 1H), 3.60–3.67 (m, 1H), 3.21 (dd, J = 4.1, 11.4 Hz, 1H), 2.26–2.34 (m, 1H), 2.09–2.18 (m, 1H), 1.96–1.99 (m, 1H), 1.62-1.88 (m, 5H), 1.54–1.57 (m, 2H), 1.44–1.52 (m, 4H), 1.32–1.41 (m, 7H), 1.26–1.29 (m, 1H), 1.12 (s, 3H), 1.08 (s, 3H), 1.05 (s, 3H), 1.03–1.05 (m, 2H), 0.86–0.92 (m, 24H), 0.67 (s, 3H), 0.08 (s, 3H), 0.07 (s, 3H), 0.04 (s, 3H), 0.03 (s, 3H).

Characterization of compound 121

Compound **121**: yield: 74%. ^{1}H NMR (500 MHz, $CDCl_3$) δ 5.49 (d, J = 3.2 Hz, 1H), 4.00 (dd, J = 3.2, 7.5 Hz, 1H), 3.76–3.84 (m, 1H), 3.22 (dd, J = 4.1, 11.5 Hz, 1H), 1.95–2.01 (m, 1H), 1.78–1.87 (m, 1H), 1.70–1.78 (m, 2H), 1.63–1.69 (m, 1H),

1.51–1.57 (m, 2H), 1.25–1.49 (m, 15H), 1.19 (d, *J* = 6.2 Hz, 3H), 1.13 (s, 3H), 1.08 (s, 3H), 1.05 (s, 3H), 1.01–1.09 (m, 5H), 0.90–0.91 (m, 3 H), 0.90 (s, 9H), 0.88 (s, 9H), 0.67 (s, 3H), 0.08 (s, 3H), 0.07 (s, 3H),0.04 (s, 3H), 0.03 (s, 3H).

Characterization of compound 122

Compound **122**: yield: 79%. ^{1}H NMR (500 MHz, $CDCl_3$) δ 5.49 (d, *J* = 2.1 Hz, 1H), 4.00 (dd, *J* = 2.8, 7.4 Hz,1H), 3.46–3.57 (m, 1H), 3.21 (dd, *J* = 3.7, 11.4 Hz, 1H), 1.94–2.02 (m, 1H), 1.78–1.87 (m, 1H), 1.69–1.78 (m, 2H), 1.64–1.67 (m, 1H), 1.24–1.55 (m, 22H), 1.12 (s, 3H), 1.08 (s, 3 H), 1.05 (s, 3H), 0.99–1.08 (m, 5H), 0.94 (t, *J* = 2.1 Hz, 3H), 0.89 (s, 9H), 0.88 (s, 9H), 0.67 (s, 3H), 0.08 (s, 3H), 0.07 (s, 3H), 0.04 (s, 3H),0.02 (s, 3H).

Characterization of compound 123

Compound **123**: yield: 82%. ^{1}H NMR (400 MHz, $CDCl_3$) δ 5.77–5.88 (m, 1H), 5.49 (d, *J* = 3.2 Hz, 1H), 5.10–5.17 (m, 2H), 4.00 (dd, *J* = 7.5, 3.2 Hz, 1H), 3.60–3.68 (m, 1H), 3.21 (dd, *J* = 4.1, 11.4 Hz, 1H), 2.26–2.34 (m, 1H), 2.09–2.19 (m, 1H), 1.94–2.01 (m, 1H), 1.63–1.87 (m, 5H), 1.49–1.55 (m, 2H), 1.40–1.43 (m, 4H), 1.24–1.35 (m, 10H), 1.12 (s, 3H), 1.08 (s, 3H), 1.04 (s, 3H), 1.02–1.04 (m, 2H), 0.84–0.94 (m, 24H), 0.67 (s, 3H), 0.08 (s, 3H), 0.07 (s, 3H), 0.04 (s, 3H), 0.02 (s, 3H).

Characterization of compound 107

By a similar procedure described for **56**, **107–109** and **117–119** were obtained as white solids. Compound **107**: yield: 73%. ^{1}H NMR (500 MHz, $CDCl_3$) δ 5.52 (d, *J* = 2.9 Hz, 1H), 3.90 (dd, *J* = 2.9, 7.8 Hz, 1H), 3.75–3.82 (m, 1H), 3.26 (dd, *J* = 4.8, 11.0 Hz, 1H), 1.98–2.03 (m, 1H), 1.84–1.93 (m, 1H), 1.63–1.84 (m, 5H), 1.29–1.52 (m, 18H), 1.19 (d, *J* = 6.3 Hz, 3H), 1.18 (s, 3H), 1.14 (s, 3H), 1.10 (s, 3H), 0.92 (d, *J* = 6.4 Hz, 3H), 0.69 (s, 3H). ^{13}C NMR (125 MHz, DMSO-d_6) δ 150.83, 125.02, 75.16, 72.01, 65.74, 56.24, 55.03, 49.55, 42.08, 40.95, 39.16 (2C), 36.22, 35.98, 35.54, 35.15, 28.11, 28.08, 27.24, 27.08, 25.83, 25.78, 25.65, 23.62 (2C), 20.82, 20.32, 18.60, 11.61. HRMS (ESI): calcd for $C_{29}H_{50}NaO_3$ $[M+Na]^+$, 469.3652, found 469.3674.

Characterization of compound 108

Compound **108**: yield: 75%. ^{1}H NMR (500 MHz, $CDCl_3$) δ 5.52 (d, J = 2.8 Hz, 1H), 3.90 (dd, J = 2.4, 7.5 Hz, 1H), 3.46–3.55 (m, 1H), 3.26 (dd, J = 4.5, 10.5 Hz, 1H), 1.98–2.03 (m, 1H), 1.84–1.92 (m, 1H), 1.66–1.84 (m, 5H), 1.21–1.62 (m, 15H), 1.18 (s, 3H), 1.14 (s, 3H), 1.10 (s, 3H), 1.06–1.15 (m, 5H), 0.90–0.95 (m, 6H), 0.69 (s, 3H). ^{13}C NMR (125 MHz, $CDCl_3$) δ 153.33, 123.67, 77.14, 74.06, 73.36, 56.24, 55.42, 49.85, 42.69, 41.47, 40.34, 39.47, 37.03, 36.89, 36.27, 35.94, 35.66, 30.16, 28.53, 27.29, 27.08, 26.19, 26.15, 26.09, 23.32, 21.34, 20.68, 18.73, 11.77, 9.87. HRMS (ESI): calcd for $C_{30}H_{52}NaO_3$ [M+Na]$^+$, 483.3809, found 483.3809.

Characterization of compound 109

Compound **109**: yield: 85%. ^{1}H NMR (400 MHz, $CDCl_3$) δ 5.77–5.90 (m, 1H), 5.52 (d, J = 2.8 Hz, 1H), 5.10–5.17 (m, 2H), 3.90 (dd, J = 2.3, 7.6 Hz, 1H), 3.61–3.68 (m, 1H), 3.26 (dd, J = 4.9， 10.4 Hz, 1H), 2.25–2.35 (m, 1H), 2.09–2.19 (m, 1H), 1.96–2.05 (m, 1H), 1.85–1.94 (m, 1H), 1.66–1.85 (m, 4H), 1.24–1.51 (m, 15H), 1.18 (s, 3H), 1.14 (d, J = 5.1 Hz, 3H), 1.10 (s, 3H), 0.97-1.09 (m, 4H), 0.92 (d, J = 6.4 Hz, 3H), 0.69 (s, 3H). ^{13}C NMR (100 MHz, $CDCl_3$) δ 152.44, 134.01, 122.79, 117.10, 76.23, 75.78, 73.15, 69.80, 55.36, 54.54, 48.98, 41.80, 41.05, 40.58, 39.45, 38.59, 36.00, 35.98, 35.39, 35.03, 34.75, 27.62, 26.41, 26.19, 25.25, 25.22, 22.42, 20.45, 19.79, 17.83, 10.88. HRMS (ESI): calcd for $C_{31}H_{52}NaO_3$ [M+Na]$^+$, 495.3809, found 495.3802.

Characterization of compound 124

Compound **124**: yield: 70%. ^{1}H NMR (400 MHz, $CDCl_3$) δ 5.51 (d, J = 3.1 Hz, 1H), 3.90 (dd, J = 2.8, 8.1 Hz, 1H), 3.75–3.81 (m, 1H), 3.25 (dd, J = 4.9, 10.3 Hz, 1H), 1.98–2.03 (m, 1H), 1.70–1.92 (m, 6H), 1.33–1.50 (m, 14H), 1.28–1.33 (m, 6H), 1.19 (d, J = 6.1 Hz, 3H), 1.17 (s, 3H), 1.14 (s, 3H), 1.10 (s, 3H), 0.91 (d, J = 6.4 Hz, 3H), 0.68 (s, 3H). ^{13}C NMR (100 MHz, CD_3OD-d_4) δ 154.05, 125.63, 77.92, 74.49, 68.60, 58.08, 57.05, 51.69, 43.85, 42.55, 41.00, 40.71, 40.27, 37.99, 37.75, 37.13, 31.84, 31.33, 30.81, 29.64, 28.05, 27.83, 27.23, 26.97, 24.18, 23.52, 21.94, 21.66, 19.35, 12.34. HRMS (ESI): calcd for $C_{30}H_{52}NaO_3$ [M+Na]$^+$, 483.3809, found 483.3803.

Characterization of compound 125

Compound **125**: yield: 72%. ^{1}H NMR (400 MHz, $CDCl_3$) δ 5.51 (d, J = 2.9 Hz, 1H), 3.90 (dd, J = 3.4, 7.5 Hz, 1H), 3.48–3.55 (m, 1H), 3.25 (dd, J = 5.1, 10.6 Hz, 1H), 1.97–2.05 (m, 1H), 1.85–1.92 (m, 1H), 1.69–1.85 (m, 5H), 1.25–1.51 (m, 22H), 1.17 (s, 3H), 1.14 (s, 3H), 1.10 (s, 3H), 0.88–0.95 (m, 6H), 0.68 (s, 3H). ^{13}C NMR (100 MHz, CD_3OD-d_4) δ 152.64, 124.21, 76.52, 73.08, 72.51, 56.67, 55.65, 50.29, 42.44, 41.14, 39.59, 39.31, 36.58, 36.34, 35.70, 30.42, 29.94, 29.62, 29.40, 28.21, 26.64, 26.42, 25.82, 25.67, 25.45, 22.75, 20.53, 20.25, 17.94, 10.93, 8.94. HRMS (ESI): calcd for $C_{31}H_{54}NaO_3$ $[M+Na]^+$, 497.3965, found 497.3990.

Characterization of compound 126

Compound **126**: yield: 81%. ^{1}H NMR (400 MHz, $CDCl_3$) δ 5.77–5.89 (m, 1H), 5.52 (d, J = 2.8 Hz, 1H), 5.10–5.17 (m, 2H), 3.90 (dd, J = 2.3, 7.5 Hz, 1H), 3.61–3.68 (m, 1H), 3.26 (dd, J = 4.8, 10.5 Hz, 1H), 2.25–2.35 (m, 1H), 2.09–2.19 (m, 1H), 1.96–2.06 (m, 1H), 1.85–1.94 (m, 1H), 1.70–1.85 (m, 4H), 1.24–1.52 (m, 19H), 1.18 (s, 3H), 1.14 (s, 3H), 1.10 (s, 3H), 1.05–1.09 (m, 2H), 0.91 (d, J = 6.4 Hz, 3H), 0.69 (s, 3H). ^{13}C NMR (100 MHz, $CDCl_3$ +CD_3OD(minor)-d_4) δ 153.70, 135.34, 124.05, 117.72, 77.74, 74.06, 71.17, 56.71, 55.85, 50.36, 43.01, 42.05, 41.77, 40.29, 39.86, 37.17, 36.99, 36.70, 36.26, 36.05, 30.48, 28.83, 27.29, 27.26, 26.39, 26.32, 25.99, 23.65, 21.49, 21.06, 19.02, 12.05. HRMS (ESI): calcd for $C_{32}H_{54}NaO_3$ $[M+Na]^+$, 509.3965, found 509.3976.
