# Supplementary material for: Discovery of a potent HMG-CoA reductase degrader that eliminates statin-induced reductase accumulation and lowers cholesterol
Source: Nat Commun. 2018 Dec 3;9:5138. doi: 10.1038/s41467-018-07590-3 (PMC6277434; doi:10.1038/s41467-018-07590-3)
Supplement: Supplementary file 3 — Description of Additional Supplementary Files [file 41467_2018_7590_MOESM3_ESM.pdf]

Description of Additional Supplementary Files

File Name: Supplementary Data 1
Description: Unprocessed images and raw data underlying the graphs and charts in this paper
